# May the Force (Field) Be with You: On the Importance of Conformational Searches in the Prediction of NMR Chemical Shifts

**DOI:** 10.3390/md20110699

**Published:** 2022-11-08

**Authors:** Cristina Cuadrado, Antonio Hernández Daranas, Ariel M. Sarotti

**Affiliations:** 1 Instituto de Productos Naturales y Agrobiología, Consejo Superior de Investigaciones Científicas (IPNA-CSIC), 38206 La Laguna, Tenerife, Spain; 2 Instituto de Química Rosario (CONICET), Facultad de Ciencias Bioquímicas y Farmacéuticas, Universidad Nacional de Rosario, Suipacha 531, Rosario 2000, Argentina

**Keywords:** structural elucidation, stereochemistry, nuclear magnetic resonance, quantum mechanical calculations, molecular mechanics force field

## Abstract

NMR data prediction is increasingly important in structure elucidation. The impact of force field selection was assessed, along with geometry and energy cutoffs. Based on the conclusions, we propose a new approach named mix-*J*-DP4, which provides a remarkable increase in the confidence level of complex stereochemical assignments—100% in our molecular test set—with a very modest increment in computational cost.

## 1. Introduction

Nuclear magnetic resonance (NMR) is the most commonly used spectroscopic technique for elucidating the structure of small organic compounds [1]. However, the intrinsic complexity of some molecular architectures makes this task problematic [2,3]. Over the past decade, the increasing ease of use and success of computational methods have popularized their use in structural elucidation tasks, extending their use to increasingly challenging molecules [4,5,6,7]. These methods generally rely on the use of NMR data calculated at DFT levels, followed by refined strategies to correlate them with experimental information [8,9,10]. Among them, Bayesian analysis (DP4, DP4+, and *J*-DP4) [11,12,13], parametric corrections (DU8+) [14], or conformational selection (CASE-3D) [15] methods stand out. More recently, new approaches based on machine learning have been developed to speed up the NMR calculation stage, including IMPRESSION, CASCADE, and ML-*J*-DP4 [16,17,18].

A common, and usually overlooked, step in all of these methods comprises the molecular mechanics (MM) conformational search that generates conformer ensembles for each candidate structure. MM-generated structures can be either used directly or after a DFT optimization step as inputs for NMR chemical shifts and *J* couplings calculations. However, it is important to note that optimization at high levels of theory (DFT) implies large computational times for complex structures with a high number of conformations or configurations [6,7,19]. Molecular mechanics force fields (FF) have undergone a significant evolution over the years, including different parametrizations of the explicit functions used to define the global energy of the system [20]. The MMFF force field has been proposed for performing the conformational searches in some of the most common metrics used for structure prediction, including DP4 and *J*-DP4 [11,13]. However, in a substantial number of publications, the conformational sampling has been carried out with other FFs or is simply not described [21,22,23,24,25,26,27]. This suggests that many users (and reviewers) do not give enough importance to this key step. The most obvious consequence of using a different FF is the conformational landscape that results from the different number and types of conformations found within a given cutoff value. This can be critical because missing important conformations may lead to erroneous results. In addition, the effect of subtle geometric differences in the predicted NMR parameters is unclear but potentially very important for the quality of the predictions.

## 2. Results and Discussion

**Figure 1:**
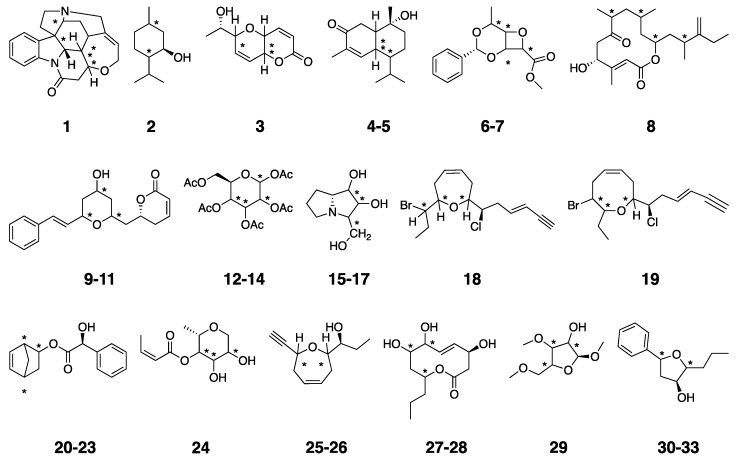
Test set of molecules used in this work. The number of possible candidate structures generated for each one depends on the number of carbon atoms marked with an asterisk, where configurations were changed.

In this report, we evaluated the impact of FF selection in the prediction of NMR data for structural elucidation within the DP4 [11] and *J*-DP4 [13] formalisms, using a test set of 33 challenging examples (see Supplementary Material). They showed a wide diversity of structures, conformational freedom, and many possible stereoisomers (Figure 1). The choice to confine the analysis to DP4/*J*-DP4 was based on the fact that both use molecular coordinates taken directly from the FF-optimized geometries for the subsequent DFT computations of NMR parameters and energies, and, therefore, a significant dependence on the FF selection should be expected. Other popular metrics that require prior geometry optimizations at DFT levels (for example, DP4+) are much less sensitive to the force field employed during the conformational sampling and were not considered in this study. Another relevant issue is that DP4 delivers the probability of a candidate structure being correct based on the analysis of errors of calculated versus experimental chemical shifts. The *J*-DP4 approach adds geometric (angular) information, obtained from measurements of ^3^*J*_HH_ values, into the DP4 formalism. ^3^*J*_HH_ values can be used either directly in d*J*-DP4 by including an additional probability term into a DP4-like equation or indirectly in the i*J*-DP4 approach, using ^3^*J*_HH_ to restrict the results of the conformational search. However, their combination (i*J*/d*J*-DP4) gives the best results.

In this study, we chose five representative and commonly used FFs: AMBER (A) and MM3 (B) [28,29], as they are good examples of “classic” FFs; MMFF (C), because DP4 and *J*-DP4 were developed using it [30]; and OPLS2005 (D) and OPLS4 (E), as they are two representative examples of modern FFs [31,32]. We also studied the effect of two important yet underestimated parameters that have a strong influence on the conformers ensemble composition, namely, the energy cutoff value and the geometric criterion to eliminate redundant conformers. Regarding the first, a value of 10 kJ/mol has been recommended as a tradeoff between CPU time and the risk of missing important conformations due to the inaccurate prediction of energies [11,12]. However, not only are a wide range of values reported (between 5 and 40 kJ/mol), but in a large number of papers, that important criterion is not described [23,24,33,34]. This trend is accentuated with the second parameter, as the criterion for defining duplicates is mostly unreported but deeply impacts the number of final conformations used for the DFT analysis. The most common ones are RMSD (root mean square deviations) and MAD (maximum atom deviation).

**Figure 2:**
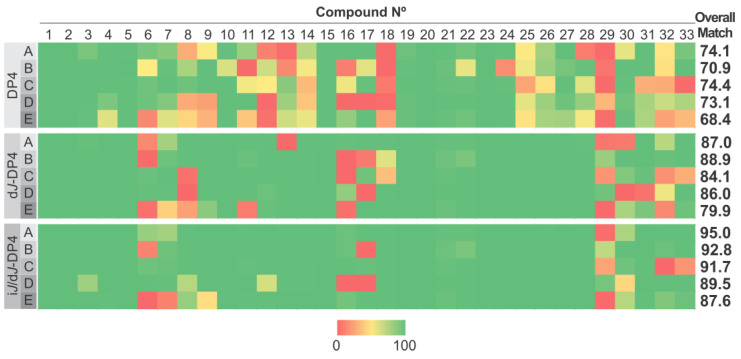
Performance of DP4 (**top**), d*J*-DP4 (**middle**), and i*J*/d*J*-DP4 (**bottom**) with the test set at the five FF studied (A: MM3, B: AMBER, C: MMFF, D: OPLS2005, E: OPLS4), using a 21 kJ/MAD 05 conformational criterion.

To understand the effect of FF selection on the classification outcome, we first evaluated DP4 and *J*-DP4, keeping all the resulting conformations within a 21 kJ/mol energy threshold with a maximum atom deviation (MAD) of 0.5 Å as represented in Figure 2. To simplify further discussions, this selection will be referred to as 21 kJ/MAD 05. Applying DP4 probability, which only uses information from NMR chemical shifts, MMFF showed the best results, with a 74.4% overall success, while, surprisingly, the modern OPLS4 force field showed the worst results, with a 68.4% overall success. These outcomes were improved by including ^3^*J*_HH_ information in the d*J*-DP4 approach, which showed the best results for MM3, with 88.9%. OPLS4 followed, with 79.9%. Finally, as expected, incorporating ^3^*J*_HH_ values as described for the i*J*/d*J*-DP4 formalism yielded the best results, with AMBER and OPLS4 affording the best (95.0%) and worst (87.6%) performances, respectively. 

Although the averaged performances between the different FFs are rather similar, it is important to highlight that the results obtained for certain molecules strongly depend on the FF employed. Two different behaviors are noticed regarding the influence of the FF on the result, namely, robustness and sensitiveness. The former comprises those molecular systems which lead to consistent DP4 results, regardless of the FF used (for example, compounds **1**–**3**, **5**, and **19**–**21**). The second type includes structures showing significant differences in the fitness of the assignment, according to the FF (for example, compounds **6**, **8**, **11**, and **16**). In analyzing the structures within the test set, it is not easy to anticipate which ones will be more or less sensitive. This can be clarified by comparing the dissimilar performances of diastereoisomeric pairs. For example, compounds **7** and **15** are much less sensitive toward the FF than their corresponding diastereoisomers **6** and **16**, respectively. This suggests that the sensitivity cannot be linked to any structural property. Moreover, in the case of sensitive systems, it is also difficult to anticipate which FF will be optimal. For example, at level D, compound **6** is correctly elucidated, whereas compound **16** is misassigned, but the opposite trend is observed when using the geometries optimized at level E. Considering that both DP4 and *J*-DP4 feed on chemical shifts calculated from FF-minimized structures, the previous results indicate that subtle structural differences (consequences of the parametrization of each FF) have an impact on the calculated chemical shifts and, hence, on the resulting DP4-like probabilities.

**Figure 3:**
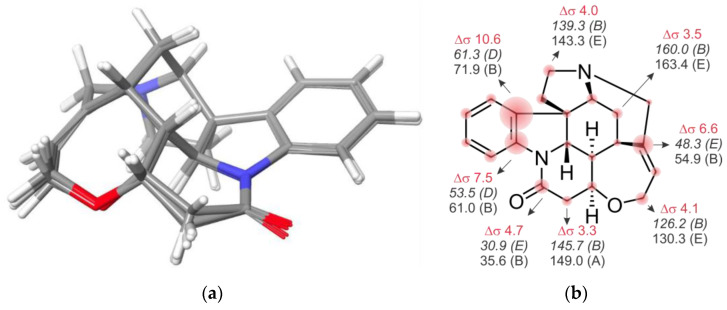
(**a**) Overlay of the global minima structures of strychnine (**1**) obtained for each FF. (**b**) ^13^CMaxΔσ values (red) computed for strychnine at the B3LYP/6-31G** level, using the five geometries shown in (**a**) as inputs. The minimum (italics) and maximum (plane) σ values are shown for some representative nuclei, with the corresponding FF in parenthesis.

Being aware of this uncertainty, we tried to dissect the effects arising from geometric structural differences from those derived from inaccuracies in energy calculations. For the first issue, we chose strychnine as a representative example of a rigid molecule where the results are independent of the energy factor (see below), in addition to the fact that it has a wide variety of chemical environments. Figure 3 displays the overlayed global minima of the correct isomer of the strychnine optimized at the five FF studied herein. All structures show a close similarity, though some small differences may be encountered, mainly in the torsion angles and the out-of-plane torsion of the amide group. However, important differences were found in the corresponding isotropic shielding values (σ) calculated at the B3LYP/6-31G** level. The difference between the maximum and minimum ^13^C σ (MaxΔσ) ranged from 1.5 ppm to 10.6 ppm (average 4 ppm), depending on the nucleus, whereas, for ^1^H, the MaxΔσ ranged from 0.25 to 0.77 ppm (average 0.54 ppm). Such large variations affect the scaled chemical shifts, with MaxΔδ values ranging from 0.8 to 8.4 ppm, with an average of 3.2 ppm, and from 0.07 to 0.42 ppm, with an average of 0.18 ppm, for the ^13^C and ^1^H data, respectively. In an effort to understand the origin of this observation, we analyzed all bond distances in strychnine isomers. 

For each bond, we found that the maximum differences between the geometries provided by the FFs under study ranged between 0.006 and 0.06 Å (average 0.02 Å). Similar observations were made for other systems under study, indicating that small variations in the interatomic distances and angles have a great impact on the calculated isotropic shielding values and therefore on the scaled chemical shifts. Naturally, this affects the errors (differences in the calculated and experimental data) used to calculate the DP4 and *J*-DP4 probabilities. For instance, the correct isomer of strychnine showed ^1^H CMAE and MaxErr values ranging from 0.08 and 0.19 (OPLS4) to 0.14 and 0.36 ppm (OPLS2005), along with ^13^C CMAE and MaxErr values from 1.22 and 3.88 (MM3) to 1.86 and 8.43 ppm (OPLS4). In the particular case of strychnine, this heterogeneity of NMR predictions does not affect the overall evidence for the assignment (both DP4 and *J*-DP4 values are >99.9% for all FFs). However, it is clear that, in other less straightforward systems, wider variations in the DP4 values should be expected.

**Figure 4:**
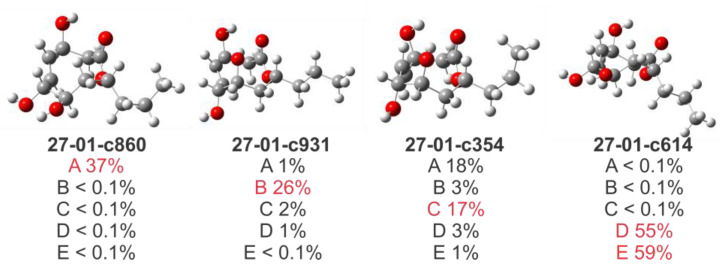
Global minima conformation of **27** found at the B3LYP/6-31G** level, using the structures optimized at the five FF shown. Boltzmann´s amplitudes of the global minima for each FF are shown in red, and the contributions of those shapes at the remaining levels are shown in black.

We next explored the influence of the FFs geometries on the relative B3LYP/6-31G** SCF energies used to calculate the Boltzmann amplitudes in flexible systems. We demonstrated that the conformational landscapes can be strongly influenced by the FF employed in the conformational search. This finding can be illustrated with two case studies (compounds **9** and **27**). For all possible isomers (eight in each case), a conformational sampling was performed at the MMFF level (21 kJ/MAD 05). To keep the number and type of conformations constant, the full set of conformers found (6301 for **9** and 6863 for **27**) were re-optimized at the MM3, AMBER, OPLS2005, and OPLS4 levels. The DFT energies of all molecule shapes were calculated at the B3LYP/6-31G** level, and the Boltzmann amplitudes (w_i_) were estimated at 25 °C. As shown in the SI, a remarkable discrepancy was observed for the contributing structures. The *R*^2^ values obtained by correlating the five sets of w_i_ values ranged between 0.12 and 0.57 and between <0.01 and 0.30 for compounds **9** and **27**, respectively. This becomes evident in comparing the Boltzmann’s amplitudes of the global minima found for **27** at each level of theory with the values computed for those shapes at the remaining four levels (Figure 4). For example, the most stable conformer located at the B3LYP/6-31G**//AMBER level contributes less than 0.1% according to the other levels of theory. 

According to the previous results, it is clear that subtle geometric differences in the MM-optimized geometries have a strong impact on both the NMR shielding constants and the Boltzmann amplitudes, with unpredictable effects on the calculated chemical shifts. The averaged CMAE calculated for the right isomers of the test set ranged from 0.12 ppm (MMFF) to 0.15 ppm (AMBER) for the ^1^H data and from 1.4 ppm (OPLS2005) to 1.7 ppm (MM3) for the ^13^C data, indicating that, on average, all FFs show a similar behavior in terms of accuracy (see SI). However, after analyzing each system individually, notable differences appear. For example, in the case of **17**, the ^1^H CMAE calculated for OPLS2005 was 0.53 ppm, which is 4.6 times larger than that computed for AMBER (0.12 ppm), but for compound **7**, OPLS2005 gave the best ^1^H prediction (CMAE 0.07 vs. 0.11–0.17 ppm computed for other levels). The same occurred in analyzing carbon chemical shifts. With AMBER, compound **14** shows a clearly smaller ^13^C MAE (up to 3.5 times) than the other FFs, but for the other 13 systems, the former FF was the least accurate. The difficulty in anticipating the optimal FF in terms of NMR accuracy for a given system is complemented by the relative nature of DP4, meaning that a proper assignment will be made as long as the errors computed for the right isomer are smaller than those of the other candidates. Despite the FF affording a good NMR prediction for the right isomer, it cannot be ruled out that the errors are even smaller for other candidates, hence leading to a wrong assignment. 

**Figure 5:**
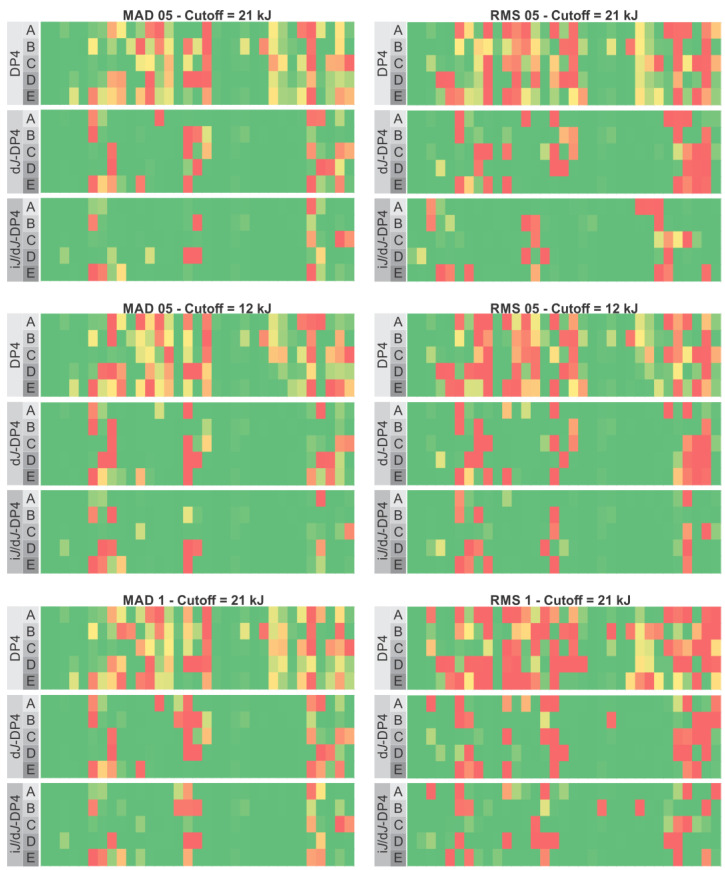
Performances of DP4 (**top**), d*J*-DP4 (**middle**), and i*J*/d*J*-DP4 (**bottom**) with the test set in the five FF studied (A: MM3, B: AMBER, C: MMFF, D: OPLS2005, E: OPLS4), after evaluating different strategies to remove duplicate conformations and energy thresholds.

The previous results also illustrate how another two factors clearly influence the performance of the calculations: (i) the selected FF energy cutoff and (ii) the geometric method used to eliminate the redundant conformers obtained in the search. The first parameter may have a deep impact on the results, since the final outcomes can be correct only if the appropriate conformations are included in the pool used for the DFT calculations [35]. This suggests that using high values could be the harmless option to avoid incorrect energy evaluation by the selected FF and the subsequent erroneous trimming of feasible conformations. On the other hand, the lower the energy cutoff, the smaller the number of structures used in the calculations and the required computational time (the average number was reduced by 50% by passing from 21 kJ/mol to a 12 kJ/mol cutoff). Another important issue that is often overlooked is the method used to eliminate redundant conformer geometries that otherwise would undergo DFT calculations. Therefore, we compared the use of RMSD versus MAD algorithms using two different cutoffs (0.5 Å and 1.0 Å), which, coupled with the two energy cutoffs (12 kJ/mol and 21 kJ/mol), yielded six combinations (Figure 5). In each case, the best results were obtained with i*J*/d*J*-DP4, suggesting that the superior performance of the last is independent of the conformational search setup. No important differences in the effect of the energy cutoff values were apparent under the tested conditions. Using MAD-0.5 Å and a 21 kJ/mol cutoff, the average performances (among the five FFs evaluated) were 73%, 87%, and 92% for DP4, d*J*-DP4, and i*J*/d*J*-DP4, respectively. These are similar to the values of 72%, 88%, and 91%, respectively, obtained with a 12 kJ/mol cutoff. A similar trend was observed using RMSD-0.5, with 66%, 84%, and 87% scores for 21 kJ/mol and 65%, 85%, and 89% scores for 12 kJ/mol. However, it is worth noting that, although both energy cutoffs provided statistically equivalent performances, there were a few intriguing examples where the performance significantly changed (see SI), making it difficult to draw a general conclusion. It was also evident that each FF generates a different number of conformations for the study. As a general trend, AMBER returned the largest number of structures, and OPLS2005 returned the least. The MAD method provided consistently better results when selected for eliminating redundant conformers than those using RMSD. The best overall results were obtained with MAD 0.5 Å (91% average for five FFs), whereas RMSD 1 Å gave the poorest (82% average for 5 FFs). We consider this a consequence of the loss of structural diversity exerted by RSMD. By imposing more stringent criteria to declare non-equivalency, it causes the loss of similar (yet not identical) structures. This could mask subtle conformational differences that negatively impact the quality of the results. Thus, based on the results, and considering both the classification performance and the computational cost, for practical applications, we recommend using MAD-05 Å and 12 kJ/mol cutoffs under i*J*/d*J*-DP4 formalism.

**Figure 6:**
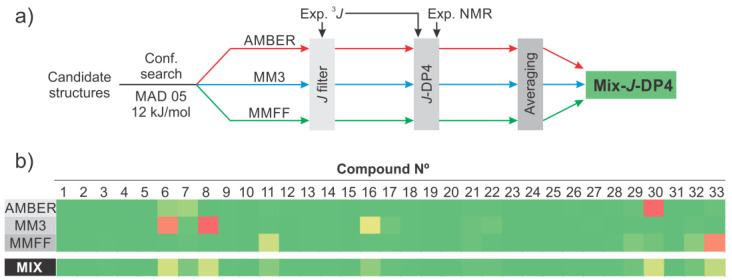
(**a**) Workflow of mix-*J*-DP4, (**b**) i*J*/d*J*-DP4 results obtained with AMBER, MM3, and MMFF using 12 kJ/MAD 05, and averaged values (mix).

Within these boundaries, AMBER, MMFF, and MM3 performed better (95%, 93%, and 92%, respectively) than the modern OPLS2005 (89%) and OPLS4 (88%). Although no single FF afforded optimal classification performance, it was interesting to note that the misassigned cases were very sensitive to the FF employed. This led us to propose a new method based on the combined results from several FFs. Figure 6a shows the workflow of the so-called mix-*J*-DP4 approach. It involves the parallel computing of i*J*/d*J*-DP4 probabilities with AMBER, MMFF, and MM3 geometries, using the optimal selection criteria discussed above. The three independent results are then averaged into a single probability. This is in line with recent recommendations of not relying on a single metric but on the results of different calculations in order to reinforce the confidence in the assignment [36]. Although this methodology increases the overall computational cost (relative to a single i*J*/d*J*-DP4 calculation), it is still faster than a standard DP4 procedure. In addition, it provides both an excellent classification performance (100% in our test set, Figure 6b) and a strong backup to the results. 

**Figure 7:**
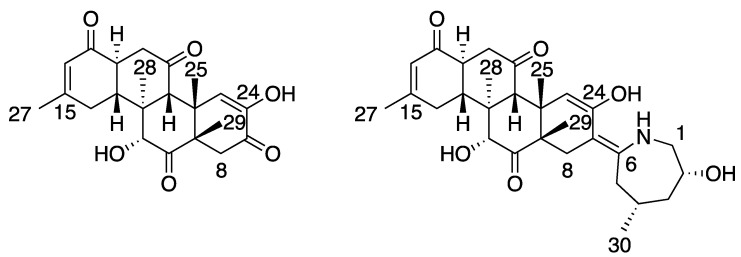
Structures of zoarenone (**34**) and zoaramine (**35**).

The impact of the previous approach can be illustrated in the stereochemical assignment of zoarenone (**34**) and zoaramine (**35**) [37]*,* two complex metabolites (Figure 7) belonging to the family of norzoanthamine, [38] a group of bioactive alkaloids isolated from the marine cnidiaria *Zoanthus pulchelus* [39,40].

**Figure 8:**
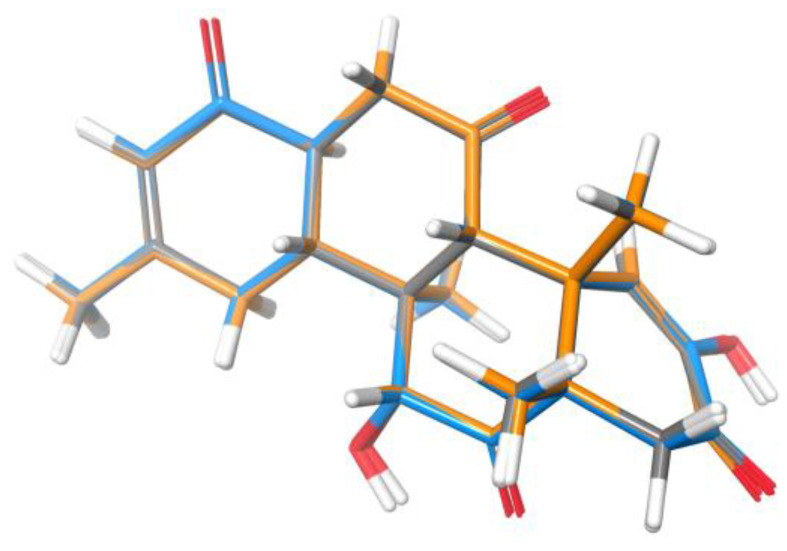
Overlay of the global minima structures of the correct structure of zoarenone (**34**) obtained for each FF (AMBER: orange, MM3: grey, and MMFF: blue).

Zoarenone (**34**) is a rigid tetracyclic compound comprising seven asymmetric carbons. Following the mix-*J*-DP4 procedure, the corresponding 64 diastereoisomers were built in silico, and the conformational searches and energy minimizations were performed with the AMBER, MM3, and MMFF force fields using MAD-05 Å and 12 kJ/mol cutoffs. After ^3^*J*_HH_ filtering using the H13-H18 coupling, the number of suitable structures was reduced to almost half. The three independent *J*-DP4 calculations strongly supported the right isomer in high probability (>99%), despite the C9 epimer (9R*) showing the best probability using only H data (>90%). This reinforces the general recommendation that, whenever possible, all types of data should be employed to obtain meaningful results. In addition, the fact that the three FFs gave similar *J*-DP4 results grants a much stronger confidence in the assignment than what would have been achieved through the traditional procedure of using only one FF. As shown in Figure 8, a great similarity is observed in the global minima structures found for each force field, which would explain the homogeneity of the results found in this case study. 

**Figure 9:**
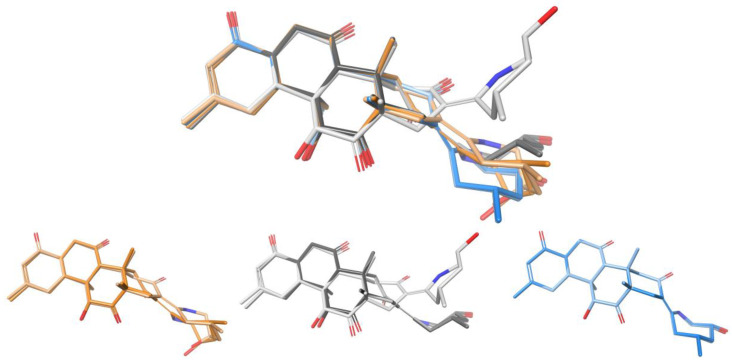
Overlay of the conformers with >10% Boltzmann contribution for the correct structure of zoaramine (**35**) obtained for each FF (AMBER: orange, MM3: grey, and MMFF: blue).

However, in the case of zoaramine (**35**), divergent *J*-DP4 results were obtained when using different force fields. Considering the structural similarity between **35** and **34** (the former incorporates a flexible seven-membered ring into the backbone of **34**), this emphasizes the unpredictability of the optimal force field for a given molecule. On this occasion, AMBER failed, giving a <0.1% probability to the right isomer, while MM3 and MMFF succeeded, obtaining a >99.9% probability for it. Therefore, combining the three results, a mean value of 66.6% probability for the right isomer would be obtained, giving more confidence to the assignment. This time, MM3 gave the right assignment for all calculated parameters (H-DP4: 99%, C-DP4: 78%, and *J*-DP4: 86%), while MMFF failed in its carbon prediction (H-DP4: >99.9%, C-DP4: 2%, and *J*-DP4: 99%). The underperformance of MMFF in C-DP4 is associated with the poorest prediction of ^13^C chemical shifts (CMAE of 3.99 ppm vs. 2.88 ppm for MMFF and MM3 geometries, respectively). On the other hand, using AMBER geometries, the C2 epimer of **35** was identified as the most likely structure, both according to H-DP4 and C-DP4, which is a consequence of the best overall fitting between the experimental and calculated data (C-CMAE 2.44 ppm; H-CMAE 0.17 ppm). Interestingly, in accordance with our previous observations for compounds **9** and **27** (Figure 4), the energy landscapes computed for **35** show substantial differences for each FF (Figure 9). Thus, while B3LYP/6-31G**//MMFF selected conformers c3 and c4 with 69% and 31% Boltzmann amplitudes, the B3LYP/6-31G**//MM3 level used four conformers (c10: 42%, c14: 21%, c11: 17%, and c17: 10%) to describe the conformational behavior of the right isomer. On the contrary, B3LYP/6-31G**//AMBER chose different conformations (c13: 85% and c5: 11%) for the right isomer and c144: 64% and c145: 36% for the incorrectly selected 2S* epimer. This could be the main reason behind AMBER´s poor performance in this particular case. 

## 3. Materials and Methods

Conformational searches were carried out using the Conformational Search protocol in the gas phase using the MMFF, and the found conformers were re-optimized with the AMBER, MM3, OPLS2005, and OPLS4 force fields, with 21 kJ/mol as the energy cutoff and a MAD of 0.5 Å. For each FF, an energy cutoff was carried out with 21 and 12 kJ/mol, using MAD (0.5 and 1 Å) or RMSD (0.5 and 1 Å). These conformers were subjected to gauge including atomic orbitals (GIAO) NMR calculations at DFT levels (B3LYP/6-31G** levels of theory as recommended for DP4 and *J*-DP4) using Gaussian 09. The *J* calculations were carried out at the same level of theory considering only the Fermi contact term and were scaled as recommended in *J*-DP4. For i*J*-DP4 calculations, the constrained search was performed by keeping only the suitable conformations (that is, those conformations showing dihedral angles in agreement with the experimental ^3^*J*_HH_ values). 

Unscaled chemical shifts (δ_u_) were calculated using TMS as a reference standard according to the following expression: δ_u_ = σ_0_ − σ_x_, where σ_x_ is the Boltzmann-averaged shielding tensor (over all significantly populated conformations) and σ_0_ is the shielding tensor of the TMS computed at the same level of theory used to calculate σ_x_. Boltzmann averaging was performed according to Equation (1):

(1) $$\sigma_{x}=\frac{\sum_{i}\sigma_{i}^{x}e^{\left(-\frac{E_{i}}{\mathrm{RT}}\right)}}{\sum_{i}e^{\left(-\frac{E_{i}}{\mathrm{RT}}\right)}}$$

where σ*_i_*^x^ is the shielding constant for the nucleus x in the conformer *i*, R is the molar gas constant (8.3145 J/K mol), T is the temperature used for the calculations (298 K), and E*_i_* is the relative energy of the conformer *i* (to the lowest energy conformer) obtained from a single-point NMR calculation at the corresponding level of theory. The scaled chemical shifts (δs) were computed as δ_s_ = (δ_u_ − b)/m, where m and b are the slope and intercept, respectively, resulting from a linear regression calculation on a plot of δ_u_ against δ_exp_. The *J*-DP4 calculations were carried out using a home-made Matlab script using the statistical parameters reported in the original paper.

## 4. Conclusions

In summary, by combining the i*J*/d*J*-DP4 results obtained with three different force fields (AMBER, MMFF, and MM3), we have developed a new methodology that allows for the assignment of relative configurations in complex molecules with a high certainty. The modest increment in the overall computational cost is fully justified by the remarkable increase in the confidence in the assignments. The mix-*J*-DP4 can be easily computed using the Excel spreadsheet previously reported for *J*-DP4.

## Data Availability

Not applicable.

## Supplementary Materials

The following supporting information can be downloaded at: https://www.mdpi.com/article/10.3390/md20110699/s1, Computational details, structures and cartesian coordinates, calculated NMR data, and results for DP4 and *J*-DP4 methods (PDF).
