# Supplementary material for: May the Force (Field) Be with You: On the Importance of Conformational Searches in the Prediction of NMR Chemical Shifts
Source: Mar Drugs. 2022 Nov 8;20(11):699. doi: 10.3390/md20110699 (PMC9694776; doi:10.3390/md20110699)
Supplement: Supplementary file 1 [file marinedrugs-20-00699-s001.zip › marinedrugs-2008265-supplementary.pdf]

Table of contents


*1. Correct structures of compounds 1-33. ..... 3*

*2. Experimental chemical shifts and isotropic magnetic shielding constants of studied isomers at MMFF 21 kJ/mol MAD 0.5 Å. ..... 4*

*3. Comparison of isotropic magnetic shielding constants ($\sigma$) for the correct isomer in all FF for strychnine ..... 46*

*4. Summary of errors ..... 48*

*5. Scatter plots of the Boltzmann´s amplitudes ($w_i$) ..... 52*

*6. DP4, dJ-DP4 and iJ/dJ-DP4 results for compounds 1-33 ..... 60*

*7. Summary of general results ..... 80*

*8. Zoarenone and Zoaramine as examples ..... 83*

## 1. Correct structures of compounds 1-33.

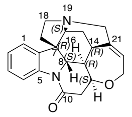
1

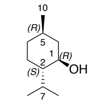
2

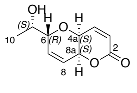
3

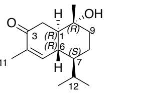
4

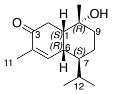
5

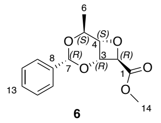
6

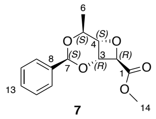
7

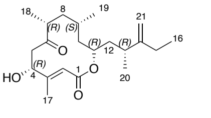
8

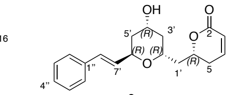
9

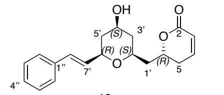
10

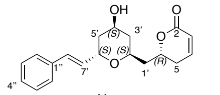
11

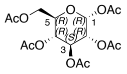
12

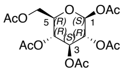
13

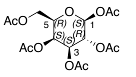
14

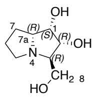
15

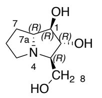
16

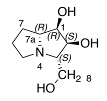
17

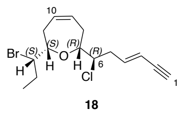
18

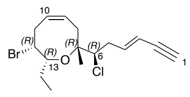
19

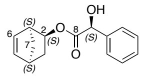
20

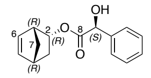
21

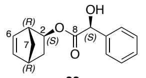
22

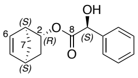
23

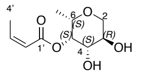
24

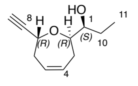
25

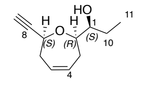
26

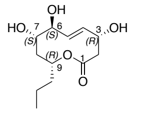
27

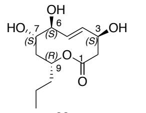
28

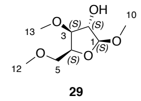
29

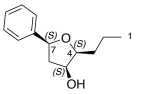
30

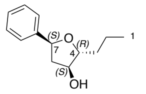
31

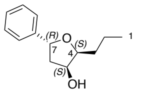
32

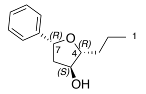
33

2. **Experimental chemical shifts and isotropic magnetic shielding constants of studied isomers at MMFF 21 kJ/mol MAD 0.5 Å.**

**Table S1.** Boltzmann averaged GIAO isotropic magnetic shielding constants (σ) of isomers 1-13 calculated at the B3LYP/6-31G** level of theory for compound **1**[1]

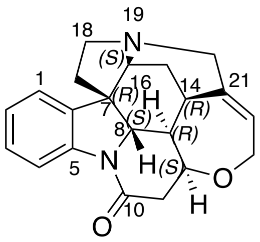


**1**

| Isotropic shielding constants | | | | | | | | |
|---|---|---|---|---|---|---|---|---|
| **Nuclei** | **Experimental** | **Isomer 1** | **Isomer 2** | **Isomer 3** | **Isomer 4** | **Isomer 5** | **Isomer 6** | **Isomer 7** |
| C 1 | 122.26 | 75.41 | 74.46 | 74.46 | 74.61 | 73.65 | 72.94 | 73.64 |
| C 2 | 124.20 | 72.70 | 72.09 | 72.10 | 72.63 | 72.71 | 72.38 | 72.71 |
| C 3 | 128.56 | 67.27 | 66.67 | 66.68 | 67.08 | 66.38 | 66.3 | 66.39 |
| C 4 | 116.23 | 80.77 | 80.07 | 80.08 | 81.79 | 80.88 | 80.95 | 80.89 |
| C 5 | 142.23 | 56.07 | 54.16 | 54.15 | 54.98 | 54.66 | 53.71 | 54.66 |
| C 6 | 132.72 | 63.80 | 63.35 | 63.35 | 64.32 | 64.04 | 64.83 | 64.04 |
| C 7 | 51.96 | 138.10 | 137.51 | 137.50 | 139.63 | 138.98 | 133.11 | 138.98 |
| C 8 | 60.10 | 130.19 | 126.20 | 126.20 | 126.48 | 128.09 | 114.09 | 128.10 |
| C 10 | 169.28 | 31.97 | 33.43 | 33.44 | 30.50 | 33.96 | 31.34 | 33.96 |
| C 11 | 42.48 | 148.80 | 154.32 | 154.32 | 148.37 | 149.84 | 147.00 | 149.84 |
| C 12 | 76.85 | 111.36 | 115.47 | 115.47 | 111.68 | 103.13 | 114.45 | 103.12 |
| C 13 | 48.22 | 142.06 | 141.92 | 141.91 | 136.45 | 138.33 | 128.22 | 138.33 |
| C 14 | 31.60 | 157.84 | 164.66 | 164.66 | 161.17 | 152.06 | 157.83 | 152.06 |
| C 15 | 26.84 | 162.26 | 170.39 | 170.39 | 165.67 | 170.62 | 161.27 | 170.62 |
| C 16 | 60.28 | 131.18 | 126.62 | 126.64 | 132.41 | 128.73 | 111.86 | 128.74 |
| C 17 | 42.85 | 146.49 | 153.18 | 153.18 | 146.04 | 148.79 | 145.24 | 148.78 |
| C 18 | 50.35 | 141.84 | 140.25 | 140.25 | 141.99 | 139.97 | 138.77 | 139.97 |
| C 20 | 52.68 | 138.82 | 140.76 | 140.76 | 140.15 | 141.48 | 133.28 | 141.48 |
| C 21 | 140.45 | 53.43 | 40.46 | 40.44 | 55.10 | 28.64 | 54.07 | 28.65 |
| C 22 | 127.34 | 65.62 | 71.64 | 71.64 | 71.70 | 73.50 | 88.43 | 73.51 |
| C 23 | 64.60 | 128.72 | 120.77 | 120.79 | 120.10 | 124.25 | 117.04 | 124.25 |
| H 1 | 7.17 | 24.59 | 24.51 | 24.51 | 24.60 | 24.52 | 24.48 | 24.52 |
| H 2 | 7.10 | 24.76 | 24.70 | 24.70 | 24.78 | 24.75 | 24.76 | 24.75 |
| H 3 | 7.26 | 24.49 | 24.44 | 24.44 | 24.50 | 24.44 | 24.44 | 24.44 |
| H 4 | 8.09 | 23.52 | 23.47 | 23.48 | 23.72 | 23.63 | 23.69 | 23.63 |
| H 8 | 3.86 | 27.80 | 28.04 | 28.03 | 28.32 | 28.01 | 28.21 | 28.01 |
| H 11a | 3.13 | 29.11 | 29.12 | 29.12 | 28.94 | 29.34 | 28.83 | 29.34 |
| H 11b | 2.67 | 28.76 | 29.19 | 29.19 | 29.29 | 29.14 | 29.11 | 29.14 |
| H 12 | 4.29 | 27.70 | 27.68 | 27.68 | 28.04 | 27.47 | 28.21 | 27.47 |
| H 13 | 1.28 | 30.66 | 28.69 | 28.69 | 30.07 | 28.53 | 29.05 | 28.53 |
| H 14 | 3.15 | 28.86 | 28.06 | 28.06 | 28.62 | 28.94 | 27.69 | 28.94 |
| H 15a | 2.36 | 29.58 | 30.05 | 30.05 | 30.39 | 30.30 | 30.02 | 30.14 |

[1] Compound **1**: NMR data from Berger, S. Braun, S. 2014, 200 and More NMR Experiments, Ed. Wiley-VCH pp 802-803

| | | | | | | | | |
|---|---|---|---|---|---|---|---|---|
| H 15b | 1.46 | 30.38 | 30.22 | 30.22 | 29.73 | 30.14 | 30.53 | 30.30 |
| H 16 | 3.96 | 27.87 | 28.28 | 28.28 | 27.96 | 28.21 | 27.47 | 28.21 |
| H 17a | 1.89 | 29.83 | 29.19 | 29.20 | 29.87 | 29.33 | 29.65 | 29.33 |
| H 17b | 1.89 | 29.98 | 30.13 | 30.13 | 30.15 | 30.20 | 30.13 | 30.2 |
| H 18a | 3.22 | 28.95 | 28.61 | 28.61 | 29.11 | 28.91 | 28.43 | 28.57 |
| H 18b | 2.88 | 28.75 | 28.84 | 28.84 | 28.73 | 28.57 | 28.95 | 28.91 |
| H 20a | 3.72 | 29.21 | 29.02 | 29.02 | 28.06 | 27.95 | 28.55 | 28.71 |
| H 20b | 2.75 | 28.16 | 27.97 | 27.98 | 29.18 | 28.71 | 28.03 | 27.95 |
| H 22 | 5.92 | 25.75 | 26.97 | 26.97 | 26.69 | 26.36 | 27.74 | 26.36 |
| H 23a | 4.15 | 27.86 | 27.49 | 27.50 | 27.18 | 27.27 | 27.46 | 27.51 |
| H 23b | 4.07 | 28.06 | 27.04 | 27.05 | 27.50 | 27.51 | 26.97 | 27.27 |
| J 1,2 | 7.49 | 7.46 | 7.45 | 7.45 | 7.40 | 7.44 | 7.41 | 7.44 |
| J 1,3 | 1.08 | 0.97 | 0.95 | 0.95 | 0.95 | 0.93 | 0.94 | 0.93 |
| J 1,4 | 0.23 | 0.68 | 0.70 | 0.70 | 0.70 | 0.71 | 0.70 | 0.71 |
| J 2,3 | 7.44 | 7.00 | 7.02 | 7.03 | 7.01 | 7.03 | 7.05 | 7.03 |
| J 2,4 | 0.98 | 0.83 | 0.83 | 0.83 | 0.81 | 0.83 | 0.82 | 0.83 |
| J 3,4 | 7.90 | 8.09 | 8.04 | 8.04 | 8.06 | 8.00 | 8.03 | 8.00 |
| J 8,13 | 10.40 | 9.62 | 4.03 | 4.03 | 9.13 | 7.27 | 7.02 | 7.27 |
| J 11a,12 | 3.34 | 2.60 | 7.21 | 7.20 | 6.08 | 11.82 | 6.72 | 11.82 |
| J 11b,12 | 8.47 | 8.15 | 8.71 | 8.71 | 9.12 | 4.64 | 6.94 | 4.64 |
| J 12,13 | 3.30 | 3.06 | 6.48 | 6.48 | 8.77 | 5.63 | 10.6 | 5.63 |
| J 13,14 | 3.29 | 2.81 | 5.45 | 5.45 | 4.73 | 3.92 | 0.42 | 3.93 |
| J 14,15a | 4.11 | 4.69 | 2.09 | 2.09 | 2.11 | 4.30 | 6.59 | 2.03 |
| J 14,15b | 1.96 | 1.75 | 4.21 | 4.21 | 4.12 | 2.03 | 3.47 | 4.30 |
| J 14,22 | 0.47 | -3.35 | -1.91 | -1.91 | -2.77 | -1.54 | -4.43 | -1.54 |
| J 14,20a | 1.61 | -0.54 | -0.48 | -0.48 | -2.09 | -1.19 | -0.58 | -0.60 |
| J 15a,16 | 4.33 | 3.73 | 4.68 | 4.69 | 1.82 | 1.25 | 3.32 | 5.05 |
| J 15b,16 | 2.42 | 2.00 | 1.29 | 1.29 | 4.04 | 5.05 | 1.45 | 1.25 |
| J 20a,22 | 1.79 | -0.52 | -0.68 | -0.68 | -2.74 | -3.06 | -0.97 | -0.80 |
| J 22,23a | 7.00 | 7.22 | 3.14 | 3.14 | 2.38 | 5.62 | 3.85 | 6.43 |
| J 22,23b | 6.10 | 6.14 | 2.74 | 2.74 | 4.54 | 6.43 | 2.27 | 5.62 |

| Nuclei | Experimental | Isomer 8 | Isomer 9 | Isomer 10 | Isomer 11 | Isomer 12 | Isomer 13 |
|---|---|---|---|---|---|---|---|
| C 1 | 122.26 | 73.49 | 79.58 | 78.67 | 76.38 | 75.11 | 74.46 |
| C 2 | 124.20 | 72.48 | 71.55 | 71.32 | 71.15 | 71.16 | 75.54 |
| C 3 | 128.56 | 66.44 | 68.54 | 68.15 | 67.81 | 67.72 | 69.71 |
| C 4 | 116.23 | 80.78 | 79.75 | 80.21 | 80.44 | 81.17 | 86.26 |
| C 5 | 142.23 | 54.9 | 56.65 | 56.48 | 54.66 | 53.95 | 56.35 |
| C 6 | 132.72 | 64.64 | 51.29 | 51.27 | 56.7 | 56.83 | 51.43 |
| C 7 | 51.96 | 132.74 | 136.94 | 137.85 | 136.02 | 134.95 | 131.55 |
| C 8 | 60.10 | 117.71 | 126.97 | 127.39 | 127.56 | 127.73 | 127.61 |
| C 10 | 169.28 | 33.34 | 35.36 | 34.62 | 33.02 | 31.57 | 14.48 |
| C 11 | 42.48 | 149.34 | 148.6 | 147.87 | 149.74 | 150.93 | 148.6 |
| C 12 | 76.85 | 115.72 | 111.11 | 119.02 | 108.94 | 115.91 | 112.47 |
| C 13 | 48.22 | 130.72 | 153.97 | 144.75 | 145.41 | 145.18 | 142.56 |
| C 14 | 31.60 | 157.97 | 155.21 | 159.62 | 152.38 | 158.7 | 152.28 |
| C 15 | 26.84 | 159.28 | 162.52 | 165.25 | 166.08 | 165.07 | 157.19 |
| C 16 | 60.28 | 114.27 | 132.44 | 133.22 | 132.95 | 130.87 | 117.9 |
| C 17 | 42.85 | 146.22 | 150.43 | 150.93 | 154.38 | 153.19 | 143.86 |

| | | | | | | | |
|---|---|---|---|---|---|---|---|
| C 18 | 50.35 | 139.55 | 137.86 | 138.08 | 141.05 | 140.91 | 132.08 |
| C 20 | 52.68 | 135.79 | 132.60 | 134.36 | 138.98 | 138.54 | 133.07 |
| C 21 | 140.45 | 50.80 | 47.42 | 51.01 | 36.64 | 43.60 | 42.64 |
| C 22 | 127.34 | 84.20 | 67.35 | 72.29 | 72.40 | 74.75 | 61.11 |
| C 23 | 64.60 | 122.30 | 125.02 | 122.17 | 127.07 | 117.66 | 127.17 |
| H 1 | 7.17 | 24.46 | 24.56 | 24.59 | 24.53 | 24.49 | 24.34 |
| H 2 | 7.10 | 24.73 | 24.73 | 24.74 | 24.71 | 24.70 | 24.99 |
| H 3 | 7.26 | 24.43 | 24.51 | 24.51 | 24.51 | 24.51 | 24.75 |
| H 4 | 8.09 | 23.67 | 23.56 | 23.66 | 23.61 | 23.71 | 24.94 |
| H 8 | 3.86 | 27.86 | 27.26 | 27.61 | 28.18 | 27.92 | 27.75 |
| H 11a | 3.13 | 29.13 | 29.28 | 29.41 | 29.15 | 29.31 | 29.67 |
| H 11b | 2.67 | 28.90 | 28.98 | 29.12 | 29.23 | 29.13 | 29.04 |
| H 12 | 4.29 | 27.89 | 27.98 | 27.62 | 27.74 | 27.67 | 28.16 |
| H 13 | 1.28 | 29.29 | 29.54 | 29.18 | 29.53 | 29.05 | 30.03 |
| H 14 | 3.15 | 28.13 | 28.82 | 28.48 | 28.85 | 28.13 | 28.93 |
| H 15a | 2.36 | 29.83 | 30.05 | 30.07 | 30.03 | 30.08 | 30.32 |
| H 15b | 1.46 | 30.51 | 29.47 | 29.69 | 29.92 | 29.81 | 29.29 |
| H 16 | 3.96 | 27.44 | 27.82 | 27.88 | 27.62 | 27.59 | 27.86 |
| H 17a | 1.89 | 29.67 | 28.40 | 28.93 | 29.43 | 29.45 | 30.11 |
| H 17b | 1.89 | 30.00 | 30.72 | 30.64 | 30.01 | 29.98 | 29.68 |
| H 18a | 3.22 | 28.49 | 29.18 | 29.23 | 29.05 | 29.13 | 28.11 |
| H 18b | 2.88 | 28.83 | 28.82 | 28.89 | 28.69 | 28.68 | 27.96 |
| H 20a | 3.72 | 28.59 | 27.93 | 27.82 | 27.99 | 27.93 | 27.57 |
| H 20b | 2.75 | 28.02 | 29.07 | 29.1 | 28.83 | 29.05 | 28.37 |
| H 22 | 5.92 | 26.84 | 25.76 | 26.68 | 26.16 | 27.08 | 26.35 |
| H 23a | 4.15 | 27.52 | 28.12 | 27.31 | 27.42 | 26.91 | 28.34 |
| H 23b | 4.07 | 28.09 | 27.67 | 27.51 | 27.67 | 27.59 | 28.20 |
| J 1,2 | 7.49 | 7.43 | 7.24 | 7.22 | 7.25 | 7.25 | 7.70 |
| J 1,3 | 1.08 | 0.93 | 0.91 | 0.89 | 0.89 | 0.90 | 0.78 |
| J 1,4 | 0.23 | 0.71 | 0.75 | 0.76 | 0.77 | 0.76 | 0.55 |
| J 2,3 | 7.44 | 7.05 | 7.07 | 7.09 | 7.07 | 7.07 | 7.02 |
| J 2,4 | 0.98 | 0.83 | 0.78 | 0.78 | 0.78 | 0.78 | 1.07 |
| J 3,4 | 7.90 | 8.01 | 7.91 | 7.88 | 7.91 | 7.92 | 8.07 |
| J 8,13 | 10.40 | 8.28 | 11.72 | 9.54 | 13.17 | 12.94 | 8.5 |
| J 11a,12 | 3.34 | 3.93 | 4.42 | 10.33 | 6.24 | 11.62 | 3.83 |
| J 11b,12 | 8.47 | 7.76 | 2.01 | 3.42 | 8.81 | 4.79 | 2.37 |
| J 12,13 | 3.30 | 4.54 | 3.52 | 11.07 | 7.91 | 9.93 | 3.29 |
| J 13,14 | 3.29 | -1.62 | 6.02 | 8.88 | 6.05 | 8.43 | 2.88 |
| J 14,15a | 4.11 | 6.87 | 1.44 | 1.90 | 4.07 | 4.17 | 1.57 |
| J 14,15b | 1.96 | 2.89 | 4.57 | 3.84 | 1.44 | 1.35 | 4.82 |
| J 14,22 | 0.47 | -4.08 | -3.92 | -3.38 | -1.90 | -1.80 | -2.90 |
| J 14,20a | 1.61 | -0.38 | -2.22 | -2.37 | -1.27 | -1.34 | -2.04 |
| J 15a,16 | 4.33 | 3.11 | 2.92 | 2.89 | 1.49 | 1.57 | 6.78 |
| J 15b,16 | 2.42 | 1.64 | 2.18 | 2.30 | 3.77 | 3.44 | 0.79 |
| J 20a,22 | 1.79 | -0.81 | -1.78 | -2.44 | -2.74 | -2.50 | -2.99 |
| J 22,23a | 7.00 | 6.52 | 5.53 | 2.49 | 6.23 | 2.39 | 5.99 |
| J 22,23b | 6.10 | 5.41 | 7.82 | 4.69 | 6.55 | 3.78 | 7.38 |

**Table S2.** Boltzmann averaged GIAO isotropic magnetic shielding constants (σ) of isomers 1-4 calculated at the B3LYP/6-31G** level of theory for compound **2**[2]

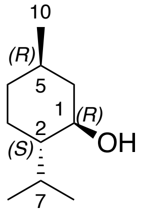


**2**

| Isotropic shielding constants | | | | | |
|---|---|---|---|---|---|
| **Nuclei** | **Experimental** | **Isomer 1** | **Isomer 2** | **Isomer 3** | **Isomer 4** |
| C 1 | 71.6 | 122.89 | 123.38 | 121.66 | 125.06 |
| C 2 | 50.2 | 142.56 | 142.99 | 141.98 | 140.48 |
| C 3 | 23.2 | 169.11 | 165.42 | 166.09 | 169.38 |
| C 4 | 34.6 | 159.39 | 156.13 | 156.56 | 160.16 |
| C 5 | 31.7 | 161.94 | 163.19 | 159.07 | 161.75 |
| C 6 | 45.1 | 149.53 | 145.34 | 144.57 | 150.54 |
| C 7 | 25.9 | 158.36 | 157.92 | 161.16 | 161.6 |
| C 8 | 16.1 | 169.63 | 169.6 | 173.61 | 171.93 |
| C 9 | 16.1 | 169.73 | 169.85 | 169.95 | 169.85 |
| C 10 | 22.2 | 169.1 | 167.63 | 167.82 | 170.15 |
| H 1 | 3.43 | 27.55 | 27.58 | 28 | 27.61 |
| H 3a | 0.99 | 30.36 | 30.24 | 30.55 | 30.22 |
| H 3b | 1.64 | 29.78 | 29.92 | 30.13 | 30.09 |
| H 2 | 1.13 | 30.71 | 30.73 | 30.37 | 30.56 |
| H 4a | 0.87 | 30.16 | 30.06 | 30.83 | 30.3 |
| H 4b | 1.68 | 30.25 | 30.84 | 30.17 | 30.42 |
| H 5 | 1.44 | 29.99 | 29.67 | 30.35 | 29.77 |
| H 6ax | 1.98 | 30.06 | 30.62 | 30.34 | 30.49 |
| H 6b | 0.99 | 30.51 | 30.47 | 30.61 | 30.09 |
| H 7 | 2.19 | 29.75 | 29.81 | 29.37 | 29.55 |
| H 8 | 0.94 | 30.74 | 30.76 | 30.84 | 30.81 |
| H 9 | 0.83 | 30.79 | 30.79 | 30.84 | 30.85 |
| H 10 | 0.94 | 30.51 | 30.96 | 30.91 | 30.83 |
| J 1,2 | 9.9 | 2.57 | 2.13 | 9.29 | 7.02 |
| J 1ax,6ax | 12 | 3.33 | 3.18 | 9.63 | 7.66 |
| J 1ax,6eq | 4 | 2.64 | 1.98 | 4.18 | 3.66 |
| J 5ax,6ax | 10.9 | 2.29 | 3.41 | 11.35 | 4.44 |
| J 5ax,6eq | 4.5 | 5.41 | 11.17 | 3.33 | 5.18 |

[2] Compound **2**: NMR data from S. Berger, D. Sicker. **2009**, Classics in Spectroscopy: Isolation and Structure Elucidation of Natural Products. 1st Edition Ed. Wiley-VCH pp 373-387.

**Table S3.** Boltzmann averaged GIAO isotropic magnetic shielding constants (σ) of isomers 1-13 calculated at the B3LYP/6-31G** level of theory for compound **3**[3]

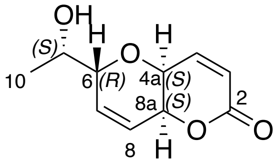


**3**

| | | Isotropic shielding constants | | | | | | | |
|---|---|---|---|---|---|---|---|---|---|
| **Nuclei** | **Experimental** | **Isomer 1** | **Isomer 2** | **Isomer 3** | **Isomer 4** | **Isomer 5** | **Isomer 6** | **Isomer 7** | **Isomer 8** |
| C 2 | 161.73 | 35.09 | 36.72 | 33.54 | 33.47 | 33.41 | 33.55 | 36.58 | 35.13 |
| C 3 | 123.28 | 67.95 | 69.93 | 71.34 | 71.8 | 72.64 | 71.37 | 69.77 | 67.85 |
| C 4 | 144.24 | 57.34 | 52.05 | 49.07 | 48.54 | 47.76 | 49.08 | 52.4 | 57.52 |
| C 4a | 64.9 | 124.75 | 124.31 | 119.02 | 123.99 | 122.65 | 119.11 | 124.53 | 124.8 |
| C 6 | 74.79 | 111 | 115.11 | 108.53 | 109.43 | 111.98 | 107.04 | 116.34 | 109.74 |
| C 7 | 129.57 | 60.51 | 64.87 | 65.14 | 66.44 | 68.65 | 65.17 | 64.61 | 60.36 |
| C 8 | 124.68 | 68.52 | 66.25 | 64.04 | 62.85 | 60.52 | 64.06 | 66.6 | 68.51 |
| C 8a | 70 | 121.52 | 121.65 | 116.43 | 116.66 | 116.71 | 116.63 | 121.59 | 121.52 |
| C 9 | 69.66 | 120.8 | 120.38 | 120.84 | 122.76 | 118.47 | 120.72 | 120.36 | 120.89 |
| C 10 | 18.72 | 173.28 | 173.17 | 173.29 | 173.4 | 169.85 | 172.98 | 173.17 | 173.04 |
| H 3 | 6.07 | 25.61 | 25.86 | 25.82 | 25.85 | 25.9 | 25.82 | 25.84 | 25.6 |
| H 4 | 6.88 | 25.07 | 24.98 | 24.88 | 24.91 | 24.86 | 24.88 | 25 | 25.07 |
| H 4a | 4.92 | 27.94 | 26.79 | 27.33 | 27.04 | 26.64 | 27.36 | 26.86 | 27.95 |
| H 6 | 4.08 | 27.69 | 27.88 | 27.41 | 27.82 | 27.54 | 27.69 | 27.57 | 27.96 |
| H 7 | 6.06 | 25.41 | 25.72 | 25.79 | 25.8 | 25.78 | 25.8 | 25.68 | 25.45 |
| H 8 | 6.04 | 25.42 | 25.52 | 25.41 | 25.36 | 25.19 | 25.44 | 25.48 | 25.46 |
| H 8a | 4.97 | 27.31 | 26.67 | 26.89 | 26.98 | 27.04 | 26.9 | 26.69 | 27.31 |
| H 9 | 3.96 | 27.71 | 27.84 | 27.76 | 27.58 | 27.67 | 27.81 | 27.8 | 27.72 |
| H 10a-c | 1.25 | 30.63 | 30.66 | 30.66 | 30.61 | 30.67 | 30.64 | 30.67 | 30.63 |
| J 3,4 | 10 | 10.19 | 10.36 | 10.23 | 10.24 | 10.25 | 10.23 | 10.35 | 10.18 |
| J 3,4a | 1.6 | -0.91 | -3.09 | -3.33 | -3.39 | -3.42 | -3.33 | -2.93 | -0.93 |
| J 4,4a | 3.1 | 5.9 | 2.97 | 2.34 | 2.37 | 2.39 | 2.35 | 3.18 | 5.9 |
| J 4a,8a | 5.6 | 1.6 | 7.22 | 8.93 | 9.01 | 9.06 | 8.94 | 6.8 | 1.61 |
| J 4,8a | 1 | -0.28 | 0.96 | -0.26 | -0.26 | -0.26 | -0.26 | 0.88 | -0.28 |
| J 8,8a | 2.6 | 5.34 | 2.95 | 2.21 | 2.23 | 2.21 | 2.21 | 3.15 | 5.32 |
| J 6,8a | 2.6 | 2.58 | 3.22 | 4.41 | 3.08 | 3.43 | 4.34 | 3.1 | 2.61 |
| J 7,8 | 10.5 | 10.44 | 10.66 | 10.68 | 10.67 | 10.62 | 10.72 | 10.62 | 10.45 |
| J 6,8 | 2.6 | -3.2 | -3.42 | -3.36 | -2.91 | -3.2 | -3.27 | -3.48 | -3.16 |
| J 6,7 | 1.6 | 1.88 | 2.04 | 2.11 | 3.18 | 3.08 | 2.1 | 2.14 | 1.88 |
| J 6,9 | 4.2 | 2.19 | 7.07 | 2.32 | 6.66 | 2.03 | 7.22 | 2.34 | 6.91 |

[3] Compound **3**: NMR data from Maity, S.; Kanikarapu, S.; Murumudi, K.; Kunwar, A. C.; Yadav, J. S.; Mohapatra, D. K. *J. Org. Chem.* **2017**, *82*, 4561

**Table S4.** Boltzmann averaged GIAO isotropic magnetic shielding constants (σ) of isomers 1-8 calculated at the B3LYP/6-31G** level of theory for compound **4**[4]

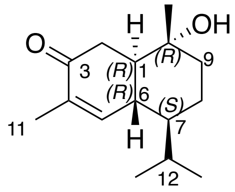


**4**

| | | Isotropic shielding constants | | | | | | | |
|---|---|---|---|---|---|---|---|---|---|
| **Nuclei** | **Experimental** | **Isomer 1** | **Isomer 2** | **Isomer 3** | **Isomer 4** | **Isomer 5** | **Isomer 6** | **Isomer 7** | **Isomer 8** |
| C 1 | 51.1 | 146.73 | 141.87 | 145.29 | 145.21 | 144.33 | 144.94 | 140.97 | 146.16 |
| C 2 | 38.3 | 153.58 | 153.67 | 154.85 | 152.15 | 151.5 | 156.32 | 152.82 | 153.24 |
| C 3 | 200.1 | -2.09 | -1.93 | -0.17 | -1.76 | 0.33 | -1.65 | -2.04 | -2.29 |
| C 4 | 135.4 | 59.24 | 57.39 | 58.88 | 55.81 | 55.03 | 57.84 | 58.32 | 60.48 |
| C 5 | 146 | 48.9 | 53.42 | 47.37 | 54.53 | 56.95 | 50.55 | 51.57 | 46.34 |
| C 6 | 40.8 | 146.91 | 149.64 | 153.41 | 151.86 | 151.35 | 152.25 | 150.45 | 148.17 |
| C 7 | 45 | 145.16 | 143.71 | 145.76 | 140.71 | 140.37 | 145.45 | 143.83 | 144.7 |
| C 8 | 21.5 | 163.98 | 168.04 | 169.17 | 164 | 166.05 | 167.7 | 169.12 | 164.66 |
| C 9 | 41.6 | 151.02 | 147.15 | 153.6 | 146.03 | 147.48 | 153.21 | 148.47 | 152.52 |
| C 10 | 71.2 | 120.69 | 121.63 | 121.02 | 120.83 | 120.81 | 121.53 | 122.14 | 121.41 |
| C 11 | 15.1 | 172.69 | 172.49 | 172.79 | 172.19 | 172.29 | 172.71 | 172.61 | 172.83 |
| C 12 | 26.2 | 161.73 | 161.67 | 160.41 | 159.71 | 159.58 | 160.75 | 161.43 | 162.17 |
| C 13 | 15.9 | 167.09 | 175.67 | 168.88 | 168.72 | 169.94 | 175.21 | 169.06 | 168.29 |
| C 14 | 21.4 | 168.65 | 169.08 | 175.09 | 169.77 | 168.71 | 168.92 | 175.52 | 167.24 |
| C 15 | 26.2 | 166.69 | 167.32 | 160.49 | 164.06 | 160.2 | 161.89 | 161.29 | 161.46 |
| H 1 | 1.83 | 29.26 | 29.67 | 29.44 | 29.44 | 29.84 | 29.44 | 30 | 29.59 |
| H 2a | 2.1 | 29.78 | 29.76 | 29.4 | 28.61 | 29.57 | 28.9 | 29.28 | 29.31 |
| H 2b | 2.75 | 28.93 | 28.87 | 29.51 | 29.59 | 28.98 | 29.37 | 29.21 | 29.21 |
| H 5 | 6.8 | 25.09 | 24.89 | 24.7 | 24.99 | 25.1 | 24.86 | 24.8 | 25.07 |
| H 6 | 2.07 | 29.22 | 29.41 | 28.86 | 28.63 | 28.8 | 29.4 | 28.83 | 28.6 |
| H 7 | 1.22 | 30.2 | 30.44 | 30.13 | 30.6 | 30.78 | 30.02 | 30.54 | 30.21 |
| H 8a | 1.84 | 29.84 | 30.16 | 29.84 | 30.01 | 30.06 | 30.18 | 29.85 | 29.69 |
| H 8b | 1.44 | 30.29 | 30.41 | 30.38 | 30.6 | 30.21 | 30.4 | 30.34 | 30.02 |
| H 9a | 1.69 | 30.67 | 30.43 | 30.02 | 30.44 | 30.22 | 30.65 | 30.18 | 29.88 |
| H 9b | 1.2 | 29.8 | 30.06 | 30.62 | 30.13 | 30.54 | 29.85 | 30.52 | 30.8 |
| H 11a-c | 1.79 | 30.05 | 30.05 | 30.05 | 30.02 | 30.03 | 30.06 | 30.06 | 30.07 |
| H 12 | 2.2 | 29.55 | 29.52 | 29.76 | 29.95 | 29.88 | 29.73 | 29.53 | 29.69 |
| H 13a-c | 0.83 | 30.78 | 30.86 | 30.82 | 30.76 | 30.79 | 30.83 | 30.81 | 30.8 |
| H 14a-c | 0.99 | 30.77 | 30.8 | 30.77 | 30.79 | 30.76 | 30.82 | 30.81 | 30.8 |
| H 15a-c | 1.16 | 30.64 | 30.66 | 30.74 | 30.67 | 30.69 | 30.58 | 30.71 | 30.68 |
| J 1,6 | 8.4 | 10.48 | 9.62 | 4.02 | 5.03 | 4.3 | 4.07 | 9 | 9.78 |
| J 1,2a | 13.8 | 12.79 | 12.84 | 13.89 | 1.74 | 5.36 | 4 | 3.1 | 3.57 |
| J 1,2b | 3 | 3.61 | 3.18 | 4.1 | 6.08 | 1.88 | 12.74 | 12.9 | 12.86 |

[4] Compound **4** and **5**: NMR data from He, K.; Zeng, L.; Shi, G.; Zhao, G. X.; Kozlowski, J. F.; McLaughlin, J. L. *J. Nat. Prod*. **1997**, *60*, 38

**Table S5.** Boltzmann averaged GIAO isotropic magnetic shielding constants (σ) of isomers 1-8 calculated at the B3LYP/6-31G** level of theory for compound **5**

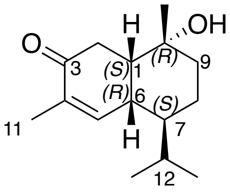


**5**

| | | Isotropic shielding constants | | | | | | | |
|---|---|---|---|---|---|---|---|---|---|
| **Nuclei** | **Experimental** | **Isomer 1** | **Isomer 2** | **Isomer 3** | **Isomer 4** | **Isomer 5** | **Isomer 6** | **Isomer 7** | **Isomer 8** |
| C 1 | 44.1 | 146.73 | 141.87 | 145.29 | 145.21 | 144.33 | 144.94 | 140.97 | 146.16 |
| C 2 | 34.28 | 153.58 | 153.67 | 154.85 | 152.15 | 151.5 | 156.32 | 152.82 | 153.24 |
| C 3 | 198.78 | -2.09 | -1.93 | -0.17 | -1.76 | 0.33 | -1.65 | -2.04 | -2.29 |
| C 4 | 134.4 | 59.24 | 57.39 | 58.88 | 55.81 | 55.03 | 57.84 | 58.32 | 60.48 |
| C 5 | 147.94 | 48.9 | 53.42 | 47.37 | 54.53 | 56.95 | 50.55 | 51.57 | 46.34 |
| C 6 | 36.75 | 146.91 | 149.64 | 153.41 | 151.86 | 151.35 | 152.25 | 150.45 | 148.17 |
| C 7 | 42.24 | 145.16 | 143.71 | 145.76 | 140.71 | 140.37 | 145.45 | 143.83 | 144.7 |
| C 8 | 20.6 | 163.98 | 168.04 | 169.17 | 164 | 166.05 | 167.7 | 169.12 | 164.66 |
| C 9 | 33.52 | 151.02 | 147.15 | 153.6 | 146.03 | 147.48 | 153.21 | 148.47 | 152.52 |
| C 10 | 70.32 | 120.69 | 121.63 | 121.02 | 120.83 | 120.81 | 121.53 | 122.14 | 121.41 |
| C 11 | 26.35 | 172.69 | 172.49 | 172.79 | 172.19 | 172.29 | 172.71 | 172.61 | 172.83 |
| C 12 | 20.4 | 161.73 | 161.67 | 160.41 | 159.71 | 159.58 | 160.75 | 161.43 | 162.17 |
| C 13 | 14.84 | 167.09 | 175.67 | 168.88 | 168.72 | 169.94 | 175.21 | 169.06 | 168.29 |
| C 14 | 18.94 | 168.65 | 169.08 | 175.09 | 169.77 | 168.71 | 168.92 | 175.52 | 167.24 |
| C 15 | 26.63 | 166.69 | 167.32 | 160.49 | 164.06 | 160.2 | 161.89 | 161.29 | 161.46 |
| H 1 | 2.13 | 29.26 | 29.67 | 29.44 | 29.44 | 29.84 | 29.44 | 30 | 29.59 |
| H 2a | 2.61 | 29.78 | 29.76 | 29.4 | 28.61 | 29.57 | 28.9 | 29.28 | 29.31 |
| H 2b | 2.41 | 28.93 | 28.87 | 29.51 | 29.59 | 28.98 | 29.37 | 29.21 | 29.21 |
| H 5 | 6.77 | 25.09 | 24.89 | 24.7 | 24.99 | 25.1 | 24.86 | 24.8 | 25.07 |
| H 6 | 2.28 | 29.22 | 29.41 | 28.86 | 28.63 | 28.8 | 29.4 | 28.83 | 28.6 |
| H 7 | 1.45 | 30.2 | 30.44 | 30.13 | 30.6 | 30.78 | 30.02 | 30.54 | 30.21 |
| H 8a | 1.59 | 29.84 | 30.16 | 29.84 | 30.01 | 30.06 | 30.18 | 29.85 | 29.69 |
| H 8b | 1.13 | 30.29 | 30.41 | 30.38 | 30.6 | 30.21 | 30.4 | 30.34 | 30.02 |
| H 9a | 1.5 | 30.67 | 30.43 | 30.02 | 30.44 | 30.22 | 30.65 | 30.18 | 29.88 |
| H 9b | 1.5 | 29.8 | 30.06 | 30.62 | 30.13 | 30.54 | 29.85 | 30.52 | 30.8 |
| H 11a-c | 1.72 | 30.05 | 30.05 | 30.05 | 30.02 | 30.03 | 30.06 | 30.06 | 30.07 |
| H 12 | 1.82 | 29.55 | 29.52 | 29.76 | 29.95 | 29.88 | 29.73 | 29.53 | 29.69 |
| H 13a-c | 0.86 | 30.78 | 30.86 | 30.82 | 30.76 | 30.79 | 30.83 | 30.81 | 30.8 |
| H 14a-c | 0.83 | 30.77 | 30.8 | 30.77 | 30.79 | 30.76 | 30.82 | 30.81 | 30.8 |
| H 15a-c | 1.25 | 30.64 | 30.66 | 30.74 | 30.67 | 30.69 | 30.58 | 30.71 | 30.68 |
| J 1,6 | 4.2 | 10.48 | 9.62 | 4.02 | 5.03 | 4.3 | 4.07 | 9 | 9.78 |
| J 1,2a | 4.2 | 12.79 | 12.84 | 13.89 | 1.74 | 5.36 | 4 | 3.1 | 3.57 |
| J 1,2b | 13.8 | 3.61 | 3.18 | 4.1 | 6.08 | 1.88 | 12.74 | 12.9 | 12.86 |
| J 5,6 | 5.9 | 3.04 | 2.63 | 6.07 | 3.13 | 2.96 | 6.02 | 2.68 | 3.06 |
| J 6,7 | 10.5 | 3.98 | 10.14 | 10.74 | 4.63 | 4.31 | 10.61 | 10.06 | 3.48 |
| J 7,12 | 13.8 | 6.43 | 3.28 | 2.73 | 8.68 | 8.59 | 2.89 | 3.25 | 7.14 |

**Table S6.** Boltzmann averaged GIAO isotropic magnetic shielding constants (σ) of isomers 1-16 calculated at the B3LYP/6-31G** level of theory for compound **6**[5]

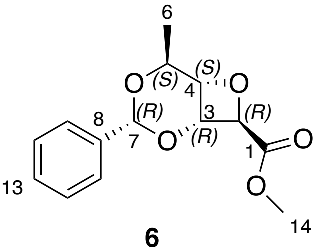


**6**

| Isotropic shielding constants | | | | | | | | | |
|---|---|---|---|---|---|---|---|---|---|
| **Nuclei** | **Experimental** | **Isomer 1** | **Isomer 2** | **Isomer 3** | **Isomer 4** | **Isomer 5** | **Isomer 6** | **Isomer 7** | **Isomer 8** |
| C 1 | 170.44 | 19.51 | 22.36 | 22.78 | 23.15 | 19.88 | 22.03 | 22.67 | 22.97 |
| C 2 | 81.67 | 102.46 | 104.31 | 100.08 | 100.54 | 103.45 | 103.74 | 98.62 | 99.19 |
| C 3 | 70.54 | 119.97 | 119.98 | 126.9 | 125.13 | 120.99 | 122.74 | 123 | 121.22 |
| C 4 | 79.31 | 111.74 | 113.36 | 109.06 | 111.43 | 112.22 | 111.51 | 106.99 | 110.43 |
| C 5 | 77.79 | 119.04 | 119.08 | 120.48 | 120.31 | 121.75 | 125.68 | 115.52 | 116.09 |
| C 6 | 20.17 | 175.48 | 175.5 | 176.7 | 175.95 | 175.31 | 172.87 | 170.81 | 170.69 |
| C 7 | 97.1 | 90.87 | 91.88 | 90.97 | 90.91 | 98.43 | 95.64 | 93.58 | 93.03 |
| C 8 | 137.27 | 54.95 | 55.32 | 56.63 | 56.74 | 54.41 | 55.08 | 56.92 | 56.73 |
| C 14 | 52.57 | 140.74 | 140.99 | 140.33 | 140.48 | 140.75 | 140.45 | 140.42 | 140.57 |
| H 2 | 5.53 | 26.36 | 26.45 | 26.49 | 26.48 | 26.42 | 26.29 | 26.37 | 26.43 |
| H 3 | 5.05 | 26.8 | 26.74 | 26.56 | 26.47 | 26.86 | 26.78 | 26.84 | 26.77 |
| H 4 | 4.55 | 26.99 | 27.35 | 25.68 | 27.02 | 27.06 | 27.32 | 26.09 | 27.5 |
| H 5 | 4.44 | 28.06 | 28.12 | 27.38 | 27.35 | 27.46 | 27.52 | 27.52 | 27.37 |
| H 6a-c | 1.42 | 30.5 | 30.41 | 30.59 | 30.49 | 30.46 | 30.62 | 30.55 | 30.57 |
| H 7 | 6.16 | 25.85 | 25.89 | 25.67 | 25.7 | 25.46 | 25.53 | 25.45 | 25.55 |
| H 14a-c | 3.82 | 28.09 | 28.58 | 27.98 | 28.05 | 28.13 | 28.21 | 27.98 | 28.06 |
| J 2,3 | 6.2 | 3.29 | 6.34 | 7.04 | 7.43 | 3.48 | 6.4 | 6.79 | 7.48 |
| J 2,4 | 1 | -0.72 | -0.56 | -0.87 | -0.43 | -0.84 | -0.49 | -0.83 | -0.36 |
| J 3,4 | 7.4 | 6.85 | 6.34 | 8.15 | 7.21 | 6.47 | 5.95 | 7.61 | 6.79 |
| J 4,5 | 6.2 | 1.11 | 1.23 | 6.74 | 6.51 | 2.05 | 6.56 | 8.04 | 7.95 |

| Isotropic shielding constants | | | | | | | | | |
|---|---|---|---|---|---|---|---|---|---|
| **Nuclei** | **Experimental** | **Isomer 9** | **Isomer 10** | **Isomer 11** | **Isomer 12** | **Isomer 13** | **Isomer 14** | **Isomer 15** | **Isomer 16** |
| C 1 | 170.44 | 23.29 | 23.21 | 19.69 | 20.42 | 23.14 | 22.68 | 21.37 | 19.8 |
| C 2 | 81.67 | 97.22 | 96.04 | 102.37 | 105.26 | 99.21 | 99.18 | 104.16 | 103.33 |
| C 3 | 70.54 | 114.61 | 116.06 | 124.43 | 122.32 | 125.14 | 127.64 | 120.09 | 119.94 |
| C 4 | 79.31 | 110.01 | 106.54 | 110.48 | 113.36 | 111.81 | 109.43 | 112.61 | 109.97 |
| C 5 | 77.79 | 111.71 | 110.98 | 117.82 | 114.8 | 121.39 | 122.49 | 124.23 | 126.1 |
| C 6 | 20.17 | 171.45 | 171.63 | 170.27 | 169.91 | 176.73 | 177.04 | 175.74 | 175.36 |
| C 7 | 97.1 | 88.04 | 87.61 | 90.69 | 91.42 | 91.48 | 90.57 | 95.53 | 94.19 |
| C 8 | 137.27 | 57.66 | 57.29 | 55.41 | 56.62 | 56.26 | 56.18 | 55.64 | 55.13 |
| C 14 | 52.57 | 140.56 | 140.32 | 140.51 | 140.61 | 140.52 | 140.33 | 140.19 | 140.65 |
| H 2 | 5.53 | 26.28 | 26.16 | 26.34 | 26.09 | 26.44 | 26.45 | 26.39 | 25.97 |
| H 3 | 5.05 | 27.21 | 27.31 | 26.75 | 26.72 | 26.57 | 26.67 | 26.76 | 26.72 |
| H 4 | 4.55 | 27.82 | 26.6 | 27.24 | 27.23 | 27.3 | 26.14 | 27.22 | 27.13 |
| H 5 | 4.44 | 27.45 | 27.6 | 26.8 | 27.55 | 27.41 | 27.54 | 27.95 | 28.2 |
| H 6a-c | 1.42 | 30.62 | 30.65 | 30.51 | 30.49 | 30.43 | 30.58 | 30.51 | 30.63 |
| H 7 | 6.16 | 25.65 | 25.78 | 25.33 | 25.69 | 25.39 | 25.35 | 25.1 | 25.07 |
| H 14a-c | 3.82 | 28.06 | 27.96 | 28.01 | 28.07 | 28.05 | 27.96 | 27.95 | 28.04 |
| J 2,3 | 6.2 | 7.51 | 6.9 | 7.32 | 5.52 | 7.33 | 6.94 | 6.18 | 4.06 |
| J 2,4 | 1 | -0.39 | -0.74 | -0.18 | -0.71 | -0.43 | -0.83 | -0.5 | -0.6 |
| J 3,4 | 7.4 | 6.04 | 6.74 | 6.72 | 7.2 | 7.1 | 8.01 | 6.22 | 7.28 |
| J 4,5 | 6.2 | 8.12 | 8.1 | 7.34 | 6.94 | 5.57 | 6.24 | 3.16 | 0.98 |

[5] Compounds **6** and **7**: NMR data from S. W Johnson, D. Angus, C. Taillefumier, J. H Jones, D. J Watkin, E. Floyd, J.G Buchanan, G. W.J Fleet, *Tetrahedron: Asymmetry,* **2000**, *11*, 20

**Table S7.** Boltzmann averaged GIAO isotropic magnetic shielding constants (σ) of isomers 1-16 calculated at the B3LYP/6-31G** level of theory for compound **7**

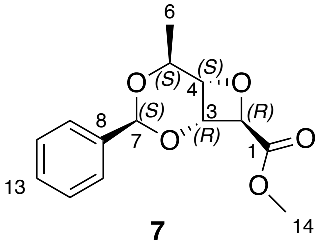


**7**

| Nuclei | Experimental | Isomer 1 | Isomer 2 | Isomer 3 | Isomer 4 | Isomer 5 | Isomer 6 | Isomer 7 | Isomer 8 |
|---|---|---|---|---|---|---|---|---|---|
| | | **Isotropic shielding constants** | | | | | | | |
| C 1 | 170.4 | 19.51 | 22.36 | 22.78 | 23.15 | 19.88 | 22.03 | 22.67 | 22.97 |
| C 2 | 84.1 | 102.46 | 104.31 | 100.08 | 100.54 | 103.45 | 103.74 | 98.62 | 99.19 |
| C 3 | 72.1 | 119.97 | 119.98 | 126.9 | 125.13 | 120.99 | 122.74 | 123 | 121.22 |
| C 4 | 79.4 | 111.74 | 113.36 | 109.06 | 111.43 | 112.22 | 111.51 | 106.99 | 110.43 |
| C 5 | 70.8 | 119.04 | 119.08 | 120.48 | 120.31 | 121.75 | 125.68 | 115.52 | 116.09 |
| C 6 | 16.2 | 175.48 | 175.5 | 176.7 | 175.95 | 175.31 | 172.87 | 170.81 | 170.69 |
| C 7 | 92.3 | 90.87 | 91.88 | 90.97 | 90.91 | 98.43 | 95.64 | 93.58 | 93.03 |
| C 8 | 138.3 | 54.95 | 55.32 | 56.63 | 56.74 | 54.41 | 55.08 | 56.92 | 56.73 |
| C 14 | 52.7 | 140.74 | 140.99 | 140.33 | 140.48 | 140.75 | 140.45 | 140.42 | 140.57 |
| H 2 | 5.08 | 26.36 | 26.45 | 26.49 | 26.48 | 26.42 | 26.29 | 26.37 | 26.43 |
| H 3 | 4.93 | 26.8 | 26.74 | 26.56 | 26.47 | 26.86 | 26.78 | 26.84 | 26.77 |
| H 4 | 4.69 | 26.99 | 27.35 | 25.68 | 27.02 | 27.06 | 27.32 | 26.09 | 27.5 |
| H 5 | 4.43 | 28.06 | 28.12 | 27.38 | 27.35 | 27.46 | 27.52 | 27.52 | 27.37 |
| H 6a-c | 1.38 | 30.5 | 30.41 | 30.59 | 30.49 | 30.46 | 30.62 | 30.55 | 30.57 |
| H 7 | 5.72 | 25.85 | 25.89 | 25.67 | 25.7 | 25.46 | 25.53 | 25.45 | 25.55 |
| H 14a-c | 3.83 | 28.09 | 28.58 | 27.98 | 28.05 | 28.13 | 28.21 | 27.98 | 28.06 |
| J 2,3 | 2.4 | 3.29 | 6.34 | 7.04 | 7.43 | 3.48 | 6.4 | 6.79 | 7.48 |
| J 2,4 | 1 | -0.72 | -0.56 | -0.87 | -0.43 | -0.84 | -0.49 | -0.83 | -0.36 |
| J 3,4 | 4.9 | 6.85 | 6.34 | 8.15 | 7.21 | 6.47 | 5.95 | 7.61 | 6.79 |
| J 4,5 | 2.2 | 1.11 | 1.23 | 6.74 | 6.51 | 2.05 | 6.56 | 8.04 | 7.95 |

| Nuclei | Experimental | Isomer 9 | Isomer 10 | Isomer 11 | Isomer 12 | Isomer 13 | Isomer 14 | Isomer 15 | Isomer 16 |
|---|---|---|---|---|---|---|---|---|---|
| | | **Isotropic shielding constants** | | | | | | | |
| C 1 | 170.4 | 23.29 | 23.21 | 19.69 | 20.42 | 23.14 | 22.68 | 21.37 | 19.8 |
| C 2 | 84.1 | 97.22 | 96.04 | 102.37 | 105.26 | 99.21 | 99.18 | 104.16 | 103.33 |
| C 3 | 72.1 | 114.61 | 116.06 | 124.43 | 122.32 | 125.14 | 127.64 | 120.09 | 119.94 |
| C 4 | 79.4 | 110.01 | 106.54 | 110.48 | 113.36 | 111.81 | 109.43 | 112.61 | 109.97 |
| C 5 | 70.8 | 111.71 | 110.98 | 117.82 | 114.8 | 121.39 | 122.49 | 124.23 | 126.1 |
| C 6 | 16.2 | 171.45 | 171.63 | 170.27 | 169.91 | 176.73 | 177.04 | 175.74 | 175.36 |
| C 7 | 92.3 | 88.04 | 87.61 | 90.69 | 91.42 | 91.48 | 90.57 | 95.53 | 94.19 |
| C 8 | 138.3 | 57.66 | 57.29 | 55.41 | 56.62 | 56.26 | 56.18 | 55.64 | 55.13 |
| C 14 | 52.7 | 140.56 | 140.32 | 140.51 | 140.61 | 140.52 | 140.33 | 140.19 | 140.65 |
| H 2 | 5.08 | 26.28 | 26.16 | 26.34 | 26.09 | 26.44 | 26.45 | 26.39 | 25.97 |
| H 3 | 4.93 | 27.21 | 27.31 | 26.75 | 26.72 | 26.57 | 26.67 | 26.76 | 26.72 |
| H 4 | 4.69 | 27.82 | 26.6 | 27.24 | 27.23 | 27.3 | 26.14 | 27.22 | 27.13 |
| H 5 | 4.43 | 27.45 | 27.6 | 26.8 | 27.55 | 27.41 | 27.54 | 27.95 | 28.2 |
| H 6a-c | 1.38 | 30.62 | 30.65 | 30.51 | 30.49 | 30.43 | 30.58 | 30.51 | 30.63 |
| H 7 | 5.72 | 25.65 | 25.78 | 25.33 | 25.69 | 25.39 | 25.35 | 25.1 | 25.07 |
| H 14a-c | 3.83 | 28.06 | 27.96 | 28.01 | 28.07 | 28.05 | 27.96 | 27.95 | 28.04 |
| J 2,3 | 2.4 | 7.51 | 6.9 | 7.32 | 5.52 | 7.33 | 6.94 | 6.18 | 4.06 |
| J 2,4 | 1 | -0.39 | -0.74 | -0.18 | -0.71 | -0.43 | -0.83 | -0.5 | -0.6 |
| J 3,4 | 4.9 | 6.04 | 6.74 | 6.72 | 7.2 | 7.1 | 8.01 | 6.22 | 7.28 |
| J 4,5 | 2.2 | 8.12 | 8.1 | 7.34 | 6.94 | 5.57 | 6.24 | 3.16 | 0.98 |

**Table S8.** Boltzmann averaged GIAO isotropic magnetic shielding constants (σ) of isomers 1-16 calculated at the B3LYP/6-31G** level of theory for compound **8**[6]

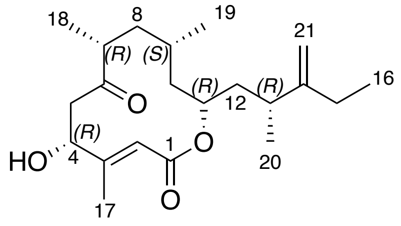


**8**

| Isotropic shielding constants | | | | | | | | | |
|---|---|---|---|---|---|---|---|---|---|
| **Nuclei** | **Experimental** | **Isomer 1** | **Isomer 2** | **Isomer 3** | **Isomer 4** | **Isomer 5** | **Isomer 6** | **Isomer 7** | **Isomer 8** |
| C 1 | 167.6 | 29.4 | 27.72 | 28.16 | 29.04 | 28.48 | 29.88 | 27.59 | 30.35 |
| C 2 | 116.9 | 72.61 | 72.14 | 72.49 | 70.38 | 70.98 | 71.43 | 70.96 | 71.69 |
| C 3 | 154.7 | 39.89 | 43.6 | 41.73 | 39.68 | 39.49 | 40.02 | 42.46 | 40.62 |
| C 4 | 73 | 116.86 | 117.34 | 115.6 | 115.7 | 117.22 | 117.95 | 116.25 | 115.84 |
| C 5 | 44.7 | 150.22 | 146.9 | 153.91 | 147.62 | 148.36 | 146.42 | 152.6 | 148.66 |
| C 6 | 217 | -23.01 | -27.42 | -25.99 | -17.98 | -24.28 | -25.65 | -25.58 | -19.34 |
| C 7 | 50.5 | 144.76 | 139.3 | 144.8 | 144.24 | 143.32 | 143.93 | 143.24 | 144.77 |
| C 8 | 39.8 | 147.04 | 147.16 | 148.74 | 148.79 | 148.69 | 152.47 | 148.3 | 148.2 |
| C 9 | 32.9 | 163.74 | 156.94 | 164.45 | 161.14 | 160.85 | 160.92 | 160.38 | 162.6 |
| C 10 | 44.9 | 151.24 | 142.74 | 147.41 | 148.18 | 147.93 | 152.76 | 146.85 | 151.69 |
| C 11 | 74.4 | 120.95 | 120.05 | 120.92 | 115.89 | 117.17 | 123.45 | 116.59 | 121.13 |
| C 12 | 41.2 | 147.16 | 146.75 | 148.16 | 146.95 | 146.68 | 147.72 | 147.78 | 149.02 |
| C 13 | 36.9 | 151.69 | 152.62 | 152.3 | 151.86 | 152.63 | 151.07 | 151.92 | 151.36 |
| C 14 | 155.2 | 39.08 | 39.2 | 39.24 | 39.03 | 37.59 | 39.44 | 39.04 | 38.99 |
| C 15 | 26.2 | 160.48 | 160.04 | 161.19 | 161.11 | 161.75 | 164.77 | 163.8 | 163.5 |
| C 16 | 12.3 | 177.5 | 177.44 | 177.77 | 177.57 | 177.3 | 178.11 | 177.74 | 177.75 |
| C 17 | 16.7 | 173.42 | 174.38 | 174.25 | 176.88 | 171.53 | 169.51 | 172.27 | 178.76 |
| C 18 | 17.9 | 172.74 | 172.13 | 172.85 | 173.17 | 173.3 | 176.28 | 173.23 | 172.75 |
| C 19 | 23.1 | 165.66 | 169.34 | 168.96 | 168.8 | 168.41 | 168.49 | 170.25 | 167.59 |
| C 20 | 21.1 | 167.8 | 167.47 | 168.02 | 167.99 | 171.06 | 169.9 | 169.9 | 170.74 |
| C 21 | 107.1 | 85.75 | 86.87 | 86.29 | 86.3 | 86.15 | 85.8 | 85.79 | 85.69 |
| H 2 | 5.93 | 25.48 | 25.48 | 25.5 | 25.83 | 25.55 | 25.64 | 25.63 | 25.87 |
| H 4 | 4.46 | 27.43 | 27.43 | 27.48 | 27.13 | 27.42 | 27.41 | 27.42 | 27.16 |
| H 5a | 3.07 | 28.75 | 28.84 | 28.88 | 29.33 | 28.81 | 28.75 | 28.94 | 29.31 |
| H 5b | 2.52 | 29.68 | 29.37 | 29.64 | 28.78 | 29.52 | 29.5 | 29.49 | 28.88 |
| H 7 | 2.16 | 29.52 | 29.27 | 29.33 | 29.35 | 29.16 | 28.79 | 29.28 | 29.55 |
| H 8a | 0.94 | 31 | 30.87 | 30.6 | 29.8 | 31.02 | 30.29 | 30.06 | 30.17 |
| H 8b | 2.09 | 29.97 | 29.38 | 30.78 | 31.08 | 29.8 | 30.29 | 30.8 | 30.57 |
| H 9 | 0.97 | 29.41 | 30.85 | 29.63 | 30.13 | 30.12 | 30 | 30.23 | 29.56 |
| H 10a | 1.44 | 31.08 | 30.22 | 30.61 | 30.05 | 30.63 | 30.24 | 30.39 | 30.11 |
| H 10b | 1.29 | 30.14 | 30.58 | 30.18 | 31.07 | 30.38 | 29.98 | 30.38 | 30.89 |
| H 11 | 5.03 | 26.53 | 26.51 | 26.75 | 27.27 | 27.13 | 26.82 | 27.3 | 26.62 |
| H 12a | 1.55 | 29.7 | 30.15 | 29.86 | 29.15 | 30.33 | 30.45 | 30.11 | 30.39 |

[6] Compound **8**: NMR data from Hangyou, M.; Ishiyama, H.; Takahashi, Y.; Kobayashi, *J. Org. Lett.* **2009**, *11*, 5046

| | | | | | | | | | |
|---|---|---|---|---|---|---|---|---|---|
| H 12b | 1.62 | 30.25 | 30.14 | 30.27 | 30.34 | 29.48 | 29.75 | 29.6 | 29.63 |
| H 13 | 2.23 | 29.42 | 29.46 | 29.29 | 29.22 | 29.31 | 29.39 | 29.39 | 29.47 |
| H 15 | 2.01 | 29.62 | 29.6 | 29.58 | 29.6 | 29.63 | 29.42 | 29.69 | 29.69 |
| H 16a-c | 1.04 | 30.69 | 30.69 | 30.69 | 30.69 | 30.69 | 30.67 | 30.69 | 30.69 |
| H 17a-c | 2.12 | 29.66 | 29.81 | 29.75 | 29.56 | 29.74 | 29.8 | 29.84 | 29.56 |
| H 18a-c | 1.08 | 30.74 | 30.77 | 30.79 | 30.75 | 30.75 | 30.77 | 30.78 | 30.73 |
| H 19a-c | 0.92 | 30.78 | 30.91 | 30.82 | 30.89 | 30.85 | 30.76 | 30.86 | 30.82 |
| H 20a-c | 1.02 | 30.69 | 30.72 | 30.7 | 30.68 | 30.55 | 30.63 | 30.62 | 30.69 |
| H 21a | 4.78 | 26.61 | 26.61 | 26.64 | 26.65 | 26.77 | 26.8 | 26.78 | 26.78 |
| H 21b | 4.76 | 26.72 | 26.77 | 26.73 | 26.7 | 26.76 | 26.7 | 26.74 | 26.73 |
| J 4,5a | 3.1 | 2.84 | 2.82 | 1.97 | 4.43 | 2.95 | 3.22 | 2.42 | 4.25 |
| J 4,5b | 5.45 | 5.34 | 5.04 | 5.98 | 11.3 | 5.03 | 4.82 | 6.49 | 10.11 |
| J 7,8a | 7.3 | 1.84 | 3.87 | 11.36 | 9.71 | 1.48 | 9.93 | 10.41 | 6.32 |
| J 7,8b | 2.8 | 8.9 | 5.24 | 1.67 | 1.54 | 9.67 | 1.49 | 2.09 | 4.23 |
| J 10a,11 | 11.4 | 1.02 | 9.52 | 2.69 | 9.7 | 3.62 | 0.93 | 2.41 | 4.76 |
| J 10b,11 | 3.6 | 5.21 | 2.17 | 4.89 | 1.16 | 7.02 | 8.75 | 10.08 | 2.7 |
| J 9,10a | 7 | 8.37 | 4.14 | 5.4 | 0.87 | 6.26 | 3.31 | 4.48 | 1.61 |
| J 9,10b | 2.3 | 0.73 | 4.65 | 2.82 | 9.34 | 3.13 | 3.38 | 3.54 | 7.08 |
| J 11,12a | 8.7 | 10.32 | 9.25 | 9.81 | 10.23 | 3.62 | 5.93 | 6.53 | 4.27 |
| J 11,12b | 3.6 | 0.99 | 1.31 | 1.78 | 2.44 | 9.06 | 6.03 | 6.27 | 8.39 |
| J 12a,13 | 5 | 3.36 | 3.17 | 3.62 | 3.24 | 9.32 | 6.79 | 6.88 | 7.43 |
| J 12b,13 | 9 | 10.25 | 10.27 | 10.18 | 10.36 | 4.09 | 7.33 | 7.33 | 6.05 |

**Isotropic shielding constants**

| Nuclei | Experimental | Isomer 9 | Isomer 10 | Isomer 11 | Isomer 12 | Isomer 13 | Isomer 14 | Isomer 15 | Isomer 16 |
|---|---|---|---|---|---|---|---|---|---|
| C 1 | 167.6 | 29.62 | 27.6 | 26.29 | 28.9 | 28.64 | 29.97 | 27.5 | 30.32 |
| C 2 | 116.9 | 72.61 | 71.81 | 72.6 | 70.1 | 71.51 | 73.03 | 72.62 | 71.37 |
| C 3 | 154.7 | 40.16 | 45.84 | 45.4 | 39.86 | 38.9 | 40.05 | 41 | 40.31 |
| C 4 | 73 | 116.85 | 117.72 | 116 | 115.76 | 117.19 | 117.6 | 117.11 | 115.16 |
| C 5 | 44.7 | 150.15 | 147.11 | 153.37 | 147.63 | 148.39 | 147.23 | 151.51 | 148.95 |
| C 6 | 217 | -23.02 | -26.99 | -26.54 | -17.98 | -24.25 | -25.7 | -26.54 | -17.51 |
| C 7 | 50.5 | 144.69 | 138.81 | 146.97 | 144.08 | 143.38 | 143.11 | 143.53 | 145.53 |
| C 8 | 39.8 | 147.1 | 147.55 | 152.2 | 148.74 | 148.64 | 152.81 | 147.82 | 147.07 |
| C 9 | 32.9 | 163.82 | 156.23 | 163.37 | 161.02 | 161.03 | 160.25 | 161.1 | 163.75 |
| C 10 | 44.9 | 151.94 | 143.71 | 149.36 | 148.52 | 148.04 | 151.46 | 144.86 | 150.81 |
| C 11 | 74.4 | 121.63 | 118.77 | 119.95 | 116.38 | 116.75 | 120.37 | 116.8 | 120.58 |
| C 12 | 41.2 | 148.9 | 147.26 | 149.45 | 146.94 | 146.84 | 147.02 | 146.79 | 147.34 |
| C 13 | 36.9 | 151.82 | 152.07 | 149.95 | 152.6 | 152.54 | 152.52 | 152.45 | 151.59 |
| C 14 | 155.2 | 38.31 | 38.42 | 41.18 | 37.85 | 38.85 | 39.27 | 38.78 | 39.24 |
| C 15 | 26.2 | 162.38 | 163.07 | 167.71 | 161.9 | 160.39 | 161.37 | 160.96 | 160.66 |
| C 16 | 12.3 | 177.67 | 177.64 | 178.89 | 177.21 | 177.25 | 177.61 | 177.86 | 177.46 |
| C 17 | 16.7 | 173.47 | 175.09 | 174.97 | 176.49 | 172.22 | 173 | 173.31 | 179.76 |
| C 18 | 17.9 | 172.73 | 172.43 | 171.52 | 173.28 | 173.18 | 175.34 | 173.33 | 172.82 |
| C 19 | 23.1 | 165.76 | 168.75 | 170.14 | 168.55 | 168.69 | 168.73 | 170.77 | 165.67 |
| C 20 | 21.1 | 171.28 | 170.85 | 168.78 | 171.13 | 167.73 | 168.08 | 167.69 | 167.98 |
| C 21 | 107.1 | 85.97 | 86.01 | 85.49 | 85.99 | 86.48 | 86.1 | 87.35 | 85.61 |
| H 2 | 5.93 | 25.49 | 25.47 | 25.52 | 25.82 | 25.57 | 25.6 | 25.71 | 25.85 |
| H 4 | 4.46 | 27.43 | 27.43 | 27.51 | 27.09 | 27.41 | 27.38 | 27.39 | 27.13 |
| H 5a | 3.07 | 28.75 | 28.82 | 28.75 | 29.3 | 28.81 | 28.81 | 28.89 | 29.32 |

| | | | | | | | | | |
|---|---|---|---|---|---|---|---|---|---|
| H 5b | 2.52 | 29.68 | 29.37 | 29.71 | 28.79 | 29.54 | 29.54 | 29.55 | 28.85 |
| H 7 | 2.16 | 29.51 | 29.28 | 29.24 | 29.32 | 29.2 | 29.22 | 29.2 | 29.62 |
| H 8a | 0.94 | 31 | 30.83 | 30.36 | 29.79 | 31.04 | 30.56 | 30.06 | 29.9 |
| H 8b | 2.09 | 29.94 | 29.31 | 30.99 | 31.07 | 29.82 | 29.76 | 30.75 | 31.06 |
| H 9 | 0.97 | 29.4 | 30.84 | 29.33 | 30.13 | 30.09 | 30.16 | 30.25 | 29.5 |
| H 10a | 1.44 | 30.98 | 30.24 | 30.32 | 30.11 | 31.02 | 30.56 | 30.62 | 30.16 |
| H 10b | 1.29 | 30.13 | 30.48 | 30.27 | 30.94 | 30.05 | 29.71 | 30.23 | 31.08 |
| H 11 | 5.03 | 26.47 | 26.77 | 27.33 | 27.23 | 27.15 | 27.09 | 27.2 | 26.57 |
| H 12a | 1.55 | 29.6 | 29.92 | 29.46 | 29.33 | 30.29 | 30.42 | 30.25 | 30.24 |
| H 12b | 1.62 | 30.38 | 30.43 | 30.35 | 30.3 | 29.27 | 29.03 | 29.5 | 29.73 |
| H 13 | 2.23 | 29.42 | 29.43 | 29.39 | 29.3 | 29.21 | 29.3 | 29.2 | 29.49 |
| H 15 | 2.01 | 29.65 | 29.59 | 29.27 | 29.63 | 29.59 | 29.61 | 29.56 | 29.71 |
| H 16a-c | 1.04 | 30.68 | 30.69 | 30.67 | 30.7 | 30.67 | 30.66 | 30.67 | 30.71 |
| H 17a-c | 2.12 | 29.66 | 29.87 | 29.83 | 29.59 | 29.7 | 29.74 | 29.79 | 29.5 |
| H 18a-c | 1.08 | 30.73 | 30.75 | 30.74 | 30.74 | 30.75 | 30.78 | 30.78 | 30.73 |
| H 19a-c | 0.92 | 30.78 | 30.88 | 30.81 | 30.86 | 30.88 | 30.81 | 30.86 | 30.78 |
| H 20a-c | 1.02 | 30.66 | 30.58 | 30.7 | 30.6 | 30.68 | 30.7 | 30.72 | 30.71 |
| H 21a | 4.78 | 26.76 | 26.79 | 26.87 | 26.77 | 26.62 | 26.61 | 26.66 | 26.63 |
| H 21b | 4.76 | 26.72 | 26.74 | 26.69 | 26.77 | 26.72 | 26.68 | 26.75 | 26.74 |
| J 4,5a | 3.1 | 2.84 | 2.89 | 2.17 | 4.71 | 2.94 | 3 | 2.47 | 5.46 |
| J 4,5b | 5.45 | 5.33 | 4.92 | 5.82 | 11.25 | 5.1 | 5.15 | 5.54 | 10.56 |
| J 7,8a | 7.3 | 1.74 | 4.27 | 11.85 | 9.72 | 1.5 | 8.72 | 11.52 | 9.2 |
| J 7,8b | 2.8 | 8.98 | 5.19 | 2.22 | 1.52 | 9.67 | 2.37 | 2.21 | 1.6 |
| J 10a,11 | 11.4 | 1.06 | 9.57 | 8.02 | 9.66 | 1.13 | 0.82 | 2.48 | 5.25 |
| J 10b,11 | 3.6 | 5.28 | 1.91 | 3.85 | 1.12 | 9.67 | 8.23 | 10.12 | 1.02 |
| J 9,10a | 7 | 8.34 | 4.48 | 3.93 | 0.74 | 9.17 | 4.28 | 4.99 | 0.66 |
| J 9,10b | 2.3 | 0.76 | 4.4 | 8.19 | 9.06 | 0.74 | 2.86 | 3.15 | 8.41 |
| J 11,12a | 8.7 | 9.77 | 8.19 | 3.33 | 8.89 | 2.27 | 2.49 | 1.76 | 1.14 |
| J 11,12b | 3.6 | 2.68 | 3.53 | 10.34 | 4.18 | 10.05 | 10.09 | 9.54 | 10.16 |
| J 12a,13 | 5 | 4.49 | 4.85 | 11.82 | 4.58 | 10.47 | 10.62 | 10.75 | 9.97 |
| J 12b,13 | 9 | 8.49 | 8.48 | 3.56 | 9 | 3.09 | 3.26 | 2.94 | 3.65 |

**Table S9.** Boltzmann averaged GIAO isotropic magnetic shielding constants (σ) of isomers 1-8 calculated at the B3LYP/6-31G** level of theory for compound **9**[7]

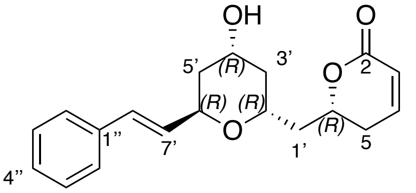


**9**

| Isotropic shielding constants | | | | | | | | | |
|---|---|---|---|---|---|---|---|---|---|
| **Nuclei** | **Experimental** | **Isomer 1** | **Isomer 2** | **Isomer 3** | **Isomer 4** | **Isomer 5** | **Isomer 6** | **Isomer 7** | **Isomer 8** |
| C 2 | 164.7 | 33.71 | 33.28 | 34.11 | 34.25 | 34.48 | 34.5 | 33.95 | 34 |
| C 3 | 121.5 | 72.08 | 71.92 | 72.08 | 72.11 | 71.76 | 71.8 | 72.49 | 72.55 |
| C 4 | 145.7 | 52.45 | 52.38 | 52.87 | 52.78 | 53.57 | 53.57 | 52.1 | 51.94 |
| C 5 | 29.5 | 159.74 | 159.66 | 161.14 | 160.7 | 159.88 | 159.79 | 160.9 | 161.1 |
| C 6 | 75.2 | 117.6 | 118.69 | 116.74 | 117.12 | 117.92 | 117.26 | 116.46 | 116.38 |
| C 1' | 40.6 | 147.44 | 145.34 | 148.83 | 149.02 | 146.45 | 146.5 | 149.45 | 151.11 |
| C 2' | 71.6 | 125.51 | 132.87 | 122.55 | 119.64 | 120.66 | 122.74 | 129.98 | 125.12 |
| C 3' | 40.8 | 149.4 | 151.72 | 150.74 | 149.14 | 149.62 | 149.25 | 152.29 | 150.8 |
| C 4' | 68.1 | 127.3 | 127.02 | 126.44 | 124.32 | 124.46 | 126.42 | 127.03 | 127.39 |
| C 5' | 41.4 | 153.83 | 154.42 | 153.02 | 149.31 | 148.21 | 153.37 | 153.91 | 152.9 |
| C 6' | 76.3 | 120.8 | 120.87 | 117.88 | 114.99 | 115.46 | 118.04 | 120.04 | 121.17 |
| C 7' | 129.5 | 63.63 | 67.05 | 60.91 | 62.29 | 61.5 | 60.63 | 63.26 | 62.81 |
| C 8' | 130.7 | 62.44 | 63.99 | 63.7 | 63.28 | 63.01 | 63.65 | 62.74 | 62.54 |
| C 1'' | 136.8 | 58.8 | 61.13 | 58.37 | 58.98 | 58.48 | 58.21 | 59.33 | 58.74 |
| C 2''-6'' | 126.7 | 69.69 | 69.275 | 70.095 | 70.06 | 70.025 | 70.07 | 69.845 | 70 |
| C 3''-5'' | 128.8 | 68.465 | 68.25 | 68.39 | 68.29 | 68.36 | 68.425 | 68.22 | 68.345 |
| C 4'' | 128 | 70.72 | 70.13 | 70.72 | 70.43 | 70.54 | 70.79 | 70.28 | 70.57 |
| H 3 | 6.03 | 25.76 | 25.73 | 25.85 | 25.84 | 25.85 | 25.85 | 25.83 | 25.8 |
| H 4 | 6.89 | 24.98 | 24.96 | 25.03 | 25.04 | 25.09 | 25.09 | 25.02 | 24.98 |
| H 5a | 2.4 | 29.75 | 29.89 | 29.72 | 29.85 | 29.85 | 29.76 | 29.8 | 29.86 |
| H 5b | 2.51 | 29.57 | 29.54 | 29.28 | 29.25 | 29.57 | 29.55 | 28.86 | 28.87 |
| H 6 | 4.67 | 26.8 | 26.6 | 26.97 | 26.97 | 26.87 | 26.91 | 27.05 | 27.05 |
| H 1'a | 2.18 | 30.15 | 30.22 | 30.08 | 29.98 | 30.1 | 30.14 | 30.05 | 29.62 |
| H 1'b | 1.89 | 29.55 | 30.02 | 29.81 | 29.81 | 29.95 | 30.02 | 29.86 | 29.78 |
| H 2' | 3.73 | 27.37 | 27.11 | 27.42 | 28.08 | 27.91 | 27.33 | 27.1 | 27.36 |
| H 3'a | 2.04 | 30.25 | 30.4 | 30.43 | 29.93 | 30.71 | 30.3 | 30.22 | 30.15 |
| H 3'b | 1.3 | 30.37 | 30.22 | 30.15 | 30.44 | 29.9 | 30.49 | 30.19 | 30.26 |
| H 4' | 3.92 | 27.67 | 27.8 | 27.55 | 27.99 | 27.89 | 27.55 | 27.68 | 27.67 |
| H 5'a | 1.37 | 29.91 | 29.92 | 30.24 | 30.58 | 30.05 | 29.99 | 29.84 | 30.14 |
| H 5'b | 2.1 | 30.04 | 29.85 | 30.07 | 29.89 | 30.53 | 30.26 | 30.12 | 29.93 |
| H 6' | 4.01 | 26.95 | 27.01 | 27.21 | 27.89 | 27.83 | 27.17 | 27.22 | 26.94 |
| H 7' | 6.19 | 25.89 | 25.62 | 25.75 | 25.64 | 25.69 | 25.75 | 25.26 | 25.77 |
| H 8' | 6.58 | 24.76 | 24.72 | 24.9 | 25.01 | 24.95 | 24.89 | 24.83 | 24.8 |
| H 2''-6'' | 7.38 | 24.285 | 24.15 | 24.505 | 24.49 | 24.505 | 24.515 | 24.415 | 24.465 |
| H 3''-5'' | 7.31 | 24.39 | 24.355 | 24.415 | 24.405 | 24.405 | 24.42 | 24.385 | 24.405 |

[7] Compound **9**: NMR data from Della-Felice, F.; de Assis, F. F.; Sarotti, A. M.; Pilli, R. A. *Synthesis* **2019**, *51*, 154

| H 4'' | 7.24 | 24.55 | 24.54 | 24.54 | 24.53 | 24.53 | 24.54 | 24.52 | 24.54 |
|---|---|---|---|---|---|---|---|---|---|
| J 3,4 | 9.7 | 10.14 | 10.1 | 10.07 | 10.08 | 10.1 | 10.09 | 10.08 | 10.1 |
| J 4,5a | 2.5 | 3.37 | 2.89 | 2.99 | 2.92 | 3.07 | 3.06 | 2.91 | 2.88 |
| J 4,5b | 6 | 6.05 | 6.51 | 6.44 | 6.5 | 6.35 | 6.37 | 6.5 | 6.5 |
| J 5a,6 | 4.1 | 3.3 | 2.74 | 2.78 | 2.68 | 2.99 | 3.03 | 2.75 | 2.7 |
| J 5b,6 | 11.6 | 10 | 11.5 | 11.45 | 11.66 | 10.93 | 10.93 | 11.98 | 12.14 |
| J 6,1'a | 6 | 1.06 | 1.27 | 4.97 | 3.68 | 1.85 | 2.61 | 5.71 | 4.49 |
| J 6,1'b | 6 | 9.7 | 9.86 | 5.52 | 5.92 | 9.19 | 8.21 | 3.56 | 3.81 |
| J 1'a,2' | 8 | 8.25 | 9.54 | 3.21 | 3.4 | 9.08 | 8.84 | 2.42 | 4.47 |
| J 1'b,2' | 5.5 | 3.75 | 1.52 | 7.5 | 6.01 | 1.8 | 2.02 | 8.66 | 7.33 |
| J 2',3'a | 1.8 | 8.16 | 11.02 | 1.62 | 1.52 | 10.35 | 10.42 | 2.28 | 3.34 |
| J 2',3'b | 11.3 | 2.91 | 1.94 | 10.55 | 10.54 | 1.57 | 1.67 | 9.69 | 7.25 |
| J 4',3'a | 4.3 | 8.3 | 3.06 | 2.92 | 4.24 | 9.97 | 2.46 | 3.91 | 3.53 |
| J 4',3'b | 11.3 | 3.61 | 2.95 | 2.52 | 9.82 | 4.34 | 2.88 | 3.09 | 7.55 |
| J 4',5'a | 11.3 | 3.85 | 2.7 | 2.86 | 9.9 | 4.41 | 2.79 | 3.43 | 7.99 |
| J 4',5'b | 4.3 | 8.83 | 3.38 | 2.83 | 4.36 | 9.9 | 2.96 | 3.51 | 3.68 |
| J 6',5'a | 11.3 | 3.95 | 0.98 | 11.1 | 10.82 | 1.73 | 1.83 | 6.7 | 4.9 |
| J 6',5'b | 1.8 | 5.36 | 7.63 | 1.84 | 1.74 | 10.81 | 11.09 | 2.11 | 4.81 |
| J 6',7' | 5.8 | 3.47 | 2.99 | 5.59 | 6.17 | 6.31 | 5.67 | 5.59 | 4.42 |
| J 7',8' | 16 | 15.39 | 15.75 | 15.06 | 15.05 | 15.03 | 15.06 | 15.31 | 15.26 |

**Table S10.** Boltzmann averaged GIAO isotropic magnetic shielding constants (σ) of isomers 1-8 calculated at the B3LYP/6-31G** level of theory for compound **10**[8]

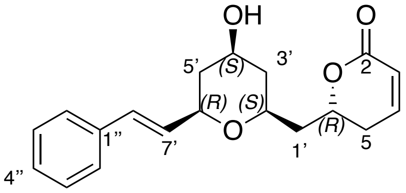


**10**

| | | Isotropic shielding constants | | | | | | | |
|---|---|---|---|---|---|---|---|---|---|
| **Nuclei** | **Experimental** | **Isomer 1** | **Isomer 2** | **Isomer 3** | **Isomer 4** | **Isomer 5** | **Isomer 6** | **Isomer 7** | **Isomer 8** |
| C 2 | 164.4 | 33.71 | 33.28 | 34.11 | 34.25 | 34.48 | 34.5 | 33.95 | 34 |
| C 3 | 121.8 | 72.08 | 71.92 | 72.08 | 72.11 | 71.76 | 71.8 | 72.49 | 72.55 |
| C 4 | 145.3 | 52.45 | 52.38 | 52.87 | 52.78 | 53.57 | 53.57 | 52.1 | 51.94 |
| C 5 | 30.3 | 159.74 | 159.66 | 161.14 | 160.7 | 159.88 | 159.79 | 160.9 | 161.1 |
| C 6 | 74.7 | 117.6 | 118.69 | 116.74 | 117.12 | 117.92 | 117.26 | 116.46 | 116.38 |
| C 1' | 41.9 | 147.44 | 145.34 | 148.83 | 149.02 | 146.45 | 146.5 | 149.45 | 151.11 |
| C 2' | 65.6 | 125.51 | 132.87 | 122.55 | 119.64 | 120.66 | 122.74 | 129.98 | 125.12 |
| C 3' | 41.8 | 149.4 | 151.72 | 150.74 | 149.14 | 149.62 | 149.25 | 152.29 | 150.8 |
| C 4' | 64.7 | 127.3 | 127.02 | 126.44 | 124.32 | 124.46 | 126.42 | 127.03 | 127.39 |
| C 5' | 38.7 | 153.83 | 154.42 | 153.02 | 149.31 | 148.21 | 153.37 | 153.91 | 152.9 |
| C 6' | 72.7 | 120.8 | 120.87 | 117.88 | 114.99 | 115.46 | 118.04 | 120.04 | 121.17 |
| C 7' | 129 | 63.63 | 67.05 | 60.91 | 62.29 | 61.5 | 60.63 | 63.26 | 62.81 |
| C 8' | 131.8 | 62.44 | 63.99 | 63.7 | 63.28 | 63.01 | 63.65 | 62.74 | 62.54 |
| C 1'' | 136.7 | 58.8 | 61.13 | 58.37 | 58.98 | 58.48 | 58.21 | 59.33 | 58.74 |
| 2''-6'' | 126.8 | 69.69 | 69.275 | 70.095 | 70.06 | 70.025 | 70.07 | 69.845 | 70 |
| 3''-5'' | 128.9 | 68.465 | 68.25 | 68.39 | 68.29 | 68.36 | 68.425 | 68.22 | 68.345 |
| C 4'' | 128 | 70.72 | 70.13 | 70.72 | 70.43 | 70.54 | 70.79 | 70.28 | 70.57 |
| H 3 | 6.05 | 25.76 | 25.73 | 25.85 | 25.84 | 25.85 | 25.85 | 25.83 | 25.8 |
| H 4 | 6.89 | 24.98 | 24.96 | 25.03 | 25.04 | 25.09 | 25.09 | 25.02 | 24.98 |
| H 5a | 2.39 | 29.75 | 29.89 | 29.72 | 29.85 | 29.85 | 29.76 | 29.8 | 29.86 |
| H 5b | 2.39 | 29.57 | 29.54 | 29.28 | 29.25 | 29.57 | 29.55 | 28.86 | 28.87 |
| H 6 | 4.8 | 26.8 | 26.6 | 26.97 | 26.97 | 26.87 | 26.91 | 27.05 | 27.05 |
| H 1'a | 1.92 | 30.15 | 30.22 | 30.08 | 29.98 | 30.1 | 30.14 | 30.05 | 29.62 |
| H 1'b | 1.86 | 29.55 | 30.02 | 29.81 | 29.81 | 29.95 | 30.02 | 29.86 | 29.78 |
| H 2' | 4.14 | 27.37 | 27.11 | 27.42 | 28.08 | 27.91 | 27.33 | 27.1 | 27.36 |
| H 3'a | 1.95 | 30.25 | 30.4 | 30.43 | 29.93 | 30.71 | 30.3 | 30.22 | 30.15 |
| H 3'b | 1.31 | 30.37 | 30.22 | 30.15 | 30.44 | 29.9 | 30.49 | 30.19 | 30.26 |
| H 4' | 4.04 | 27.67 | 27.8 | 27.55 | 27.99 | 27.89 | 27.55 | 27.68 | 27.67 |
| H 5'a | 2.16 | 29.91 | 29.92 | 30.24 | 30.58 | 30.05 | 29.99 | 29.84 | 30.14 |
| H 5'b | 1.76 | 30.04 | 29.85 | 30.07 | 29.89 | 30.53 | 30.26 | 30.12 | 29.93 |
| H 6' | 4.8 | 26.95 | 27.01 | 27.21 | 27.89 | 27.83 | 27.17 | 27.22 | 26.94 |
| H 7' | 6.22 | 25.89 | 25.62 | 25.75 | 25.64 | 25.69 | 25.75 | 25.26 | 25.77 |
| H 8' | 6.6 | 24.76 | 24.72 | 24.9 | 25.01 | 24.95 | 24.89 | 24.83 | 24.8 |
| H 2''-6'' | 7.42 | 24.285 | 24.15 | 24.505 | 24.49 | 24.505 | 24.515 | 24.415 | 24.465 |
| H 3''-5'' | 7.32 | 24.39 | 24.355 | 24.415 | 24.405 | 24.405 | 24.42 | 24.385 | 24.405 |
| H 4'' | 7.23 | 24.55 | 24.54 | 24.54 | 24.53 | 24.53 | 24.54 | 24.52 | 24.54 |
| J 3,4 | 9.7 | 10.14 | 10.1 | 10.07 | 10.08 | 10.1 | 10.09 | 10.08 | 10.1 |
| J 2',3'b | 10.6 | 8.16 | 11.02 | 1.62 | 1.52 | 10.35 | 10.42 | 2.28 | 3.34 |
| J 4',3'b | 10.6 | 8.3 | 3.06 | 2.92 | 4.24 | 9.97 | 2.46 | 3.91 | 3.53 |

[8] Compound **10** and **11**: NMR data from Della-Felice, F.; Sarotti, A. M.; Pilli, R. A. *J. Org. Chem.* **2017**, *82*, 9191

| | | | | | | | | | |
|---|---|---|---|---|---|---|---|---|---|
| J 4',5'a | 4.2 | 3.85 | 2.7 | 2.86 | 9.9 | 4.41 | 2.79 | 3.43 | 7.99 |
| J 4',5'b | 10.6 | 8.83 | 3.38 | 2.83 | 4.36 | 9.9 | 2.96 | 3.51 | 3.68 |
| J 6',5'a | 2 | 3.95 | 0.98 | 11.1 | 10.82 | 1.73 | 1.83 | 6.7 | 4.9 |
| J 6',5'b | 5.8 | 5.36 | 7.63 | 1.84 | 1.74 | 10.81 | 11.09 | 2.11 | 4.81 |
| J 6',7' | 4.2 | 3.47 | 2.99 | 5.59 | 6.17 | 6.31 | 5.67 | 5.59 | 4.42 |
| J 7',8' | 16.4 | 15.39 | 15.75 | 15.06 | 15.05 | 15.03 | 15.06 | 15.31 | 15.26 |

**Table S11.** Boltzmann averaged GIAO isotropic magnetic shielding constants (σ) of isomers 1-8 calculated at the B3LYP/6-31G** level of theory for compound **11**

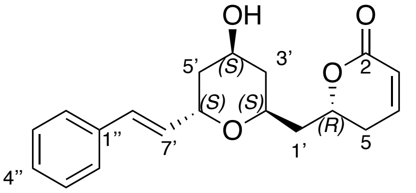


**11**

| | | Isotropic shielding constants | | | | | | | |
|---|---|---|---|---|---|---|---|---|---|
| **Nuclei** | **Experimental** | **Isomer 1** | **Isomer 2** | **Isomer 3** | **Isomer 4** | **Isomer 5** | **Isomer 6** | **Isomer 7** | **Isomer 8** |
| C 2 | 164.7 | 33.71 | 33.28 | 34.11 | 34.25 | 34.48 | 34.5 | 33.95 | 34 |
| C 3 | 121.6 | 72.08 | 71.92 | 72.08 | 72.11 | 71.76 | 71.8 | 72.49 | 72.55 |
| C 4 | 145.4 | 52.45 | 52.38 | 52.87 | 52.78 | 53.57 | 53.57 | 52.1 | 51.94 |
| C 5 | 29.5 | 159.74 | 159.66 | 161.14 | 160.7 | 159.88 | 159.79 | 160.9 | 161.1 |
| C 6 | 75.3 | 117.6 | 118.69 | 116.74 | 117.12 | 117.92 | 117.26 | 116.46 | 116.38 |
| C 1' | 40.6 | 147.44 | 145.34 | 148.83 | 149.02 | 146.45 | 146.5 | 149.45 | 151.11 |
| C 2' | 66.1 | 125.51 | 132.87 | 122.55 | 119.64 | 120.66 | 122.74 | 129.98 | 125.12 |
| C 3' | 41 | 149.4 | 151.72 | 150.74 | 149.14 | 149.62 | 149.25 | 152.29 | 150.8 |
| C 4' | 64.6 | 127.3 | 127.02 | 126.44 | 124.32 | 124.46 | 126.42 | 127.03 | 127.39 |
| C 5' | 38.3 | 153.83 | 154.42 | 153.02 | 149.31 | 148.21 | 153.37 | 153.91 | 152.9 |
| C 6' | 72.7 | 120.8 | 120.87 | 117.88 | 114.99 | 115.46 | 118.04 | 120.04 | 121.17 |
| C 7' | 129.1 | 63.63 | 67.05 | 60.91 | 62.29 | 61.5 | 60.63 | 63.26 | 62.81 |
| C 8' | 132.4 | 62.44 | 63.99 | 63.7 | 63.28 | 63.01 | 63.65 | 62.74 | 62.54 |
| C 1'' | 136.7 | 58.8 | 61.13 | 58.37 | 58.98 | 58.48 | 58.21 | 59.33 | 58.74 |
| C 2''-6'' | 126.7 | 69.69 | 69.275 | 70.095 | 70.06 | 70.025 | 70.07 | 69.845 | 70 |
| C 3''-5'' | 128.9 | 68.465 | 68.25 | 68.39 | 68.29 | 68.36 | 68.425 | 68.22 | 68.345 |
| C 4'' | 20 | 70.72 | 70.13 | 70.72 | 70.43 | 70.54 | 70.79 | 70.28 | 70.57 |
| H 3 | 6.03 | 25.76 | 25.73 | 25.85 | 25.84 | 25.85 | 25.85 | 25.83 | 25.8 |
| H 4 | 6.88 | 24.98 | 24.96 | 25.03 | 25.04 | 25.09 | 25.09 | 25.02 | 24.98 |
| H 5a | 2.39 | 29.75 | 29.89 | 29.72 | 29.85 | 29.85 | 29.76 | 29.8 | 29.86 |
| H 5b | 2.46 | 29.57 | 29.54 | 29.28 | 29.25 | 29.57 | 29.55 | 28.86 | 28.87 |
| H 6 | 4.67 | 26.8 | 26.6 | 26.97 | 26.97 | 26.87 | 26.91 | 27.05 | 27.05 |
| H 1'a | 1.84 | 30.15 | 30.22 | 30.08 | 29.98 | 30.1 | 30.14 | 30.05 | 29.62 |
| H 1'b | 2.02 | 29.55 | 30.02 | 29.81 | 29.81 | 29.95 | 30.02 | 29.86 | 29.78 |
| H 2' | 3.97 | 27.37 | 27.11 | 27.42 | 28.08 | 27.91 | 27.33 | 27.1 | 27.36 |
| H 3'a | 2.01 | 30.25 | 30.4 | 30.43 | 29.93 | 30.71 | 30.3 | 30.22 | 30.15 |
| H 3'b | 1.37 | 30.37 | 30.22 | 30.15 | 30.44 | 29.9 | 30.49 | 30.19 | 30.26 |
| H 4' | 4.07 | 27.67 | 27.8 | 27.55 | 27.99 | 27.89 | 27.55 | 27.68 | 27.67 |
| H 5'a | 1.77 | 29.91 | 29.92 | 30.24 | 30.58 | 30.05 | 29.99 | 29.84 | 30.14 |
| H 5'b | 2.16 | 30.04 | 29.85 | 30.07 | 29.89 | 30.53 | 30.26 | 30.12 | 29.93 |
| H 6' | 4.76 | 26.95 | 27.01 | 27.21 | 27.89 | 27.83 | 27.17 | 27.22 | 26.94 |
| H 7' | 6.28 | 25.89 | 25.62 | 25.75 | 25.64 | 25.69 | 25.75 | 25.26 | 25.77 |
| H 8' | 6.03 | 24.76 | 24.72 | 24.9 | 25.01 | 24.95 | 24.89 | 24.83 | 24.8 |
| H 2''-6'' | 6.88 | 24.285 | 24.15 | 24.505 | 24.49 | 24.505 | 24.515 | 24.415 | 24.465 |
| H 3''-5'' | 2.39 | 24.39 | 24.355 | 24.415 | 24.405 | 24.405 | 24.42 | 24.385 | 24.405 |
| H 4'' | 2.46 | 24.55 | 24.54 | 24.54 | 24.53 | 24.53 | 24.54 | 24.52 | 24.54 |
| J 3,4 | 9.6 | 10.14 | 10.1 | 10.07 | 10.08 | 10.1 | 10.09 | 10.08 | 10.1 |
| J 4,5a | 2.7 | 3.37 | 2.89 | 2.99 | 2.92 | 3.07 | 3.06 | 2.91 | 2.88 |
| J 4,5b | 5.5 | 6.05 | 6.51 | 6.44 | 6.5 | 6.35 | 6.37 | 6.5 | 6.5 |

| | | | | | | | | | |
|---|---|---|---|---|---|---|---|---|---|
| J 5a,6 | 5 | 3.3 | 2.74 | 2.78 | 2.68 | 2.99 | 3.03 | 2.75 | 2.7 |
| J 5b,6 | 11 | 10 | 11.5 | 11.45 | 11.66 | 10.93 | 10.93 | 11.98 | 12.14 |
| J 6,1'a | 6.3 | 1.06 | 1.27 | 4.97 | 3.68 | 1.85 | 2.61 | 5.71 | 4.49 |
| J 6,1'b | 6.1 | 9.7 | 9.86 | 5.52 | 5.92 | 9.19 | 8.21 | 3.56 | 3.81 |
| J 1'a,2' | 5.3 | 8.25 | 9.54 | 3.21 | 3.4 | 9.08 | 8.84 | 2.42 | 4.47 |
| J 1'b,2' | 7.7 | 3.75 | 1.52 | 7.5 | 6.01 | 1.8 | 2.02 | 8.66 | 7.33 |
| J 2',3'a | 2.2 | 8.16 | 11.02 | 1.62 | 1.52 | 10.35 | 10.42 | 2.28 | 3.34 |
| J 2',3'b | 10.3 | 2.91 | 1.94 | 10.55 | 10.54 | 1.57 | 1.67 | 9.69 | 7.25 |
| J 4',3'a | 4.2 | 8.3 | 3.06 | 2.92 | 4.24 | 9.97 | 2.46 | 3.91 | 3.53 |
| J 4',3'b | 10.3 | 3.61 | 2.95 | 2.52 | 9.82 | 4.34 | 2.88 | 3.09 | 7.55 |
| J 4',5'a | 10.3 | 3.85 | 2.7 | 2.86 | 9.9 | 4.41 | 2.79 | 3.43 | 7.99 |
| J 4',5'b | 4.2 | 8.83 | 3.38 | 2.83 | 4.36 | 9.9 | 2.96 | 3.51 | 3.68 |
| J 6',5'a | 5.7 | 3.95 | 0.98 | 11.1 | 10.82 | 1.73 | 1.83 | 6.7 | 4.9 |
| J 6',5'b | 2 | 5.36 | 7.63 | 1.84 | 1.74 | 10.81 | 11.09 | 2.11 | 4.81 |
| J 6',7' | 5 | 3.47 | 2.99 | 5.59 | 6.17 | 6.31 | 5.67 | 5.59 | 4.42 |
| J 7',8' | 16.2 | 15.39 | 15.75 | 15.06 | 15.05 | 15.03 | 15.06 | 15.31 | 15.26 |

**Table S12.** Boltzmann averaged GIAO isotropic magnetic shielding constants (σ) of isomers 1-16 calculated at the B3LYP/6-31G** level of theory for compound **12**[9]

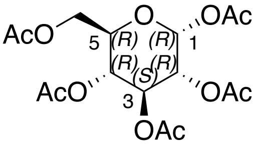

**12**

| Isotropic shielding constants | | | | | | | | | |
|---|---|---|---|---|---|---|---|---|---|
| **Nuclei** | **Experimental** | **Isomer 1** | **Isomer 2** | **Isomer 3** | **Isomer 4** | **Isomer 5** | **Isomer 6** | **Isomer 7** | **Isomer 8** |
| C 1 | 89.1 | 96.2 | 96.56 | 96.45 | 95.41 | 96.45 | 95.46 | 95.06 | 95.3 |
| C 2 | 69.2 | 119.3 | 118.51 | 118.86 | 119.35 | 117.28 | 117.6 | 118.43 | 118.06 |
| C 3 | 69.8 | 118.79 | 118.66 | 120.78 | 121.91 | 117.84 | 114.52 | 117.31 | 116.27 |
| C 4 | 67.9 | 122.22 | 122.44 | 122.81 | 119.44 | 121.29 | 122.1 | 123.62 | 123.83 |
| C 5 | 69.8 | 121.01 | 116.79 | 121.2 | 114.49 | 118.61 | 115.98 | 118.38 | 115.46 |
| C 6 | 61.5 | 128.04 | 128.14 | 127.75 | 127.98 | 128.66 | 128.61 | 128.16 | 128.16 |
| H 5 | 4.14 | 27.31 | 27.53 | 27.43 | 27.45 | 27.52 | 28.03 | 27.66 | 27.96 |
| H 1 | 6.35 | 25.17 | 25.37 | 25.45 | 25.17 | 25.1 | 25.84 | 25.46 | 25.84 |
| H 2 | 5.12 | 26.74 | 26.86 | 26.77 | 26.31 | 26.67 | 26.57 | 26.37 | 26.19 |
| H 3 | 5.5 | 25.98 | 25.76 | 26.11 | 26.17 | 26.26 | 26.52 | 26.52 | 26.84 |
| H 4 | 5.15 | 26.84 | 26.86 | 26.52 | 26.18 | 26.52 | 26.58 | 26.13 | 26.26 |
| H 6 | 4.15 | 27.78 | 27.73 | 27.8 | 27.48 | 27.79 | 27.82 | 27.86 | 27.56 |
| J 1,2 | 3.3 | 2.67 | 7.87 | 1.82 | 2.58 | 2.67 | 7.89 | 1.86 | 0.99 |
| J 2,3 | 10.3 | 2.48 | 2.54 | 4.04 | 8.62 | 9.28 | 8.47 | 2.78 | 2.46 |
| J 3,4 | 9.8 | 2.38 | 2.33 | 2.64 | 2.54 | 8.21 | 8.15 | 8.89 | 8.12 |
| J 4,5 | 9.8 | 9.73 | 9.48 | 8.83 | 2.99 | 9.81 | 9.68 | 9.76 | 8.51 |
| J 5,6a | 2.2 | 1.51 | 1.52 | 1.79 | 7.13 | 1.37 | 1.35 | 1.44 | 2.24 |
| J 5,6b | 4.1 | 3.41 | 3.91 | 3.5 | 5.57 | 3.46 | 4.25 | 3.79 | 4.3 |

| Isotropic shielding constants | | | | | | | | | |
|---|---|---|---|---|---|---|---|---|---|
| **Nuclei** | **Experimental** | **Isomer 9** | **Isomer 10** | **Isomer 11** | **Isomer 12** | **Isomer 13** | **Isomer 14** | **Isomer 15** | **Isomer 16** |
| C 1 | 89.1 | 96.85 | 96.88 | 97.71 | 97.36 | 95.57 | 95.23 | 95.9 | 94.53 |
| C 2 | 69.2 | 119.83 | 119.26 | 117.81 | 117.22 | 119.44 | 119.49 | 119.06 | 119.31 |
| C 3 | 69.8 | 120.47 | 120.37 | 117.66 | 121.52 | 119.52 | 115.82 | 119.93 | 118.54 |
| C 4 | 67.9 | 120.47 | 120.9 | 119.23 | 118.66 | 120.43 | 121.9 | 122.47 | 123.43 |
| C 5 | 69.8 | 120.97 | 116.12 | 119.61 | 119.35 | 119.66 | 116.72 | 120.31 | 116.58 |
| C 6 | 61.5 | 128.29 | 128.61 | 128.15 | 127.48 | 129.92 | 130.93 | 129.68 | 130.32 |
| H 5 | 4.14 | 26.95 | 27.23 | 27.11 | 27.03 | 27.12 | 27.77 | 27.28 | 27.62 |
| H 1 | 6.35 | 25.22 | 25.46 | 25.16 | 25.17 | 25 | 25.73 | 25.2 | 25.81 |
| H 2 | 5.12 | 26.37 | 26.57 | 26.61 | 26.75 | 26.29 | 26.24 | 26.8 | 26.37 |
| H 3 | 5.5 | 26.39 | 26.06 | 25.97 | 25.93 | 26.42 | 26.81 | 26.57 | 26.82 |
| H 4 | 5.15 | 26.67 | 26.89 | 26.77 | 26.63 | 26.13 | 26.34 | 26.34 | 26.49 |
| H 6 | 4.15 | 27.5 | 27.21 | 27.65 | 27.49 | 27.22 | 26.93 | 27.21 | 26.88 |
| J 1,2 | 3.3 | 2.79 | 7.82 | 7.14 | 2.98 | 2.72 | 7.77 | 1.6 | 1.21 |
| J 2,3 | 10.3 | 2.72 | 2.66 | 8.2 | 9.16 | 9.76 | 9.23 | 3.04 | 2.49 |
| J 3,4 | 9.8 | 4.21 | 4 | 8.24 | 9 | 2.55 | 2.65 | 2.35 | 2.75 |
| J 4,5 | 9.8 | 1.68 | 1.32 | 6.34 | 6.77 | 1 | 0.79 | 1.12 | 2.01 |
| J 5,6a | 2.2 | 4.77 | 5.23 | 1.86 | 3.65 | 7.61 | 7.76 | 7.43 | 7.28 |
| J 5,6b | 4.1 | 6.8 | 7.1 | 3.76 | 7.33 | 6.14 | 6.04 | 6.25 | 6.49 |

[9] Compound **12**, **13** and **14**: NMR data measured in our lab

**Table S13.** Boltzmann averaged GIAO isotropic magnetic shielding constants (σ) of isomers 1-16 calculated at the B3LYP/6-31G** level of theory for compound **13**

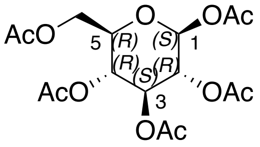

**13**

| | | | | Isotropic shielding constants | | | | | |
|---|---|---|---|---|---|---|---|---|---|
| **Nuclei** | **Experimental** | **Isomer 1** | **Isomer 2** | **Isomer 3** | **Isomer 4** | **Isomer 5** | **Isomer 6** | **Isomer 7** | **Isomer 8** |
| C 1 | 91.7 | 96.2 | 96.56 | 96.45 | 95.41 | 96.45 | 95.46 | 95.06 | 95.3 |
| C 2 | 70.2 | 119.3 | 118.51 | 118.86 | 119.35 | 117.28 | 117.6 | 118.43 | 118.06 |
| C 3 | 72.8 | 118.79 | 118.66 | 120.78 | 121.91 | 117.84 | 114.52 | 117.31 | 116.27 |
| C 4 | 67.7 | 122.22 | 122.44 | 122.81 | 119.44 | 121.29 | 122.1 | 123.62 | 123.83 |
| C 5 | 72.7 | 121.01 | 116.79 | 121.2 | 114.49 | 118.61 | 115.98 | 118.38 | 115.46 |
| C 6 | 61.4 | 128.04 | 128.14 | 127.75 | 127.98 | 128.66 | 128.61 | 128.16 | 128.16 |
| H 1 | 5.72 | 25.17 | 25.37 | 25.45 | 25.17 | 25.1 | 25.84 | 25.46 | 25.84 |
| H 2 | 5.16 | 26.74 | 26.86 | 26.77 | 26.31 | 26.67 | 26.57 | 26.37 | 26.19 |
| H 3 | 5.26 | 25.98 | 25.76 | 26.11 | 26.17 | 26.26 | 26.52 | 26.52 | 26.84 |
| H 4 | 5.15 | 26.84 | 26.86 | 26.52 | 26.18 | 26.52 | 26.58 | 26.13 | 26.26 |
| H 5 | 3.85 | 27.31 | 27.53 | 27.43 | 27.45 | 27.52 | 28.03 | 27.66 | 27.96 |
| H 6 | 4.21 | 27.78 | 27.73 | 27.8 | 27.48 | 27.79 | 27.82 | 27.86 | 27.56 |
| J 1,2 | 8.4 | 2.67 | 7.87 | 1.82 | 2.58 | 2.67 | 7.89 | 1.86 | 0.99 |
| J 2,3 | 9.5 | 2.48 | 2.54 | 4.04 | 8.62 | 9.28 | 8.47 | 2.78 | 2.46 |
| J 3,4 | 9.5 | 2.38 | 2.33 | 2.64 | 2.54 | 8.21 | 8.15 | 8.89 | 8.12 |
| J 4,5 | 10 | 9.73 | 9.48 | 8.83 | 2.99 | 9.81 | 9.68 | 9.76 | 8.51 |
| J 5,6a | 2 | 1.51 | 1.52 | 1.79 | 7.13 | 1.37 | 1.35 | 1.44 | 2.24 |
| J 5,6b | 4.4 | 3.41 | 3.91 | 3.5 | 5.57 | 3.46 | 4.25 | 3.79 | 4.3 |

| | | | | Isotropic shielding constants | | | | | |
|---|---|---|---|---|---|---|---|---|---|
| **Nuclei** | **Experimental** | **Isomer 9** | **Isomer 10** | **Isomer 11** | **Isomer 12** | **Isomer 13** | **Isomer 14** | **Isomer 15** | **Isomer 16** |
| C 1 | 91.7 | 96.85 | 96.88 | 97.71 | 97.36 | 95.57 | 95.23 | 95.9 | 94.53 |
| C 2 | 70.2 | 119.83 | 119.26 | 117.81 | 117.22 | 119.44 | 119.49 | 119.06 | 119.31 |
| C 3 | 72.8 | 120.47 | 120.37 | 117.66 | 121.52 | 119.52 | 115.82 | 119.93 | 118.54 |
| C 4 | 67.7 | 120.47 | 120.9 | 119.23 | 118.66 | 120.43 | 121.9 | 122.47 | 123.43 |
| C 5 | 72.7 | 120.97 | 116.12 | 119.61 | 119.35 | 119.66 | 116.72 | 120.31 | 116.58 |
| C 6 | 61.4 | 128.29 | 128.61 | 128.15 | 127.48 | 129.92 | 130.93 | 129.68 | 130.32 |
| H 1 | 5.72 | 25.22 | 25.46 | 25.16 | 25.17 | 25 | 25.73 | 25.2 | 25.81 |
| H 2 | 5.16 | 26.37 | 26.57 | 26.61 | 26.75 | 26.29 | 26.24 | 26.8 | 26.37 |
| H 3 | 5.26 | 26.39 | 26.06 | 25.97 | 25.93 | 26.42 | 26.81 | 26.57 | 26.82 |
| H 4 | 5.15 | 26.67 | 26.89 | 26.77 | 26.63 | 26.13 | 26.34 | 26.34 | 26.49 |
| H 5 | 3.85 | 26.95 | 27.23 | 27.11 | 27.03 | 27.12 | 27.77 | 27.28 | 27.62 |
| H 6 | 4.21 | 27.5 | 27.21 | 27.65 | 27.49 | 27.22 | 26.93 | 27.21 | 26.88 |
| J 1,2 | 8.4 | 2.79 | 7.82 | 7.14 | 2.98 | 2.72 | 7.77 | 1.6 | 1.21 |
| J 2,3 | 9.5 | 2.72 | 2.66 | 8.2 | 9.16 | 9.76 | 9.23 | 3.04 | 2.49 |
| J 3,4 | 9.5 | 4.21 | 4 | 8.24 | 9 | 2.55 | 2.65 | 2.35 | 2.75 |
| J 4,5 | 10 | 1.68 | 1.32 | 6.34 | 6.77 | 1 | 0.79 | 1.12 | 2.01 |
| J 5,6a | 2 | 4.77 | 5.23 | 1.86 | 3.65 | 7.61 | 7.76 | 7.43 | 7.28 |
| J 5,6b | 4.4 | 6.8 | 7.1 | 3.76 | 7.33 | 6.14 | 6.04 | 6.25 | 6.49 |

**Table S14.** Boltzmann averaged GIAO isotropic magnetic shielding constants (σ) of isomers 1-16 calculated at the B3LYP/6-31G** level of theory for compound **14**

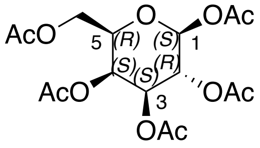


**14**

| Nuclei | Experimental | Isomer 1 | Isomer 2 | Isomer 3 | Isomer 4 | Isomer 5 | Isomer 6 | Isomer 7 | Isomer 8 |
|---|---|---|---|---|---|---|---|---|---|
| | | | | Isotropic shielding constants | | | | | |
| C 1 | 92.2 | 96.2 | 96.56 | 96.45 | 95.41 | 96.45 | 95.46 | 95.06 | 95.3 |
| C 2 | 67.8 | 119.3 | 118.51 | 118.86 | 119.35 | 117.28 | 117.6 | 118.43 | 118.06 |
| C 3 | 70.8 | 118.79 | 118.66 | 120.78 | 121.91 | 117.84 | 114.52 | 117.31 | 116.27 |
| C 4 | 66.8 | 122.22 | 122.44 | 122.81 | 119.44 | 121.29 | 122.1 | 123.62 | 123.83 |
| C 5 | 71.7 | 121.01 | 116.79 | 121.2 | 114.49 | 118.61 | 115.98 | 118.38 | 115.46 |
| C 6 | 61 | 128.04 | 128.14 | 127.75 | 127.98 | 128.66 | 128.61 | 128.16 | 128.16 |
| H 1 | 5.72 | 25.17 | 25.37 | 25.45 | 25.17 | 25.1 | 25.84 | 25.46 | 25.84 |
| H 2 | 5.35 | 26.74 | 26.86 | 26.77 | 26.31 | 26.67 | 26.57 | 26.37 | 26.19 |
| H 3 | 5.1 | 25.98 | 25.76 | 26.11 | 26.17 | 26.26 | 26.52 | 26.52 | 26.84 |
| H 4 | 5.45 | 26.84 | 26.86 | 26.52 | 26.18 | 26.52 | 26.58 | 26.13 | 26.26 |
| H 5 | 4.07 | 27.31 | 27.53 | 27.43 | 27.45 | 27.52 | 28.03 | 27.66 | 27.96 |
| H 6 | 4.16 | 26.98 | 27.08 | 27.02 | 27.13 | 26.96 | 26.9 | 26.91 | 27.06 |
| J 1,2 | 8 | 2.67 | 7.87 | 1.82 | 2.58 | 2.67 | 7.89 | 1.86 | 0.99 |
| J 2,3 | 9.7 | 2.48 | 2.54 | 4.04 | 8.62 | 9.28 | 8.47 | 2.78 | 2.46 |
| J 3,4 | 3.2 | 2.38 | 2.33 | 2.64 | 2.54 | 8.21 | 8.15 | 8.89 | 8.12 |
| J 4,5 | 0.8 | 9.73 | 9.48 | 8.83 | 2.99 | 9.81 | 9.68 | 9.76 | 8.51 |
| J 5,6a | 6.8 | 1.51 | 1.52 | 1.79 | 7.13 | 1.37 | 1.35 | 1.44 | 2.24 |
| J 5,6b | 6.4 | 3.41 | 3.91 | 3.5 | 5.57 | 3.46 | 4.25 | 3.79 | 4.3 |

| Nuclei | Experimental | Isomer 9 | Isomer 10 | Isomer 11 | Isomer 12 | Isomer 13 | Isomer 14 | Isomer 15 | Isomer 16 |
|---|---|---|---|---|---|---|---|---|---|
| | | | | Isotropic shielding constants | | | | | |
| C 1 | 92.2 | 96.85 | 96.88 | 97.71 | 97.36 | 95.57 | 95.23 | 95.9 | 94.53 |
| C 2 | 67.8 | 119.83 | 119.26 | 117.81 | 117.22 | 119.44 | 119.49 | 119.06 | 119.31 |
| C 3 | 70.8 | 120.47 | 120.37 | 117.66 | 121.52 | 119.52 | 115.82 | 119.93 | 118.54 |
| C 4 | 66.8 | 120.47 | 120.9 | 119.23 | 118.66 | 120.43 | 121.9 | 122.47 | 123.43 |
| C 5 | 71.7 | 120.97 | 116.12 | 119.61 | 119.35 | 119.66 | 116.72 | 120.31 | 116.58 |
| C 6 | 61 | 128.29 | 128.61 | 128.15 | 127.48 | 129.92 | 130.93 | 129.68 | 130.32 |
| H 1 | 5.72 | 25.22 | 25.46 | 25.16 | 25.17 | 25 | 25.73 | 25.2 | 25.81 |
| H 2 | 5.35 | 26.37 | 26.57 | 26.61 | 26.75 | 26.29 | 26.24 | 26.8 | 26.37 |
| H 3 | 5.1 | 26.39 | 26.06 | 25.97 | 25.93 | 26.42 | 26.81 | 26.57 | 26.82 |
| H 4 | 5.45 | 26.67 | 26.89 | 26.77 | 26.63 | 26.13 | 26.34 | 26.34 | 26.49 |
| H 5 | 4.07 | 26.95 | 27.23 | 27.11 | 27.03 | 27.12 | 27.77 | 27.28 | 27.62 |
| H 6 | 4.16 | 27.45 | 27.75 | 26.8 | 26.93 | 27.8 | 27.95 | 27.77 | 27.7 |
| J 1,2 | 8 | 2.79 | 7.82 | 7.14 | 2.98 | 2.72 | 7.77 | 1.6 | 1.21 |
| J 2,3 | 9.7 | 2.72 | 2.66 | 8.2 | 9.16 | 9.76 | 9.23 | 3.04 | 2.49 |
| J 3,4 | 3.2 | 4.21 | 4 | 8.24 | 9 | 2.55 | 2.65 | 2.35 | 2.75 |
| J 4,5 | 0.8 | 1.68 | 1.32 | 6.34 | 6.77 | 1 | 0.79 | 1.12 | 2.01 |
| J 5,6a | 6.8 | 4.77 | 5.23 | 1.86 | 3.65 | 7.61 | 7.76 | 7.43 | 7.28 |
| J 5,6b | 6.4 | 6.8 | 7.1 | 3.76 | 7.33 | 6.14 | 6.04 | 6.25 | 6.49 |

**Table S15.** Boltzmann averaged GIAO isotropic magnetic shielding constants (σ) of isomers 1-8 calculated at the B3LYP/6-31G** level of theory for compound **15**[10]

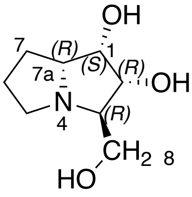


**15**

| Isotropic shielding constants | | | | | | | | | |
|---|---|---|---|---|---|---|---|---|---|
| **Nuclei** | **Experimental** | **Isomer 1** | **Isomer 2** | **Isomer 3** | **Isomer 4** | **Isomer 5** | **Isomer 6** | **Isomer 7** | **Isomer 8** |
| C 1 | 73.7 | 106.99 | 118.93 | 112.64 | 113.83 | 107.16 | 123.95 | 113.54 | 115.49 |
| C 2 | 77.5 | 105.06 | 114.82 | 119.07 | 104.96 | 101.55 | 111.3 | 116.79 | 102.02 |
| C 3 | 71.9 | 115.88 | 118.67 | 120.25 | 121.16 | 127.49 | 127.28 | 128.97 | 121.77 |
| C 8 | 65.2 | 126.09 | 128.47 | 131.55 | 130.02 | 131.7 | 131.28 | 126.84 | 130.85 |
| C 7a | 67.9 | 116.68 | 125.81 | 120.74 | 123.71 | 123.72 | 120.76 | 122.35 | 119.74 |
| C 7 | 26.0 | 159.96 | 166.37 | 159.07 | 166.91 | 163.95 | 168.72 | 159.86 | 168.07 |
| C 6 | 28.8 | 165.66 | 163.09 | 164.04 | 162.18 | 162.95 | 163.57 | 162.99 | 162.07 |
| C 5 | 57.5 | 136.43 | 137.27 | 136.43 | 138.19 | 145.72 | 146.62 | 143.01 | 145.77 |
| H 1 | 3.87 | 28.18 | 27.83 | 28.2 | 27.87 | 28.02 | 28.18 | 27.96 | 28.38 |
| H 2 | 3.88 | 27.62 | 27.55 | 27.7 | 27.7 | 27.63 | 27.46 | 27.65 | 27.16 |
| H 3 | 2.76 | 28.97 | 28.97 | 29.2 | 29.06 | 29.53 | 29.78 | 28.46 | 29.55 |
| H 8 | 3.78 | 28.39 | 28.04 | 28.34 | 27.94 | 28.01 | 27.99 | 27.52 | 28.14 |
| H 5α | 3.05 | 28.95 | 29.32 | 29.31 | 29.43 | 28.96 | 28.98 | 29.4 | 29.01 |
| H 5β | 2.65 | 29.03 | 28.99 | 28.87 | 28.89 | 30.02 | 30.14 | 29.33 | 29.79 |
| H 6α | 1.74 | 30.33 | 30.12 | 30.17 | 29.98 | 29.83 | 29.88 | 30.09 | 29.77 |
| H 6β | 1.93 | 29.85 | 29.87 | 30.19 | 29.91 | 29.63 | 29.62 | 30.21 | 29.67 |
| H 7a | 3.47 | 28.3 | 28.22 | 28.32 | 27.61 | 29.49 | 29.65 | 28.04 | 28.76 |
| H 7α | 1.68 | 29.86 | 30.27 | 29.76 | 30.25 | 29.98 | 30.35 | 29.72 | 30.34 |
| H 7β | 2.08 | 30.02 | 29.39 | 30.39 | 29.51 | 30.35 | 29.66 | 30.49 | 29.93 |
| H 8' | 3.58 | 28.22 | 28.34 | 27.79 | 27.91 | 28.04 | 27.97 | 27.73 | 27.94 |
| J 2,3 | 9 | 0.85 | 7.92 | 6.38 | 3.08 | 7 | 6.72 | 8.59 | 3.29 |
| J 1,7a | 4 | 0.63 | 3.79 | 2.08 | 4.69 | 7.59 | 2.87 | 0.84 | 3.53 |
| J 1,2 | 4 | 0.55 | 3.88 | 4.54 | 0.99 | 4.15 | 5.39 | 5.01 | 0.28 |
| J 5α,6α | 8.1 | 3.44 | 6.92 | 7.05 | 7.38 | 1.62 | 1.85 | 10.52 | 3.43 |
| J 5α,6β | 6.1 | 6.15 | 5.48 | 5.42 | 5.4 | 8.63 | 8.65 | 4.72 | 7.85 |
| J 3,8 | 6.4 | 0.46 | 4.83 | 3.86 | 0.55 | 0.87 | 1.62 | 4.43 | 0.85 |
| J 3,8´ | 3.4 | 3.81 | 1.88 | 0.48 | 2.94 | 1.98 | 3.32 | 9.43 | 2.7 |
| J 5β,6α | 6.4 | 5.75 | 5.73 | 5.8 | 5.81 | 8.92 | 9.17 | 5.91 | 8.53 |
| J 5β,6β | 4.9 | 9.34 | 5.5 | 5.35 | 4.99 | 7.17 | 6.89 | 1.43 | 6.09 |
| J 7a,7α | 8.1 | 10.09 | 8.63 | 8.29 | 8.63 | 4.46 | 4.52 | 8.07 | 4.98 |
| J 7a,7β | 6.6 | 5.24 | 5.72 | 6.51 | 6.29 | 9.53 | 10.06 | 8.01 | 10.09 |

[10] Compound **15** and **16**: NMR data from Asano, N. Kuroi, H. Ikeda, K. Kizu, H. Kameda, Y. Kato, A. Adachi, I. Watson, A.A. Nash, R.J. Fleet, G.W. *Tetrahedron Assymmtry*, **2000**, *11*, 1

**Table S16.** Boltzmann averaged GIAO isotropic magnetic shielding constants (σ) of isomers 1-8 calculated at the B3LYP/6-31G** level of theory for compound **16**

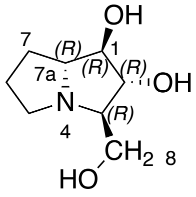


**16**

| Isotropic shielding constants | | | | | | | | | |
|---|---|---|---|---|---|---|---|---|---|
| **Nuclei** | **Experimental** | **Isomer 1** | **Isomer 2** | **Isomer 3** | **Isomer 4** | **Isomer 5** | **Isomer 6** | **Isomer 7** | **Isomer 8** |
| C 1 | 82.9 | 106.99 | 118.93 | 112.64 | 113.83 | 107.16 | 123.95 | 113.54 | 115.49 |
| C 2 | 79.8 | 105.06 | 114.82 | 119.07 | 104.96 | 101.55 | 111.3 | 116.79 | 102.02 |
| C 3 | 72.1 | 115.88 | 118.67 | 120.25 | 121.16 | 127.49 | 127.28 | 128.97 | 121.77 |
| C 8 | 65.3 | 126.09 | 128.47 | 131.55 | 130.02 | 131.7 | 131.28 | 126.84 | 130.85 |
| C 7a | 69.2 | 116.68 | 125.81 | 120.74 | 123.71 | 123.72 | 120.76 | 122.35 | 119.74 |
| C 7 | 32.5 | 159.96 | 166.37 | 159.07 | 166.91 | 163.95 | 168.72 | 159.86 | 168.07 |
| C 6 | 27.3 | 165.66 | 163.09 | 164.04 | 162.18 | 162.95 | 163.57 | 162.99 | 162.07 |
| C 5 | 57.7 | 136.43 | 137.27 | 136.43 | 138.19 | 145.72 | 146.62 | 143.01 | 145.77 |
| H 7a | 3.32 | 28.3 | 28.22 | 28.32 | 27.61 | 29.49 | 29.65 | 28.04 | 28.76 |
| H 1 | 3.76 | 28.18 | 27.83 | 28.2 | 27.87 | 28.02 | 28.18 | 27.96 | 28.38 |
| H 2 | 3.81 | 27.62 | 27.55 | 27.7 | 27.7 | 27.63 | 27.46 | 27.65 | 27.16 |
| H 3 | 2.77 | 28.97 | 28.97 | 29.2 | 29.06 | 29.53 | 29.78 | 28.46 | 29.55 |
| H 8 | 3.67 | 28.39 | 28.04 | 28.34 | 27.94 | 28.01 | 27.99 | 27.52 | 28.14 |
| H 8' | 3.80 | 28.22 | 28.34 | 27.79 | 27.91 | 28.04 | 27.97 | 27.73 | 27.94 |
| H 7α | 1.97 | 29.86 | 30.27 | 29.76 | 30.25 | 29.98 | 30.35 | 29.72 | 30.34 |
| H 7β | 1.97 | 30.02 | 29.39 | 30.39 | 29.51 | 30.35 | 29.66 | 30.49 | 29.93 |
| H 6α | 1.82 | 30.33 | 30.12 | 30.17 | 29.98 | 29.83 | 29.88 | 30.09 | 29.77 |
| H 6β | 1.90 | 29.85 | 29.87 | 30.19 | 29.91 | 29.63 | 29.62 | 30.21 | 29.67 |
| H 5α | 2.96 | 28.95 | 29.32 | 29.31 | 29.43 | 28.96 | 28.98 | 29.4 | 29.01 |
| H 5β | 2.81 | 29.03 | 28.99 | 28.87 | 28.89 | 30.02 | 30.14 | 29.33 | 29.79 |
| J 2,3 | 8.8 | 0.85 | 7.92 | 6.38 | 3.08 | 7 | 6.72 | 8.59 | 3.29 |
| J 1,7a | 7.1 | 0.63 | 3.79 | 2.08 | 4.69 | 7.59 | 2.87 | 0.84 | 3.53 |
| J 1,2 | 8.8 | 0.55 | 3.88 | 4.54 | 0.99 | 4.15 | 5.39 | 5.01 | 0.28 |
| J 5α,6α | 5.6 | 3.44 | 6.92 | 7.05 | 7.38 | 1.62 | 1.85 | 10.52 | 3.43 |
| J 5α,6β | 5.6 | 6.15 | 5.48 | 5.42 | 5.4 | 8.63 | 8.65 | 4.72 | 7.85 |
| J 3,8 | 3.9 | 0.46 | 4.83 | 3.86 | 0.55 | 0.87 | 1.62 | 4.43 | 0.85 |
| J 3,8´ | 6.5 | 3.81 | 1.88 | 0.48 | 2.94 | 1.98 | 3.32 | 9.43 | 2.7 |
| J 5β,6α | 5.9 | 5.75 | 5.73 | 5.8 | 5.81 | 8.92 | 9.17 | 5.91 | 8.53 |
| J 5β,6β | 7.3 | 9.34 | 5.5 | 5.35 | 4.99 | 7.17 | 6.89 | 1.43 | 6.09 |

**Table S17.** Boltzmann averaged GIAO isotropic magnetic shielding constants (σ) of isomers 1-8 calculated at the B3LYP/6-31G** level of theory for compound **17**[11]

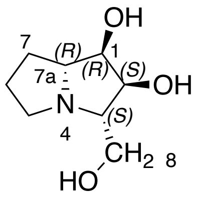


**17**

| Isotropic shielding constants | | | | | | | | | |
|---|---|---|---|---|---|---|---|---|---|
| **Nuclei** | **Experimental** | **Isomer 1** | **Isomer 2** | **Isomer 3** | **Isomer 4** | **Isomer 5** | **Isomer 6** | **Isomer 7** | **Isomer 8** |
| C 1 | 76.9 | 106.99 | 118.93 | 112.64 | 113.83 | 107.16 | 123.95 | 113.54 | 115.49 |
| C 2 | 72.5 | 105.06 | 114.82 | 119.07 | 104.96 | 101.55 | 111.3 | 116.79 | 102.02 |
| C 3 | 67.1 | 115.88 | 118.67 | 120.25 | 121.16 | 127.49 | 127.28 | 128.97 | 121.77 |
| C 8 | 60.9 | 126.09 | 128.47 | 131.55 | 130.02 | 131.7 | 131.28 | 126.84 | 130.85 |
| C 7a | 72.1 | 116.68 | 125.81 | 120.74 | 123.71 | 123.72 | 120.76 | 122.35 | 119.74 |
| C 7 | 30.9 | 159.96 | 166.37 | 159.07 | 166.91 | 163.95 | 168.72 | 159.86 | 168.07 |
| C 6 | 27.4 | 165.66 | 163.09 | 164.04 | 162.18 | 162.95 | 163.57 | 162.99 | 162.07 |
| C 5 | 49.1 | 136.43 | 137.27 | 136.43 | 138.19 | 145.72 | 146.62 | 143.01 | 145.77 |
| H 7a | 3.39 | 28.3 | 28.22 | 28.32 | 27.61 | 29.49 | 29.65 | 28.04 | 28.76 |
| H 1 | 3.76 | 28.18 | 27.83 | 28.2 | 27.87 | 28.02 | 28.18 | 27.96 | 28.38 |
| H 2 | 3.88 | 27.62 | 27.55 | 27.7 | 27.7 | 27.63 | 27.46 | 27.65 | 27.16 |
| H 3 | 3.21 | 28.97 | 28.97 | 29.2 | 29.06 | 29.53 | 29.78 | 28.46 | 29.55 |
| H 8 | 3.84 | 28.39 | 28.04 | 28.34 | 27.94 | 28.01 | 27.99 | 27.52 | 28.14 |
| H 8' | 3.81 | 28.22 | 28.34 | 27.79 | 27.91 | 28.04 | 27.97 | 27.73 | 27.94 |
| H 7α | 2.14 | 29.86 | 30.27 | 29.76 | 30.25 | 29.98 | 30.35 | 29.72 | 30.34 |
| H 7β | 1.50 | 30.02 | 29.39 | 30.39 | 29.51 | 30.35 | 29.66 | 30.49 | 29.93 |
| H 6α | 1.87 | 30.33 | 30.12 | 30.17 | 29.98 | 29.83 | 29.88 | 30.09 | 29.77 |
| H 6β | 1.69 | 29.85 | 29.87 | 30.19 | 29.91 | 29.63 | 29.62 | 30.21 | 29.67 |
| H 5α | 2.91 | 28.95 | 29.32 | 29.31 | 29.43 | 28.96 | 28.98 | 29.4 | 29.01 |
| H 5β | 2.79 | 29.03 | 28.99 | 28.87 | 28.89 | 30.02 | 30.14 | 29.33 | 29.79 |
| J 2,3 | 9 | 0.85 | 7.92 | 6.38 | 3.08 | 7 | 6.72 | 8.59 | 3.29 |
| J 1,7a | 2.4 | 0.63 | 3.79 | 2.08 | 4.69 | 7.59 | 2.87 | 0.84 | 3.53 |
| J 1,2 | 5.5 | 0.55 | 3.88 | 4.54 | 0.99 | 4.15 | 5.39 | 5.01 | 0.28 |
| J 3,8 | 4.1 | 0.46 | 4.83 | 3.86 | 0.55 | 0.87 | 1.62 | 4.43 | 0.85 |
| J 3,8´ | 8 | 3.81 | 1.88 | 0.48 | 2.94 | 1.98 | 3.32 | 9.43 | 2.7 |
| J 7a,7α | 7.9 | 10.09 | 8.63 | 8.29 | 8.63 | 4.46 | 4.52 | 8.07 | 4.98 |
| J 7a,7β | 7.9 | 5.24 | 5.72 | 6.51 | 6.29 | 9.53 | 10.06 | 8.01 | 10.09 |

[11] Compound **17**: NMR data from Izquierdo, I. Plaza, M.T. Tamayo, J.A. Franco, F. Sánchez-Cantalejo, F. *Tetrahedron*, **2010**, *66*, 3788

**Table S18.** Boltzmann averaged GIAO isotropic magnetic shielding constants (σ) of isomers 1-8 calculated at the B3LYP/6-31G** level of theory for compound **18**[12]

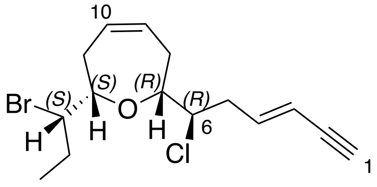


**18**

| | | Isotropic shielding constants | | | | | | | |
|---|---|---|---|---|---|---|---|---|---|
| **Nuclei** | **Experimental** | **Isomer 1** | **Isomer 2** | **Isomer 3** | **Isomer 4** | **Isomer 5** | **Isomer 6** | **Isomer 7** | **Isomer 8** |
| C 1 | 77 | 118.05 | 118.43 | 118.31 | 118.63 | 118.14 | 118.55 | 118.28 | 118.59 |
| C 2 | 82 | 117.52 | 117.56 | 117.57 | 117.4 | 117.53 | 117.53 | 117.62 | 117.42 |
| C 3 | 111.8 | 81.78 | 83.16 | 83.13 | 82.53 | 82.24 | 83.21 | 83.39 | 82.3 |
| C 4 | 142 | 53 | 51.31 | 51.51 | 52.01 | 52.55 | 51.22 | 51.39 | 52.44 |
| C 5 | 37.6 | 149.85 | 151.32 | 150.89 | 151.15 | 150.09 | 151.02 | 151.16 | 151.05 |
| C 6 | 63 | 121.93 | 122.87 | 122.87 | 124.02 | 122.13 | 122.77 | 123.3 | 123.9 |
| C 7 | 81.2 | 113.51 | 106.49 | 108.46 | 111.09 | 113.25 | 106.69 | 107.6 | 111 |
| C 8 | 32.7 | 157.16 | 156.97 | 157.56 | 159.49 | 157.75 | 156.81 | 158.32 | 159.53 |
| C 10 | 82.5 | 111.07 | 106.17 | 105.31 | 110.78 | 109.97 | 105.31 | 105.8 | 110.84 |
| C 13 | 28 | 158.04 | 160.1 | 160.98 | 160.79 | 159.69 | 160.37 | 160.39 | 159.34 |
| C 14 | 12.7 | 177.28 | 177.19 | 177.55 | 177.48 | 177.74 | 177.36 | 177.3 | 177.49 |
| C 15 | 33.8 | 156.71 | 157.56 | 155.98 | 156.92 | 159.99 | 156.64 | 158.11 | 156.87 |
| C 16 | 129.2 | 64.99 | 62.59 | 63.27 | 65.19 | 65.09 | 63.13 | 62.63 | 64.82 |
| C 17 | 129 | 65.42 | 63.82 | 62.44 | 66.27 | 64.92 | 63.33 | 62.74 | 66.13 |
| H 1 | 2.83 | 29.24 | 29.25 | 29.26 | 29.26 | 29.25 | 29.26 | 29.25 | 29.26 |
| H 3 | 5.59 | 25.83 | 26.14 | 26.07 | 26.01 | 25.92 | 26.13 | 26.15 | 25.99 |
| H 4 | 6.27 | 25.35 | 25.09 | 25.21 | 25.02 | 25.32 | 25.07 | 25.18 | 25.01 |
| H 5a | 2.56 | 29.37 | 29.29 | 29.43 | 29.24 | 29.4 | 29.26 | 29.46 | 29.21 |
| H 5b | 2.78 | 28.84 | 29.11 | 28.96 | 29.04 | 28.9 | 29.04 | 29.1 | 29.12 |
| H 7 | 3.52 | 27.21 | 28.27 | 28.21 | 27.53 | 27.23 | 28.29 | 28.25 | 27.5 |
| H 8a | 2.3 | 29.9 | 29.27 | 29.64 | 29.22 | 29.86 | 29.3 | 29.58 | 29.28 |
| H 8b | 2.55 | 28.73 | 29.41 | 28.98 | 29.26 | 28.76 | 29.35 | 29.06 | 29.24 |
| H 9 | 5.8 | 25.86 | 25.63 | 25.57 | 25.81 | 25.83 | 25.61 | 25.57 | 25.83 |
| H 10 | 5.83 | 25.84 | 25.58 | 25.6 | 25.87 | 25.83 | 25.62 | 25.56 | 25.84 |
| H 11a | 2.55 | 29.84 | 29.47 | 29.11 | 29.04 | 29.59 | 29.61 | 29.11 | 28.92 |
| H 11b | 2.36 | 28.71 | 29.11 | 29.59 | 29.66 | 28.92 | 29.06 | 29.46 | 29.66 |
| H 12 | 3.59 | 27.23 | 28.07 | 28.15 | 27.23 | 27.31 | 28.1 | 28.2 | 27.24 |
| H 14a | 1.85 | 29.66 | 29.73 | 29.73 | 29.83 | 29.99 | 29.93 | 30.04 | 29.92 |
| H 14b | 1.98 | 29.69 | 29.98 | 29.82 | 29.9 | 29.92 | 29.8 | 29.74 | 29.8 |
| H 15 | 0.94 | 30.69 | 30.69 | 30.69 | 30.67 | 30.63 | 30.68 | 30.69 | 30.7 |
| J 6,7 | 4 | 1.47 | 5.99 | 2.2 | 8.01 | 1.61 | 6.33 | 2.61 | 8.22 |
| J 7,8a | 2 | 0.39 | 8.42 | 0.44 | 9.34 | 0.39 | 8.62 | 0.43 | 9.35 |
| J 7,8b | 10 | 9.15 | 0.74 | 9.1 | 1.65 | 9.21 | 0.66 | 9.11 | 1.07 |
| J 8a,9 | 4 | 7.74 | 4.39 | 8.88 | 3.57 | 7.79 | 4.23 | 8.92 | 3.08 |
| J 8b,9 | 4 | 2.64 | 8.37 | 3.99 | 7.95 | 2.68 | 8.55 | 4.01 | 8.07 |
| J 9,10 | 8 | 10.81 | 10.75 | 10.67 | 10.76 | 10.77 | 10.76 | 10.67 | 10.76 |

[12] Compound **18** and **19**: NMR data from A. Fukuzawa and T. Masamune ,*Tetrahedron Lett.,* **1981**, *22*, 4081

| | | | | | | | | | |
|---|---|---|---|---|---|---|---|---|---|
| J 10,11a | 4 | 7.71 | 8.7 | 4.1 | 3.42 | 7.74 | 8.66 | 4.04 | 3.09 |
| J 10,11b | 4 | 2.65 | 3.82 | 8.87 | 6.86 | 2.82 | 3.95 | 8.9 | 7.08 |
| J 11a,12 | 10 | 0.39 | 0.49 | 8.97 | 9.69 | 0.4 | 0.59 | 9.04 | 9.47 |
| J 11b,12 | 2 | 9 | 9.09 | 0.48 | 0.85 | 8.74 | 8.86 | 0.48 | 0.64 |
| J 12,13 | 4 | 1.47 | 2.56 | 4.02 | 3.12 | 3.98 | 3.57 | 2.94 | 2.89 |

**Table S19.** Boltzmann averaged GIAO isotropic magnetic shielding constants (σ) of isomers 1-8 calculated at the B3LYP/6-31G** level of theory for compound **19**

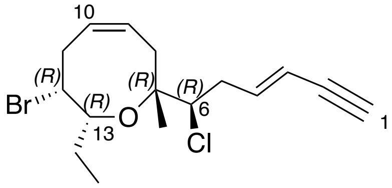


**19**

| | | Isotropic shielding constants | | | | | | | |
|---|---|---|---|---|---|---|---|---|---|
| **Nuclei** | **Experimental** | **Isomer 1** | **Isomer 2** | **Isomer 3** | **Isomer 4** | **Isomer 5** | **Isomer 6** | **Isomer 7** | **Isomer 8** |
| C 1 | 76.8 | 118.33 | 118.34 | 117.84 | 118.03 | 118.24 | 118.13 | 117.92 | 118.35 |
| C 2 | 81.9 | 117.59 | 117.51 | 117.8 | 117.78 | 117.7 | 117.71 | 117.57 | 117.59 |
| C 3 | 111.8 | 83.32 | 82.06 | 82.67 | 83.14 | 83.45 | 82.95 | 81.82 | 83.08 |
| C 4 | 141.9 | 51.19 | 52.9 | 52.27 | 51.61 | 51.3 | 51.8 | 52.8 | 51.16 |
| C 5 | 36.6 | 152.33 | 150.32 | 149.79 | 150.56 | 152.04 | 149.64 | 149.26 | 150.91 |
| C 6 | 63 | 123.9 | 123.47 | 122.39 | 122.19 | 124.2 | 124.75 | 120.92 | 122.51 |
| C 7 | 82.1 | 106 | 113.52 | 114.35 | 103.61 | 104.08 | 113.37 | 116.81 | 105.14 |
| C 8 | 29.4 | 161.12 | 158.12 | 158.22 | 158.68 | 161.41 | 161.15 | 154.88 | 157.22 |
| C 9 | 130.3 | 61.46 | 61.49 | 64.14 | 64.14 | 63.69 | 66.68 | 61.06 | 61.95 |
| C 10 | 129.9 | 64.36 | 68.32 | 66.45 | 63.42 | 63.81 | 63.97 | 69.81 | 64.05 |
| C 11 | 35.5 | 153.51 | 153.64 | 153.33 | 155.99 | 156.19 | 151.12 | 153.14 | 153.56 |
| C 13 | 81.5 | 107.14 | 114.84 | 113.22 | 106.16 | 105.01 | 111.7 | 114.95 | 107.66 |
| C 14 | 28.9 | 160.93 | 160.33 | 160.87 | 163.05 | 162.15 | 162.31 | 160.5 | 161.47 |
| C 15 | 10.3 | 179.54 | 178.95 | 180.47 | 181.25 | 180.15 | 180.49 | 178.66 | 180.11 |
| H 1 | 2.86 | 29.25 | 29.24 | 29.22 | 29.23 | 29.25 | 29.24 | 29.23 | 29.25 |
| H 3 | 5.61 | 26.15 | 26.07 | 26.12 | 26.18 | 26.19 | 26.15 | 25.96 | 26.13 |
| H 4 | 6.29 | 25.12 | 24.95 | 25.21 | 25.17 | 25.17 | 25.12 | 25.19 | 25.12 |
| H 5a | 2.5 | 29.08 | 29.26 | 29.08 | 29.21 | 29.2 | 29.03 | 28.82 | 28.97 |
| H 5b | 2.8 | 29.53 | 29.13 | 29.34 | 29.36 | 29.52 | 29.49 | 29.33 | 29.39 |
| H 6 | 3.97 | 27.83 | 28.04 | 28.04 | 27.97 | 27.81 | 27.9 | 28.13 | 28.08 |
| H 7 | 3.51 | 28.29 | 27.32 | 27.52 | 28.38 | 28.3 | 27.95 | 26.89 | 28.39 |
| H 8a | 2.56 | 29.44 | 29.5 | 29.74 | 29.29 | 29.45 | 29.28 | 29.75 | 29.26 |
| H 8b | 2.32 | 29.23 | 29.12 | 28.98 | 29.62 | 29.37 | 29.42 | 28.86 | 29.47 |
| H 9 | 5.9 | 25.58 | 25.69 | 25.79 | 25.57 | 25.53 | 25.81 | 25.71 | 25.59 |
| H 10 | 5.7 | 25.76 | 25.77 | 25.65 | 25.31 | 25.3 | 25.73 | 25.86 | 25.76 |
| H 11a | 3.12 | 28.72 | 28.78 | 29.09 | 29.33 | 28.84 | 28.95 | 29.13 | 29.22 |
| H 11b | 2.65 | 29.22 | 29.17 | 28.92 | 28.83 | 29.35 | 28.97 | 28.74 | 28.71 |
| H 12 | 4.1 | 27.6 | 27.56 | 27.64 | 27.55 | 27.66 | 27.42 | 27.58 | 27.56 |
| H 13 | 3.49 | 27.94 | 27.52 | 27.6 | 28.17 | 28.22 | 27.71 | 27.41 | 27.97 |
| H 14a | 1.54 | 30.03 | 30.07 | 29.95 | 30.04 | 29.86 | 29.93 | 29.74 | 29.98 |
| H 14b | 1.74 | 30.01 | 29.97 | 29.73 | 29.87 | 30.21 | 29.9 | 30.15 | 29.85 |
| H 15a-c | 0.89 | 30.93 | 30.89 | 30.71 | 30.79 | 30.78 | 30.75 | 30.82 | 30.97 |
| J 6,7 | 4 | 2.96 | 7.44 | 2.78 | 4.12 | 2.88 | 7.46 | 1.43 | 5.61 |
| J 7,8a | 1 | 0.52 | 9.88 | 1.62 | 8.98 | 0.48 | 6.84 | 0.85 | 8.96 |
| J 7,8b | 10 | 9.44 | 1.3 | 9.19 | 0.66 | 9.44 | 2.99 | 9.77 | 0.72 |
| J 8a,9 | 8 | 8.26 | 6.06 | 6.53 | 6.68 | 8.34 | 7.72 | 5.64 | 6.84 |
| J 8b,9 | 1.5 | 6.81 | 6.03 | 5.93 | 8.37 | 6.65 | 6.74 | 5.2 | 8.34 |
| J 9,10 | 10 | 10.45 | 11.17 | 11.23 | 10.63 | 10.64 | 11.05 | 11.4 | 10.45 |
| J 10,11a | 2 | 9.05 | 7.13 | 7.75 | 6.73 | 8.41 | 7.11 | 8.63 | 7.15 |

| | | | | | | | | | |
|---|---|---|---|---|---|---|---|---|---|
| J 10,11b | 6 | 7.19 | 7.95 | 7.1 | 8.41 | 6.72 | 6.14 | 6.71 | 9.02 |
| J 11a,12 | 10 | 11.21 | 8.64 | 6.69 | 2.5 | 3.81 | 2.62 | 6.36 | 5.41 |
| J 11b,12 | 5 | 5.54 | 5.55 | 3.01 | 3.81 | 2.3 | 9.45 | 9.67 | 10.98 |
| J 12,13 | 2 | 1.78 | 0.76 | 9.39 | 9.52 | 9.32 | 9.28 | 0.73 | 1.77 |

**Table S20.** Boltzmann averaged GIAO isotropic magnetic shielding constants (σ) of isomers 1-4 calculated at the B3LYP/6-31G** level of theory for compound **20**[13]

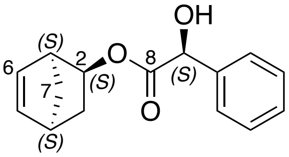


**20**

| Isotropic shielding constants | | | | | |
|---|---|---|---|---|---|
| **Nuclei** | **Experimental** | **Isomer 1** | **Isomer 2** | **Isomer 3** | **Isomer 4** |
| C 1 | 45.5 | 145.29 | 143.53 | 143.87 | 145.37 |
| C 2 | 76.8 | 115.08 | 115.56 | 115.56 | 115.24 |
| C 3 | 34.1 | 155.53 | 155.48 | 155.08 | 155.81 |
| C 4 | 42 | 148.88 | 150.17 | 150.29 | 148.72 |
| C 5 | 138.2 | 54.28 | 51.52 | 51.18 | 54.62 |
| C 6 | 130.9 | 60.9 | 59.41 | 59.65 | 60.41 |
| C 7 | 47.4 | 141.9 | 141.37 | 141.4 | 142.03 |
| C 8 | 173.7 | 18.29 | 18.32 | 18.4 | 18.19 |
| C 9 | 72.6 | 116.43 | 116.34 | 116.17 | 116.41 |
| C 10,11,12,13,14,15 | 138.3 | 66.93 | 66.83 | 66.805 | 66.905 |
| H 1 | 3 | 28.77 | 29.21 | 29.05 | 28.93 |
| H 2 | 5.34 | 26.51 | 27 | 27.03 | 26.48 |
| H 3x | 2.13 | 30.85 | 29.9 | 30.48 | 29.52 |
| H 3n | 0.99 | 29.6 | 30.17 | 29.98 | 30.59 |
| H 4 | 2.82 | 29.27 | 29.24 | 29.31 | 29.24 |
| H 5 | 6.22 | 25.32 | 25.22 | 25.22 | 25.3 |
| H 6 | 5.4 | 25.59 | 25.48 | 25.46 | 25.89 |
| H 7anti | 1.28 | 30.54 | 30.45 | 30.11 | 30.28 |
| H 7syn | 1.4 | 30.21 | 30.2 | 30.4 | 30.58 |
| H 9 | 5.06 | 26.58 | 26.56 | 26.56 | 26.59 |
| H 10,11,12,13,14,15 | 7.35 | 24.362 | 24.318 | 24.326 | 24.332 |
| J 5,6 | 5.6 | 6.61 | 6.63 | 6.63 | 6.61 |
| J 1,6 | 2.8 | 3.24 | 3.58 | 3.57 | 3.25 |
| J 2,3n | 3.6 | 2.4 | 6.54 | 1.88 | 7.44 |
| J 3n,4 | 3.1 | 7.38 | 1.89 | 6.42 | 2.39 |
| J 2,3x | 3.9 | 0.55 | 0.59 | 3.78 | 3.9 |
| J 3x,4 | 8.3 | 3.92 | 3.8 | 0.6 | 0.55 |
| J 1,7syn | 1.9 | 1.97 | 1.62 | 2.09 | 1.79 |

[13] Compounds **20**-**23**: NMR data from Pisano, P. L.; Sarotti, A. M.; Pellegrinet, S. C. *Tetrahedron Lett*. **2009**, *50*, 6121

**Table S21.** Boltzmann averaged GIAO isotropic magnetic shielding constants (σ) of isomers 1-4 calculated at the B3LYP/6-31G** level of theory for compound **21**

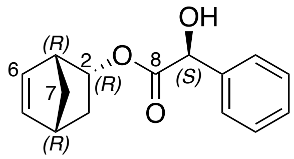


**21**

| Isotropic shielding constants | | | | | |
|---|---|---|---|---|---|
| **Nuclei** | **Experimental** | **Isomer 1** | **Isomer 2** | **Isomer 3** | **Isomer 4** |
| C 1 | 45.6 | 145.29 | 143.53 | 143.87 | 145.37 |
| C 2 | 77 | 115.08 | 115.56 | 115.56 | 115.24 |
| C 3 | 34.2 | 155.53 | 155.48 | 155.08 | 155.81 |
| C 4 | 42 | 148.88 | 150.17 | 150.29 | 148.72 |
| C 5 | 138.5 | 54.28 | 51.52 | 51.18 | 54.62 |
| C 6 | 131 | 60.9 | 59.41 | 59.65 | 60.41 |
| C 7 | 47.4 | 141.9 | 141.37 | 141.4 | 142.03 |
| C 8 | 173.5 | 18.29 | 18.32 | 18.4 | 18.19 |
| C 9 | 72.5 | 116.43 | 116.34 | 116.17 | 116.41 |
| C 10,11,12,13,14,15 | 138.1 | 66.93 | 66.84 | 66.81 | 66.91 |
| H 1 | 3.19 | 28.77 | 29.21 | 29.05 | 28.93 |
| H 2 | 5.34 | 26.51 | 27 | 27.03 | 26.48 |
| H 3x | 0.68 | 30.85 | 29.9 | 30.48 | 29.52 |
| H 3n | 2.04 | 29.6 | 30.17 | 29.98 | 30.59 |
| H 4 | 2.78 | 29.27 | 29.24 | 29.31 | 29.24 |
| H 5 | 6.22 | 25.32 | 25.22 | 25.22 | 25.3 |
| H 6 | 5.92 | 25.59 | 25.48 | 25.46 | 25.89 |
| H 7anti | 1.28 | 30.54 | 30.45 | 30.11 | 30.28 |
| H 7syn | 1.47 | 30.21 | 30.2 | 30.4 | 30.58 |
| H 9 | 5.08 | 26.58 | 26.56 | 26.56 | 26.59 |
| H 10,11,12,13,14,15 | 7.35 | 24.36 | 24.32 | 24.33 | 24.33 |
| J 5,6 | 5.6 | 6.61 | 6.63 | 6.63 | 6.61 |
| J 1,6 | 2.8 | 3.24 | 3.58 | 3.57 | 3.25 |
| J 2,3n | 3.2 | 2.4 | 6.54 | 1.88 | 7.44 |
| J 3n,4 | 3.6 | 7.38 | 1.89 | 6.42 | 2.39 |
| J 2,3x | 8.1 | 0.55 | 0.59 | 3.78 | 3.9 |
| J 3x,4 | 3.9 | 3.92 | 3.8 | 0.6 | 0.55 |
| J 1,7syn | 1.9 | 1.97 | 1.62 | 2.09 | 1.79 |

**Table S22.** Boltzmann averaged GIAO isotropic magnetic shielding constants (σ) of isomers 1-4 calculated at the B3LYP/6-31G** level of theory for compound **22**

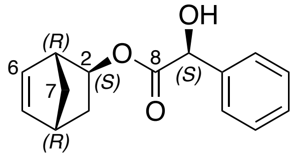


**22**

| Isotropic shielding constants | | | | | |
|---|---|---|---|---|---|
| **Nuclei** | **Experimental** | **Isomer 1** | **Isomer 2** | **Isomer 3** | **Isomer 4** |
| C 1 | 47 | 145.29 | 143.53 | 143.87 | 145.37 |
| C 2 | 77.1 | 115.08 | 115.56 | 115.56 | 115.24 |
| C 3 | 34.3 | 155.53 | 155.48 | 155.08 | 155.81 |
| C 4 | 40.4 | 148.88 | 150.17 | 150.29 | 148.72 |
| C 5 | 141.3 | 54.28 | 51.52 | 51.18 | 54.62 |
| C 6 | 132.1 | 60.9 | 59.41 | 59.65 | 60.41 |
| C 7 | 46 | 141.9 | 141.37 | 141.4 | 142.03 |
| C 8 | 173.7 | 18.29 | 18.32 | 18.4 | 18.19 |
| C 9 | 72.8 | 116.43 | 116.34 | 116.17 | 116.41 |
| H 1 | 2.68 | 28.77 | 29.21 | 29.05 | 28.93 |
| H 2 | 4.74 | 26.51 | 27 | 27.03 | 26.48 |
| H 3x | 1.72 | 30.85 | 29.9 | 30.48 | 29.52 |
| H 3n | 1.44 | 29.6 | 30.17 | 29.98 | 30.59 |
| H 4 | 2.82 | 29.27 | 29.24 | 29.31 | 29.24 |
| H 5 | 6.22 | 25.32 | 25.22 | 25.22 | 25.3 |
| H 6 | 5.9 | 25.59 | 25.48 | 25.46 | 25.89 |
| H 7anti | 1.44 | 30.54 | 30.45 | 30.11 | 30.28 |
| H 7syn | 1.44 | 30.21 | 30.2 | 30.4 | 30.58 |
| H 9 | 5.14 | 26.58 | 26.56 | 26.56 | 26.59 |
| J 5,6 | 5.5 | 6.61 | 6.63 | 6.63 | 6.61 |
| J 1,6 | 3.3 | 3.24 | 3.58 | 3.57 | 3.25 |
| J 3n,4 | 2.5 | 0.55 | 0.59 | 3.78 | 3.9 |

**Table S23.** Boltzmann averaged GIAO isotropic magnetic shielding constants (σ) of isomers 1-4 calculated at the B3LYP/6-31G** level of theory for compound **23**

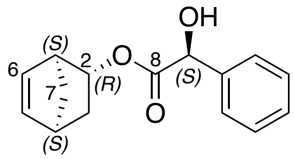


**23**

| Isotropic shielding constants | | | | | |
|---|---|---|---|---|---|
| **Nuclei** | **Experimental** | **Isomer 1** | **Isomer 2** | **Isomer 3** | **Isomer 4** |
| C 1 | 47.1 | 145.29 | 143.53 | 143.87 | 145.37 |
| C 2 | 77.1 | 115.08 | 115.56 | 115.56 | 115.24 |
| C 3 | 34.4 | 155.53 | 155.48 | 155.08 | 155.81 |
| C 4 | 40.4 | 148.88 | 150.17 | 150.29 | 148.72 |
| C 5 | 141.4 | 54.28 | 51.52 | 51.18 | 54.62 |
| C 6 | 132 | 60.9 | 59.41 | 59.65 | 60.41 |
| C 7 | 46.1 | 141.9 | 141.37 | 141.4 | 142.03 |
| C 8 | 173.5 | 18.29 | 18.32 | 18.4 | 18.19 |
| C 9 | 72.8 | 116.43 | 116.34 | 116.17 | 116.41 |
| H 1 | 2.94 | 28.77 | 29.21 | 29.05 | 28.93 |
| H 2 | 4.74 | 26.51 | 27 | 27.03 | 26.48 |
| H 3x | 1.11 | 30.85 | 29.9 | 30.48 | 29.52 |
| H 3n | 1.62 | 29.6 | 30.17 | 29.98 | 30.59 |
| H 4 | 2.77 | 29.27 | 29.24 | 29.31 | 29.24 |
| H 5 | 6.22 | 25.32 | 25.22 | 25.22 | 25.3 |
| H 6 | 5.95 | 25.59 | 25.48 | 25.46 | 25.89 |
| H 7anti | 1.56 | 30.54 | 30.45 | 30.11 | 30.28 |
| H 7syn | 1.56 | 30.21 | 30.2 | 30.4 | 30.58 |
| H 9 | 5.15 | 26.58 | 26.56 | 26.56 | 26.59 |
| J 5,6 | 5.7 | 6.61 | 6.63 | 6.63 | 6.61 |
| J 1,6 | 3.3 | 3.24 | 3.58 | 3.57 | 3.25 |
| J 2,3n | 2.3 | 2.4 | 6.54 | 1.88 | 7.44 |
| J 3n,4 | 2.5 | 7.38 | 1.89 | 6.42 | 2.39 |
| J 2,3x | 3.4 | 0.55 | 0.59 | 3.78 | 3.9 |

**Table S24.** Boltzmann averaged GIAO isotropic magnetic shielding constants (σ) of isomers 1-8 calculated at the B3LYP/6-31G** level of theory for compound **24**[14]

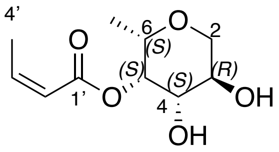


**24**

| Isotropic shielding constants | | | | | | | | | |
|---|---|---|---|---|---|---|---|---|---|
| **Nuclei** | **Experimental** | **Isomer 1** | **Isomer 2** | **Isomer 3** | **Isomer 4** | **Isomer 5** | **Isomer 6** | **Isomer 7** | **Isomer 8** |
| C 2 | 69.7 | 125.22 | 123.44 | 124.43 | 123.1 | 127.54 | 123.92 | 123.79 | 120.43 |
| C 3 | 67.7 | 122.85 | 121.76 | 118.09 | 120.47 | 121.29 | 121.81 | 122.67 | 120.54 |
| C 4 | 74.7 | 119.85 | 119.9 | 111.42 | 115.49 | 118.75 | 120.72 | 113.06 | 121.23 |
| C 5 | 72.9 | 114.83 | 117.74 | 111.97 | 112.93 | 115.3 | 116.68 | 116.19 | 116.68 |
| C 6 | 73.8 | 121.89 | 120.27 | 118.06 | 119.02 | 121.53 | 119.91 | 116.8 | 116.28 |
| C 7 | 16.7 | 172.91 | 171.95 | 171.81 | 171.77 | 176.62 | 173.87 | 172.83 | 172.62 |
| C 1' | 167 | 33.38 | 33.58 | 28.76 | 28.94 | 29.62 | 32.06 | 29 | 32.79 |
| C 2' | 119.9 | 74.78 | 74.69 | 75.19 | 74.81 | 74.46 | 74.27 | 74.61 | 74.32 |
| C 3' | 147 | 46.09 | 46.51 | 44.46 | 45.3 | 45.9 | 46.94 | 45.93 | 46.82 |
| C 4' | 15.7 | 175.35 | 175.36 | 175.28 | 175.21 | 175.33 | 175.55 | 175.54 | 175.5 |
| H 2ax | 3.18 | 28.24 | 27.8 | 28.61 | 28.45 | 27.88 | 27.93 | 28.58 | 28.36 |
| H 2eq | 3.99 | 28.05 | 28.2 | 27.87 | 27.63 | 28.06 | 28.04 | 27.73 | 27.7 |
| H 3 | 3.83 | 28.12 | 28.01 | 27.85 | 27.98 | 27.9 | 28.29 | 27.65 | 28.24 |
| H 4 | 3.67 | 27.5 | 27.6 | 28.07 | 28.13 | 27.84 | 27.58 | 27.98 | 28.37 |
| H 5 | 5.18 | 27.33 | 26.79 | 27.36 | 26.95 | 26.66 | 27.29 | 26.9 | 26.6 |
| H 6 | 3.67 | 27.83 | 27.92 | 28.31 | 28.47 | 27.54 | 27.72 | 28.15 | 28.39 |
| H 7a-c | 1.14 | 30.7 | 30.68 | 30.62 | 30.6 | 30.62 | 30.6 | 30.6 | 30.63 |
| H 2' | 5.89 | 25.95 | 25.97 | 25.82 | 25.83 | 25.81 | 25.89 | 25.73 | 25.91 |
| H 3' | 6.41 | 25.16 | 25.17 | 25.06 | 25.09 | 25.13 | 25.19 | 25.12 | 25.17 |
| H 4'a-c | 2.14 | 29.6 | 29.61 | 29.57 | 29.58 | 29.59 | 29.61 | 29.6 | 29.6 |
| J 2ax,3 | 10 | 8.79 | 1.37 | 10.46 | 1.23 | 2.2 | 2.14 | 10.25 | 1.17 |
| J 2eq,3 | 5.4 | 4.93 | 2.28 | 5.31 | 1.61 | 2.89 | 3.53 | 5.4 | 1.78 |
| J 3,4 | 9.6 | 2.91 | 3.97 | 8.17 | 3.27 | 3.02 | 4.04 | 8.64 | 2.63 |
| J 4,5 | 3.5 | 2.43 | 2.75 | 8.16 | 7.72 | 6.7 | 3.77 | 2.81 | 3.41 |
| J 5,6 | 1.1 | 8.16 | 8.79 | 8.95 | 9.01 | 4.81 | 2.21 | 0.84 | 0.72 |

[14] Compounds **24**: NMR data from Reátegui, R. F.; Gloer, J. B.; Campbell, J.; Shearer, C. A. *J. Nat. Prod.* **2005**, *68*, 701.

**Table S25.** Boltzmann averaged GIAO isotropic magnetic shielding constants (σ) of isomers 1-4 calculated at the B3LYP/6-31G** level of theory for compound **25**[15]

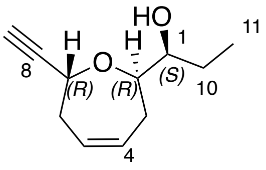


**25**

| Isotropic shielding constants | | | | | |
|---|---|---|---|---|---|
| **Nuclei** | **Experimental** | **Isomer 1** | **Isomer 2** | **Isomer 3** | **Isomer 4** |
| C 1 | 75.3 | 114.02 | 115.3 | 114.66 | 114.65 |
| C 2 | 76 | 115.19 | 105.21 | 106.24 | 114.6 |
| C 3 | 30.8 | 158.24 | 156.73 | 157.45 | 156.49 |
| C 4 | 130.9 | 60.85 | 62.38 | 61.54 | 61.56 |
| C 5 | 127.1 | 66.23 | 64.09 | 64.27 | 66.48 |
| C 6 | 35.7 | 154.97 | 152.29 | 152.49 | 154.98 |
| C 7 | 66.4 | 125.65 | 120.72 | 120.87 | 125.51 |
| C 8 | 82.3 | 118.11 | 115.82 | 115.76 | 117.86 |
| C 9 | 74.4 | 123.48 | 125.03 | 125.17 | 123.42 |
| C 10 | 25.3 | 164.3 | 163.04 | 164.48 | 162.57 |
| C 11 | 10.4 | 178.26 | 178.94 | 178.3 | 179.03 |
| H 1 | 3.55 | 28.24 | 28.29 | 28.32 | 28.34 |
| H 2 | 4.03 | 27.64 | 28.73 | 28.45 | 27.8 |
| H 3a | 2.24 | 29.78 | 29.29 | 29.87 | 29.23 |
| H 3b | 2.38 | 29.17 | 29.71 | 29.15 | 29.66 |
| H 4 | 5.88 | 25.53 | 25.64 | 25.58 | 25.58 |
| H 5 | 5.73 | 25.72 | 25.7 | 25.69 | 25.74 |
| H 6a | 2.51 | 29.46 | 28.9 | 29.48 | 29.07 |
| H 6b | 2.63 | 29.01 | 29.49 | 28.9 | 29.37 |
| H 7 | 4.82 | 27.05 | 27.71 | 27.73 | 27.01 |
| H 9 | 2.45 | 29.94 | 29.93 | 29.95 | 29.91 |
| H 10a | 1.49 | 30.25 | 30.32 | 30.24 | 30.3 |
| H 10b | 1.49 | 30.48 | 30.34 | 30.46 | 30.33 |
| H 11a-c | 0.98 | 30.74 | 30.72 | 30.73 | 30.74 |
| J 2,3b | 10.6 | 9.33 | 0.6 | 9.51 | 0.58 |
| J 2,3a | 1.8 | 0.38 | 9.54 | 0.49 | 9.4 |
| J 6b,7 | 3.8 | 3.82 | 0.76 | 9.95 | 4.56 |
| J 4,5 | 11 | 10.64 | 11 | 10.87 | 10.7 |
| J 3a,4 | 7.5 | 8.44 | 3.55 | 8.71 | 4.11 |
| J 3b,4 | 0 | 4.09 | 8.75 | 3.7 | 8.2 |
| J 1,2 | 3.85 | 2.15 | 6.06 | 2.31 | 5.25 |
| J 1,10b | 8.3 | 9.19 | 8.95 | 9.18 | 8.6 |
| J 1,10a | 4.1 | 2.08 | 2.14 | 1.98 | 2.4 |

[15] Compounds **25** and **26**: NMR data measured in our lab

**Table S26.** Boltzmann averaged GIAO isotropic magnetic shielding constants (σ) of isomers 1-4 calculated at the B3LYP/6-31G** level of theory for compound **26**

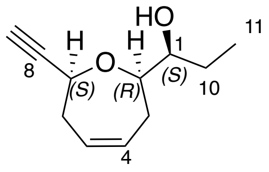


**26**

| Isotropic shielding constants | | | | | |
|---|---|---|---|---|---|
| **Nuclei** | **Experimental** | **Isomer 1** | **Isomer 2** | **Isomer 3** | **Isomer 4** |
| C 1 | 75.7 | 114.02 | 115.3 | 114.66 | 114.65 |
| C 2 | 83.4 | 115.19 | 105.21 | 106.24 | 114.6 |
| C 3 | 31.5 | 158.24 | 156.73 | 157.45 | 156.49 |
| C 4 | 130.6 | 60.85 | 62.38 | 61.54 | 61.56 |
| C 5 | 128.3 | 66.23 | 64.09 | 64.27 | 66.48 |
| C 6 | 38.5 | 154.97 | 152.29 | 152.49 | 154.98 |
| C 7 | 70.4 | 125.65 | 120.72 | 120.87 | 125.51 |
| C 8 | 83.7 | 118.11 | 115.82 | 115.76 | 117.86 |
| C 9 | 72.3 | 123.48 | 125.03 | 125.17 | 123.42 |
| C 10 | 25.4 | 164.3 | 163.04 | 164.48 | 162.57 |
| C 11 | 10.4 | 178.26 | 178.94 | 178.3 | 179.03 |
| H 1 | 3.56 | 28.24 | 28.29 | 28.32 | 28.34 |
| H 2 | 3.35 | 27.64 | 28.73 | 28.45 | 27.8 |
| H 3a | 2.23 | 29.78 | 29.29 | 29.87 | 29.23 |
| H 3b | 2.37 | 29.17 | 29.71 | 29.15 | 29.66 |
| H 4 | 5.85 | 25.53 | 25.64 | 25.58 | 25.58 |
| H 5 | 5.73 | 25.72 | 25.7 | 25.69 | 25.74 |
| H 6a | 2.47 | 29.46 | 28.9 | 29.48 | 29.07 |
| H 6b | 2.66 | 29.01 | 29.49 | 28.9 | 29.37 |
| H 7 | 4.19 | 27.05 | 27.71 | 27.73 | 27.01 |
| H 9 | 2.45 | 29.94 | 29.93 | 29.95 | 29.91 |
| H 10a | 1.5 | 30.25 | 30.32 | 30.24 | 30.3 |
| H 10b | 1.5 | 30.48 | 30.34 | 30.46 | 30.33 |
| H 11a-c | 0.97 | 30.74 | 30.72 | 30.73 | 30.74 |
| J 2,3b | 10.5 | 9.33 | 0.6 | 9.51 | 0.58 |
| J 2,3a | 1.5 | 0.38 | 9.54 | 0.49 | 9.4 |
| J 6b,7 | 10.55 | 3.82 | 0.76 | 9.95 | 4.56 |
| J 6a,7 | 1.95 | 3.79 | 10.04 | 0.72 | 3.78 |
| J 4,5 | 11.15 | 10.64 | 11 | 10.87 | 10.7 |
| J 3a,4 | 7.9 | 8.44 | 3.55 | 8.71 | 4.11 |
| J 3b,4 | 3.1 | 4.09 | 8.75 | 3.7 | 8.2 |
| J 1,2 | 4 | 2.15 | 6.06 | 2.31 | 5.25 |
| J 1,10b | 8.5 | 9.19 | 8.95 | 9.18 | 8.6 |
| J 1,10a | 4 | 2.08 | 2.14 | 1.98 | 2.4 |

**Table S27.** Boltzmann averaged GIAO isotropic magnetic shielding constants (σ) of isomers 1-8 calculated at the B3LYP/6-31G** level of theory for compound **27**[16]

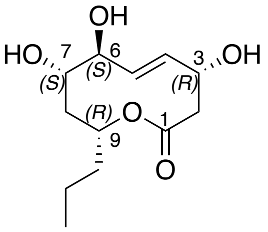


**27**

| Nuclei | Experimental | Isomer 1 | Isomer 2 | Isomer 3 | Isomer 4 | Isomer 5 | Isomer 6 | Isomer 7 | Isomer 8 |
|---|---|---|---|---|---|---|---|---|---|
| | | | | Isotropic shielding constants | | | | | |
| C 1 | 169.4 | 23.2 | 24.14 | 22.75 | 24.78 | 22.42 | 24.98 | 23 | 25.32 |
| C 2 | 45.2 | 149.69 | 150.07 | 148.93 | 149.22 | 148.16 | 149.32 | 149.18 | 150.61 |
| C 3 | 71.6 | 123.46 | 120.38 | 123.99 | 119.03 | 123.27 | 119.34 | 124.17 | 119.21 |
| C 4 | 136.4 | 55.95 | 54.97 | 61.54 | 55.45 | 58.2 | 59.24 | 57.06 | 55.53 |
| C 5 | 128.1 | 62.95 | 61.98 | 63.68 | 61.31 | 62.89 | 59.74 | 63.83 | 60.36 |
| C 6 | 78.1 | 115.71 | 113.42 | 116.48 | 115.24 | 116.71 | 120.3 | 112.22 | 116.66 |
| C 7 | 76 | 115.76 | 113.49 | 114.82 | 118.38 | 119.11 | 118.32 | 113.69 | 115.48 |
| C 8 | 41.1 | 156.47 | 151.17 | 149.26 | 152.93 | 152.49 | 158.02 | 148.69 | 156.36 |
| C 9 | 72.5 | 119.09 | 117.25 | 121.5 | 123.07 | 123.17 | 120.39 | 121.79 | 120.87 |
| C 10 | 39 | 155.28 | 150.82 | 150.35 | 151 | 150.81 | 151.19 | 150.17 | 154.71 |
| C 11 | 18.1 | 169.65 | 169.72 | 170.51 | 170.11 | 169.54 | 169.28 | 170.77 | 169.79 |
| C 12 | 13.2 | 176.07 | 176.08 | 177.48 | 176.12 | 176.07 | 176.42 | 177.55 | 176.29 |
| H 2a | 2.51 | 29.28 | 29.23 | 29.22 | 29.2 | 29.24 | 29.12 | 29.29 | 29.19 |
| H 2b | 2.21 | 29.43 | 29.47 | 29.31 | 29.47 | 29.29 | 29.51 | 29.32 | 29.38 |
| H 3 | 4.22 | 27.21 | 27.24 | 27.22 | 27.19 | 27.23 | 26.99 | 27.34 | 27.1 |
| H 4 | 5.61 | 25.36 | 25.64 | 25.61 | 25.82 | 25.68 | 25.51 | 25.71 | 25.25 |
| H 5 | 5.06 | 25.64 | 25.99 | 25.79 | 25.66 | 25.64 | 25.91 | 25.76 | 25.81 |
| H 6 | 3.57 | 27.66 | 27.83 | 27.09 | 27.74 | 27.59 | 27.69 | 27.76 | 27.7 |
| H 7 | 3.23 | 27.56 | 27.92 | 27.98 | 27.45 | 27.6 | 27.72 | 27.88 | 27.55 |
| H 8a | 1.71 | 30.22 | 29.97 | 29.27 | 29.67 | 30.09 | 30.03 | 29.88 | 29.71 |
| H 8b | 1.71 | 29.54 | 29.74 | 30.06 | 30.06 | 29.64 | 29.85 | 29.93 | 30.18 |
| H 9 | 4.68 | 26.88 | 27.15 | 26.68 | 26.41 | 26.3 | 26.69 | 26.59 | 26.82 |
| H 10a | 1.39 | 30.11 | 29.94 | 30.41 | 30.23 | 30.39 | 29.66 | 30.46 | 29.98 |
| H 10b | 1.39 | 30.01 | 30.42 | 29.96 | 30.33 | 30.18 | 30.23 | 30.02 | 30.2 |
| H 11a | 1.19 | 30.33 | 30.41 | 30.42 | 30.34 | 30.39 | 30.46 | 30.45 | 30.32 |
| H 11b | 1.19 | 30.28 | 30.4 | 30.57 | 30.42 | 30.38 | 30.38 | 30.59 | 30.33 |
| H 12a-c | 0.79 | 30.8 | 30.86 | 30.77 | 30.85 | 30.84 | 30.85 | 30.76 | 30.8 |
| J 2a,3 | 5.8 | 4.42 | 4.93 | 4.55 | 4.77 | 4.48 | 5.12 | 4.52 | 5.87 |
| J 2b,3 | 10.4 | 2.83 | 10.09 | 2.71 | 12.3 | 2.73 | 11.68 | 2.81 | 11.4 |
| J 3,4 | 8.6 | 3.27 | 6.92 | 3.72 | 7.77 | 3.28 | 7.47 | 3.73 | 8.09 |
| J 6,7 | 9.1 | 7.69 | 8 | 2.35 | 2.54 | 3.22 | 3.99 | 8.01 | 7.36 |

[16] Compounds **27** and **28**: NMR data from J. McNulty, D. McLeod, H. A. Jenkis, *Eur. J. Org. Chem.* **2016**

**Table S28.** Boltzmann averaged GIAO isotropic magnetic shielding constants (σ) of isomers 1-8 calculated at the B3LYP/6-31G** level of theory for compound **28**

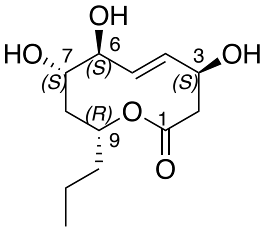

**28**

| | | | | Isotropic shielding constants | | | | | |
|---|---|---|---|---|---|---|---|---|---|
| **Nuclei** | **Experimental** | **Isomer 1** | **Isomer 2** | **Isomer 3** | **Isomer 4** | **Isomer 5** | **Isomer 6** | **Isomer 7** | **Isomer 8** |
| C 1 | 170.3 | 23.2 | 24.14 | 22.75 | 24.78 | 22.42 | 24.98 | 23 | 25.32 |
| C 2 | 44.5 | 149.69 | 150.07 | 148.93 | 149.22 | 148.16 | 149.32 | 149.18 | 150.61 |
| C 3 | 67.6 | 123.46 | 120.38 | 123.99 | 119.03 | 123.27 | 119.34 | 124.17 | 119.21 |
| C 4 | 136.3 | 55.95 | 54.97 | 61.54 | 55.45 | 58.2 | 59.24 | 57.06 | 55.53 |
| C 5 | 127.2 | 62.95 | 61.98 | 63.68 | 61.31 | 62.89 | 59.74 | 63.83 | 60.36 |
| C 6 | 79.7 | 115.71 | 113.42 | 116.48 | 115.24 | 116.71 | 120.3 | 112.22 | 116.66 |
| C 7 | 76.9 | 115.76 | 113.49 | 114.82 | 118.38 | 119.11 | 118.32 | 113.69 | 115.48 |
| C 8 | 42 | 156.47 | 151.17 | 149.26 | 152.93 | 152.49 | 158.02 | 148.69 | 156.36 |
| C 9 | 73 | 119.09 | 117.25 | 121.5 | 123.07 | 123.17 | 120.39 | 121.79 | 120.87 |
| C 10 | 39 | 155.28 | 150.82 | 150.35 | 151 | 150.81 | 151.19 | 150.17 | 154.71 |
| C 11 | 19 | 169.65 | 169.72 | 170.51 | 170.11 | 169.54 | 169.28 | 170.77 | 169.79 |
| C 12 | 14.2 | 176.07 | 176.08 | 177.48 | 176.12 | 176.07 | 176.42 | 177.55 | 176.29 |
| H 2a | 2.44 | 29.28 | 29.23 | 29.22 | 29.2 | 29.24 | 29.12 | 29.29 | 29.19 |
| H 2b | 2.55 | 29.43 | 29.47 | 29.31 | 29.47 | 29.29 | 29.51 | 29.32 | 29.38 |
| H 3 | 4.67 | 27.21 | 27.24 | 27.22 | 27.19 | 27.23 | 26.99 | 27.34 | 27.1 |
| H 4 | 5.88 | 25.36 | 25.64 | 25.61 | 25.82 | 25.68 | 25.51 | 25.71 | 25.25 |
| H 5 | 5.58 | 25.64 | 25.99 | 25.79 | 25.66 | 25.64 | 25.91 | 25.76 | 25.81 |
| H 6 | 3.74 | 27.66 | 27.83 | 27.09 | 27.74 | 27.59 | 27.69 | 27.76 | 27.7 |
| H 7 | 3.35 | 27.56 | 27.92 | 27.98 | 27.45 | 27.6 | 27.72 | 27.88 | 27.55 |
| H 9 | 4.74 | 26.88 | 27.15 | 26.68 | 26.41 | 26.3 | 26.69 | 26.59 | 26.82 |
| H 10a | 1.49 | 30.11 | 29.94 | 30.41 | 30.23 | 30.39 | 29.66 | 30.46 | 29.98 |
| H 10b | 1.49 | 30.01 | 30.42 | 29.96 | 30.33 | 30.18 | 30.23 | 30.02 | 30.2 |
| H 11a | 1.3 | 30.33 | 30.41 | 30.42 | 30.34 | 30.39 | 30.46 | 30.45 | 30.32 |
| H 11b | 1.3 | 30.28 | 30.4 | 30.57 | 30.42 | 30.38 | 30.38 | 30.59 | 30.33 |
| H 12a-c | 0.89 | 30.8 | 30.86 | 30.77 | 30.85 | 30.84 | 30.85 | 30.76 | 30.8 |
| H 8a | 1.83 | 30.22 | 29.97 | 29.27 | 29.67 | 30.09 | 30.03 | 29.88 | 29.71 |
| H 8b | 1.83 | 29.54 | 29.74 | 30.06 | 30.06 | 29.64 | 29.85 | 29.93 | 30.18 |
| J 2a,3 | 3.3 | 4.42 | 4.93 | 4.55 | 4.77 | 4.48 | 5.12 | 4.52 | 5.87 |
| J 2b,3 | 3.9 | 2.83 | 10.09 | 2.71 | 12.3 | 2.73 | 11.68 | 2.81 | 11.4 |
| J 3,4 | 3.1 | 3.27 | 6.92 | 3.72 | 7.77 | 3.28 | 7.47 | 3.73 | 8.09 |

**Table S29.** Boltzmann averaged GIAO isotropic magnetic shielding constants (σ) of isomers 1-8 calculated at the B3LYP/6-31G** level of theory for compound **29**[17]

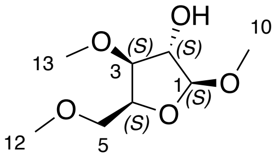


**29**

| | | Isotropic shielding constants | | | | | | | |
|---|---|---|---|---|---|---|---|---|---|
| **Nuclei** | **Experimental** | **Isomer 1** | **Isomer 2** | **Isomer 3** | **Isomer 4** | **Isomer 5** | **Isomer 6** | **Isomer 7** | **Isomer 8** |
| C 1 | 109.6 | 84.89 | 85.41 | 84.72 | 85.94 | 78.53 | 81.07 | 76.85 | 80.18 |
| C 2 | 78.8 | 116.58 | 113.58 | 116.89 | 116.14 | 119.04 | 117.4 | 117.21 | 113.79 |
| C 3 | 86 | 107.21 | 106.77 | 109 | 110.46 | 112.24 | 109.06 | 103.1 | 107.09 |
| C 4 | 80.1 | 112.07 | 110.7 | 106.75 | 111.58 | 112.1 | 112.25 | 105.96 | 111.03 |
| C 5 | 72.2 | 119.35 | 117.53 | 116.34 | 119.32 | 120.53 | 117.54 | 115.44 | 117.48 |
| C 10 | 59.2 | 137.9 | 138.26 | 138.6 | 138.7 | 137.88 | 137.78 | 138.32 | 138.2 |
| C 12 | 58.6 | 135.43 | 135.63 | 134.62 | 133.97 | 136.77 | 135.64 | 136.38 | 135.66 |
| C 13 | 55.8 | 134.29 | 134.39 | 133.92 | 134.11 | 134.18 | 134.22 | 134.59 | 134.08 |
| H 1 | 4.83 | 26.87 | 27.03 | 27.02 | 27.09 | 26.99 | 26.91 | 26.89 | 27.01 |
| H 2 | 4.21 | 27.46 | 27.66 | 27.48 | 27.77 | 27.72 | 27.54 | 27.66 | 27.38 |
| H 3 | 3.77 | 28.07 | 27.97 | 28.27 | 28.4 | 27.75 | 27.92 | 28.23 | 28.09 |
| H 4 | 4.48 | 27.26 | 27.62 | 27.47 | 27.61 | 27.56 | 27.45 | 27.36 | 27.32 |
| H 5a | 3.66 | 28.34 | 28.51 | 28.6 | 28.43 | 28.56 | 28.38 | 28.34 | 28.38 |
| H 5b | 3.57 | 28.48 | 28.4 | 28.57 | 28.16 | 28.25 | 28.52 | 28.62 | 28.26 |
| H 10a-c | 3.43 | 28.36 | 28.4 | 28.45 | 28.48 | 28.52 | 28.5 | 28.52 | 28.47 |
| H 12a-c | 3.44 | 28.36 | 28.37 | 28.37 | 28.32 | 28.41 | 28.42 | 28.55 | 28.46 |
| H 13a-c | 3.44 | 28.54 | 28.51 | 28.55 | 28.51 | 28.41 | 28.49 | 28.48 | 28.52 |
| J 1,2 | 1.3 | 3.89 | 3.79 | 4.67 | 4.74 | 0.17 | 0.89 | 0.19 | 2.62 |
| J 2,3 | 2.3 | 4.84 | 5.31 | 5 | 4.19 | 4.81 | 4.35 | 0.27 | 4.69 |
| J 3,4 | 5.9 | 5.47 | 5.31 | 0.43 | 3.6 | 8.33 | 6.01 | 0.93 | 5.91 |
| J 4,5a | 4.2 | 4.53 | 3.83 | 3.15 | 5.25 | 2.57 | 3.87 | 1.9 | 5.58 |
| J 4,5b | 7.7 | 3.99 | 5.81 | 2.03 | 6.49 | 0.65 | 5.93 | 1.18 | 3.82 |

[17] Compounds **29**: NMR data measured in our lab (Francisco, C. G.; Martin, C. G.; Suárez, E. J. *Org. Chem.* **1998**, *63*, 2099 )

**Table S30.** Boltzmann averaged GIAO isotropic magnetic shielding constants (σ) of isomers 1-4 calculated at the B3LYP/6-31G** level of theory for compound **30**[18]

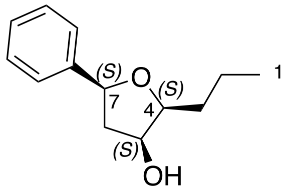


**30**

| Isotropic shielding constants | | | | | |
|---|---|---|---|---|---|
| **Nuclei** | **Experimental** | **Isomer 1** | **Isomer 2** | **Isomer 3** | **Isomer 4** |
| C 1 | 14.3 | 175.53 | 175.79 | 175.76 | 175.54 |
| C 2 | 19.6 | 168.96 | 168.85 | 168.78 | 168.48 |
| C 3 | 30.9 | 156.16 | 152.28 | 151.38 | 157.09 |
| C 4 | 83.6 | 109.06 | 105.91 | 105.78 | 109.33 |
| C 5 | 73.1 | 117.98 | 115.47 | 115.94 | 117.59 |
| C 6 | 44.3 | 148.14 | 148.16 | 147.87 | 147.56 |
| C 7 | 78.7 | 113.26 | 113.4 | 112.38 | 112.3 |
| C 8 | 143 | 49.06 | 50.22 | 51.14 | 51.13 |
| C 9 | 125.9 | 71.79 | 72.5 | 72.02 | 72.59 |
| C 10 | 128.4 | 68.46 | 68.48 | 69.16 | 69.02 |
| C 11 | 127.3 | 70.91 | 71.05 | 71.35 | 71.44 |
| C 12 | 128.4 | 68.3 | 68.69 | 69.02 | 68.9 |
| C 13 | 125.9 | 72.58 | 72.24 | 72.1 | 72.61 |
| H 1a-c | 0.92 | 30.76 | 30.77 | 30.77 | 30.75 |
| H 2a | 1.47 | 30.21 | 30.09 | 30.07 | 30.26 |
| H 2b | 1.4 | 30.18 | 30.26 | 30.27 | 30.04 |
| H 3a | 1.71 | 29.99 | 30.38 | 30.38 | 30 |
| H 3b | 1.63 | 30.15 | 30.31 | 30.27 | 30.39 |
| H 4 | 3.68 | 27.66 | 27.63 | 27.79 | 27.59 |
| H 5 | 4.19 | 27.81 | 27.72 | 27.66 | 27.69 |
| H 6a | 2.62 | 29.31 | 29.29 | 29.92 | 30.07 |
| H 6b | 1.83 | 29.54 | 29.74 | 29.56 | 29.37 |
| H 7 | 4.78 | 26.5 | 26.43 | 26.2 | 26.12 |
| H 9 | 7.25 | 24.31 | 24.2 | 24.32 | 24.34 |
| H 10 | 7.24 | 24.34 | 24.34 | 24.4 | 24.4 |
| H 11 | 7.17 | 24.53 | 24.54 | 24.56 | 24.56 |
| H 12 | 7.24 | 24.34 | 24.36 | 24.39 | 24.39 |
| H 13 | 7.25 | 24.16 | 24.33 | 24.28 | 24.3 |
| J 4,5 | 3.5 | 2.74 | 3.21 | 2.58 | 2.33 |
| J 5,6a | 6.2 | 4.52 | 4.99 | 4.68 | 3.92 |
| J 5,6b | 1.9 | 0.89 | 4.57 | 3.9 | 1.19 |
| J 6a,7 | 8.8 | 10.2 | 7.74 | 7.67 | 10.48 |
| J 6b,7 | 6.2 | 1.33 | 5.7 | 6.7 | 6.23 |

[18] Compounds **30**-**33**: NMR data from Grimblat, N. Gavín, J.A. Daranas, A.H., Sarotti, A, *Org. Lett.* **2019**, *21*, 11, 4003-4007

**Table S31.** Boltzmann averaged GIAO isotropic magnetic shielding constants (σ) of isomers 1-4 calculated at the B3LYP/6-31G** level of theory for compound **31**

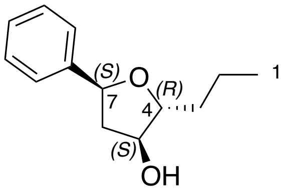


**31**

| Isotropic shielding constants | | | | | |
|---|---|---|---|---|---|
| **Nuclei** | **Experimental** | **Isomer 1** | **Isomer 2** | **Isomer 3** | **Isomer 4** |
| C 1 | 14.1 | 175.53 | 175.79 | 175.76 | 175.54 |
| C 2 | 19.1 | 168.96 | 168.85 | 168.78 | 168.48 |
| C 3 | 35.3 | 156.16 | 152.28 | 151.38 | 157.09 |
| C 4 | 85.6 | 109.06 | 105.91 | 105.78 | 109.33 |
| C 5 | 76.8 | 117.98 | 115.47 | 115.94 | 117.59 |
| C 6 | 43.4 | 148.14 | 148.16 | 147.87 | 147.56 |
| C 7 | 79.7 | 113.26 | 113.4 | 112.38 | 112.3 |
| C 8 | 143.3 | 49.06 | 50.22 | 51.14 | 51.13 |
| C 9 | 125.6 | 71.79 | 72.5 | 72.02 | 72.59 |
| C 10 | 128.5 | 68.46 | 68.48 | 69.16 | 69.02 |
| C 11 | 127.3 | 70.91 | 71.05 | 71.35 | 71.44 |
| C 12 | 128.5 | 68.3 | 68.69 | 69.02 | 68.9 |
| C 13 | 125.6 | 72.58 | 72.24 | 72.1 | 72.61 |
| H 1a-c | 0.89 | 30.76 | 30.77 | 30.77 | 30.75 |
| H 2a | 1.46 | 30.21 | 30.09 | 30.07 | 30.26 |
| H 2b | 1.37 | 30.18 | 30.26 | 30.27 | 30.04 |
| H 3a | 1.46 | 29.99 | 30.38 | 30.38 | 30 |
| H 3b | 1.46 | 30.15 | 30.31 | 30.27 | 30.39 |
| H 4 | 3.9 | 27.66 | 27.63 | 27.79 | 27.59 |
| H 5 | 4.05 | 27.81 | 27.72 | 27.66 | 27.69 |
| H 6a | 2.57 | 29.31 | 29.29 | 29.92 | 30.07 |
| H 6b | 1.87 | 29.54 | 29.74 | 29.56 | 29.37 |
| H 7 | 4.96 | 26.5 | 26.43 | 26.2 | 26.12 |
| H 9 | 7.26 | 24.31 | 24.2 | 24.32 | 24.34 |
| H 10 | 7.25 | 24.34 | 24.34 | 24.4 | 24.4 |
| H 11 | 7.18 | 24.53 | 24.54 | 24.56 | 24.56 |
| H 12 | 7.25 | 24.34 | 24.36 | 24.39 | 24.39 |
| H 13 | 7.26 | 24.16 | 24.33 | 24.28 | 24.3 |
| J 4,5 | 4.5 | 2.74 | 3.21 | 2.58 | 2.33 |
| J 5,6a | 6.5 | 4.52 | 4.99 | 4.68 | 3.92 |
| J 5,6b | 5.5 | 0.89 | 4.57 | 3.9 | 1.19 |
| J 6a,7 | 7.3 | 10.2 | 7.74 | 7.67 | 10.48 |
| J 6b,7 | 7.3 | 1.33 | 5.7 | 6.7 | 6.23 |

**Table S32.** Boltzmann averaged GIAO isotropic magnetic shielding constants (σ) of isomers 1-4 calculated at the B3LYP/6-31G** level of theory for compound **32**

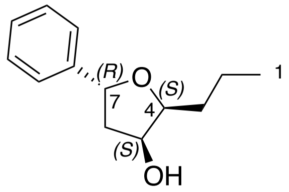


**32**

| Isotropic shielding constants | | | | | |
|---|---|---|---|---|---|
| **Nuclei** | **Experimental** | **Isomer 1** | **Isomer 2** | **Isomer 3** | **Isomer 4** |
| C 1 | 14.1 | 175.53 | 175.79 | 175.76 | 175.54 |
| C 2 | 19.1 | 168.96 | 168.85 | 168.78 | 168.48 |
| C 3 | 35.6 | 156.16 | 152.28 | 151.38 | 157.09 |
| C 4 | 87.1 | 109.06 | 105.91 | 105.78 | 109.33 |
| C 5 | 76.7 | 117.98 | 115.47 | 115.94 | 117.59 |
| C 6 | 44 | 148.14 | 148.16 | 147.87 | 147.56 |
| C 7 | 79.5 | 113.26 | 113.4 | 112.38 | 112.3 |
| C 8 | 142.1 | 49.06 | 50.22 | 51.14 | 51.13 |
| C 9 | 125.8 | 71.79 | 72.5 | 72.02 | 72.59 |
| C 10 | 128.3 | 68.46 | 68.48 | 69.16 | 69.02 |
| C 11 | 127.4 | 70.91 | 71.05 | 71.35 | 71.44 |
| C 12 | 128.3 | 68.3 | 68.69 | 69.02 | 68.9 |
| C 13 | 125.8 | 72.58 | 72.24 | 72.1 | 72.61 |
| H 1a-c | 0.91 | 30.76 | 30.77 | 30.77 | 30.75 |
| H 2a | 1.46 | 30.21 | 30.09 | 30.07 | 30.26 |
| H 2b | 1.41 | 30.18 | 30.26 | 30.27 | 30.04 |
| H 3a | 1.54 | 29.99 | 30.38 | 30.38 | 30 |
| H 3b | 1.5 | 30.15 | 30.31 | 30.27 | 30.39 |
| H 4 | 3.83 | 27.66 | 27.63 | 27.79 | 27.59 |
| H 5 | 4.09 | 27.81 | 27.72 | 27.66 | 27.69 |
| H 6a | 1.89 | 29.31 | 29.29 | 29.92 | 30.07 |
| H 6b | 2.13 | 29.54 | 29.74 | 29.56 | 29.37 |
| H 7 | 5.04 | 26.5 | 26.43 | 26.2 | 26.12 |
| H 9 | 7.27 | 24.31 | 24.2 | 24.32 | 24.34 |
| H 10 | 7.26 | 24.34 | 24.34 | 24.4 | 24.4 |
| H 11 | 7.19 | 24.53 | 24.54 | 24.56 | 24.56 |
| H 12 | 7.26 | 24.34 | 24.36 | 24.39 | 24.39 |
| H 13 | 7.27 | 24.16 | 24.33 | 24.28 | 24.3 |
| J 4,5 | 2.7 | 2.74 | 3.21 | 2.58 | 2.33 |
| J 5,6a | 5.9 | 4.52 | 4.99 | 4.68 | 3.92 |
| J 5,6b | 1.9 | 0.89 | 4.57 | 3.9 | 1.19 |
| J 6a,7 | 10.3 | 10.2 | 7.74 | 7.67 | 10.48 |
| J 6b,7 | 5.7 | 1.33 | 5.7 | 6.7 | 6.23 |

**Table S33.** Boltzmann averaged GIAO isotropic magnetic shielding constants (σ) of isomers 1-4 calculated at the B3LYP/6-31G** level of theory for compound **33**

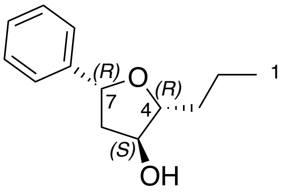


**33**

| Isotropic shielding constants | | | | | |
|---|---|---|---|---|---|
| **Nuclei** | **Experimental** | **Isomer 1** | **Isomer 2** | **Isomer 3** | **Isomer 4** |
| C 1 | 14.3 | 175.53 | 175.79 | 175.76 | 175.54 |
| C 2 | 19.7 | 168.96 | 168.85 | 168.78 | 168.48 |
| C 3 | 31.3 | 156.16 | 152.28 | 151.38 | 157.09 |
| C 4 | 83.1 | 109.06 | 105.91 | 105.78 | 109.33 |
| C 5 | 73.8 | 117.98 | 115.47 | 115.94 | 117.59 |
| C 6 | 44.7 | 148.14 | 148.16 | 147.87 | 147.56 |
| C 7 | 78.2 | 113.26 | 113.4 | 112.38 | 112.3 |
| C 8 | 143.8 | 49.06 | 50.22 | 51.14 | 51.13 |
| C 9 | 125.3 | 71.79 | 72.5 | 72.02 | 72.59 |
| C 10 | 128.3 | 68.46 | 68.48 | 69.16 | 69.02 |
| C 11 | 127.1 | 70.91 | 71.05 | 71.35 | 71.44 |
| C 12 | 128.3 | 68.3 | 68.69 | 69.02 | 68.9 |
| C 13 | 125.3 | 72.58 | 72.24 | 72.1 | 72.61 |
| H 1a-c | 0.94 | 30.76 | 30.77 | 30.77 | 30.75 |
| H 2a | 1.48 | 30.21 | 30.09 | 30.07 | 30.26 |
| H 2b | 1.4 | 30.18 | 30.26 | 30.27 | 30.04 |
| H 3a | 1.66 | 29.99 | 30.38 | 30.38 | 30 |
| H 3b | 1.58 | 30.15 | 30.31 | 30.27 | 30.39 |
| H 4 | 3.99 | 27.66 | 27.63 | 27.79 | 27.59 |
| H 5 | 4.3 | 27.81 | 27.72 | 27.66 | 27.69 |
| H 6a | 2.02 | 29.31 | 29.29 | 29.92 | 30.07 |
| H 6b | 2.39 | 29.54 | 29.74 | 29.56 | 29.37 |
| H 7 | 5.19 | 26.5 | 26.43 | 26.2 | 26.12 |
| H 9 | 7.26 | 24.31 | 24.2 | 24.32 | 24.34 |
| H 10 | 7.25 | 24.34 | 24.34 | 24.4 | 24.4 |
| H 11 | 7.18 | 24.53 | 24.54 | 24.56 | 24.56 |
| H 12 | 7.25 | 24.34 | 24.36 | 24.39 | 24.39 |
| H 13 | 7.26 | 24.16 | 24.33 | 24.28 | 24.3 |
| J 4,5 | 2.9 | 2.74 | 3.21 | 2.58 | 2.33 |
| J 5,6a | 4.5 | 4.52 | 4.99 | 4.68 | 3.92 |
| J 5,6b | 1 | 0.89 | 4.57 | 3.9 | 1.19 |
| J 6a,7 | 9.7 | 10.2 | 7.74 | 7.67 | 10.48 |
| J 6b,7 | 6.5 | 1.33 | 5.7 | 6.7 | 6.23 |

## 3. Comparison of isotropic magnetic shielding constants (σ) for the correct isomer in all FF for strychnine

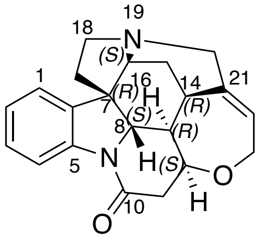


**1**

**Table S34.** Boltzmann averaged $^{13}C$ and $^{1}H$ GIAO isotropic magnetic shielding constants (σ) of isomers 1 calculated at the B3LYP/6-31G** level of theory for compound **1** for each FF and maximum Δσ

| Nuclei | AMBER | MM3 | MMFF | OPLS2005 | OPLS4 | MaxΔσ |
|---|---|---|---|---|---|---|
| C 1 | 73.65 | 75.79 | 75.41 | 72.46 | 75.06 | 3.33 |
| C 2 | 70.50 | 71.93 | 72.70 | 69.83 | 73.49 | 3.66 |
| C 3 | 65.53 | 66.95 | 67.27 | 65.18 | 68.20 | 3.02 |
| C 4 | 76.71 | 77.49 | 80.75 | 77.80 | 80.03 | 4.03 |
| C 5 | 55.24 | 61.05 | 56.06 | 53.52 | 55.47 | 7.53 |
| C 6 | 62.50 | 71.93 | 63.80 | 61.34 | 62.47 | 10.58 |
| C 7 | 138.73 | 136.02 | 138.09 | 137.58 | 138.66 | 2.71 |
| C 8 | 132.59 | 130.07 | 130.23 | 132.25 | 131.30 | 2.53 |
| C 10 | 32.32 | 35.64 | 31.98 | 30.92 | 32.63 | 4.72 |
| C 11 | 149.02 | 145.73 | 148.61 | 148.88 | 148.11 | 3.29 |
| C 12 | 113.73 | 110.58 | 111.18 | 111.66 | 113.77 | 3.19 |
| C 13 | 142.32 | 140.68 | 142.13 | 143.44 | 142.11 | 2.76 |
| C 14 | 158.13 | 155.70 | 157.80 | 155.97 | 156.11 | 2.42 |
| C 15 | 163.16 | 159.96 | 162.27 | 162.57 | 163.43 | 3.47 |
| C 16 | 130.65 | 130.35 | 131.17 | 129.64 | 127.70 | 3.47 |
| C 17 | 147.69 | 144.26 | 146.47 | 147.50 | 147.29 | 3.43 |
| C 18 | 141.06 | 139.32 | 141.82 | 141.81 | 143.28 | 3.96 |
| C 20 | 138.26 | 137.70 | 138.85 | 137.42 | 137.35 | 1.50 |
| C 21 | 51.99 | 54.91 | 53.34 | 52.19 | 48.30 | 6.62 |
| C 22 | 64.00 | 64.76 | 65.54 | 66.96 | 67.13 | 3.13 |
| C 23 | 128.68 | 126.21 | 128.71 | 127.80 | 130.30 | 4.09 |

| Nuclei | AMBER | MM3 | MMFF | OPLS2005 | OPLS4 | MaxΔσ |
|---|---|---|---|---|---|---|
| H 1 | 24.57 | 24.49 | 24.59 | 24.48 | 24.73 | 0.25 |
| H 2 | 24.67 | 24.53 | 24.75 | 24.69 | 24.82 | 0.29 |
| H 3 | 24.39 | 24.26 | 24.48 | 24.41 | 24.53 | 0.27 |
| H 4 | 23.45 | 23.36 | 23.51 | 23.79 | 23.81 | 0.46 |
| H 8 | 28.05 | 27.71 | 27.79 | 27.74 | 28.02 | 0.34 |
| H 11a | 27.87 | 27.35 | 27.71 | 28.01 | 27.62 | 0.66 |
| H 11b | 31.03 | 30.37 | 30.67 | 30.67 | 30.76 | 0.66 |
| H 12 | 29.00 | 28.39 | 28.86 | 29.16 | 28.92 | 0.77 |
| H 13 | 28.03 | 27.57 | 27.86 | 27.96 | 27.91 | 0.46 |
| H 14 | 25.79 | 25.46 | 25.72 | 25.94 | 25.90 | 0.47 |
| H 15a | 29.35 | 28.72 | 29.11 | 29.27 | 29.33 | 0.63 |
| H 15b | 28.93 | 28.43 | 28.74 | 29.14 | 28.93 | 0.71 |
| H 16 | 29.80 | 29.13 | 29.57 | 29.72 | 29.73 | 0.66 |
| H 17a | 30.62 | 29.97 | 30.37 | 30.51 | 30.49 | 0.65 |
| H 17b | 30.02 | 29.40 | 29.82 | 30.07 | 30.00 | 0.67 |
| H 18a | 30.11 | 29.55 | 29.97 | 30.09 | 30.12 | 0.58 |
| H 18b | 29.05 | 28.55 | 28.94 | 29.12 | 28.99 | 0.57 |
| H 20a | 28.80 | 28.34 | 28.75 | 28.91 | 28.63 | 0.56 |
| H 20b | 29.33 | 28.88 | 29.20 | 29.47 | 29.37 | 0.59 |
| H 22 | 28.27 | 27.78 | 28.16 | 28.37 | 28.26 | 0.59 |
| H 23a | 27.95 | 27.51 | 27.86 | 27.89 | 28.01 | 0.50 |
| H 23b | 28.11 | 27.69 | 28.07 | 28.18 | 28.06 | 0.49 |

## 4. Summary of errors

**Table S35.** CMAE for $^{1}H$ and $^{13}C$ for compounds **1**-**33**

| CMAE $^{1}H$ | **AMBER** | **MM3** | **MMFF** | **OPLS2005** | **OPLS4** |
|---|---|---|---|---|---|
| **1** | 0.08 | 0.09 | 0.09 | 0.14 | 0.08 |
| **2** | 0.08 | 0.10 | 0.08 | 0.08 | 0.11 |
| **3** | 0.11 | 0.10 | 0.11 | 0.09 | 0.14 |
| **4** | 0.10 | 0.10 | 0.09 | 0.09 | 0.12 |
| **5** | 0.09 | 0.13 | 0.09 | 0.11 | 0.12 |
| **6** | 0.18 | 0.24 | 0.08 | 0.09 | 0.19 |
| **7** | 0.14 | 0.11 | 0.17 | 0.07 | 0.12 |
| **8** | 0.12 | 0.16 | 0.10 | 0.13 | 0.12 |
| **9** | 0.12 | 0.13 | 0.14 | 0.12 | 0.15 |
| **10** | 0.09 | 0.16 | 0.11 | 0.10 | 0.09 |
| **11** | 0.12 | 0.18 | 0.17 | 0.11 | 0.14 |
| **12** | 0.26 | 0.03 | 0.10 | 0.12 | 0.18 |
| **13** | 0.30 | 0.15 | 0.09 | 0.08 | 0.12 |
| **14** | 0.24 | 0.13 | 0.13 | 0.14 | 0.11 |
| **15** | 0.13 | 0.16 | 0.14 | 0.26 | 0.11 |
| **16** | 0.13 | 0.15 | 0.13 | 0.17 | 0.12 |
| **17** | 0.12 | 0.16 | 0.14 | 0.53 | 0.14 |
| **18** | 0.15 | 0.16 | 0.13 | 0.12 | 0.09 |
| **19** | 0.16 | 0.15 | 0.12 | 0.12 | 0.12 |
| **20** | 0.26 | 0.42 | 0.32 | 0.30 | 0.35 |
| **21** | 0.18 | 0.17 | 0.15 | 0.14 | 0.22 |
| **22** | 0.19 | 0.21 | 0.18 | 0.14 | 0.23 |
| **23** | 0.32 | 0.38 | 0.34 | 0.33 | 0.33 |
| **24** | 0.17 | 0.14 | 0.11 | 0.14 | 0.10 |
| **25** | 0.19 | 0.15 | 0.17 | 0.13 | 0.13 |
| **26** | 0.23 | 0.20 | 0.18 | 0.17 | 0.13 |
| **27** | 0.12 | 0.09 | 0.10 | 0.09 | 0.11 |
| **28** | 0.15 | 0.10 | 0.11 | 0.10 | 0.12 |
| **29** | 0.11 | 0.09 | 0.08 | 0.09 | 0.12 |
| **30** | 0.17 | 0.13 | 0.14 | 0.15 | 0.13 |
| **31** | 0.11 | 0.12 | 0.10 | 0.13 | 0.09 |
| **32** | 0.11 | 0.11 | 0.10 | 0.10 | 0.09 |
| **33** | 0.14 | 0.13 | 0.14 | 0.16 | 0.12 |

| CMAE $^{13}C$ | **AMBER** | **MM3** | **MMFF** | **OPLS2005** | **OPLS4** |
|---|---|---|---|---|---|
| **1** | 1.23 | 2.22 | 1.39 | 1.22 | 1.86 |
| **2** | 0.93 | 1.21 | 1.28 | 1.37 | 1.59 |
| **3** | 2.82 | 1.77 | 1.55 | 1.29 | 1.89 |
| **4** | 1.83 | 1.97 | 1.77 | 1.91 | 2.23 |
| **5** | 2.51 | 2.50 | 2.37 | 2.26 | 2.55 |
| **6** | 2.71 | 2.58 | 1.97 | 1.68 | 1.87 |
| **7** | 3.18 | 3.02 | 1.27 | 1.68 | 2.04 |
| **8** | 1.69 | 1.38 | 2.09 | 1.25 | 1.23 |
| **9** | 1.70 | 1.53 | 1.12 | 0.93 | 1.51 |
| **10** | 1.47 | 1.30 | 1.32 | 0.70 | 1.41 |
| **11** | 1.59 | 1.41 | 1.07 | 0.78 | 1.54 |
| **12** | 0.43 | 0.89 | 0.64 | 1.44 | 0.81 |
| **13** | 1.49 | 0.59 | 0.62 | 1.03 | 0.89 |
| **14** | 0.48 | 0.72 | 1.06 | 1.33 | 1.69 |
| **15** | 1.99 | 2.11 | 2.07 | 2.53 | 1.37 |
| **16** | 1.02 | 1.62 | 1.14 | 1.26 | 1.31 |
| **17** | 1.25 | 2.11 | 1.81 | 2.53 | 1.31 |
| **18** | 2.47 | 2.94 | 2.55 | 1.51 | 1.48 |
| **19** | 2.21 | 2.73 | 2.35 | 1.49 | 1.41 |
| **20** | 2.40 | 3.45 | 3.66 | 2.83 | 4.42 |
| **21** | 1.40 | 1.06 | 1.07 | 1.29 | 1.18 |
| **22** | 1.40 | 1.27 | 1.08 | 1.35 | 1.12 |
| **23** | 2.45 | 3.52 | 0.71 | 2.86 | 4.43 |
| **24** | 2.05 | 1.72 | 1.63 | 1.90 | 2.02 |
| **25** | 2.07 | 2.39 | 2.46 | 1.69 | 1.72 |
| **26** | 2.90 | 2.57 | 2.37 | 1.72 | 1.55 |
| **27** | 1.88 | 2.13 | 0.99 | 0.83 | 1.12 |
| **28** | 2.36 | 1.83 | 0.92 | 1.05 | 1.06 |
| **29** | 2.49 | 2.09 | 2.27 | 2.60 | 2.08 |
| **30** | 1.22 | 1.31 | 1.77 | 1.22 | 1.81 |
| **31** | 0.91 | 1.22 | 1.54 | 1.08 | 0.43 |
| **32** | 1.00 | 1.39 | 1.61 | 1.42 | 1.50 |
| **33** | 0.87 | 1.18 | 1.52 | 0.99 | 1.53 |

**Table S36.** Maximum Error for $^{1}H$ and $^{13}C$ for compounds **1**-**33**

| MaxErr $^{1}H$ | **AMBER** | **MM3** | **MMFF** | **OPLS2005** | **OPLS4** |
|---|---|---|---|---|---|
| **1** | 0.20 | 0.23 | 0.27 | 0.36 | 0.19 |
| **2** | 0.17 | 0.28 | 0.22 | 0.19 | 0.27 |
| **3** | 0.28 | 0.29 | 0.27 | 0.16 | 0.29 |
| **4** | 0.23 | 0.23 | 0.27 | 0.26 | 0.34 |
| **5** | 0.20 | 0.30 | 0.21 | 0.21 | 0.31 |
| **6** | 0.36 | 0.77 | 0.16 | 0.15 | 0.33 |
| **7** | 0.21 | 0.31 | 0.32 | 0.19 | 0.22 |
| **8** | 0.28 | 0.41 | 0.29 | 0.27 | 0.33 |
| **9** | 0.30 | 0.28 | 0.43 | 0.41 | 0.44 |
| **10** | 0.22 | 0.51 | 0.30 | 0.25 | 0.24 |
| **11** | 0.38 | 0.59 | 0.51 | 0.34 | 0.42 |
| **12** | 0.55 | 0.06 | 0.16 | 0.25 | 0.26 |
| **13** | 0.50 | 0.22 | 0.17 | 0.13 | 0.25 |
| **14** | 0.51 | 0.24 | 0.21 | 0.20 | 0.24 |
| **15** | 0.38 | 0.37 | 0.36 | 0.45 | 0.31 |
| **16** | 0.27 | 0.47 | 0.34 | 0.47 | 0.23 |
| **17** | 0.31 | 0.39 | 0.31 | 1.15 | 0.28 |
| **18** | 0.33 | 0.37 | 0.29 | 0.28 | 0.27 |
| **19** | 0.39 | 0.52 | 0.32 | 0.28 | 0.33 |
| **20** | 0.29 | 0.36 | 0.31 | 0.29 | 0.49 |
| **21** | 0.28 | 0.32 | 0.32 | 0.28 | 0.52 |
| **22** | 0.28 | 0.36 | 0.32 | 0.25 | 0.51 |
| **23** | 0.34 | 0.34 | 0.33 | 0.30 | 0.36 |
| **24** | 0.48 | 0.35 | 0.38 | 0.42 | 0.36 |
| **25** | 0.46 | 0.46 | 0.51 | 0.23 | 0.26 |
| **26** | 0.49 | 0.49 | 0.52 | 0.26 | 0.33 |
| **27** | 0.32 | 0.18 | 0.29 | 0.25 | 0.34 |
| **28** | 0.39 | 0.38 | 0.39 | 0.31 | 0.24 |
| **29** | 0.23 | 0.18 | 0.18 | 0.27 | 0.45 |
| **30** | 0.38 | 0.29 | 0.38 | 0.40 | 0.39 |
| **31** | 0.30 | 0.23 | 0.23 | 0.44 | 0.25 |
| **32** | 0.26 | 0.31 | 0.40 | 0.32 | 0.25 |
| **33** | 0.31 | 0.38 | 0.35 | 0.41 | 0.39 |

| MaxErr $^{13}C$ | **AMBER** | **MM3** | **MMFF** | **OPLS2005** | **OPLS4** |
|---|---|---|---|---|---|
| **1** | 4.65 | 7.33 | 4.62 | 3.58 | 8.43 |
| **2** | 1.98 | 2.05 | 2.50 | 2.41 | 2.90 |
| **3** | 9.09 | 4.08 | 3.32 | 3.64 | 5.22 |
| **4** | 5.99 | 6.49 | 5.98 | 6.99 | 7.97 |
| **5** | 3.75 | 4.24 | 4.73 | 4.17 | 3.70 |
| **6** | 4.21 | 10.26 | 4.36 | 3.82 | 5.02 |
| **7** | 4.87 | 8.95 | 3.94 | 3.16 | 3.94 |
| **8** | 6.44 | 4.11 | 5.38 | 3.79 | 3.81 |
| **9** | 7.81 | 5.10 | 2.87 | 2.89 | 4.95 |
| **10** | 8.78 | 4.34 | 4.69 | 1.88 | 4.05 |
| **11** | 8.50 | 4.38 | 3.89 | 2.08 | 4.61 |
| **12** | 0.94 | 1.33 | 1.50 | 2.96 | 2.36 |
| **13** | 2.36 | 1.24 | 1.03 | 1.62 | 1.72 |
| **14** | 1.01 | 1.54 | 2.32 | 2.67 | 3.70 |
| **15** | 1.81 | 3.36 | 2.22 | 2.68 | 2.15 |
| **16** | 3.43 | 4.56 | 3.43 | 4.16 | 2.83 |
| **17** | 3.53 | 6.10 | 3.43 | 6.02 | 2.42 |
| **18** | 8.47 | 11.86 | 8.18 | 6.07 | 6.63 |
| **19** | 8.36 | 11.54 | 8.03 | 6.03 | 6.62 |
| **20** | 2.24 | 2.67 | 1.81 | 2.53 | 3.19 |
| **21** | 3.71 | 2.91 | 2.53 | 3.77 | 3.76 |
| **22** | 3.75 | 3.35 | 2.45 | 3.78 | 3.91 |
| **23** | 2.28 | 2.67 | 1.71 | 2.66 | 2.99 |
| **24** | 5.20 | 4.74 | 3.64 | 3.33 | 4.11 |
| **25** | 7.48 | 7.91 | 7.90 | 5.20 | 5.88 |
| **26** | 6.00 | 7.45 | 7.23 | 5.46 | 6.01 |
| **27** | 5.43 | 4.95 | 3.12 | 2.10 | 3.50 |
| **28** | 6.30 | 5.45 | 2.73 | 2.39 | 2.69 |
| **29** | 4.35 | 4.74 | 3.59 | 4.75 | 4.79 |
| **30** | 2.68 | 3.93 | 5.52 | 3.01 | 5.14 |
| **31** | 2.56 | 2.63 | 4.42 | 2.01 | 4.26 |
| **32** | 2.37 | 3.46 | 4.83 | 4.27 | 4.60 |
| **33** | 2.58 | 2.57 | 3.19 | 2.52 | 2.94 |

**5. Scatter plots of the Boltzmann´s amplitudes ($w_i$)**

**Figure 2.** Scatter plots of the Boltzmann´s amplitudes ($w_i$) calculated for the case study **9** at the B3LYP/6-31G** level using the geometries optimized at the different FFs under study as inputs.

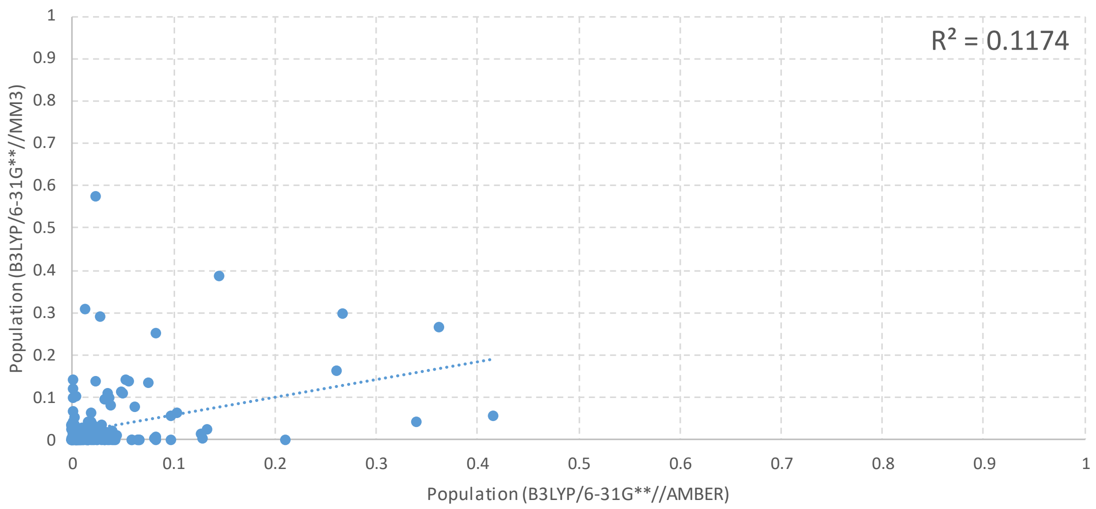


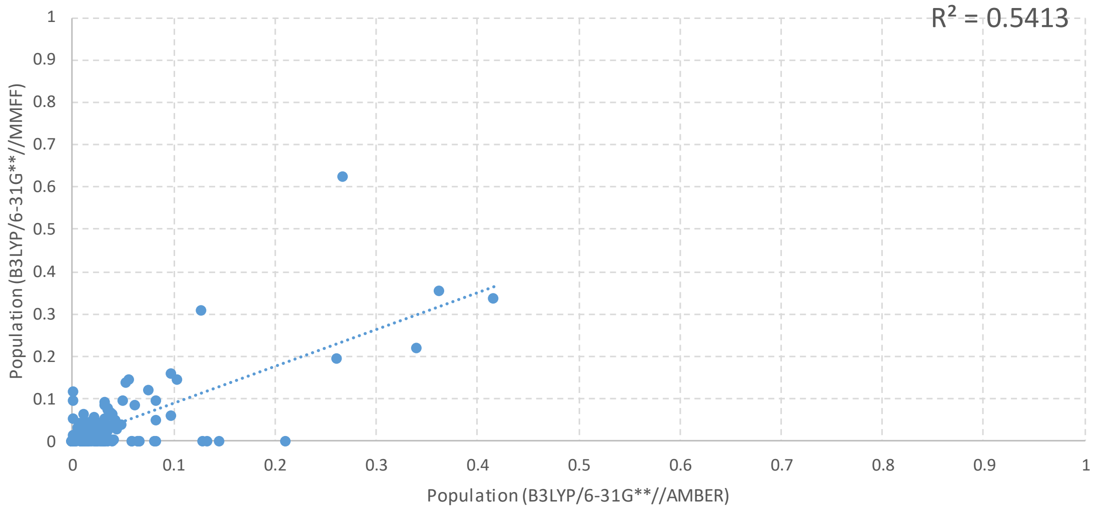


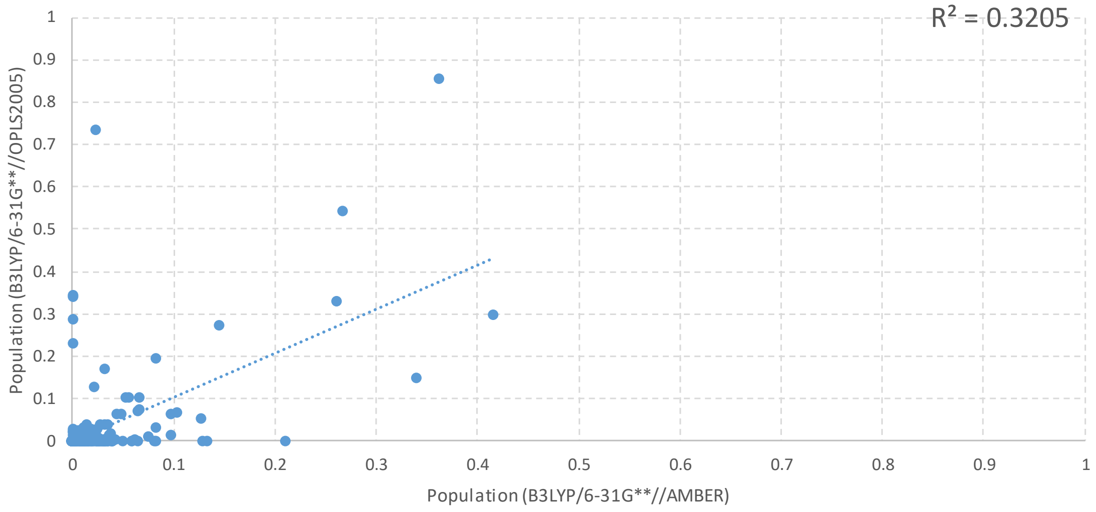

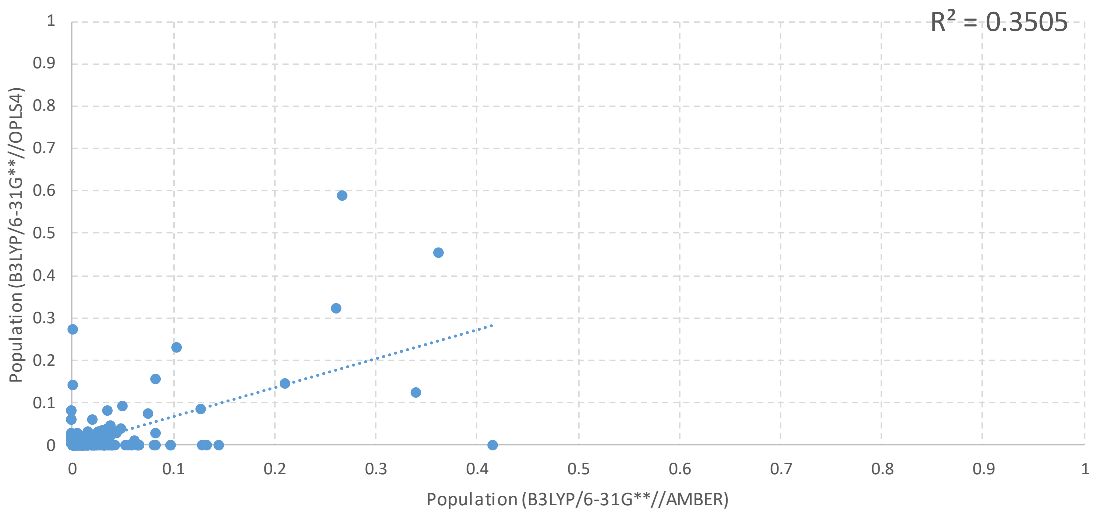

$R^2$ = 0.3505
Population (B3LYP/6-31G**//OPLS4)
Population (B3LYP/6-31G**//AMBER)
0
0.1
0.2
0.3
0.4
0.5
0.6
0.7
0.8
0.9
1


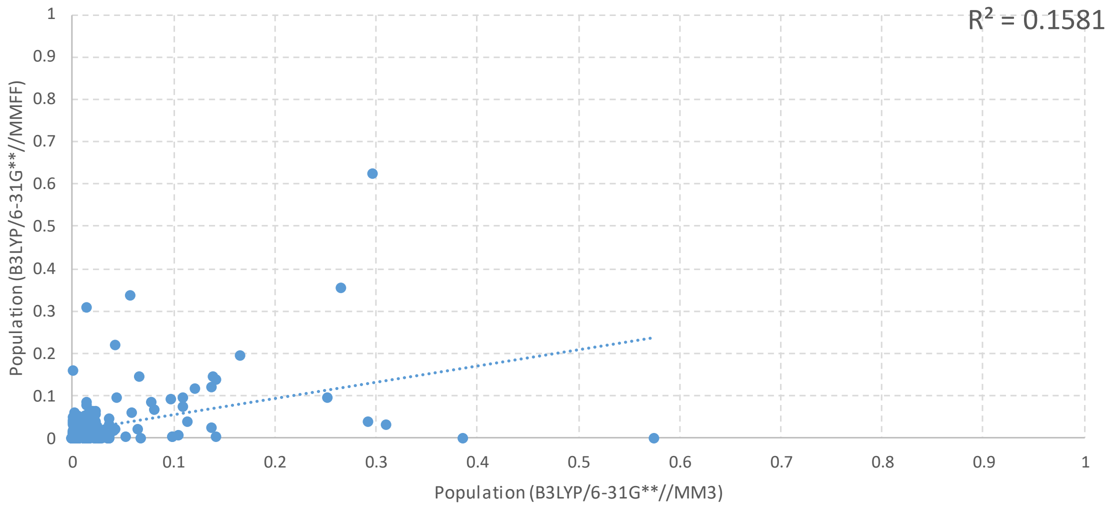

$R^2$ = 0.1581
Population (B3LYP/6-31G**//MMFF)
Population (B3LYP/6-31G**//MM3)
0
0.1
0.2
0.3
0.4
0.5
0.6
0.7
0.8
0.9
1


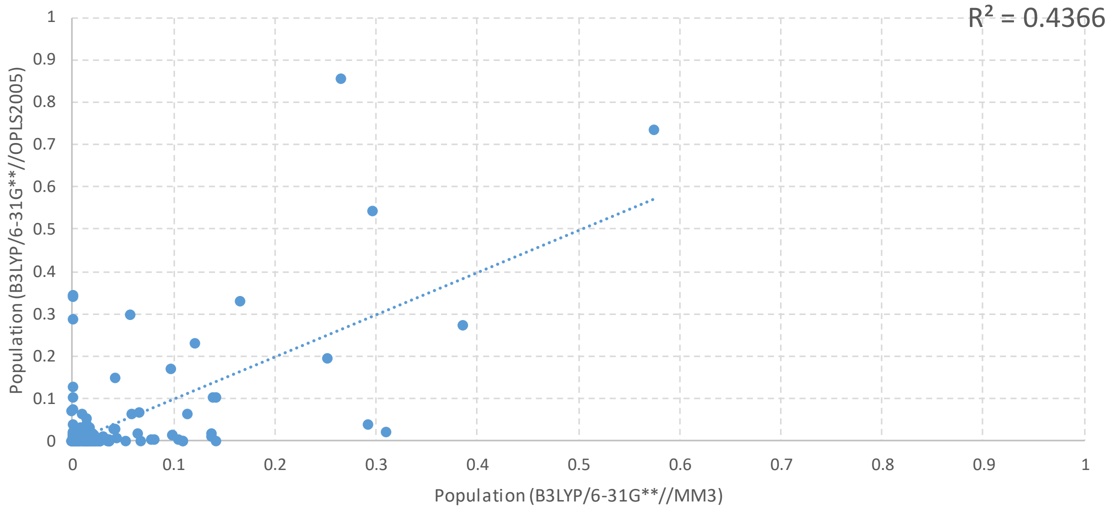

$R^2$ = 0.4366
Population (B3LYP/6-31G**//OPLS2005)
Population (B3LYP/6-31G**//MM3)
0
0.1
0.2
0.3
0.4
0.5
0.6
0.7
0.8
0.9
1

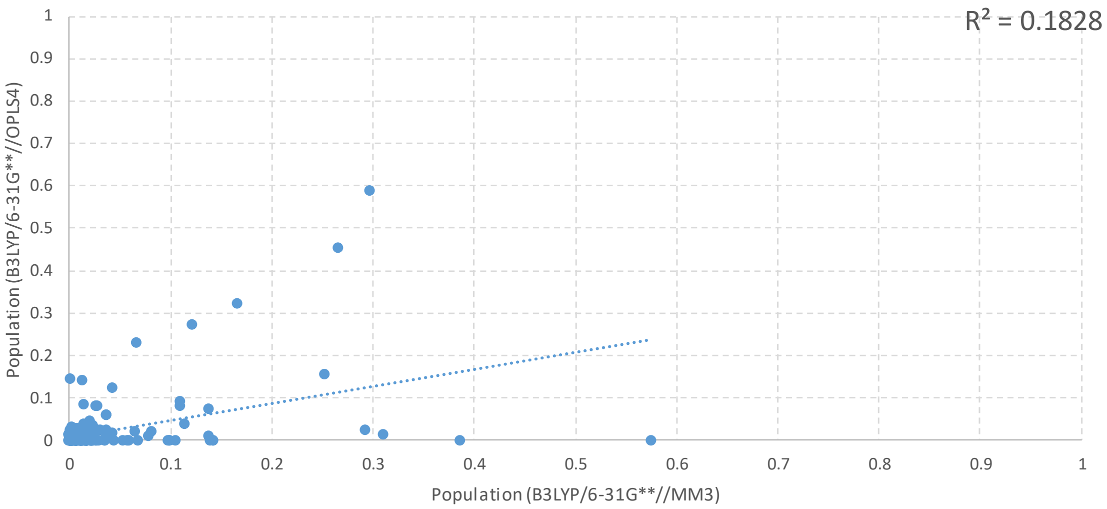
R² = 0.1828
Population (B3LYP/6-31G**//OPLS4)
Population (B3LYP/6-31G**//MM3)
0
0.1
0.2
0.3
0.4
0.5
0.6
0.7
0.8
0.9
1

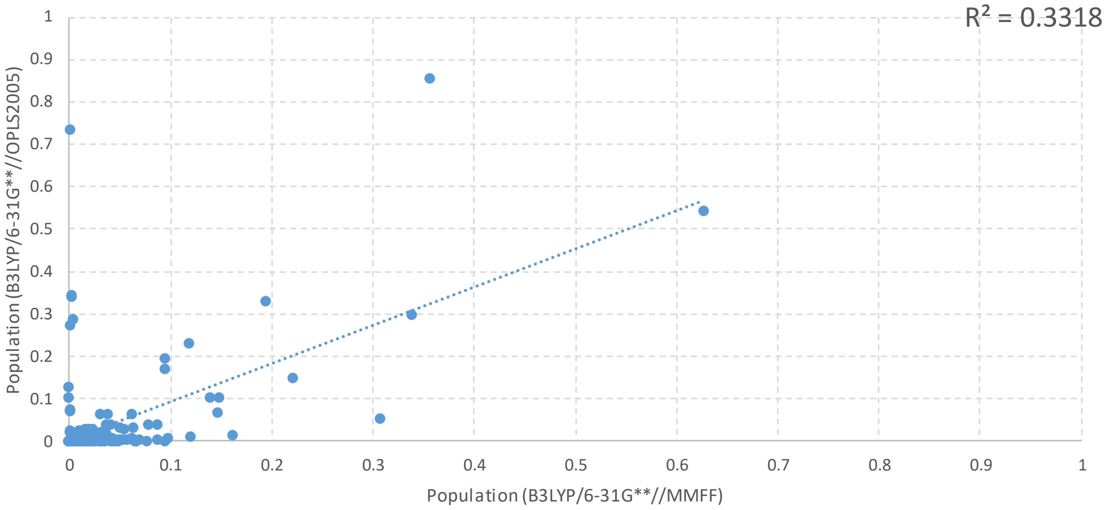
R² = 0.3318
Population (B3LYP/6-31G**//OPLS2005)
Population (B3LYP/6-31G**//MMFF)
0
0.1
0.2
0.3
0.4
0.5
0.6
0.7
0.8
0.9
1

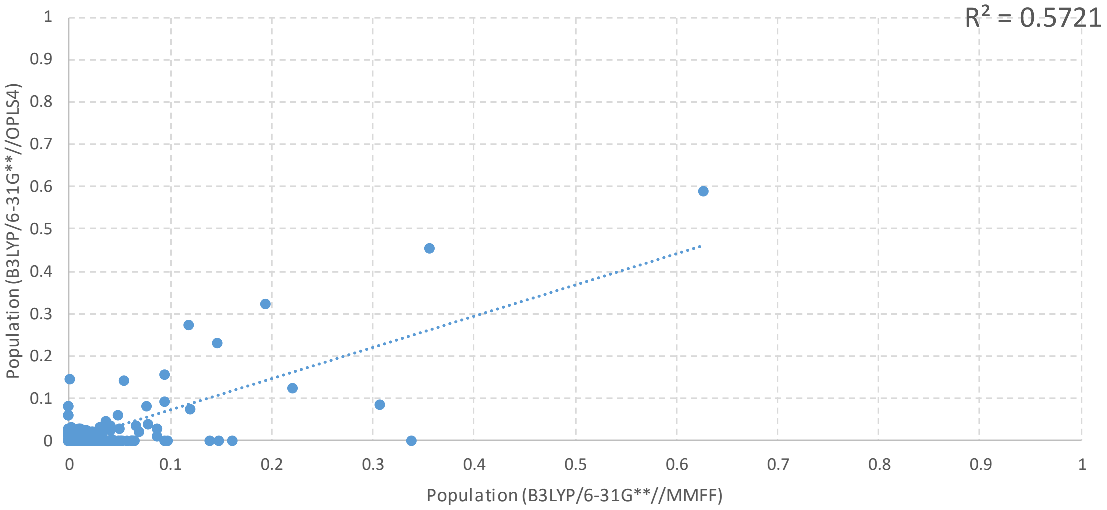
R² = 0.5721
Population (B3LYP/6-31G**//OPLS4)
Population (B3LYP/6-31G**//MMFF)
0
0.1
0.2
0.3
0.4
0.5
0.6
0.7
0.8
0.9
1

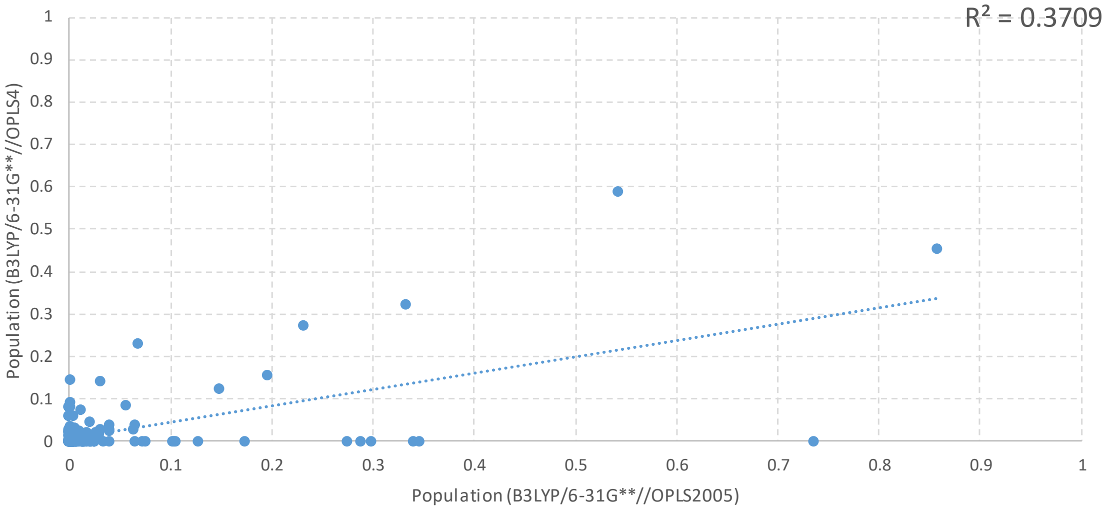

R² = 0.3709
1
0.9
0.8
0.7
0.6
0.5
0.4
0.3
0.2
0.1
0
Population (B3LYP/6-31G**//OPLS4)
0
0.1
0.2
0.3
0.4
0.5
0.6
0.7
0.8
0.9
1
Population (B3LYP/6-31G**//OPLS2005)

**Figure 3.** Scatter plots of the Boltzmann´s amplitudes ($w_i$) calculated for the case study **27** at the B3LYP/6-31G** level using the geometries optimized at the different FFs under study as inputs.

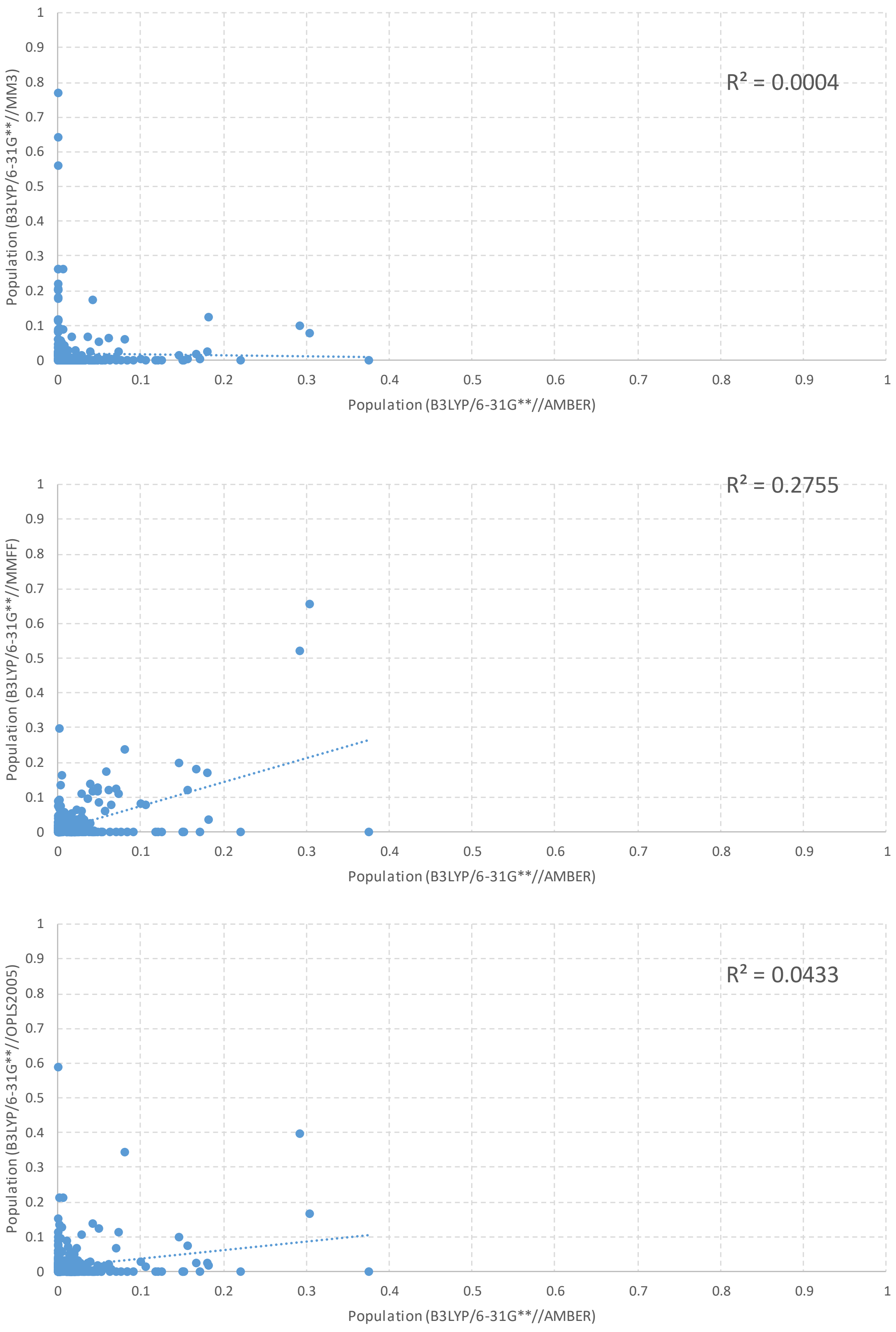

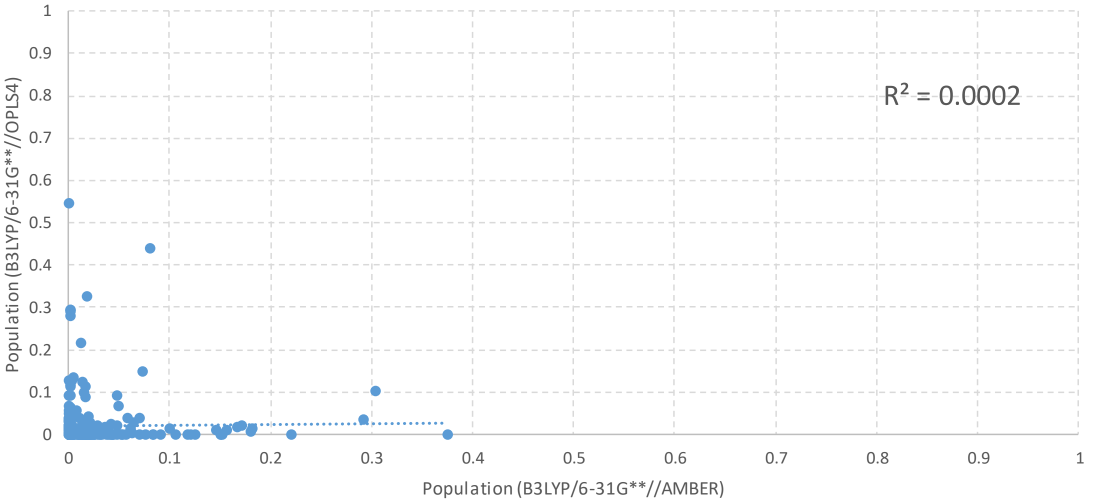
R² = 0.0002
Population (B3LYP/6-31G**//OPLS4)
Population (B3LYP/6-31G**//AMBER)
0
0.1
0.2
0.3
0.4
0.5
0.6
0.7
0.8
0.9
1

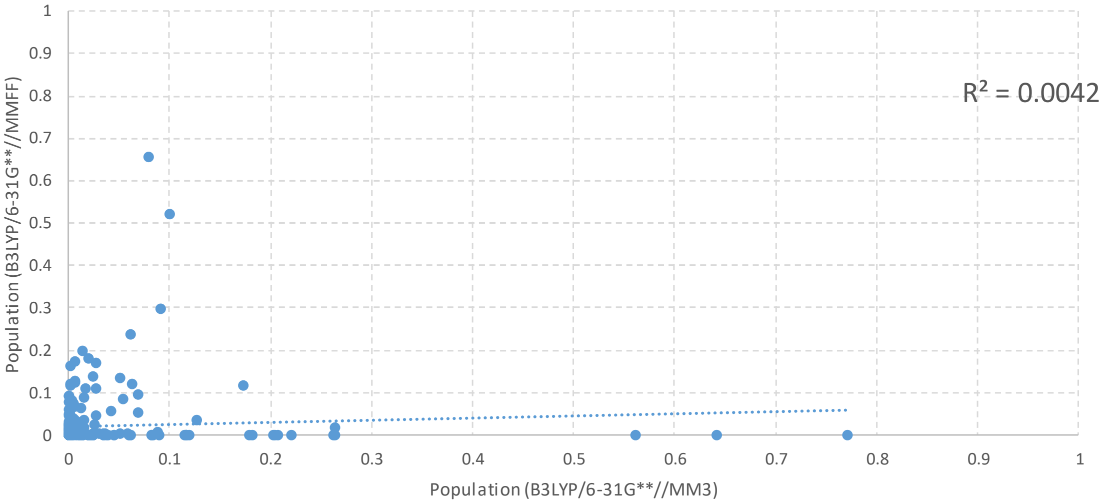
R² = 0.0042
Population (B3LYP/6-31G**//MMFF)
Population (B3LYP/6-31G**//MM3)
0
0.1
0.2
0.3
0.4
0.5
0.6
0.7
0.8
0.9
1

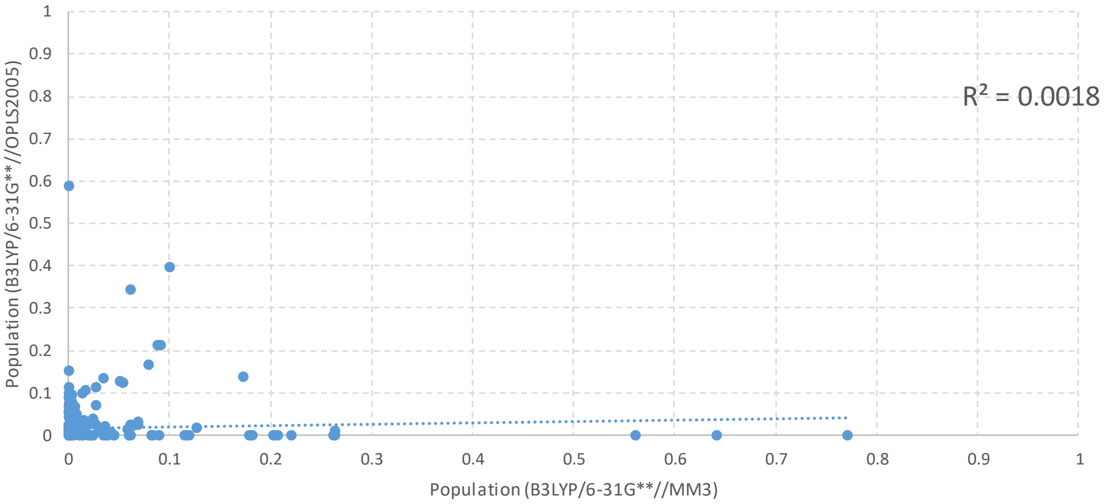
R² = 0.0018
Population (B3LYP/6-31G**//OPLS2005)
Population (B3LYP/6-31G**//MM3)
0
0.1
0.2
0.3
0.4
0.5
0.6
0.7
0.8
0.9
1

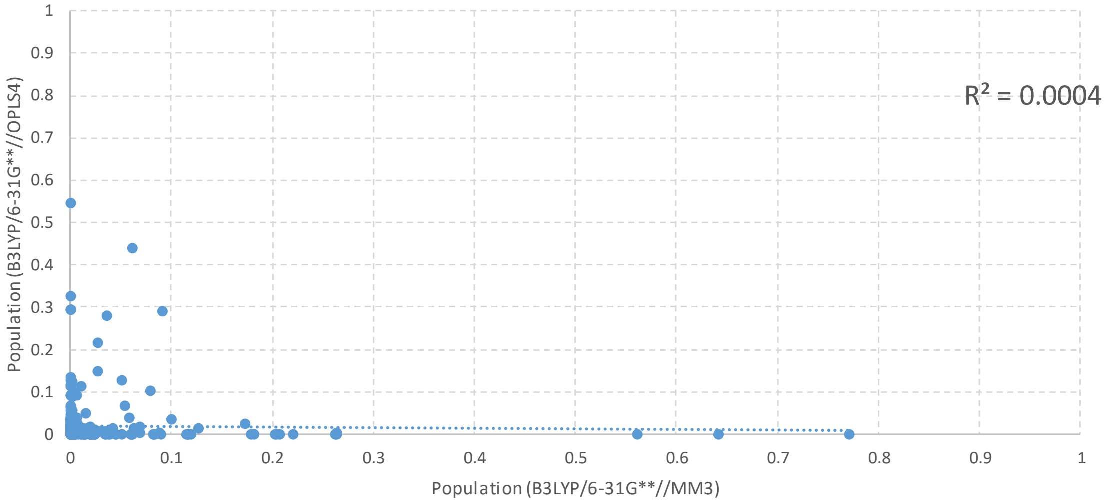
R² = 0.0004
Population (B3LYP/6-31G**//OPLS4)
Population (B3LYP/6-31G**//MM3)
0
0.1
0.2
0.3
0.4
0.5
0.6
0.7
0.8
0.9
1

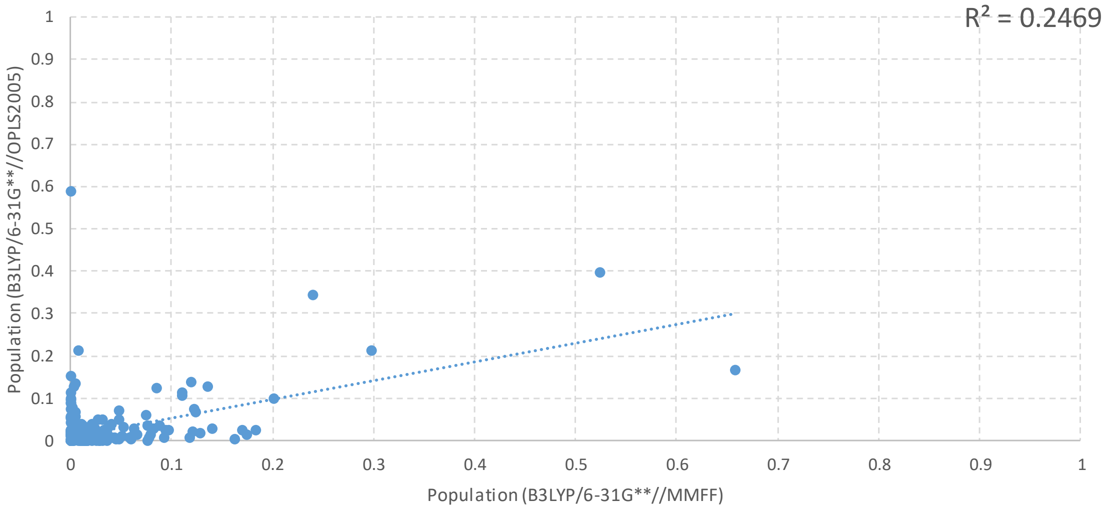
R² = 0.2469
Population (B3LYP/6-31G**//OPLS2005)
Population (B3LYP/6-31G**//MMFF)
0
0.1
0.2
0.3
0.4
0.5
0.6
0.7
0.8
0.9
1

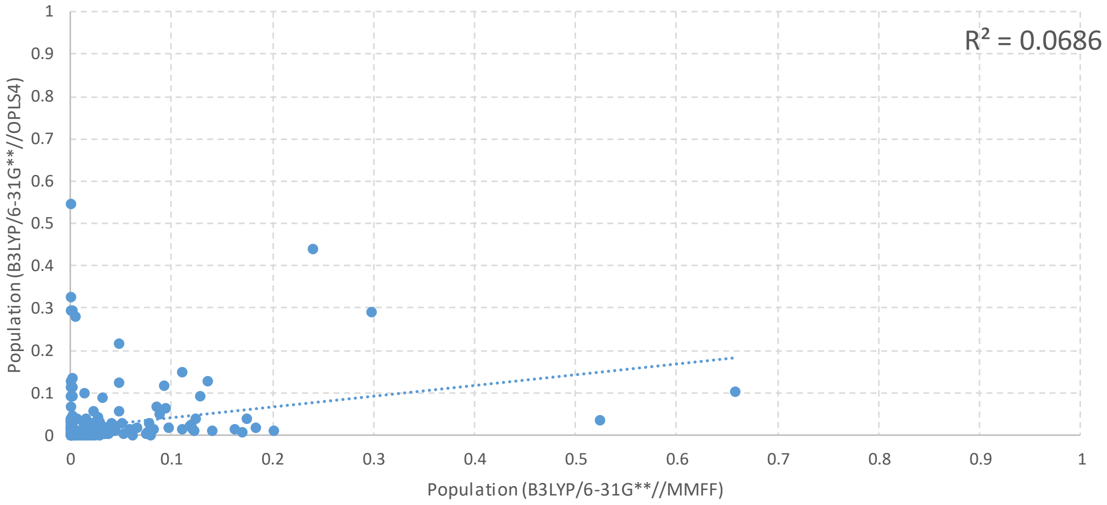
R² = 0.0686
Population (B3LYP/6-31G**//OPLS4)
Population (B3LYP/6-31G**//MMFF)
0
0.1
0.2
0.3
0.4
0.5
0.6
0.7
0.8
0.9
1

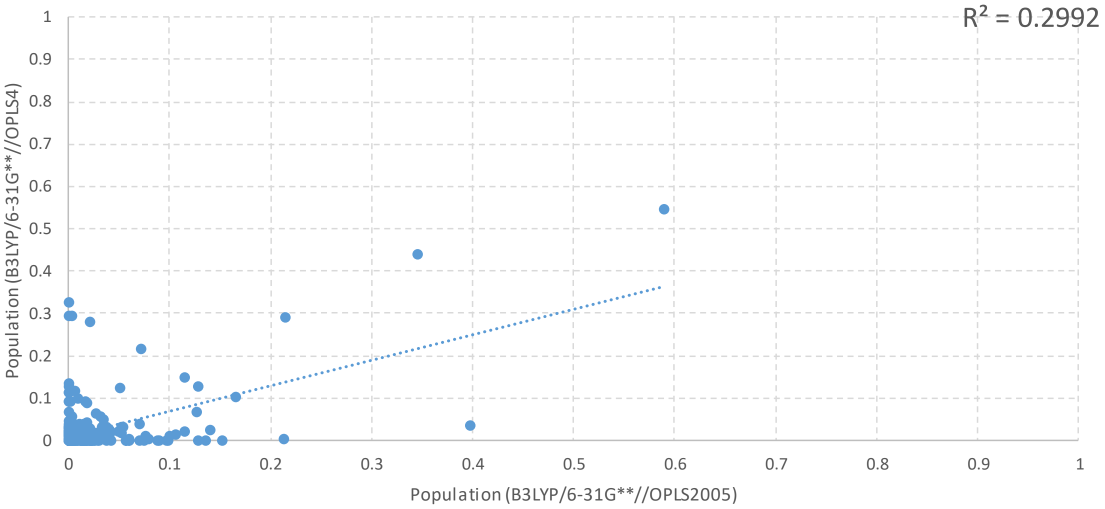
R² = 0.2992
Population (B3LYP/6-31G**//OPLS4)
Population (B3LYP/6-31G**//OPLS2005)
0
0.1
0.2
0.3
0.4
0.5
0.6
0.7
0.8
0.9
1

## 6. DP4, dJ-DP4 and iJ/dJ-DP4 results for compounds 1-33

**Table S37.** DP4 results for compounds **1-33**

| | DP4 – 12 kJ/mol – MAD 05 Å | | | | |
|---|---|---|---|---|---|
| Comp. № | **AMBER** | **MM3** | **MMFF** | **OPLS2005** | **OPLS4** |
| **1** | >99.9 | >99.9 | >99.9 | >99.9 | >99.9 |
| **2** | >99.9 | >99.9 | >99.9 | >99.9 | >99.9 |
| **3** | 94.0 | >99.9 | >99.9 | 99.5 | 99.9 |
| **4** | 99.8 | 99.7 | 99.5 | 92.8 | 61.9 |
| **5** | >99.9 | >99.9 | >99.9 | >99.9 | >99.9 |
| **6** | 92.7 | 52.7 | 98.6 | 92.5 | 1.4 |
| **7** | 92.1 | 98.9 | 99.9 | 2.9 | 62.6 |
| **8** | <0.1 | <0.1 | 95.5 | <0.1 | 48.6 |
| **9** | 53.2 | 99.7 | 97.4 | 20.5 | 9.7 |
| **10** | 99.3 | 73.3 | 99.9 | 99.9 | 99.8 |
| **11** | 1.8 | 53.1 | 59.1 | 99.8 | 47.2 |
| **12** | 43.7 | 67.6 | 52.2 | 26.2 | 1.1 |
| **13** | 2.7 | 13.5 | 87.2 | 62.7 | 52.6 |
| **14** | 62.3 | 50.9 | 1.7 | 23.8 | 33.0 |
| **15** | 99.5 | >99.9 | >99.9 | >99.9 | 99.9 |
| **16** | 11.5 | 51.3 | 59.2 | <0.1 | 55.7 |
| **17** | >99.9 | 99.6 | 98.5 | <0.1 | 99.9 |
| **18** | 8.6 | 0.7 | 8.2 | 40.8 | 31.1 |
| **19** | 96.4 | 99.5 | 99.5 | 99.5 | 98.2 |
| **20** | >99.9 | >99.9 | >99.9 | >99.9 | >99.9 |
| **21** | 99.4 | 97.8 | 97.5 | 99.7 | 98.2 |
| **22** | 95.2 | 69.7 | 98.1 | 99.8 | 95.9 |
| **23** | >99.9 | >99.9 | >99.9 | >99.9 | >99.9 |
| **24** | 99.5 | 13.9 | 98.9 | 99.3 | 99.1 |
| **25** | 59.8 | 55.6 | 35.7 | 96.3 | >99.9 |
| **26** | 79.1 | 80.9 | 32.1 | 81.3 | 99.2 |
| **27** | 99.6 | 93.6 | 99.8 | 95.5 | 74.2 |
| **28** | 7.6 | 98.6 | 60.2 | 93.0 | 66.7 |
| **29** | 3.5 | 14.3 | 4.6 | 44.1 | 0.9 |
| **30** | 0.6 | 99.7 | 99.3 | 98.6 | 99.1 |
| **31** | 95.0 | 99.4 | 34.0 | 74.8 | 0.1 |
| **32** | 84.7 | 27.0 | 23.1 | 71.3 | 7.3 |
| **33** | 90.9 | 99.5 | 0.7 | 75.6 | 37.0 |
| **Average** | **68.9** | **73.0** | **73.9** | **72.4** | **66.1** |

| | DP4 – 21 kJ/mol – MAD 05 Å | | | | |
|---|---|---|---|---|---|
| Comp. Nº | **AMBER** | **MM3** | **MMFF** | **OPLS2005** | **OPLS4** |
| **1** | >99.9 | >99.9 | >99.9 | >99.9 | >99.9 |
| **2** | >99.9 | >99.9 | >99.9 | >99.9 | >99.9 |
| **3** | 92.3 | >99.9 | >99.9 | 99.5 | 99.4 |
| **4** | 99.8 | 99.6 | 99.5 | 92.2 | 60.4 |
| **5** | >99.9 | >99.9 | >99.9 | >99.9 | >99.9 |
| **6** | 92.7 | 50.7 | 96.1 | 96.4 | 12.1 |
| **7** | 89.1 | 98.2 | 99.9 | 90.1 | 61.2 |
| **8** | 26.2 | 77.4 | 96.6 | 25.5 | 41.9 |
| **9** | 51.8 | >99.9 | 99.5 | 21.2 | 28.6 |
| **10** | 99.2 | 63.1 | 99.8 | 99.9 | 99.8 |
| **11** | 82.3 | 1.1 | 54.3 | 99.9 | 35.7 |
| **12** | 9.5 | 66.4 | 50.1 | 0.7 | 3.8 |
| **13** | 1.6 | 12.8 | 88.4 | 81.7 | 65.8 |
| **14** | 73.0 | 53.8 | 31.5 | 35.7 | 56.9 |
| **15** | 99.9 | >99.9 | >99.9 | >99.9 | >99.9 |
| **16** | 99.9 | 8.3 | 55.7 | 0.2 | 84.9 |
| **17** | 99.8 | 60.1 | 98.4 | <0.1 | >99.9 |
| **18** | 1.0 | 0.5 | 7.0 | 1.9 | 22.6 |
| **19** | 97.0 | 99.3 | 99.5 | 98.4 | 98.6 |
| **20** | >99.9 | >99.9 | >99.9 | >99.9 | >99.9 |
| **21** | 99.4 | 97.8 | 98.4 | 99.7 | 98.3 |
| **22** | 95.2 | 58.2 | 97.9 | 99.8 | 95.8 |
| **23** | >99.9 | >99.9 | >99.9 | >99.9 | >99.9 |
| **24** | 99.0 | 13.9 | 99.0 | 99.5 | 99.8 |
| **25** | 51.5 | 55.5 | 22.8 | 58.7 | 46.7 |
| **26** | 80.1 | 82.9 | 46.7 | 81.2 | 78.2 |
| **27** | 99.6 | 93.6 | 99.8 | 96.3 | 80.2 |
| **28** | 7.4 | 98.6 | 60.2 | 84.3 | 53.9 |
| **29** | 0.7 | 4.9 | 0.9 | 30.1 | 1.2 |
| **30** | 57.9 | 99.7 | 99.2 | 96.1 | 99.2 |
| **31** | 99.5 | 99.4 | 27.0 | 78.5 | 76.9 |
| **32** | 47.7 | 46.0 | 25.6 | 67.7 | 22.5 |
| **33** | 92.9 | 99.4 | 1.2 | 76.2 | 31.3 |
| **Average** | **74.1** | **70.9** | **74.4** | **73.1** | **68.4** |

| Comp. № | DP4 – 21 kJ/mol – MAD 1 Å | | | | |
|---|---|---|---|---|---|
| | **AMBER** | **MM3** | **MMFF** | **OPLS2005** | **OPLS4** |
| **1** | >99.9 | >99.9 | >99.9 | >99.9 | >99.9 |
| **2** | >99.9 | >99.9 | >99.9 | >99.9 | >99.9 |
| **3** | 92.3 | >99.9 | >99.9 | 99.5 | 99.4 |
| **4** | 99.8 | 99.6 | 99.5 | 92.2 | 60.4 |
| **5** | >99.9 | >99.9 | >99.9 | >99.9 | >99.9 |
| **6** | 92.7 | 50.2 | 94.8 | 95.1 | 12.1 |
| **7** | 89.1 | 98.2 | 99.9 | 90.1 | 61.2 |
| **8** | 26.2 | 78.8 | 96.5 | 17.4 | 41.7 |
| **9** | 55.4 | 6.3 | 99.8 | 39.6 | 5.3 |
| **10** | 99.5 | 13.7 | 99.7 | 99.9 | 99.9 |
| **11** | 65.6 | 99.0 | 25.4 | 99.8 | 8.9 |
| **12** | 9.5 | 64.6 | 50.1 | 1.9 | 3.9 |
| **13** | 1.6 | 12.9 | 85.8 | 78.6 | 59.4 |
| **14** | 73.0 | 54.3 | 50.9 | 37.2 | 60.4 |
| **15** | 99.1 | 95.6 | >99.9 | >99.9 | 99.9 |
| **16** | 28.0 | 41.5 | 60.2 | <0.1 | 97.3 |
| **17** | 99.9 | 73.4 | 99.0 | <0.1 | >99.9 |
| **18** | 0.8 | 0.4 | 7.2 | 2.4 | 26.2 |
| **19** | 97.0 | 99.3 | 99.4 | 98.1 | 98.4 |
| **20** | >99.9 | >99.9 | >99.9 | >99.9 | >99.9 |
| **21** | 99.4 | 97.8 | 98.4 | 99.7 | 98.3 |
| **22** | 95.2 | 58.2 | 97.9 | 99.8 | 95.8 |
| **23** | >99.9 | >99.9 | >99.9 | >99.9 | >99.9 |
| **24** | 98.9 | 4.8 | 98.5 | 99.6 | 99.7 |
| **25** | 57.7 | 61.7 | 36.3 | 66.6 | 46.7 |
| **26** | 80.9 | 39.9 | 33.3 | 55.0 | 78.1 |
| **27** | 98.1 | 94.0 | 99.9 | 98.9 | 94.1 |
| **28** | 56.2 | 98.8 | 29.1 | 72.3 | 40.2 |
| **29** | 1.1 | 3.8 | 1.1 | 54.1 | 2.1 |
| **30** | 27.3 | 98.5 | 99.2 | 98.7 | 90.5 |
| **31** | 99.6 | 99.6 | 18.8 | 84.1 | 67.4 |
| **32** | 44.7 | 4.0 | 27.1 | 57.2 | 23.1 |
| **33** | 93.5 | 99.1 | 1.1 | 80.9 | 73.2 |
| **Average** | **72.2** | **68.1** | **73.0** | **73.3** | **68.0** |

| | DP4 – 12 kJ/mol – RMSD 0.5 Å | | | | |
|---|---|---|---|---|---|
| Comp. Nº | **AMBER** | **MM3** | **MMFF** | **OPLS2005** | **OPLS4** |
| **1** | >99.9 | >99.9 | >99.9 | >99.9 | >99.9 |
| **2** | >99.9 | >99.9 | >99.9 | >99.9 | >99.9 |
| **3** | 93.5 | >99.9 | 73.5 | 99.5 | 99.9 |
| **4** | 99.9 | >99.9 | 97.6 | <0.1 | 84.2 |
| **5** | >99.9 | >99.9 | 99.4 | 0.4 | 2.9 |
| **6** | 5.2 | 53.0 | 97.6 | 92.5 | 1.4 |
| **7** | 83.3 | 98.9 | 99.9 | 2.9 | 62.6 |
| **8** | <0.1 | <0.1 | 33.8 | <0.1 | 62.8 |
| **9** | <0.1 | 18.7 | <0.1 | <0.1 | 0.2 |
| **10** | 98.7 | 99.2 | >99.9 | >99.9 | >99.9 |
| **11** | <0.1 | 95.2 | 99.6 | 99.9 | 2.6 |
| **12** | 57.9 | 21.8 | 27.0 | 3.1 | 1.4 |
| **13** | 3.0 | 11.0 | 8.1 | 85.9 | 36.5 |
| **14** | 54.2 | 42.0 | 5.6 | 5.0 | 37.8 |
| **15** | 98.0 | 8<0.1 | >99.9 | >99.9 | >99.9 |
| **16** | 0.1 | >99.9 | 46.1 | 99.9 | <0.1 |
| **17** | 93.7 | 3.4 | 94.2 | <0.1 | 99.9 |
| **18** | 22.0 | 2.7 | 5.1 | 9.6 | 50.6 |
| **19** | 95.7 | 71.4 | >99.9 | 88.2 | 22.6 |
| **20** | >99.9 | >99.9 | >99.9 | >99.9 | >99.9 |
| **21** | 99.4 | 97.8 | 97.5 | 99.7 | 98.2 |
| **22** | 95.2 | 69.7 | 98.1 | 99.8 | 95.9 |
| **23** | >99.9 | >99.9 | >99.9 | >99.9 | >99.9 |
| **24** | >99.9 | 6.1 | 99.4 | 99.5 | 98.9 |
| **25** | 59.8 | 50.9 | 80.7 | 96.3 | >99.9 |
| **26** | 79.0 | 74.8 | 31.4 | 77.4 | 99.1 |
| **27** | 99.8 | 94.5 | >99.9 | 95.2 | 61.6 |
| **28** | <0.1 | 96.8 | 3.1 | 92.2 | 93.6 |
| **29** | 23.7 | 15.6 | 15.5 | 51.4 | 5.0 |
| **30** | 1.9 | 97.3 | 52.4 | 99.6 | 78.4 |
| **31** | 99.2 | 99.6 | <0.1 | 86.9 | <0.1 |
| **32** | 33.9 | 1.3 | 0.5 | 0.3 | 29.7 |
| **33** | 86.0 | 99.1 | 26.9 | 90.4 | 86.5 |
| **Average** | **63.1** | **66.7** | **63.4** | **65.9** | **61.0** |

| | DP4 – 21 kJ/mol – RMSD 0.5 Å | | | | |
|---|---|---|---|---|---|
| Comp. Nº | **AMBER** | **MM3** | **MMFF** | **OPLS2005** | **OPLS4** |
| **1** | >99.9 | >99.9 | >99.9 | >99.9 | >99.9 |
| **2** | >99.9 | >99.9 | >99.9 | >99.9 | >99.9 |
| **3** | 93.1 | >99.9 | 72.6 | 99.5 | 99.3 |
| **4** | 99.9 | 99.9 | 97.4 | <0.1 | 82.8 |
| **5** | >99.9 | >99.9 | 99.4 | 0.4 | 7.7 |
| **6** | 5.2 | 50.2 | 93.9 | 95.1 | 12.1 |
| **7** | 45.0 | 98.2 | 99.9 | 90.1 | 61.2 |
| **8** | 96.0 | 41.4 | 56.6 | 71.7 | 65.3 |
| **9** | <0.1 | 48.4 | <0.1 | <0.1 | 0.6 |
| **10** | 98.4 | 93.0 | >99.9 | >99.9 | >99.9 |
| **11** | 1.8 | 61.1 | 15.8 | >99.9 | 1.5 |
| **12** | 11.9 | 21.1 | 11.2 | 1.8 | 6.1 |
| **13** | 1.8 | 10.7 | 75.8 | 94.9 | 54.6 |
| **14** | 71.8 | 45.6 | 29.7 | 4.4 | 73.7 |
| **15** | 98.0 | 81.6 | >99.9 | >99.9 | >99.9 |
| **16** | 0.1 | >99.9 | 39.5 | 28.2 | 77.5 |
| **17** | 82.4 | 3.7 | 92.7 | <0.1 | >99.9 |
| **18** | 2.8 | 2.3 | 5.0 | 1.4 | 49.7 |
| **19** | 95.7 | 72.6 | >99.9 | 86.5 | 21.8 |
| **20** | >99.9 | >99.9 | >99.9 | >99.9 | >99.9 |
| **21** | 99.4 | 97.8 | 98.4 | 99.7 | 98.3 |
| **22** | 95.2 | 58.1 | 97.9 | 99.8 | 95.8 |
| **23** | >99.9 | >99.9 | >99.9 | >99.9 | >99.9 |
| **24** | 99.9 | 6.2 | 99.5 | 99.7 | 99.7 |
| **25** | 51.6 | 50.8 | 58.1 | 58.4 | 46.6 |
| **26** | 79.8 | 76.1 | 36.7 | 77.9 | 70.5 |
| **27** | 99.8 | 94.8 | 0.9 | 87.8 | 53.0 |
| **28** | <0.1 | 96.3 | >99.9 | 95.7 | 94.6 |
| **29** | 2.4 | 2.3 | 3.1 | 27.5 | 0.5 |
| **30** | 2.8 | 97.7 | 52.4 | 99.6 | 82.5 |
| **31** | 99.8 | 99.7 | <0.1 | 93.2 | 0.1 |
| **32** | 12.1 | 1.1 | 1.3 | 0.4 | 24.1 |
| **33** | 36.8 | 98.6 | 28.8 | 89.1 | 83.9 |
| **Average** | **60.1** | **66.9** | **62.6** | **66.7** | **62.5** |

| | **DP4 – 21 kJ/mol – RMSD 1 Å** | | | | |
|---|---|---|---|---|---|
| Comp. Nº | **AMBER** | **MM3** | **MMFF** | **OPLS2005** | **OPLS4** |
| **1** | >99.9 | >99.9 | >99.9 | >99.9 | >99.9 |
| **2** | >99.9 | >99.9 | >99.9 | >99.9 | >99.9 |
| **3** | 6.7 | 87.5 | 35.0 | >99.9 | >99.9 |
| **4** | 99.9 | >99.9 | 97.6 | <0.1 | 84.2 |
| **5** | >99.9 | >99.9 | 99.4 | 0.5 | 8.1 |
| **6** | 2.7 | 22.3 | 95.4 | 84.1 | 1.2 |
| **7** | 80.2 | 49.9 | 98.1 | 0.9 | 48.9 |
| **8** | <0.1 | 90.5 | 99.9 | 0.9 | <0.1 |
| **9** | <0.1 | 0.2 | 59.2 | 0.1 | <0.1 |
| **10** | 99.9 | 97.9 | >99.9 | >99.9 | >99.9 |
| **11** | <0.1 | 99.8 | <0.1 | 2.7 | <0.1 |
| **12** | 1.0 | 36.7 | 6.7 | 0.2 | 0.2 |
| **13** | 19.5 | 43.2 | 96.4 | 94.0 | 0.1 |
| **14** | 64.0 | 0.4 | 2.2 | 61.7 | 12.9 |
| **15** | 3.1 | 0.1 | 92.8 | 99.9 | 90.1 |
| **16** | 21.1 | >99.9 | 1.2 | 0.3 | 0.3 |
| **17** | 96.8 | 3.2 | >99.9 | <0.1 | 98.0 |
| **18** | 28.4 | 0.8 | 4.6 | <0.1 | 97.5 |
| **19** | 84.3 | 93.0 | 99.9 | 0.2 | 99.2 |
| **20** | >99.9 | >99.9 | >99.9 | >99.9 | >99.9 |
| **21** | 99.4 | >99.9 | 99.9 | >99.9 | 98.3 |
| **22** | 95.2 | 0.2 | 77.6 | >99.9 | 95.9 |
| **23** | >99.9 | >99.9 | >99.9 | >99.9 | >99.9 |
| **24** | >99.9 | 7.0 | 99.5 | 99.7 | 45.1 |
| **25** | 78.0 | 54.4 | 56.2 | 46.6 | 15.2 |
| **26** | 99.8 | 17.5 | 61.0 | 55.7 | 1.4 |
| **27** | 99.3 | 12.2 | >99.9 | 96.8 | 49.5 |
| **28** | 0.5 | >99.9 | 3.4 | 96.8 | 97.7 |
| **29** | 6.6 | 72.7 | 15.9 | 0.2 | 51.0 |
| **30** | 0.2 | >99.9 | 28.6 | 0.2 | 83.2 |
| **31** | 99.9 | 3<0.1 | <0.1 | 99.8 | <0.1 |
| **32** | 2.7 | <0.1 | <0.1 | 0.2 | 99.3 |
| **33** | <0.1 | <0.1 | 43.9 | 2.6 | 44.7 |
| **Average** | **54.2** | **55.1** | **62.9** | **49.8** | **55.2** |

**Table S38.** dJ-DP4 results for compounds **1**-**33**

| Comp. Nº | **d*J*-DP4 – 12 kJ/mol – MAD 05 Å** | | | | |
|---|---|---|---|---|---|
| | **AMBER** | **MM3** | **MMFF** | **OPLS2005** | **OPLS4** |
| **1** | >99.9 | >99.9 | >99.9 | >99.9 | >99.9 |
| **2** | >99.9 | >99.9 | >99.9 | >99.9 | >99.9 |
| **3** | 97.9 | >99.9 | >99.9 | 99.9 | >99.9 |
| **4** | >99.9 | >99.9 | >99.9 | >99.9 | >99.9 |
| **5** | >99.9 | >99.9 | >99.9 | >99.9 | >99.9 |
| **6** | 13.1 | 1.7 | 99.8 | 97.4 | 0.2 |
| **7** | 79.9 | 96.2 | 99.9 | 0.1 | 43.3 |
| **8** | >99.9 | <0.1 | 0.8 | <0.1 | 78.6 |
| **9** | >99.9 | >99.9 | >99.9 | >99.9 | 87.1 |
| **10** | >99.9 | >99.9 | >99.9 | >99.9 | >99.9 |
| **11** | 99.6 | >99.9 | 99.9 | >99.9 | 30.1 |
| **12** | >99.9 | >99.9 | >99.9 | 99.2 | 96.0 |
| **13** | 63.6 | 99.5 | >99.9 | 99.9 | >99.9 |
| **14** | >99.9 | >99.9 | 99.6 | >99.9 | >99.9 |
| **15** | >99.9 | >99.9 | >99.9 | >99.9 | >99.9 |
| **16** | 0.1 | 99.9 | 0.1 | 0.1 | 0.1 |
| **17** | >99.9 | >99.9 | 90.7 | <0.1 | 99.9 |
| **18** | >99.9 | 74.1 | 41.2 | >99.9 | >99.9 |
| **19** | >99.9 | >99.9 | >99.9 | >99.9 | >99.9 |
| **20** | >99.9 | >99.9 | >99.9 | >99.9 | >99.9 |
| **21** | 99.2 | 97.0 | 97.2 | 99.7 | 97.6 |
| **22** | 98.9 | 95.3 | 99.8 | >99.9 | 99.7 |
| **23** | >99.9 | >99.9 | >99.9 | >99.9 | >99.9 |
| **24** | >99.9 | >99.9 | >99.9 | >99.9 | >99.9 |
| **25** | >99.9 | >99.9 | >99.9 | >99.9 | >99.9 |
| **26** | >99.9 | >99.9 | >99.9 | >99.9 | >99.9 |
| **27** | >99.9 | >99.9 | >99.9 | >99.9 | >99.9 |
| **28** | 99.1 | >99.9 | >99.9 | >99.9 | >99.9 |
| **29** | 91.8 | 99.8 | 88.3 | 93.2 | 19.3 |
| **30** | 0.3 | >99.9 | 94.0 | 1.1 | 72.1 |
| **31** | >99.9 | >99.9 | 99.5 | 1.9 | 9.1 |
| **32** | 82.4 | 89.3 | 18.2 | 68.5 | 71.1 |
| **33** | 97.4 | >99.9 | 13.9 | >99.9 | 97.7 |
| **Average** | **88.6** | **92.5** | **86.1** | **80.6** | **81.9** |

| | **d*J*-DP4 – 21 kJ/mol – MAD 05 Å** | | | | |
|---|---|---|---|---|---|
| Comp. Nº | **AMBER** | **MM3** | **MMFF** | **OPLS2005** | **OPLS4** |
| **1** | >99.9 | >99.9 | >99.9 | >99.9 | >99.9 |
| **2** | >99.9 | >99.9 | >99.9 | >99.9 | >99.9 |
| **3** | 98.9 | >99.9 | >99.9 | 99.9 | >99.9 |
| **4** | >99.9 | >99.9 | >99.9 | >99.9 | >99.9 |
| **5** | >99.9 | >99.9 | >99.9 | >99.9 | >99.9 |
| **6** | 13.1 | 0.9 | 98.1 | 99.0 | 3.1 |
| **7** | 79.8 | 96.2 | >99.9 | 99.2 | 39.8 |
| **8** | 99.9 | 99.8 | 3.8 | <0.1 | 21.9 |
| **9** | >99.9 | >99.9 | >99.9 | >99.9 | 87.8 |
| **10** | >99.9 | >99.9 | >99.9 | >99.9 | >99.9 |
| **11** | >99.9 | 98.0 | 99.9 | >99.9 | 7.4 |
| **12** | 99.9 | >99.9 | >99.9 | 97.4 | 98.5 |
| **13** | <0.1 | 99.5 | >99.9 | >99.9 | >99.9 |
| **14** | >99.9 | >99.9 | >99.9 | >99.9 | >99.9 |
| **15** | >99.9 | >99.9 | >99.9 | >99.9 | >99.9 |
| **16** | 99.0 | 0.1 | <0.1 | 85.2 | 2.4 |
| **17** | 99.4 | 6.7 | 95.5 | <0.1 | >99.9 |
| **18** | >99.9 | 61.0 | 33.0 | >99.9 | >99.9 |
| **19** | >99.9 | >99.9 | >99.9 | >99.9 | >99.9 |
| **20** | >99.9 | >99.9 | >99.9 | >99.9 | >99.9 |
| **21** | 99.2 | 96.9 | 98.2 | 99.7 | 97.6 |
| **22** | 98.9 | 92.5 | 99.8 | >99.9 | 99.7 |
| **23** | >99.9 | >99.9 | >99.9 | >99.9 | >99.9 |
| **24** | >99.9 | >99.9 | >99.9 | >99.9 | >99.9 |
| **25** | >99.9 | >99.9 | >99.9 | >99.9 | >99.9 |
| **26** | >99.9 | >99.9 | >99.9 | >99.9 | >99.9 |
| **27** | >99.9 | >99.9 | >99.9 | >99.9 | >99.9 |
| **28** | 99.1 | >99.9 | >99.9 | >99.9 | >99.9 |
| **29** | 5.2 | 82.9 | 16.7 | 96.1 | 0.7 |
| **30** | 6.3 | >99.9 | 90.4 | 0.3 | 77.8 |
| **31** | >99.9 | >99.9 | 98.7 | 4.2 | 92.3 |
| **32** | 73.1 | 98.4 | 15.7 | 56.2 | 12.6 |
| **33** | 99.9 | >99.9 | 26.4 | >99.9 | 96.4 |
| **Average** | **87.0** | **88.9** | **84.1** | **86.0** | **79.9** |

| | d*J*-DP4 – 21 kJ/mol – MAD 1 Å | | | | |
|---|---|---|---|---|---|
| Comp. Nº | **AMBER** | **MM3** | **MMFF** | **OPLS2005** | **OPLS4** |
| **1** | >99.9 | >99.9 | >99.9 | >99.9 | >99.9 |
| **2** | >99.9 | >99.9 | >99.9 | >99.9 | >99.9 |
| **3** | 98.9 | >99.9 | >99.9 | 99.9 | >99.9 |
| **4** | >99.9 | >99.9 | >99.9 | >99.9 | >99.9 |
| **5** | >99.9 | >99.9 | >99.9 | >99.9 | >99.9 |
| **6** | 13.1 | 0.9 | 97.4 | 98.6 | 3.1 |
| **7** | 79.8 | 96.2 | >99.9 | 99.2 | 39.8 |
| **8** | 99.9 | 99.9 | 3.2 | <0.1 | 21.5 |
| **9** | >99.9 | 93.4 | >99.9 | >99.9 | 91.1 |
| **10** | >99.9 | >99.9 | >99.9 | >99.9 | >99.9 |
| **11** | >99.9 | >99.9 | 99.8 | >99.9 | 1.6 |
| **12** | 99.9 | >99.9 | >99.9 | 99.0 | 98.7 |
| **13** | <0.1 | 99.5 | >99.9 | >99.9 | >99.9 |
| **14** | >99.9 | >99.9 | >99.9 | >99.9 | >99.9 |
| **15** | 7<0.1 | 7.1 | >99.9 | >99.9 | >99.9 |
| **16** | 2.7 | 0.7 | <0.1 | <0.1 | 99.9 |
| **17** | 99.8 | 0.7 | 88.0 | <0.1 | >99.9 |
| **18** | >99.9 | 65.2 | 33.8 | >99.9 | >99.9 |
| **19** | >99.9 | >99.9 | >99.9 | >99.9 | >99.9 |
| **20** | >99.9 | >99.9 | >99.9 | >99.9 | >99.9 |
| **21** | 99.2 | 96.9 | 98.2 | 99.7 | 97.6 |
| **22** | 98.9 | 92.5 | 99.8 | >99.9 | 99.7 |
| **23** | >99.9 | >99.9 | >99.9 | >99.9 | >99.9 |
| **24** | >99.9 | >99.9 | >99.9 | >99.9 | >99.9 |
| **25** | >99.9 | >99.9 | >99.9 | >99.9 | >99.9 |
| **26** | >99.9 | >99.9 | >99.9 | >99.9 | >99.9 |
| **27** | >99.9 | >99.9 | >99.9 | >99.9 | >99.9 |
| **28** | >99.9 | >99.9 | >99.9 | >99.9 | >99.9 |
| **29** | 14.9 | 68.5 | 20.1 | 99.7 | 4.5 |
| **30** | 2.3 | 99.9 | 90.9 | 0.3 | 21.9 |
| **31** | >99.9 | >99.9 | 97.4 | 7.4 | 89.9 |
| **32** | 74.5 | 93.5 | 16.0 | 81.5 | 10.1 |
| **33** | 99.9 | >99.9 | 26.0 | >99.9 | 99.4 |
| **Average** | **83.5** | **85.3** | **84.0** | **84.4** | **81.2** |

| Comp. Nº | dJ-DP4 – 12 kJ/mol – RMSD 0.5 Å | | | | |
|---|---|---|---|---|---|
| | **AMBER** | **MM3** | **MMFF** | **OPLS2005** | **OPLS4** |
| **1** | >99.9 | >99.9 | >99.9 | >99.9 | >99.9 |
| **2** | >99.9 | >99.9 | >99.9 | >99.9 | >99.9 |
| **3** | 97.0 | >99.9 | 94.8 | 99.8 | >99.9 |
| **4** | >99.9 | >99.9 | >99.9 | 59.3 | >99.9 |
| **5** | >99.9 | >99.9 | >99.9 | 99.7 | 99.9 |
| **6** | 0.7 | 1.8 | 99.6 | 97.4 | 0.2 |
| **7** | 90.9 | 96.2 | >99.9 | 0.1 | 43.3 |
| **8** | >99.9 | <0.1 | <0.1 | <0.1 | 96.8 |
| **9** | 97.6 | >99.9 | <0.1 | >99.9 | 3.7 |
| **10** | >99.9 | >99.9 | >99.9 | >99.9 | >99.9 |
| **11** | 34.4 | >99.9 | >99.9 | >99.9 | <0.1 |
| **12** | >99.9 | >99.9 | >99.9 | 99.9 | 96.5 |
| **13** | 65.1 | 99.6 | 99.9 | >99.9 | >99.9 |
| **14** | >99.9 | >99.9 | 99.9 | >99.9 | >99.9 |
| **15** | >99.9 | >99.9 | 80.2 | >99.9 | >99.9 |
| **16** | <0.1 | 99.6 | <0.1 | 99.1 | <0.1 |
| **17** | >99.9 | 99.5 | 99.2 | <0.1 | >99.9 |
| **18** | >99.9 | 23.3 | 12.8 | >99.9 | >99.9 |
| **19** | >99.9 | >99.9 | >99.9 | >99.9 | >99.9 |
| **20** | >99.9 | >99.9 | >99.9 | >99.9 | >99.9 |
| **21** | 99.2 | 97.0 | 97.2 | 99.7 | 97.6 |
| **22** | 98.9 | 95.3 | 99.8 | >99.9 | 99.7 |
| **23** | >99.9 | >99.9 | >99.9 | >99.9 | >99.9 |
| **24** | >99.9 | >99.9 | >99.9 | >99.9 | >99.9 |
| **25** | >99.9 | >99.9 | >99.9 | >99.9 | >99.9 |
| **26** | >99.9 | >99.9 | >99.9 | >99.9 | >99.9 |
| **27** | >99.9 | >99.9 | >99.9 | >99.9 | >99.9 |
| **28** | <0.1 | >99.9 | 99.5 | >99.9 | >99.9 |
| **29** | 98.0 | 98.3 | 99.7 | 79.6 | 35.3 |
| **30** | 0.5 | 99.7 | 16.6 | 2.9 | 8.8 |
| **31** | >99.9 | >99.9 | <0.1 | 0.3 | <0.1 |
| **32** | 88.3 | 9.1 | 0.3 | <0.1 | 3.1 |
| **33** | 98.5 | >99.9 | 86.3 | >99.9 | 99.8 |
| **Average** | **83.9** | **88.5** | **78.4** | **79.9** | **75.3** |

| | **d*J*-DP4 – 21 kJ/mol – RMSD 0.5 Å** | | | | |
|---|---|---|---|---|---|
| Comp. Nº | **AMBER** | **MM3** | **MMFF** | **OPLS2005** | **OPLS4** |
| **1** | >99.9 | >99.9 | >99.9 | >99.9 | >99.9 |
| **2** | >99.9 | >99.9 | >99.9 | >99.9 | >99.9 |
| **3** | 97.8 | >99.9 | 94.5 | 99.8 | >99.9 |
| **4** | >99.9 | >99.9 | >99.9 | 61.1 | >99.9 |
| **5** | >99.9 | >99.9 | >99.9 | 99.7 | >99.9 |
| **6** | 0.7 | 0.9 | 96.7 | 98.6 | 3.1 |
| **7** | 84.1 | 96.2 | >99.9 | 99.2 | 39.8 |
| **8** | >99.9 | 87.7 | 0.1 | 5.9 | 88.9 |
| **9** | 99.1 | 99.8 | <0.1 | >99.9 | 3.6 |
| **10** | >99.9 | >99.9 | >99.9 | >99.9 | >99.9 |
| **11** | 99.9 | 87.2 | 5.4 | >99.9 | <0.1 |
| **12** | 99.9 | >99.9 | 99.9 | 99.5 | 99.1 |
| **13** | <0.1 | 99.6 | >99.9 | >99.9 | >99.9 |
| **14** | >99.9 | >99.9 | >99.9 | >99.9 | >99.9 |
| **15** | 99.8 | >99.9 | 70.9 | >99.9 | >99.9 |
| **16** | <0.1 | 99.9 | <0.1 | <0.1 | 99.8 |
| **17** | >99.9 | 30.1 | 99.0 | <0.1 | >99.9 |
| **18** | >99.9 | 19.3 | 17.3 | >99.9 | >99.9 |
| **19** | >99.9 | >99.9 | >99.9 | >99.9 | >99.9 |
| **20** | >99.9 | >99.9 | >99.9 | >99.9 | >99.9 |
| **21** | 99.2 | 96.9 | 98.2 | 99.7 | 97.6 |
| **22** | 98.9 | 92.5 | 99.8 | >99.9 | 99.7 |
| **23** | >99.9 | >99.9 | >99.9 | >99.9 | >99.9 |
| **24** | >99.9 | >99.9 | >99.9 | >99.9 | >99.9 |
| **25** | >99.9 | >99.9 | >99.9 | >99.9 | >99.9 |
| **26** | >99.9 | >99.9 | >99.9 | >99.9 | >99.9 |
| **27** | >99.9 | >99.9 | 97.0 | >99.9 | >99.9 |
| **28** | <0.1 | >99.9 | >99.9 | >99.9 | >99.9 |
| **29** | 1.5 | 1.2 | 63.7 | 97.3 | <0.1 |
| **30** | 1.1 | 99.8 | 16.6 | 2.0 | 13.0 |
| **31** | >99.9 | >99.9 | <0.1 | 0.8 | <0.1 |
| **32** | 93.6 | 5.7 | 0.8 | <0.1 | 2.8 |
| **33** | 93.2 | >99.9 | 87.5 | >99.9 | 99.7 |
| **Average** | **80.9** | **85.4** | **74.2** | **80.7** | **77.2** |

| | *dJ*-DP4 – 21 kJ/mol – RMSD 1 Å | | | | |
|---|---|---|---|---|---|
| Comp. Nº | **AMBER** | **MM3** | **MMFF** | **OPLS2005** | **OPLS4** |
| **1** | >99.9 | >99.9 | >99.9 | >99.9 | >99.9 |
| **2** | >99.9 | >99.9 | >99.9 | >99.9 | >99.9 |
| **3** | 0.1 | 99.7 | 73.4 | >99.9 | >99.9 |
| **4** | >99.9 | >99.9 | >99.9 | 80.6 | >99.9 |
| **5** | >99.9 | >99.9 | >99.9 | 99.8 | >99.9 |
| **6** | 0.1 | 0.6 | 99.6 | 75.5 | 0.1 |
| **7** | 96.4 | 6.4 | 99.2 | <0.1 | 14.6 |
| **8** | >99.9 | >99.9 | 91.5 | >99.9 | <0.1 |
| **9** | >99.9 | >99.9 | 99.6 | >99.9 | 99.6 |
| **10** | >99.9 | >99.9 | >99.9 | >99.9 | >99.9 |
| **11** | 20.5 | >99.9 | <0.1 | >99.9 | <0.1 |
| **12** | 99.3 | >99.9 | >99.9 | 88.5 | 96.7 |
| **13** | 29.8 | 99.9 | >99.9 | 99.8 | 99.4 |
| **14** | >99.9 | >99.9 | 99.7 | >99.9 | 99.9 |
| **15** | 1.1 | 59.0 | 1.7 | 98.8 | 99.8 |
| **16** | <0.1 | >99.9 | <0.1 | <0.1 | <0.1 |
| **17** | 99.9 | 99.9 | >99.9 | <0.1 | >99.9 |
| **18** | >99.9 | >99.9 | 99.5 | >99.9 | >99.9 |
| **19** | >99.9 | >99.9 | >99.9 | >99.9 | >99.9 |
| **20** | >99.9 | >99.9 | >99.9 | >99.9 | >99.9 |
| **21** | 99.2 | >99.9 | 99.8 | >99.9 | 97.6 |
| **22** | 98.9 | 2.6 | 98.0 | >99.9 | 99.7 |
| **23** | >99.9 | >99.9 | >99.9 | >99.9 | >99.9 |
| **24** | >99.9 | >99.9 | >99.9 | >99.9 | >99.9 |
| **25** | >99.9 | >99.9 | >99.9 | >99.9 | >99.9 |
| **26** | >99.9 | 99.9 | >99.9 | >99.9 | >99.9 |
| **27** | >99.9 | 99.5 | >99.9 | >99.9 | >99.9 |
| **28** | 97.7 | >99.9 | 99.5 | >99.9 | >99.9 |
| **29** | 64.7 | 94.8 | 5.6 | 0.1 | 98.4 |
| **30** | 0.2 | >99.9 | 8.1 | <0.1 | 12.9 |
| **31** | >99.9 | 0.1 | <0.1 | >99.9 | <0.1 |
| **32** | 95.3 | 0.2 | <0.1 | 0.4 | 99.9 |
| **33** | 7.7 | <0.1 | 93.3 | 98.3 | 97.2 |
| **Average** | **76.1** | **80.7** | **77.8** | **80.1** | **79.3** |

**Table S39.** iJ/dJ-DP4 results for compounds **1**-**33**

| | **iJ/dJ-DP4 – 12 kJ/mol – MAD 05 Å** | | | | |
|---|---|---|---|---|---|
| Comp. Nº | **AMBER** | **MM3** | **MMFF** | **OPLS2005** | **OPLS4** |
| **1** | >99.9 | >99.9 | >99.9 | >99.9 | >99.9 |
| **2** | >99.9 | >99.9 | >99.9 | >99.9 | >99.9 |
| **3** | >99.9 | >99.9 | 99.9 | 80.8 | >99.9 |
| **4** | >99.9 | >99.9 | >99.9 | >99.9 | >99.9 |
| **5** | >99.9 | >99.9 | >99.9 | >99.9 | >99.9 |
| **6** | 83.2 | 11.9 | 99.5 | 99.8 | <0.1 |
| **7** | 79.9 | 96.2 | 98.7 | 0.3 | 9.5 |
| **8** | >99.9 | <0.1 | >99.9 | 3.7 | 76.6 |
| **9** | >99.9 | >99.9 | >99.9 | >99.9 | 37.6 |
| **10** | >99.9 | >99.9 | >99.9 | >99.9 | >99.9 |
| **11** | 98.7 | >99.9 | 65.7 | >99.9 | >99.9 |
| **12** | >99.9 | >99.9 | >99.9 | >99.9 | 99.1 |
| **13** | 99.8 | 99.5 | >99.9 | >99.9 | >99.9 |
| **14** | >99.9 | >99.9 | 99.9 | >99.9 | >99.9 |
| **15** | >99.9 | >99.9 | >99.9 | >99.9 | >99.9 |
| **16** | 99.4 | 57.9 | 99.0 | 0.2 | <0.1 |
| **17** | >99.9 | 95.4 | 98.9 | 4.2 | 99.8 |
| **18** | >99.9 | 99.9 | 99.9 | >99.9 | >99.9 |
| **19** | >99.9 | >99.9 | >99.9 | >99.9 | >99.9 |
| **20** | >99.9 | >99.9 | >99.9 | >99.9 | >99.9 |
| **21** | 99.2 | 97.0 | 97.2 | 99.7 | 97.6 |
| **22** | 99.0 | 95.4 | 99.8 | >99.9 | 99.7 |
| **23** | >99.9 | >99.9 | >99.9 | >99.9 | >99.9 |
| **24** | >99.9 | >99.9 | >99.9 | >99.9 | >99.9 |
| **25** | >99.9 | >99.9 | >99.9 | >99.9 | >99.9 |
| **26** | >99.9 | >99.9 | >99.9 | >99.9 | >99.9 |
| **27** | >99.9 | >99.9 | >99.9 | >99.9 | >99.9 |
| **28** | >99.9 | >99.9 | >99.9 | >99.9 | >99.9 |
| **29** | 97.8 | 99.7 | 90.4 | 95.8 | 94.2 |
| **30** | 0.3 | 99.9 | 95.0 | 20.6 | 72.9 |
| **31** | >99.9 | >99.9 | >99.9 | >99.9 | >99.9 |
| **32** | >99.9 | 98.3 | 87.1 | >99.9 | >99.9 |
| **33** | 97.7 | >99.9 | 13.4 | >99.9 | 97.6 |
| **Average** | **95.6** | **92.5** | **95.3** | **85.0** | **87.4** |

| | **i*J*/d*J*-DP4 – 21 kJ/mol – MAD 05 Å** | | | | |
|---|---|---|---|---|---|
| Comp. Nº | **AMBER** | **MM3** | **MMFF** | **OPLS2005** | **OPLS4** |
| **1** | >99.9 | >99.9 | >99.9 | >99.9 | >99.9 |
| **2** | >99.9 | >99.9 | >99.9 | >99.9 | >99.9 |
| **3** | >99.9 | >99.9 | >99.9 | 81.1 | >99.9 |
| **4** | >99.9 | >99.9 | >99.9 | >99.9 | >99.9 |
| **5** | >99.9 | >99.9 | >99.9 | >99.9 | >99.9 |
| **6** | 83.2 | 10.2 | 96.6 | 99.9 | <0.1 |
| **7** | 79.8 | 96.2 | 97.8 | 99.3 | 9.8 |
| **8** | 99.9 | 99.5 | >99.9 | 73.4 | 83.3 |
| **9** | >99.9 | >99.9 | >99.9 | >99.9 | 45.3 |
| **10** | >99.9 | >99.9 | >99.9 | >99.9 | >99.9 |
| **11** | >99.9 | >99.9 | 99.3 | >99.9 | >99.9 |
| **12** | >99.9 | >99.9 | >99.9 | 63.8 | 99.8 |
| **13** | >99.9 | 99.6 | >99.9 | >99.9 | >99.9 |
| **14** | >99.9 | >99.9 | 99.9 | >99.9 | >99.9 |
| **15** | >99.9 | >99.9 | >99.9 | >99.9 | >99.9 |
| **16** | 97.3 | 97.3 | 99.6 | 0.1 | 94.8 |
| **17** | 99.5 | 1.0 | 98.6 | <0.1 | 99.8 |
| **18** | >99.9 | 99.9 | 99.9 | 99.9 | 99.9 |
| **19** | >99.9 | >99.9 | >99.9 | >99.9 | >99.9 |
| **20** | >99.9 | >99.9 | >99.9 | >99.9 | >99.9 |
| **21** | 99.2 | 96.9 | 98.2 | 99.7 | 97.6 |
| **22** | 99.0 | 92.6 | 99.8 | >99.9 | 99.7 |
| **23** | >99.9 | >99.9 | >99.9 | >99.9 | >99.9 |
| **24** | >99.9 | >99.9 | >99.9 | >99.9 | >99.9 |
| **25** | >99.9 | >99.9 | >99.9 | >99.9 | >99.9 |
| **26** | >99.9 | >99.9 | >99.9 | >99.9 | >99.9 |
| **27** | >99.9 | >99.9 | >99.9 | >99.9 | >99.9 |
| **28** | >99.9 | >99.9 | >99.9 | >99.9 | >99.9 |
| **29** | 2.2 | 71.1 | 22.7 | 93.8 | 0.4 |
| **30** | 76.7 | 99.9 | 95.7 | 42.2 | 73.7 |
| **31** | >99.9 | >99.9 | >99.9 | >99.9 | >99.9 |
| **32** | >99.9 | 97.4 | <0.1 | >99.9 | 91.0 |
| **33** | 99.7 | >99.9 | 16.6 | >99.9 | 96.9 |
| **Average** | **95.0** | **92.8** | **91.7** | **89.5** | **87.6** |

| | **i*J*/d*J*-DP4 – 21 kJ/mol – MAD 1 Å** | | | | |
|---|---|---|---|---|---|
| Comp. Nº | **AMBER** | **MM3** | **MMFF** | **OPLS2005** | **OPLS4** |
| **1** | >99.9 | >99.9 | >99.9 | >99.9 | >99.9 |
| **2** | >99.9 | >99.9 | >99.9 | >99.9 | >99.9 |
| **3** | >99.9 | 99.9 | >99.9 | 81.1 | >99.9 |
| **4** | >99.9 | >99.9 | >99.9 | >99.9 | >99.9 |
| **5** | >99.9 | >99.9 | >99.9 | >99.9 | >99.9 |
| **6** | 83.2 | 10.2 | 95.5 | 99.9 | <0.1 |
| **7** | 79.8 | 96.2 | 99.2 | 99.3 | 9.8 |
| **8** | 99.9 | 99.6 | >99.9 | 2.5 | 83.0 |
| **9** | >99.9 | 91.4 | >99.9 | >99.9 | 33.6 |
| **10** | >99.9 | >99.9 | >99.9 | >99.9 | >99.9 |
| **11** | >99.9 | >99.9 | 83.4 | >99.9 | 80.5 |
| **12** | >99.9 | >99.9 | >99.9 | >99.9 | 99.9 |
| **13** | >99.9 | 99.6 | >99.9 | >99.9 | >99.9 |
| **14** | >99.9 | >99.9 | >99.9 | >99.9 | >99.9 |
| **15** | 69.0 | 6.6 | >99.9 | >99.9 | >99.9 |
| **16** | 19.8 | 6.6 | 89.1 | 0.1 | 26.5 |
| **17** | 99.8 | 5.1 | >99.9 | <0.1 | >99.9 |
| **18** | >99.9 | 99.9 | 99.9 | >99.9 | >99.9 |
| **19** | >99.9 | >99.9 | >99.9 | >99.9 | >99.9 |
| **20** | >99.9 | >99.9 | >99.9 | >99.9 | >99.9 |
| **21** | 99.2 | 96.9 | 98.2 | 99.7 | 97.6 |
| **22** | 99.0 | 92.6 | 99.8 | >99.9 | 99.7 |
| **23** | >99.9 | >99.9 | >99.9 | >99.9 | >99.9 |
| **24** | >99.9 | >99.9 | >99.9 | >99.9 | >99.9 |
| **25** | >99.9 | >99.9 | >99.9 | >99.9 | >99.9 |
| **26** | >99.9 | >99.9 | >99.9 | >99.9 | >99.9 |
| **27** | >99.9 | >99.9 | >99.9 | >99.9 | >99.9 |
| **28** | >99.9 | >99.9 | >99.9 | >99.9 | >99.9 |
| **29** | 6.9 | 66.0 | 26.2 | 98.8 | 22.4 |
| **30** | 47.8 | 99.6 | 96.0 | 38.8 | 18.2 |
| **31** | >99.9 | >99.9 | >99.9 | >99.9 | >99.9 |
| **32** | >99.9 | 77.3 | <0.1 | >99.9 | 91.0 |
| **33** | 99.8 | >99.9 | 16.4 | >99.9 | 99.5 |
| **Average** | **91.0** | **86.3** | **91.0** | **88.5** | **83.7** |

| Comp. № | *iJ/dJ*-DP4 – 12 kJ/mol – RMSD 0.5 Å | | | | |
|---|---|---|---|---|---|
| | **AMBER** | **MM3** | **MMFF** | **OPLS2005** | **OPLS4** |
| **1** | >99.9 | >99.9 | >99.9 | >99.9 | >99.9 |
| **2** | >99.9 | >99.9 | >99.9 | >99.9 | >99.9 |
| **3** | >99.9 | 99.9 | >99.9 | 87.4 | >99.9 |
| **4** | >99.9 | >99.9 | >99.9 | 59.3 | >99.9 |
| **5** | >99.9 | >99.9 | >99.9 | >99.9 | >99.9 |
| **6** | 15.5 | 12.0 | 99.2 | 99.8 | <0.1 |
| **7** | 90.9 | 96.2 | 99.6 | 0.3 | 9.5 |
| **8** | >99.9 | <0.1 | 98.5 | 9.0 | 97.4 |
| **9** | 99.3 | >99.9 | 90.3 | >99.9 | 1.9 |
| **10** | >99.9 | >99.9 | >99.9 | >99.9 | >99.9 |
| **11** | 77.7 | >99.9 | >99.9 | >99.9 | >99.9 |
| **12** | >99.9 | >99.9 | >99.9 | >99.9 | 99.9 |
| **13** | 99.7 | 99.7 | >99.9 | >99.9 | >99.9 |
| **14** | >99.9 | >99.9 | >99.9 | >99.9 | >99.9 |
| **15** | >99.9 | >99.9 | 99.4 | 0.1 | >99.9 |
| **16** | >99.9 | 0.1 | 8.5 | >99.9 | <0.1 |
| **17** | >99.9 | >99.9 | >99.9 | 77.8 | >99.9 |
| **18** | 98.9 | 99.8 | 99.9 | 99.3 | 99.9 |
| **19** | >99.9 | >99.9 | >99.9 | >99.9 | >99.9 |
| **20** | >99.9 | >99.9 | >99.9 | >99.9 | >99.9 |
| **21** | 99.2 | 97.0 | 97.2 | 99.7 | 97.6 |
| **22** | 99.0 | 95.4 | 99.8 | >99.9 | 99.7 |
| **23** | >99.9 | >99.9 | >99.9 | >99.9 | >99.9 |
| **24** | >99.9 | >99.9 | >99.9 | >99.9 | >99.9 |
| **25** | >99.9 | >99.9 | >99.9 | >99.9 | >99.9 |
| **26** | >99.9 | >99.9 | >99.9 | >99.9 | >99.9 |
| **27** | >99.9 | >99.9 | >99.9 | >99.9 | >99.9 |
| **28** | >99.9 | >99.9 | >99.9 | >99.9 | >99.9 |
| **29** | 89.2 | 97.2 | 98.4 | 84.7 | 99.2 |
| **30** | 0.5 | 99.2 | 19.8 | 0.1 | 9.2 |
| **31** | >99.9 | >99.9 | >99.9 | >99.9 | >99.9 |
| **32** | >99.9 | 99.8 | 0.7 | >99.9 | >99.9 |
| **33** | 98.2 | >99.9 | 85.0 | >99.9 | 99.7 |
| **Average** | **93.0** | **90.8** | **90.8** | **85.4** | **85.3** |

| Comp. Nº | *iJ/dJ*-DP4 – 21 kJ/mol – RMSD 0.5 Å | | | | |
|---|---|---|---|---|---|
| | **AMBER** | **MM3** | **MMFF** | **OPLS2005** | **OPLS4** |
| **1** | >99.9 | >99.9 | >99.9 | >99.9 | >99.9 |
| **2** | >99.9 | >99.9 | >99.9 | >99.9 | >99.9 |
| **3** | >99.9 | 99.9 | >99.9 | 87.7 | >99.9 |
| **4** | >99.9 | >99.9 | >99.9 | 61.1 | >99.9 |
| **5** | >99.9 | >99.9 | >99.9 | >99.9 | >99.9 |
| **6** | 15.5 | 10.2 | 94.9 | 99.9 | <0.1 |
| **7** | 84.1 | 96.2 | 99.2 | 99.3 | 9.8 |
| **8** | >99.9 | 68.6 | 95.1 | 99.8 | 99.5 |
| **9** | 99.4 | >99.9 | 99.7 | >99.9 | 2.2 |
| **10** | >99.9 | >99.9 | >99.9 | >99.9 | >99.9 |
| **11** | >99.9 | >99.9 | >99.9 | >99.9 | >99.9 |
| **12** | >99.9 | >99.9 | >99.9 | >99.9 | >99.9 |
| **13** | >99.9 | 99.7 | >99.9 | >99.9 | >99.9 |
| **14** | >99.9 | 99.9 | 99.9 | >99.9 | >99.9 |
| **15** | >99.9 | >99.9 | 99.0 | 0.1 | >99.9 |
| **16** | >99.9 | 7.5 | 7.9 | 97.8 | 26.6 |
| **17** | >99.9 | 98.4 | >99.9 | <0.1 | >99.9 |
| **18** | 99.4 | 99.8 | >99.9 | 99.1 | 98.0 |
| **19** | >99.9 | >99.9 | >99.9 | >99.9 | >99.9 |
| **20** | >99.9 | >99.9 | >99.9 | >99.9 | >99.9 |
| **21** | 99.2 | 96.9 | 98.2 | 99.7 | 97.6 |
| **22** | 99.0 | 92.6 | 99.8 | >99.9 | 99.7 |
| **23** | >99.9 | >99.9 | >99.9 | >99.9 | >99.9 |
| **24** | >99.9 | >99.9 | >99.9 | >99.9 | >99.9 |
| **25** | >99.9 | >99.9 | >99.9 | >99.9 | >99.9 |
| **26** | >99.9 | >99.9 | >99.9 | >99.9 | >99.9 |
| **27** | >99.9 | >99.9 | >99.9 | >99.9 | >99.9 |
| **28** | 0.8 | >99.9 | >99.9 | >99.9 | >99.9 |
| **29** | 1.4 | 0.9 | 61.2 | 95.4 | 0.1 |
| **30** | 0.4 | 99.4 | 19.9 | 0.1 | 10.1 |
| **31** | >99.9 | >99.9 | 52.5 | >99.9 | >99.9 |
| **32** | >99.9 | 99.7 | <0.1 | 98.5 | 99.9 |
| **33** | 98.3 | >99.9 | 86.3 | >99.9 | 99.7 |
| **Average** | **87.8** | **90.0** | **88.3** | **89.0** | **83.1** |

| Comp. № | i*J*/d*J*-DP4 – 21 kJ/mol – RMSD 1 Å | | | | |
|---|---|---|---|---|---|
| | **AMBER** | **MM3** | **MMFF** | **OPLS2005** | **OPLS4** |
| **1** | >99.9 | >99.9 | >99.9 | >99.9 | >99.9 |
| **2** | >99.9 | >99.9 | >99.9 | >99.9 | >99.9 |
| **3** | <0.1 | >99.9 | >99.9 | 92.9 | >99.9 |
| **4** | >99.9 | >99.9 | >99.9 | 80.7 | >99.9 |
| **5** | >99.9 | >99.9 | >99.9 | >99.9 | >99.9 |
| **6** | 1.6 | 5.1 | 99.0 | 97.3 | 0.2 |
| **7** | 96.4 | 6.4 | 99.2 | 1.4 | 98.2 |
| **8** | >99.9 | >99.9 | 87.5 | >99.9 | 5.0 |
| **9** | >99.9 | 92.3 | 98.6 | >99.9 | 93.3 |
| **10** | >99.9 | >99.9 | >99.9 | >99.9 | >99.9 |
| **11** | 23.4 | 99.9 | >99.9 | >99.9 | 89.6 |
| **12** | 75.7 | >99.9 | >99.9 | <0.1 | >99.9 |
| **13** | 93.0 | 99.9 | >99.9 | >99.9 | 99.7 |
| **14** | >99.9 | >99.9 | 99.4 | >99.9 | 99.9 |
| **15** | 0.5 | 60.1 | 1.8 | <0.1 | 99.9 |
| **16** | 96.0 | <0.1 | >99.9 | <0.1 | 12.5 |
| **17** | 80.3 | 98.1 | >99.9 | <0.1 | >99.9 |
| **18** | >99.9 | >99.9 | >99.9 | >99.9 | >99.9 |
| **19** | >99.9 | >99.9 | >99.9 | >99.9 | >99.9 |
| **20** | >99.9 | >99.9 | >99.9 | >99.9 | >99.9 |
| **21** | 99.2 | >99.9 | 99.8 | >99.9 | 97.6 |
| **22** | 99.0 | 3.2 | 98.3 | >99.9 | 99.7 |
| **23** | >99.9 | >99.9 | >99.9 | >99.9 | >99.9 |
| **24** | >99.9 | >99.9 | >99.9 | >99.9 | >99.9 |
| **25** | >99.9 | >99.9 | >99.9 | >99.9 | >99.9 |
| **26** | >99.9 | <0.1 | >99.9 | >99.9 | >99.9 |
| **27** | >99.9 | >99.9 | >99.9 | >99.9 | >99.9 |
| **28** | 99.6 | >99.9 | 99.9 | >99.9 | >99.9 |
| **29** | 75.8 | 90.6 | 77.4 | 0.2 | 99.8 |
| **30** | <0.1 | 99.9 | 9.3 | <0.1 | <0.1 |
| **31** | >99.9 | >99.9 | <0.1 | >99.9 | >99.9 |
| **32** | >99.9 | >99.9 | 83.2 | >99.9 | >99.9 |
| **33** | <0.1 | 0.1 | 92.6 | 99.8 | 15.3 |
| **Average** | **80.0** | **80.5** | **89.3** | **77.9** | **85.2** |

**Table S40.** Dependence of DP4 and J-DP4 on the energy cutoff used during the conformational search for selected examples.

| Nº | C.Search | E. Cutoff | DP4 | dJ-DP4 | iJ/dJ-DP4 |
|---|---|---|---|---|---|
| **7** | OPLS2005 | 12 | 2.9 | 0.1 | 0.3 |
| | MAD 05 | 21 | 90.1 | 99.2 | 99.3 |
| **8** | AMBER | 12 | <0.1 | >99.9 | >99.9 |
| | RMS 05 | 21 | 96.0 | >99.9 | >99.9 |
| **11** | MMFF | 12 | 99.6 | >99.9 | >99.9 |
| | RMS 05 | 21 | 15.8 | 5.4 | >99.9 |
| **16** | AMBER | 12 | 11.5 | 0.1 | 99.4 |
| | MAD 05 | 21 | 99.9 | 99.0 | 97.3 |
| **27** | MMFF | 12 | >99.9 | >99.9 | >99.9 |
| | RMS 05 | 21 | 0.9 | 97.0 | >99.9 |
| **28** | MMFF | 12 | 3.1 | 99.4 | >99.9 |
| | RMS 05 | 21 | >99.9 | >99.9 | >99.9 |

**Table S41.** Results for Mix-J-DP4.

| | iJ/dJ-DP4 – 12 kJ/mol – MAD 05 Å (Mix-J-DP4) | | | |
|---|---|---|---|---|
| Comp. Nº | **AMBER** | **MM3** | **MMFF** | **Mix-*J*-DP4** |
| **1** | >99.9 | >99.9 | >99.9 | >99.9 |
| **2** | >99.9 | >99.9 | >99.9 | >99.9 |
| **3** | >99.9 | >99.9 | 99.9 | >99.9 |
| **4** | >99.9 | >99.9 | >99.9 | >99.9 |
| **5** | >99.9 | >99.9 | >99.9 | >99.9 |
| **6** | 83.2 | **11.9** | 99.5 | 64.9 |
| **7** | 79.9 | 96.2 | 98.7 | 91.6 |
| **8** | >99.9 | **<0.1** | >99.9 | 66.7 |
| **9** | >99.9 | >99.9 | >99.9 | >99.9 |
| **10** | >99.9 | >99.9 | >99.9 | >99.9 |
| **11** | 98.7 | >99.9 | 65.7 | 88.1 |
| **12** | >99.9 | >99.9 | >99.9 | >99.9 |
| **13** | 99.8 | 99.5 | >99.9 | 99.8 |
| **14** | >99.9 | >99.9 | 99.9 | 99.9 |
| **15** | >99.9 | >99.9 | >99.9 | >99.9 |
| **16** | 99.4 | 57.9 | 99.0 | 85.4 |
| **17** | >99.9 | 95.4 | 98.9 | 98.1 |
| **18** | >99.9 | 99.9 | 99.9 | 99.9 |
| **19** | >99.9 | >99.9 | >99.9 | >99.9 |
| **20** | >99.9 | >99.9 | >99.9 | >99.9 |
| **21** | 99.2 | 97.0 | 97.2 | 97.8 |
| **22** | 99.0 | 95.4 | 99.8 | 98.1 |
| **23** | >99.9 | >99.9 | >99.9 | >99.9 |
| **24** | >99.9 | >99.9 | >99.9 | >99.9 |
| **25** | >99.9 | >99.9 | >99.9 | >99.9 |
| **26** | >99.9 | >99.9 | >99.9 | >99.9 |
| **27** | >99.9 | >99.9 | >99.9 | >99.9 |
| **28** | >99.9 | >99.9 | >99.9 | >99.9 |
| **29** | 97.8 | 99.7 | 90.4 | 96.0 |
| **30** | **0.3** | 99.9 | 95.0 | 65.1 |
| **31** | >99.9 | >99.9 | >99.9 | >99.9 |
| **32** | >99.9 | 98.3 | 87.1 | 95.1 |
| **33** | 97.7 | >99.9 | **13.4** | 70.4 |
| **Average** | **95.6** | **92.5** | **95.3** | **94.4** |
| **Nº fails** | **1** | **2** | **1** | **0** |

## 7. Summary of general results

**Table S42.** Average number of conformers found for DP4 and dJ-DP4 approaches.

| | 21kJ | | | | 12kJ | |
|---|---|---|---|---|---|---|
| | MAD-05 | RMS-05 | MAD-1 | RMS-1 | MAD-05 | RMS-05 |
| **AMBER** | 100.9 | 32.0 | 76.6 | 7.5 | 42.2 | 12.7 |
| **MM3** | 63.0 | 25.4 | 45.3 | 6.7 | 29.4 | 11.5 |
| **MMFF** | 83.1 | 26.6 | 62.0 | 6.5 | 36.1 | 11.2 |
| **OPLS2005** | 68.2 | 25.9 | 56.3 | 7.1 | 30.7 | 11.1 |
| **OPLS4** | 81.2 | 28.7 | 56.6 | 7.3 | 35.9 | 11.1 |

**Table S43.** Average number of conformers found for iJ/dJ-DP4 approach.

| | 21kJ | | | | 12kJ | |
|---|---|---|---|---|---|---|
| | MAD-05 | RMS-05 | MAD-1 | RMS-1 | MAD-05 | RMS-05 |
| **AMBER** | 24.3 | 6.6 | 16.4 | 1.8 | 13.5 | 3.4 |
| **MM3** | 16.2 | 5.7 | 10.8 | 1.6 | 9.3 | 2.9 |
| **MMFF** | 21.0 | 6.3 | 15.1 | 1.7 | 10.9 | 3.0 |
| **OPLS2005** | 14.3 | 4.3 | 9.9 | 1.3 | 7.9 | 2.1 |
| **OPLS4** | 19.8 | 6.1 | 12.6 | 1.8 | 11.2 | 2.6 |

**Table S44.** Relative number of conformers found for DP4 and dJ-DP4 approaches.

| | 21kJ | | | | 12kJ | |
|---|---|---|---|---|---|---|
| | MAD-05 | RMS-05 | MAD-1 | RMS-1 | MAD-05 | RMS-05 |
| **AMBER** | 100.0 | 33.4 | 79.4 | 8.0 | 38.4 | 13.1 |
| **MM3** | 100.0 | 43.4 | 76.3 | 11.9 | 43.4 | 19.2 |
| **MMFF** | 100.0 | 33.7 | 76.9 | 8.4 | 40.6 | 14.2 |
| **OPLS2005** | 100.0 | 41.1 | 87.0 | 11.5 | 42.2 | 17.5 |
| **OPLS4** | 100.0 | 37.2 | 72.5 | 9.8 | 40.5 | 14.3 |

**Table S45.** Relative number of conformers found for iJ/dJ-DP4 approach.

| | 21kJ | | | | 12kJ | |
|---|---|---|---|---|---|---|
| | MAD-05 | RMS-05 | MAD-1 | RMS-1 | MAD-05 | RMS-05 |
| **AMBER** | 100.0 | 29.3 | 71.1 | 8.2 | 52.1 | 14.8 |
| **MM3** | 100.0 | 39.4 | 73.6 | 11.8 | 52.9 | 19.7 |
| **MMFF** | 100.0 | 33.2 | 75.3 | 9.6 | 47.2 | 16.0 |
| **OPLS2005** | 100.0 | 34.9 | 76.2 | 10.8 | 51.1 | 17.0 |
| **OPLS4** | 100.0 | 34.4 | 70.6 | 10.9 | 49.7 | 14.5 |

**Table S46.** Overall probability for each Force Field

| | DP4 | dJ-DP4 | iJ/dJ-DP4 |
|---|---|---|---|
| **AMBER** | 65.4 | 83.3 | 90.4 |
| **MM3** | 66.8 | 86.9 | 88.8 |
| **MMFF** | 68.4 | 80.8 | 91.1 |
| **OPLS2005** | 66.9 | 82.0 | 85.4 |
| **OPLS4** | 63.5 | 79.1 | 85.3 |

**Table S47.** Overall probability for each geometric criterion

| | DP4 | dJ-DP4 | iJ/dJ-DP4 |
|---|---|---|---|
| **MAD-05 Å** | 71.5 | 85.6 | 91.2 |
| **RMS-05 Å** | 63.9 | 80.4 | 88.3 |
| **MAD-1 Å** | 70.9 | 83.7 | 88.1 |
| **RMS-1 Å** | 55.4 | 78.8 | 82.0 |

**Table S48.** Overall probability for each energy cutoff

| | DP4 | dJ-DP4 | iJ/dJ-DP4 |
|---|---|---|---|
| **12 kJ/mol** | 67.45 | 83.57 | 90.10 |
| **21 kJ/mol** | 65.58 | 81.82 | 87.25 |

**Table S49.** Overall probability for each Force Field at 21 kJ/mol and MAD 0.5 Å

| | DP4 | dJ-DP4 | iJ/dJ-DP4 |
|---|---|---|---|
| **AMBER** | 74.4 | 87.1 | 94.6 |
| **MM3** | 70.8 | 88.2 | 92.1 |
| **MMFF** | 78.3 | 88.2 | 96.9 |
| **OPLS2005** | 72.2 | 92.3 | 88.4 |
| **OPLS4** | 69.9 | 81.3 | 86.8 |

**Table S50.** Overall probability for each Force Field at 21 kJ/mol and RMSD 0.5 Å

| | DP4 | dJ-DP4 | iJ/dJ-DP4 |
|---|---|---|---|
| **AMBER** | 62.4 | 80.1 | 89.9 |
| **MM3** | 68.1 | 87.4 | 88.7 |
| **MMFF** | 68.4 | 80.8 | 90.9 |
| **OPLS2005** | 66.2 | 88.3 | 88.0 |
| **OPLS4** | 64.6 | 83.8 | 81.5 |

**Table S51.** Overall probability for each Force Field at 21 kJ/mol and MAD 1 Å

| | DP4 | dJ-DP4 | iJ/dJ-DP4 |
|---|---|---|---|
| **AMBER** | 72.4 | 83.2 | 90.1 |
| **MM3** | 69.2 | 84.6 | 85.7 |
| **MMFF** | 78.0 | 87.6 | 96.2 |
| **OPLS2005** | 72.3 | 89.5 | 87.3 |
| **OPLS4** | 68.6 | 84.7 | 82.4 |

**Table S52.** Overall probability for each Force Field at 21 kJ/mol and RMSD 1 Å

| | DP4 | dJ-DP4 | iJ/dJ-DP4 |
|---|---|---|---|
| **AMBER** | 57.6 | 77.7 | 81.4 |
| **MM3** | 58.7 | 85.9 | 82.3 |
| **MMFF** | 69.0 | 85.1 | 89.0 |
| **OPLS2005** | 53.1 | 84.2 | 75.7 |
| **OPLS4** | 55.0 | 83.0 | 86.1 |

**Table S53.** Overall probability for each Force Field at 12 kJ/mol and MAD 0.5 Å

| | DP4 | dJ-DP4 | iJ/dJ-DP4 |
|---|---|---|---|
| **AMBER** | 67.6 | 88.5 | 95.2 |
| **MM3** | 73.7 | 92.4 | 91.8 |
| **MMFF** | 78.0 | 90.7 | 98.1 |
| **OPLS2005** | 71.4 | 85.8 | 83.5 |
| **OPLS4** | 70.2 | 84.5 | 86.2 |

**Table S54.** Overall probability for each Force Field at 12 kJ/mol and RMSD 0.5 Å

| | DP4 | dJ-DP4 | iJ/dJ-DP4 |
|---|---|---|---|
| **AMBER** | 63.3 | 83.3 | 92.3 |
| **MM3** | 67.8 | 90.7 | 89.9 |
| **MMFF** | 66.6 | 80.6 | 93.7 |
| **OPLS2005** | 65.5 | 87.4 | 83.9 |
| **OPLS4** | 62.7 | 81.8 | 83.8 |

**8. Zoarenone and Zoaramine as examples**

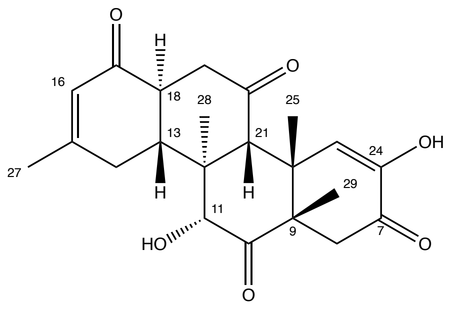


Zoarenone (**34**)

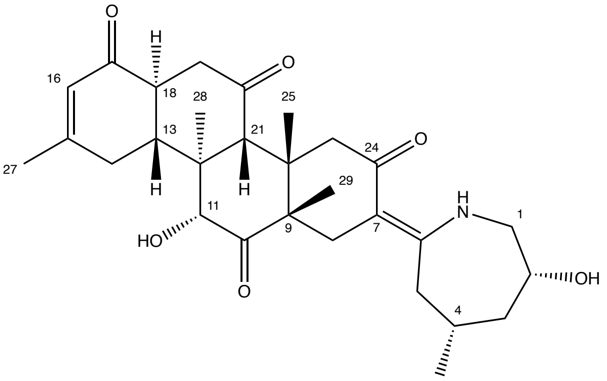


Zoaramine (**35**)

**Table S55.** $^1$H and $^{13}$C NMR data

| | Zoarenone (**34**) | | | Zoaramine (**35**) | | |
|---|---|---|---|---|---|---|
| № | $\delta_C$ | $\delta_H$ | *J* (Hz) | $\delta_C$ | $\delta_H$ | *J* (Hz) |
| 1a | | | | 49.9 | 3.42 | |
| 1b | | | | | 3.42 | |
| 2 | | | | 69.6 | 3.68 | 10.7, 2.0 |
| 3a | | | | 48.1 | 1.41 | 12.0, 10.7 |
| 3b | | | | | 2.15 | 2.0, 2.0 |
| 4 | | | | | 1.70 | 12.0, 10.1, 2.0, 1.5 |
| 5a | | | | 29.1 | 2.29 | 10.1 |
| 5b | | | | 35.1 | 2.60 | 1.5 |
| 6 | | | | 167.3 | | |
| 7 | 191.5 | | | 95.3 | | |
| 8a | 40.6 | 2.88 | | 32.1 | 3.03 | |
| 8b | | 2.51 | | | 2.09 | |
| 9 | 52.9 | | | 50.8 | | |
| 10 | 211.7 | | | 212.2 | | |
| 11 | 77.7 | 4.52 | | 76.8 | 4.53 | |
| 12 | 52.1 | | | 51.6 | | |
| 13 | 52.0 | 2.47 | 12.3, 11.0, 3.5 | 53.9 | 2.47 | 11.8, 11.0, 5.3 |
| 14a | 33.4 | 2.39 | 3.5 | 33.3 | 2.45 | 11.0 |
| 14b | | 2.90 | 11.0 | | 2.70 | 5.3 |
| 15 | 162.5 | | | 162.2 | | |
| 16 | 124.7 | 5.93 | | 125 | 5.92 | |
| 17 | 197.8 | | | 198.5 | | |
| 18 | 45.6 | 2.55 | 12.5, 12.3, 5.1 | 45.3 | 2.55 | 12.4, 11.8, 5.4 |
| 19a | 43.2 | 2.92 | 5.1 | 42.9 | 2.81 | 5.4 |
| 19b | | 2.46 | 12.5 | | 2.46 | 12.4 |
| 20 | 207.6 | | | 207.6 | | |
| 21 | 58.8 | 3.09 | | 60.6 | 3.00 | |
| 22 | 43.8 | | | 41.7 | | |
| 23a | 120.7 | 6.74 | | 43.7 | 2.24 | |
| 23b | | | | | 3.09 | |
| 24 | 144.5 | | | 191.4 | | |
| 25 | 21.7 | 1.40 | | 21.0 | 1.17 | |
| 27 | 24.6 | 2.01 | | 24.7 | 2.02 | |
| 28 | 10.7 | 0.81 | | 13.0 | 0.87 | |
| 29 | 21.0 | 1.32 | | 23.4 | 1.32 | |
| 30 | | | | 24.7 | 1.13 | |

**Table S56.** Studied isomers for Zoarenone

| Isomer | Configuration |
|---|---|
| **Isomer 1** | 9R, 11R, 12R, 13R, 18S, 21R, 22R |
| **Isomer 2** | 9S, 11R, 12R, 13R, 18S, 21R, 22R |
| **Isomer 3** | 9R, 11S, 12R, 13R, 18S, 21R, 22R |
| **Isomer 4** | 9S, 11S, 12R, 13R, 18S, 21R, 22R |
| **Isomer 5** | 9R, 11R, 12S, 13R, 18S, 21R, 22R |
| **Isomer 6** | 9S, 11R, 12S, 13R, 18S, 21R, 22R |
| **Isomer 7** | 9R, 11S, 12S, 13R, 18S, 21R, 22R |
| **Isomer 8** | 9S, 11S, 12S, 13R, 18S, 21R, 22R |
| **Isomer 9** | 9R, 11R, 12R, 13R, 18S, 21S, 22R |
| **Isomer 10** | 9S, 11R, 12R, 13R, 18S, 21S, 22R |
| **Isomer 11** | 9R, 11S, 12R, 13R, 18S, 21S, 22R |
| **Isomer 12** | 9S, 11S, 12R, 13R, 18S, 21S, 22R |
| **Isomer 13** | 9R, 11R, 12S, 13R, 18S, 21S, 22R |
| **Isomer 14** | 9S, 11R, 12S, 13R, 18S, 21S, 22R |
| **Isomer 15** | 9R, 11S, 12S, 13R, 18S, 21S, 22R |
| **Isomer 16** | 9S, 11S, 12S, 13R, 18S, 21S, 22R |
| **Isomer 17** | 9R, 11R, 12R, 13R, 18S, 21R, 22S |
| **Isomer 18** | 9S, 11R, 12R, 13R, 18S, 21R, 22S |
| **Isomer 19** | 9R, 11S, 12R, 13R, 18S, 21R, 22S |
| **Isomer 20** | 9S, 11S, 12R, 13R, 18S, 21R, 22S |
| **Isomer 21** | 9R, 11R, 12S, 13R, 18S, 21R, 22S |
| **Isomer 22** | 9S, 11R, 12S, 13R, 18S, 21R, 22S |
| **Isomer 23** | 9R, 11S, 12S, 13R, 18S, 21R, 22S |
| **Isomer 24** | 9S, 11S, 12S, 13R, 18S, 21R, 22S |
| **Isomer 25** | 9R, 11R, 12R, 13R, 18S, 21S, 22S |
| **Isomer 26** | 9S, 11R, 12R, 13R, 18S, 21S, 22S |
| **Isomer 27** | 9R, 11S, 12R, 13R, 18S, 21S, 22S |
| **Isomer 28** | 9S, 11S, 12R, 13R, 18S, 21S, 22S |
| **Isomer 29** | 9R, 11R, 12S, 13R, 18S, 21S, 22S |
| **Isomer 30** | 9S, 11R, 12S, 13R, 18S, 21S, 22S |
| **Isomer 31** | 9R, 11S, 12S, 13R, 18S, 21S, 22S |
| **Isomer 32** | 9S, 11S, 12S, 13R, 18S, 21S, 22S |
| **Isomer 33** | 9R, 11R, 12R, 13S, 18S, 21R, 22R |
| **Isomer 34** | 9S, 11R, 12R, 13S, 18S, 21R, 22R |
| **Isomer 35** | 9R, 11S, 12R, 13S, 18S, 21R, 22R |
| **Isomer 36** | 9S, 11S, 12R, 13S, 18S, 21R, 22R |
| **Isomer 37** | 9R, 11R, 12S, 13S, 18S, 21R, 22R |
| **Isomer 38** | 9S, 11R, 12S, 13S, 18S, 21R, 22R |
| **Isomer 39** | 9R, 11S, 12S, 13S, 18S, 21R, 22R |
| **Isomer 40** | 9S, 11S, 12S, 13S, 18S, 21R, 22R |
| **Isomer 41** | 9R, 11R, 12R, 13S, 18S, 21S, 22R |
| **Isomer 42** | 9S, 11R, 12R, 13S, 18S, 21S, 22R |
| **Isomer 43** | 9R, 11S, 12R, 13S, 18S, 21S, 22R |
| **Isomer 44** | 9S, 11S, 12R, 13S, 18S, 21S, 22R |
| **Isomer 45** | 9R, 11R, 12S, 13S, 18S, 21S, 22R |
| **Isomer 46** | 9S, 11R, 12S, 13S, 18S, 21S, 22R |
| **Isomer 47** | 9R, 11S, 12S, 13S, 18S, 21S, 22R |
| **Isomer 48** | 9S, 11S, 12S, 13S, 18S, 21S, 22R |
| **Isomer 49** | 9R, 11R, 12R, 13S, 18S, 21R, 22S |
| **Isomer 50** | 9S, 11R, 12R, 13S, 18S, 21R, 22S |
| **Isomer 51** | 9R, 11S, 12R, 13S, 18S, 21R, 22S |
| **Isomer 52** | 9S, 11S, 12R, 13S, 18S, 21R, 22S |
| **Isomer 53** | 9R, 11R, 12S, 13S, 18S, 21R, 22S |
| **Isomer 54** | 9S, 11R, 12S, 13S, 18S, 21R, 22S |
| **Isomer 55** | 9R, 11S, 12S, 13S, 18S, 21R, 22S |
| **Isomer 56** | 9S, 11S, 12S, 13S, 18S, 21R, 22S |
| **Isomer 57** | 9R, 11R, 12R, 13S, 18S, 21S, 22S |
| **Isomer 58** | 9S, 11R, 12R, 13S, 18S, 21S, 22S |
| **Isomer 59** | 9R, 11S, 12R, 13S, 18S, 21S, 22S |

| | |
|---|---|
| **Isomer 60** | 9S, 11S, 12R, 13S, 18S, 21S, 22S |
| **Isomer 61** | 9R, 11R, 12S, 13S, 18S, 21S, 22S |
| **Isomer 62** | 9S, 11R, 12S, 13S, 18S, 21S, 22S |
| **Isomer 63** | 9R, 11S, 12S, 13S, 18S, 21S, 22S |
| **Isomer 64** | 9S, 11S, 12S, 13S, 18S, 21S, 22S |

**Table S57.** Studied isomers for Zoaramine

| Isomer | Configuration |
|---|---|
| **Isomer 1** | 2R, 4R, 9S, 11R, 12S, 13R, 18S, 21R, 22S |
| **Isomer 2** | 2R, 4R, 9S, 11S, 12S, 13R, 18S, 21R, 22S |
| **Isomer 3** | 2S, 4R, 9S, 11R, 12S, 13R, 18S, 21R, 22S |
| **Isomer 4** | 2S, 4R, 9S, 11S, 12S, 13R, 18S, 21R, 22S |
| **Isomer 5** | 2R, 4S, 9S, 11R, 12S, 13R, 18S, 21R, 22S |
| **Isomer 6** | 2R, 4S, 9S, 11S, 12S, 13R, 18S, 21R, 22S |
| **Isomer 7** | 2S, 4S, 9S, 11R, 12S, 13R, 18S, 21R, 22S |
| **Isomer 8** | 2S, 4S, 9S, 11S, 12S, 13R, 18S, 21R, 22S |

**Table S58.** Studied Boltzmann averaged GIAO isotropic magnetic shielding constants (σ) and coupling constants of isomers calculated at the PCM/B3LYP/6-31G**//AMBER level of theory after elimination of conformations incompatible with experimental $^{3}J_{HH}$ of Zoarenone according to i*J*/d*J*-DP4.

| Isotropic shielding constants | | | | | | | | |
|---|---|---|---|---|---|---|---|---|
| Nuclei | Isomer 1 | Isomer 2 | Isomer 3 | Isomer 4 | Isomer 5 | Isomer 6 | Isomer 7 | Isomer 8 |
| C 7 | 7.17 | 6.87 | 7.41 | 7.80 | 10.17 | 6.96 | 8.74 | 6.32 |
| C 8 | 145.52 | 148.01 | 145.89 | 146.55 | 147.79 | 147.39 | 145.92 | 146.96 |
| C 9 | 136.93 | 134.64 | 136.95 | 136.10 | 135.30 | 132.69 | 135.75 | 132.78 |
| C 10 | -17.21 | -22.06 | -20.93 | -23.13 | -19.44 | -20.51 | -16.01 | -17.52 |
| C 11 | 113.32 | 113.80 | 121.11 | 119.67 | 116.00 | 116.64 | 114.76 | 114.64 |
| C 12 | 141.90 | 145.95 | 137.12 | 148.29 | 140.14 | 139.24 | 143.09 | 142.81 |
| C 13 | 142.78 | 148.88 | 141.20 | 142.45 | 138.46 | 138.14 | 145.01 | 144.47 |
| C 14 | 158.73 | 156.94 | 154.68 | 158.35 | 158.41 | 158.14 | 160.49 | 160.08 |
| C 15 | 34.37 | 33.47 | 30.07 | 32.36 | 33.15 | 33.15 | 33.80 | 33.22 |
| C 16 | 67.90 | 68.71 | 69.14 | 68.84 | 68.69 | 68.62 | 68.02 | 68.16 |
| C 17 | 2.59 | 2.86 | 4.05 | 3.11 | 3.81 | 3.67 | 3.73 | 3.60 |
| C 18 | 142.14 | 148.59 | 143.64 | 149.38 | 148.27 | 147.81 | 147.56 | 147.02 |
| C 19 | 148.90 | 152.71 | 147.89 | 147.11 | 147.57 | 147.95 | 147.42 | 147.91 |
| C 20 | -13.36 | -14.65 | -15.15 | -15.03 | -12.06 | -12.11 | -13.39 | -13.37 |
| C 21 | 128.85 | 135.62 | 127.32 | 133.45 | 133.13 | 136.74 | 136.16 | 139.04 |
| C 22 | 144.14 | 142.05 | 144.09 | 148.28 | 144.75 | 144.61 | 145.17 | 145.40 |
| C 23 | 56.86 | 60.22 | 57.93 | 61.04 | 61.09 | 58.83 | 62.29 | 58.32 |
| C 24 | 49.60 | 46.47 | 49.42 | 46.32 | 47.24 | 47.03 | 47.55 | 47.01 |
| C 25 | 167.81 | 160.53 | 167.86 | 162.69 | 169.69 | 164.55 | 168.70 | 162.93 |
| C 27 | 165.92 | 166.12 | 165.82 | 165.55 | 165.64 | 165.71 | 165.65 | 165.76 |
| C 28 | 163.98 | 152.41 | 165.18 | 163.14 | 176.87 | 176.53 | 174.89 | 173.96 |
| C 29 | 163.32 | 167.37 | 164.27 | 169.10 | 171.46 | 169.58 | 171.21 | 168.73 |
| H 8a | 29.65 | 28.78 | 29.54 | 29.26 | 29.94 | 28.81 | 29.93 | 28.96 |
| H 8b | 28.93 | 29.58 | 29.04 | 29.06 | 29.15 | 29.61 | 28.24 | 29.53 |
| H 11 | 28.03 | 27.35 | 27.55 | 27.50 | 27.61 | 27.49 | 28.11 | 28.08 |
| H 13 | 30.10 | 29.74 | 30.13 | 30.41 | 29.83 | 29.78 | 29.08 | 29.10 |
| H 14a | 29.32 | 29.73 | 29.14 | 29.38 | 29.43 | 29.42 | 29.81 | 29.82 |
| H 14b | 29.75 | 28.60 | 29.34 | 29.36 | 29.28 | 29.34 | 29.59 | 29.48 |
| H 16 | 25.86 | 25.92 | 25.89 | 25.90 | 25.88 | 25.88 | 25.87 | 25.84 |
| H 18 | 28.67 | 29.34 | 29.47 | 29.37 | 29.55 | 29.51 | 29.53 | 29.50 |
| H 19a | 28.87 | 29.03 | 28.80 | 28.69 | 28.79 | 28.80 | 28.77 | 28.79 |
| H 19b | 29.74 | 29.62 | 29.79 | 29.67 | 29.85 | 29.81 | 29.81 | 29.77 |
| H 21 | 29.78 | 29.09 | 29.60 | 29.14 | 28.93 | 29.16 | 28.53 | 28.83 |
| H 23 | 25.85 | 24.42 | 25.88 | 25.10 | 25.05 | 24.98 | 25.12 | 24.98 |
| H 25a-c | 30.21 | 30.44 | 30.35 | 30.36 | 30.59 | 30.20 | 30.56 | 30.15 |
| H 27a-c | 29.95 | 30.01 | 29.94 | 29.92 | 29.92 | 29.91 | 29.95 | 29.94 |
| H 28a-c | 30.85 | 30.47 | 30.80 | 30.81 | 31.05 | 31.02 | 31.06 | 31.05 |
| H 29a-c | 30.30 | 30.58 | 30.53 | 30.53 | 30.59 | 30.59 | 30.66 | 30.38 |

| Isotropic shielding constants | | | | | | | | |
|---|---|---|---|---|---|---|---|---|
| Nuclei | Isomer 9 | Isomer 10 | Isomer 11 | Isomer 12 | Isomer 13 | Isomer 14 | Isomer 15 | Isomer 16 |
| C 7 | 7.81 | 7.82 | 8.25 | 8.02 | 7.27 | 7.15 | 7.41 | 7.91 |
| C 8 | 147.55 | 145.39 | 148.13 | 146.36 | 144.93 | 145.54 | 147.77 | 144.07 |
| C 9 | 136.77 | 137.79 | 136.44 | 138.08 | 138.03 | 134.87 | 137.60 | 137.73 |
| C 10 | -15.88 | -20.94 | -20.35 | -24.31 | -23.18 | -21.09 | -20.00 | -21.44 |
| C 11 | 111.92 | 115.14 | 117.30 | 116.46 | 114.12 | 114.87 | 112.22 | 111.82 |
| C 12 | 146.30 | 151.55 | 143.81 | 150.46 | 143.60 | 140.05 | 142.15 | 146.89 |
| C 13 | 142.03 | 138.76 | 142.22 | 140.92 | 144.50 | 138.52 | 153.07 | 149.54 |
| C 14 | 161.83 | 152.81 | 156.63 | 156.23 | 157.81 | 159.92 | 155.91 | 152.52 |
| C 15 | 34.15 | 31.73 | 32.38 | 33.10 | 32.41 | 31.98 | 33.23 | 31.30 |
| C 16 | 67.94 | 67.99 | 68.36 | 67.39 | 68.49 | 68.63 | 68.69 | 68.72 |
| C 17 | 2.85 | 3.21 | 3.49 | 3.07 | 3.80 | 3.36 | 3.54 | 3.81 |
| C 18 | 147.67 | 144.96 | 148.03 | 144.85 | 148.19 | 150.34 | 147.42 | 144.74 |
| C 19 | 146.48 | 152.39 | 149.85 | 152.92 | 148.61 | 146.85 | 150.06 | 152.14 |
| C 20 | -16.05 | -13.85 | -13.55 | -12.53 | -13.26 | -14.61 | -12.76 | -16.21 |
| C 21 | 140.47 | 137.00 | 138.20 | 135.51 | 129.55 | 128.44 | 130.81 | 129.63 |
| C 22 | 144.67 | 146.77 | 143.49 | 147.80 | 146.19 | 145.46 | 143.45 | 148.35 |
| C 23 | 61.94 | 57.91 | 64.66 | 59.98 | 62.44 | 57.63 | 67.04 | 61.70 |
| C 24 | 46.66 | 49.01 | 46.86 | 48.27 | 46.11 | 49.38 | 47.17 | 46.74 |
| C 25 | 170.52 | 155.17 | 169.15 | 154.67 | 169.09 | 156.80 | 167.65 | 152.82 |
| C 27 | 165.71 | 165.89 | 165.73 | 165.92 | 165.70 | 165.51 | 165.94 | 165.89 |
| C 28 | 167.21 | 159.68 | 166.19 | 162.81 | 170.47 | 170.86 | 167.28 | 161.64 |
| C 29 | 165.83 | 163.17 | 168.50 | 162.96 | 168.65 | 165.86 | 167.14 | 167.63 |
| | | | | | | | | |
| H 8a | 29.79 | 29.42 | 29.66 | 29.53 | 29.33 | 28.91 | 29.73 | 29.28 |
| H 8b | 28.95 | 29.04 | 29.10 | 29.05 | 29.40 | 29.59 | 29.11 | 29.12 |
| H 11 | 28.04 | 27.82 | 27.54 | 27.48 | 27.35 | 27.64 | 27.65 | 27.45 |
| H 13 | 30.38 | 29.83 | 30.12 | 29.76 | 29.50 | 30.14 | 29.11 | 29.78 |
| H 14a | 29.05 | 28.94 | 29.61 | 29.68 | 29.58 | 29.49 | 29.54 | 29.79 |
| H 14b | 29.89 | 29.24 | 28.96 | 29.02 | 29.28 | 29.08 | 29.25 | 28.73 |
| H 16 | 25.92 | 25.88 | 25.87 | 25.83 | 25.82 | 25.92 | 25.93 | 25.91 |
| H 18 | 28.77 | 29.47 | 29.57 | 29.61 | 29.56 | 29.31 | 29.53 | 29.52 |
| H 19a | 28.64 | 29.06 | 28.75 | 29.14 | 28.75 | 28.78 | 29.07 | 29.23 |
| H 19b | 29.71 | 29.48 | 29.71 | 29.46 | 29.65 | 29.62 | 29.31 | 29.50 |
| H 21 | 28.33 | 28.64 | 29.06 | 28.97 | 29.75 | 29.49 | 29.30 | 29.24 |
| H 23 | 24.59 | 24.67 | 24.97 | 24.72 | 25.17 | 25.42 | 25.83 | 25.14 |
| H 25a-c | 30.51 | 30.28 | 30.40 | 30.57 | 30.80 | 30.33 | 30.67 | 30.34 |
| H 27a-c | 29.97 | 29.93 | 29.92 | 29.91 | 29.92 | 29.94 | 30.03 | 29.99 |
| H 28a-c | 31.01 | 30.93 | 30.95 | 30.83 | 31.01 | 30.64 | 30.66 | 30.45 |
| H 29a-c | 30.55 | 30.57 | 30.75 | 30.51 | 30.70 | 30.45 | 30.70 | 30.47 |

| Isotropic shielding constants | | | | | | | | |
|---|---|---|---|---|---|---|---|---|
| Nuclei | Isomer 17 | Isomer 18 | Isomer 19 | Isomer 20 | Isomer 21 | Isomer 22 | Isomer 23 | Isomer 24 |
| C 7 | 6.71 | 7.18 | 7.36 | 7.63 | 7.85 | 8.14 | 7.60 | 7.64 |
| C 8 | 144.65 | 147.94 | 145.38 | 144.74 | 146.25 | 147.84 | 145.56 | 147.45 |
| C 9 | 134.78 | 138.51 | 134.51 | 139.22 | 138.14 | 136.11 | 137.56 | 136.37 |
| C 10 | -18.15 | -21.23 | -21.68 | -23.79 | -24.40 | -19.72 | -22.39 | -16.55 |
| C 11 | 113.01 | 113.22 | 121.71 | 118.59 | 115.41 | 115.14 | 118.91 | 115.00 |
| C 12 | 142.88 | 146.35 | 138.05 | 145.32 | 145.45 | 138.53 | 146.76 | 142.58 |
| C 13 | 140.98 | 149.87 | 140.60 | 142.66 | 136.21 | 138.85 | 140.61 | 145.98 |
| C 14 | 159.25 | 154.80 | 155.35 | 157.03 | 156.84 | 157.95 | 160.11 | 160.90 |
| C 15 | 34.03 | 31.39 | 30.97 | 31.67 | 32.99 | 32.83 | 34.03 | 33.82 |
| C 16 | 68.04 | 68.69 | 68.94 | 68.90 | 68.63 | 69.03 | 67.94 | 68.12 |
| C 17 | 2.89 | 2.72 | 4.28 | 3.09 | 3.51 | 3.65 | 3.48 | 3.59 |
| C 18 | 142.67 | 147.84 | 144.84 | 148.06 | 146.78 | 147.74 | 146.99 | 147.63 |
| C 19 | 149.39 | 153.87 | 148.71 | 147.47 | 147.65 | 147.12 | 147.30 | 147.09 |
| C 20 | -12.12 | -14.30 | -13.40 | -15.11 | -12.59 | -12.78 | -14.30 | -13.80 |
| C 21 | 127.85 | 132.54 | 126.50 | 130.20 | 129.82 | 135.05 | 132.02 | 137.27 |
| C 22 | 145.66 | 141.33 | 145.51 | 147.03 | 147.96 | 143.60 | 146.59 | 143.76 |
| C 23 | 56.52 | 65.62 | 56.77 | 63.72 | 60.67 | 64.63 | 59.36 | 63.77 |
| C 24 | 49.51 | 46.73 | 49.93 | 45.17 | 47.89 | 46.93 | 48.27 | 47.03 |
| C 25 | 155.44 | 166.66 | 157.03 | 167.02 | 154.42 | 169.80 | 154.53 | 170.11 |
| C 27 | 165.88 | 166.20 | 165.69 | 165.53 | 165.86 | 165.83 | 165.81 | 165.73 |
| C 28 | 162.07 | 154.38 | 162.96 | 162.91 | 174.32 | 177.78 | 172.89 | 175.44 |
| C 29 | 165.59 | 166.72 | 165.67 | 168.43 | 163.11 | 168.98 | 163.24 | 166.64 |
| | | | | | | | | |
| H 8a | 29.50 | 29.04 | 29.60 | 29.11 | 29.05 | 29.10 | 29.11 | 29.01 |
| H 8b | 29.12 | 29.74 | 28.95 | 29.48 | 29.51 | 29.64 | 29.38 | 29.76 |
| H 11 | 28.02 | 27.71 | 27.46 | 27.11 | 27.57 | 27.77 | 28.14 | 28.13 |
| H 13 | 30.05 | 29.79 | 30.08 | 30.43 | 29.60 | 29.89 | 29.12 | 29.13 |
| H 14a | 29.02 | 29.92 | 29.33 | 29.32 | 29.51 | 29.46 | 29.89 | 29.84 |
| H 14b | 29.72 | 29.16 | 29.20 | 29.35 | 29.17 | 29.21 | 29.38 | 29.68 |
| H 16 | 25.85 | 25.98 | 25.84 | 25.90 | 25.90 | 25.88 | 25.87 | 25.86 |
| H 18 | 28.70 | 29.23 | 29.59 | 29.21 | 29.38 | 29.51 | 29.44 | 29.51 |
| H 19a | 28.73 | 29.23 | 28.70 | 28.72 | 28.99 | 28.78 | 28.94 | 28.73 |
| H 19b | 29.85 | 29.46 | 29.89 | 29.61 | 29.57 | 29.83 | 29.59 | 29.81 |
| H 21 | 29.75 | 29.34 | 29.59 | 29.45 | 29.13 | 29.22 | 28.81 | 28.75 |
| H 23 | 25.11 | 25.99 | 25.24 | 25.45 | 25.07 | 24.74 | 24.89 | 24.69 |
| H 25a-c | 30.37 | 30.49 | 30.35 | 30.76 | 30.62 | 30.53 | 30.38 | 30.50 |
| H 27a-c | 29.93 | 30.08 | 29.91 | 29.94 | 29.93 | 29.94 | 29.94 | 29.95 |
| H 28a-c | 30.77 | 30.56 | 30.73 | 30.97 | 30.99 | 31.01 | 31.09 | 31.02 |
| H 29a-c | 30.27 | 30.68 | 30.53 | 30.67 | 30.49 | 30.77 | 30.58 | 30.55 |

| Isotropic shielding constants | | | | | | | | |
|---|---|---|---|---|---|---|---|---|
| Nuclei | Isomer 25 | Isomer 26 | Isomer 27 | Isomer 28 | Isomer 29 | Isomer 30 | Isomer 31 | Isomer 32 |
| C 7 | 5.74 | 8.86 | 6.88 | 10.17 | 8.20 | 8.01 | 6.66 | 9.28 |
| C 8 | 147.00 | 145.14 | 147.84 | 147.40 | 146.66 | 146.99 | 147.54 | 146.02 |
| C 9 | 132.81 | 136.15 | 133.16 | 135.60 | 135.00 | 137.87 | 133.08 | 136.97 |
| C 10 | -16.91 | -15.82 | -20.96 | -19.92 | -22.03 | -21.03 | -20.51 | -20.02 |
| C 11 | 111.14 | 111.55 | 118.67 | 118.23 | 115.58 | 114.64 | 112.67 | 112.55 |
| C 12 | 145.67 | 146.52 | 144.24 | 144.71 | 146.67 | 143.19 | 141.07 | 142.10 |
| C 13 | 140.45 | 141.23 | 141.76 | 141.66 | 143.08 | 143.26 | 152.65 | 151.68 |
| C 14 | 162.07 | 161.84 | 155.30 | 156.06 | 157.23 | 157.61 | 155.87 | 155.73 |
| C 15 | 33.75 | 34.43 | 33.20 | 32.92 | 33.20 | 32.46 | 33.00 | 32.96 |
| C 16 | 67.92 | 67.82 | 67.86 | 68.08 | 68.17 | 68.41 | 69.33 | 69.20 |
| C 17 | 3.21 | 3.00 | 3.18 | 3.38 | 4.82 | 3.86 | 3.36 | 3.29 |
| C 18 | 147.76 | 147.95 | 146.68 | 147.39 | 148.17 | 148.48 | 147.92 | 147.79 |
| C 19 | 146.50 | 146.36 | 152.42 | 151.11 | 152.03 | 148.49 | 149.57 | 149.19 |
| C 20 | -15.08 | -15.50 | -12.96 | -13.04 | -13.74 | -13.99 | -12.96 | -12.23 |
| C 21 | 142.65 | 140.81 | 140.69 | 136.98 | 134.49 | 129.55 | 133.68 | 130.98 |
| C 22 | 145.67 | 145.40 | 144.29 | 144.11 | 147.15 | 145.46 | 144.60 | 144.57 |
| C 23 | 57.93 | 63.15 | 56.80 | 60.24 | 58.59 | 61.62 | 61.15 | 62.22 |
| C 24 | 46.56 | 47.97 | 47.26 | 47.60 | 46.58 | 47.79 | 46.63 | 47.73 |
| C 25 | 161.58 | 168.66 | 163.96 | 169.67 | 164.29 | 167.38 | 161.65 | 166.86 |
| C 27 | 165.78 | 165.71 | 165.86 | 165.81 | 165.73 | 165.66 | 165.97 | 165.88 |
| C 28 | 165.90 | 167.66 | 163.63 | 164.79 | 169.77 | 170.63 | 165.08 | 166.27 |
| C 29 | 167.25 | 170.93 | 169.20 | 171.51 | 169.17 | 167.62 | 168.24 | 170.73 |
| | | | | | | | | |
| H 8a | 29.51 | 29.93 | 29.63 | 29.13 | 29.10 | 29.30 | 29.63 | 29.07 |
| H 8b | 28.97 | 28.31 | 28.74 | 29.91 | 29.26 | 29.38 | 28.75 | 29.88 |
| H 11 | 28.00 | 28.01 | 27.31 | 27.38 | 27.73 | 27.65 | 27.27 | 27.37 |
| H 13 | 30.30 | 30.34 | 29.91 | 30.02 | 29.95 | 29.80 | 29.35 | 29.62 |
| H 14a | 28.78 | 28.96 | 29.64 | 29.63 | 29.47 | 29.55 | 29.48 | 29.46 |
| H 14b | 29.82 | 29.85 | 29.04 | 28.98 | 29.32 | 29.19 | 28.79 | 29.01 |
| H 16 | 25.93 | 25.92 | 25.81 | 25.84 | 25.79 | 25.86 | 25.92 | 25.92 |
| H 18 | 28.89 | 28.81 | 29.69 | 29.66 | 29.45 | 29.43 | 29.49 | 29.51 |
| H 19a | 28.60 | 28.64 | 28.83 | 28.80 | 29.15 | 28.84 | 28.98 | 28.97 |
| H 19b | 29.77 | 29.76 | 29.72 | 29.75 | 29.04 | 29.50 | 29.46 | 29.50 |
| H 21 | 28.37 | 28.08 | 28.90 | 28.70 | 29.33 | 29.47 | 29.13 | 29.00 |
| H 23 | 24.89 | 25.21 | 24.61 | 24.78 | 24.91 | 25.90 | 25.03 | 25.84 |
| H 25a-c | 30.09 | 30.47 | 30.30 | 30.71 | 30.33 | 30.51 | 30.37 | 30.84 |
| H 27a-c | 29.96 | 29.96 | 29.91 | 29.91 | 29.93 | 29.93 | 30.00 | 30.00 |
| H 28a-c | 30.99 | 31.01 | 30.89 | 30.95 | 30.68 | 30.84 | 30.55 | 30.60 |
| H 29a-c | 30.40 | 30.68 | 30.59 | 30.57 | 30.55 | 30.55 | 30.55 | 30.61 |

| Isotropic shielding constants | |
|---|---|
| Nuclei | Isomer 44 |
| C 7 | 7.83 |
| C 8 | 145.73 |
| C 9 | 138.61 |
| C 10 | -23.22 |
| C 11 | 111.94 |
| C 12 | 148.78 |
| C 13 | 144.30 |
| C 14 | 160.47 |
| C 15 | 32.66 |
| C 16 | 68.52 |
| C 17 | 4.83 |
| C 18 | 148.31 |
| C 19 | 152.61 |
| C 20 | -12.23 |
| C 21 | 134.16 |
| C 22 | 147.27 |
| C 23 | 59.75 |
| C 24 | 48.01 |
| C 25 | 153.74 |
| C 27 | 165.65 |
| C 28 | 173.57 |
| C 29 | 162.54 |
| | |
| H 8a | 29.51 |
| H 8b | 29.02 |
| H 11 | 27.51 |
| H 13 | 29.34 |
| H 14a | 29.23 |
| H 14b | 29.56 |
| H 16 | 25.81 |
| H 18 | 29.10 |
| H 19a | 28.78 |
| H 19b | 29.49 |
| H 21 | 28.99 |
| H 23 | 24.69 |
| H 25a-c | 30.55 |
| H 27a-c | 29.94 |
| H 28a-c | 30.94 |
| H 29a-c | 30.44 |

| | Isomer 1 | Isomer 2 | Isomer 3 | Isomer 4 | Isomer 5 | Isomer 6 | Isomer 7 | Isomer 8 | Isomer 9 | Isomer 10 | Isomer 11 | Isomer 12 | Isomer 13 | Isomer 14 | Isomer 15 | Isomer 16 | Isomer 17 |
|---|---|---|---|---|---|---|---|---|---|---|---|---|---|---|---|---|---|
| $J_{13,14a}$ | 9.73 | 9.37 | 9.94 | 10.25 | 9.37 | 9.48 | 9.89 | 9.86 | 10.47 | 9.79 | 9.85 | 9.52 | 9.47 | 10.15 | 8.57 | 8.61 | 9.83 |
| $J_{13,14b}$ | 2.78 | 4.35 | 2.30 | 3.21 | 4.22 | 4.03 | 3.16 | 2.93 | 3.71 | 3.24 | 3.58 | 3.23 | 3.52 | 3.26 | 4.42 | 2.55 | 2.51 |
| $J_{13,18}$ | 11.60 | 12.99 | 11.11 | 10.97 | 11.33 | 11.34 | 11.50 | 11.50 | 11.42 | 11.70 | 11.89 | 11.76 | 11.60 | 10.83 | 12.00 | 12.11 | 11.45 |
| $J_{18,19a}$ | 11.54 | 11.93 | 11.88 | 9.97 | 10.85 | 10.66 | 11.05 | 10.91 | 9.58 | 10.27 | 10.52 | 10.38 | 9.92 | 9.38 | 9.12 | 11.88 | 11.63 |
| $J_{18,19b}$ | 4.27 | 3.97 | 4.08 | 6.27 | 4.78 | 4.97 | 4.73 | 4.95 | 5.98 | 4.06 | 4.10 | 3.83 | 5.31 | 7.15 | 6.68 | 4.87 | 4.09 |

| | Isomer 18 | Isomer 19 | Isomer 20 | Isomer 21 | Isomer 22 | Isomer 23 | Isomer 24 | Isomer 25 | Isomer 26 | Isomer 27 | Isomer 28 | Isomer 29 | Isomer 30 | Isomer 31 | Isomer 32 | Isomer 44 |
|---|---|---|---|---|---|---|---|---|---|---|---|---|---|---|---|---|
| $J_{13,14a}$ | 9.68 | 9.81 | 10.19 | 9.15 | 9.28 | 9.54 | 9.89 | 10.65 | 10.54 | 9.66 | 9.74 | 9.46 | 9.79 | 8.50 | 8.63 | 3.72 |
| $J_{13,14b}$ | 2.96 | 2.37 | 3.00 | 4.09 | 4.28 | 3.52 | 3.32 | 3.63 | 3.68 | 3.30 | 3.46 | 3.26 | 3.46 | 4.88 | 4.62 | 11.53 |
| $J_{13,18}$ | 12.66 | 11.07 | 10.98 | 11.69 | 11.38 | 11.86 | 11.55 | 11.25 | 11.30 | 12.06 | 11.97 | 11.93 | 11.59 | 11.95 | 11.86 | 8.46 |
| $J_{18,19a}$ | 12.00 | 11.99 | 10.11 | 9.77 | 10.99 | 10.07 | 11.16 | 9.61 | 9.54 | 11.03 | 10.79 | 6.20 | 8.96 | 9.98 | 9.80 | 11.53 |
| $J_{18,19b}$ | 4.04 | 3.65 | 6.13 | 5.81 | 4.67 | 5.68 | 4.56 | 5.56 | 5.95 | 3.14 | 3.56 | 7.93 | 6.34 | 5.99 | 5.94 | 4.79 |

**Table S59.** Studied Boltzmann averaged GIAO isotropic magnetic shielding constants (σ) and coupling constants of isomers calculated at the PCM/B3LYP/6-31G**//MM3 level of theory after elimination of conformations incompatible with experimental $^{3}J_{HH}$ of Zoarenone according to i*J*/d*J*-DP4.

| Isotropic shielding constants | | | | | | | | |
|---|---|---|---|---|---|---|---|---|
| Nuclei | Isomer 1 | Isomer 2 | Isomer 3 | Isomer 4 | Isomer 5 | Isomer 6 | Isomer 7 | Isomer 8 |
| C 7 | 9.79 | 9.46 | 10.07 | 9.72 | 12.18 | 9.08 | 10.60 | 8.19 |
| C 8 | 143.50 | 144.24 | 144.48 | 144.74 | 145.18 | 146.03 | 143.90 | 145.48 |
| C 9 | 136.36 | 136.48 | 136.52 | 136.94 | 135.03 | 133.98 | 135.38 | 134.42 |
| C 10 | -13.29 | -17.28 | -15.90 | -18.57 | -14.23 | -15.31 | -11.77 | -13.51 |
| C 11 | 111.64 | 115.91 | 119.29 | 118.42 | 114.01 | 114.62 | 113.35 | 113.37 |
| C 12 | 140.01 | 148.22 | 136.16 | 145.05 | 138.22 | 137.11 | 141.36 | 140.87 |
| C 13 | 138.88 | 139.75 | 137.69 | 140.33 | 134.60 | 134.29 | 142.50 | 141.89 |
| C 14 | 156.57 | 157.34 | 152.52 | 154.57 | 154.92 | 154.69 | 157.41 | 157.30 |
| C 15 | 39.56 | 39.43 | 34.86 | 36.24 | 37.12 | 37.24 | 38.73 | 38.41 |
| C 16 | 68.11 | 68.27 | 69.63 | 69.16 | 69.07 | 69.06 | 68.29 | 68.42 |
| C 17 | 5.37 | 4.51 | 6.75 | 5.57 | 6.33 | 6.19 | 6.23 | 6.14 |
| C 18 | 141.44 | 147.78 | 144.72 | 148.18 | 145.57 | 145.18 | 145.74 | 145.46 |
| C 19 | 146.47 | 144.00 | 145.19 | 144.44 | 145.91 | 146.06 | 145.70 | 145.95 |
| C 20 | -8.65 | -11.69 | -10.08 | -11.01 | -8.00 | -8.25 | -9.01 | -9.13 |
| C 21 | 127.16 | 132.24 | 125.24 | 130.80 | 131.00 | 134.78 | 134.33 | 137.08 |
| C 22 | 143.73 | 148.08 | 144.02 | 149.78 | 145.25 | 145.22 | 144.84 | 145.16 |
| C 23 | 61.32 | 67.07 | 61.89 | 68.24 | 65.72 | 65.28 | 67.47 | 65.08 |
| C 24 | 55.64 | 52.85 | 55.51 | 52.39 | 53.40 | 52.95 | 53.69 | 52.89 |
| C 25 | 163.96 | 163.65 | 164.13 | 161.14 | 165.67 | 161.61 | 164.78 | 159.91 |
| C 27 | 163.08 | 162.93 | 163.07 | 162.91 | 162.83 | 162.94 | 162.96 | 163.05 |
| C 28 | 160.63 | 155.47 | 162.81 | 159.31 | 172.19 | 171.89 | 170.54 | 169.99 |
| C 29 | 161.58 | 165.89 | 160.86 | 165.76 | 168.50 | 165.86 | 168.23 | 165.21 |
| H 8a | 29.09 | 28.61 | 29.02 | 28.71 | 29.35 | 28.22 | 29.42 | 28.34 |
| H 8b | 28.57 | 28.54 | 28.64 | 28.55 | 28.50 | 29.16 | 27.27 | 29.11 |
| H 11 | 27.56 | 27.63 | 26.90 | 26.92 | 27.05 | 26.90 | 27.73 | 27.74 |
| H 13 | 29.45 | 29.63 | 29.46 | 29.72 | 29.27 | 29.21 | 28.37 | 28.39 |
| H 14a | 28.86 | 28.12 | 28.09 | 28.62 | 28.81 | 28.79 | 29.15 | 29.16 |
| H 14b | 29.05 | 29.11 | 28.79 | 28.81 | 28.47 | 28.58 | 29.15 | 29.03 |
| H 16 | 25.54 | 25.58 | 25.58 | 25.56 | 25.56 | 25.56 | 25.54 | 25.53 |
| H 18 | 27.87 | 27.58 | 28.93 | 28.80 | 28.95 | 28.91 | 28.97 | 28.94 |
| H 19a | 28.29 | 28.12 | 28.18 | 28.18 | 28.40 | 28.41 | 28.35 | 28.36 |
| H 19b | 29.29 | 28.97 | 29.37 | 28.99 | 29.29 | 29.23 | 29.25 | 29.22 |
| H 21 | 29.24 | 28.53 | 29.12 | 28.46 | 28.31 | 28.43 | 27.85 | 28.07 |
| H 23 | 25.87 | 24.99 | 25.86 | 25.19 | 24.96 | 25.12 | 25.01 | 25.12 |
| H 25a-c | 29.51 | 29.49 | 29.63 | 29.82 | 29.95 | 29.67 | 29.94 | 29.66 |
| H 27a-c | 29.44 | 29.45 | 29.43 | 29.41 | 29.42 | 29.41 | 29.44 | 29.44 |
| H 28a-c | 30.11 | 30.11 | 30.02 | 30.06 | 30.32 | 30.30 | 30.37 | 30.37 |
| H 29a-c | 29.68 | 29.93 | 29.95 | 29.87 | 30.01 | 29.96 | 30.08 | 29.73 |

| Isotropic shielding constants | | | | | | | | |
|---|---|---|---|---|---|---|---|---|
| Nuclei | Isomer 9 | Isomer 10 | Isomer 11 | Isomer 12 | Isomer 13 | Isomer 14 | Isomer 15 | Isomer 16 |
| C 7 | 10.43 | 9.68 | 10.81 | 9.80 | 9.20 | 9.50 | 10.05 | 9.06 |
| C 8 | 145.35 | 143.05 | 146.24 | 143.80 | 143.51 | 144.41 | 145.88 | 143.33 |
| C 9 | 136.21 | 137.97 | 136.33 | 138.23 | 137.32 | 136.50 | 136.59 | 138.20 |
| C 10 | -11.92 | -16.99 | -15.16 | -19.70 | -17.99 | -16.03 | -15.42 | -17.44 |
| C 11 | 110.51 | 115.12 | 115.96 | 114.65 | 112.40 | 113.49 | 110.89 | 110.15 |
| C 12 | 143.86 | 150.65 | 141.81 | 149.44 | 141.36 | 137.43 | 140.44 | 144.40 |
| C 13 | 138.71 | 133.56 | 138.26 | 136.08 | 141.74 | 135.48 | 149.87 | 146.58 |
| C 14 | 158.61 | 149.42 | 153.21 | 153.69 | 154.33 | 156.89 | 152.31 | 152.07 |
| C 15 | 38.54 | 36.99 | 37.27 | 38.54 | 36.80 | 36.58 | 36.94 | 36.61 |
| C 16 | 68.31 | 68.28 | 68.47 | 67.60 | 68.96 | 68.63 | 69.01 | 69.31 |
| C 17 | 5.52 | 5.90 | 5.98 | 5.85 | 6.56 | 5.63 | 6.04 | 6.59 |
| C 18 | 145.87 | 142.35 | 145.30 | 142.02 | 146.21 | 149.17 | 144.79 | 145.48 |
| C 19 | 143.96 | 149.78 | 148.15 | 150.20 | 147.24 | 144.26 | 147.70 | 149.97 |
| C 20 | -11.82 | -10.16 | -9.87 | -8.96 | -9.20 | -10.82 | -8.72 | -10.84 |
| C 21 | 139.90 | 134.73 | 137.11 | 133.21 | 128.74 | 126.15 | 129.52 | 126.69 |
| C 22 | 145.30 | 148.10 | 144.33 | 149.00 | 147.31 | 145.50 | 144.59 | 148.24 |
| C 23 | 66.75 | 64.23 | 69.62 | 66.54 | 66.96 | 64.59 | 71.67 | 68.02 |
| C 24 | 53.49 | 55.01 | 53.38 | 54.19 | 52.49 | 55.30 | 53.80 | 52.47 |
| C 25 | 167.24 | 151.48 | 165.77 | 151.25 | 165.66 | 154.85 | 164.86 | 148.90 |
| C 27 | 162.97 | 162.98 | 162.97 | 163.03 | 162.93 | 162.83 | 163.07 | 163.08 |
| C 28 | 163.63 | 155.06 | 162.36 | 156.70 | 166.60 | 165.64 | 164.02 | 161.22 |
| C 29 | 163.46 | 158.75 | 165.22 | 159.23 | 165.28 | 162.30 | 164.15 | 160.29 |
| | | | | | | | | |
| H 8a | 29.19 | 28.85 | 29.06 | 28.94 | 28.74 | 28.33 | 29.13 | 28.81 |
| H 8b | 28.53 | 28.56 | 28.65 | 28.57 | 28.91 | 29.17 | 28.67 | 28.62 |
| H 11 | 27.64 | 27.52 | 26.89 | 26.94 | 26.66 | 27.07 | 27.03 | 26.84 |
| H 13 | 29.69 | 29.21 | 29.41 | 29.08 | 28.88 | 29.54 | 28.34 | 28.84 |
| H 14a | 28.44 | 28.28 | 29.03 | 29.13 | 28.91 | 28.83 | 29.00 | 29.03 |
| H 14b | 29.31 | 28.66 | 28.26 | 28.44 | 28.58 | 28.45 | 28.17 | 27.89 |
| H 16 | 25.59 | 25.58 | 25.54 | 25.52 | 25.49 | 25.55 | 25.60 | 25.60 |
| H 18 | 28.16 | 28.82 | 28.96 | 28.94 | 28.92 | 28.68 | 28.84 | 28.86 |
| H 19a | 28.14 | 28.56 | 28.30 | 28.65 | 28.34 | 28.32 | 28.57 | 28.58 |
| H 19b | 29.19 | 28.93 | 29.15 | 28.91 | 28.97 | 28.98 | 28.63 | 28.77 |
| H 21 | 27.55 | 27.97 | 28.32 | 28.27 | 29.06 | 28.80 | 28.65 | 28.65 |
| H 23 | 24.53 | 24.59 | 24.96 | 24.72 | 25.06 | 25.67 | 25.58 | 25.26 |
| H 25a-c | 29.90 | 29.61 | 29.75 | 30.00 | 30.16 | 29.92 | 30.10 | 29.84 |
| H 27a-c | 29.46 | 29.44 | 29.42 | 29.42 | 29.42 | 29.43 | 29.53 | 29.53 |
| H 28a-c | 30.34 | 30.22 | 30.25 | 30.10 | 30.29 | 29.90 | 30.03 | 29.87 |
| H 29a-c | 29.94 | 29.96 | 30.14 | 29.91 | 30.12 | 29.88 | 30.11 | 29.83 |

| Isotropic shielding constants | | | | | | | | |
|---|---|---|---|---|---|---|---|---|
| Nuclei | Isomer 17 | Isomer 18 | Isomer 19 | Isomer 20 | Isomer 21 | Isomer 22 | Isomer 23 | Isomer 24 |
| C 7 | 8.98 | 9.40 | 9.74 | 10.12 | 9.70 | 10.65 | 9.48 | 10.25 |
| C 8 | 143.45 | 145.86 | 144.25 | 142.99 | 143.58 | 145.99 | 142.98 | 145.04 |
| C 9 | 135.60 | 137.31 | 135.42 | 138.13 | 138.53 | 136.24 | 138.11 | 136.39 |
| C 10 | -14.02 | -16.62 | -16.44 | -18.88 | -19.65 | -14.53 | -18.20 | -12.75 |
| C 11 | 111.85 | 111.72 | 120.13 | 117.01 | 113.53 | 113.32 | 118.21 | 113.66 |
| C 12 | 139.97 | 144.01 | 135.86 | 143.99 | 144.47 | 136.66 | 146.15 | 140.53 |
| C 13 | 138.74 | 145.70 | 138.08 | 140.23 | 132.05 | 135.08 | 137.23 | 143.57 |
| C 14 | 156.89 | 151.76 | 152.69 | 153.68 | 153.45 | 154.42 | 156.98 | 157.76 |
| C 15 | 39.40 | 36.55 | 35.36 | 35.65 | 37.18 | 36.79 | 38.75 | 38.62 |
| C 16 | 68.25 | 68.81 | 69.43 | 69.27 | 69.10 | 69.33 | 68.35 | 68.41 |
| C 17 | 5.76 | 5.59 | 7.00 | 5.59 | 6.07 | 6.19 | 5.99 | 6.06 |
| C 18 | 142.20 | 145.83 | 145.44 | 147.94 | 144.17 | 145.14 | 144.69 | 145.55 |
| C 19 | 146.99 | 151.08 | 146.21 | 144.53 | 145.91 | 145.47 | 145.70 | 145.50 |
| C 20 | -8.27 | -11.01 | -9.09 | -10.73 | -8.32 | -9.04 | -9.69 | -9.77 |
| C 21 | 126.43 | 130.83 | 124.61 | 128.92 | 126.34 | 133.77 | 129.02 | 136.34 |
| C 22 | 145.36 | 141.49 | 145.28 | 147.96 | 149.05 | 144.71 | 147.79 | 144.14 |
| C 23 | 62.57 | 70.28 | 62.31 | 67.93 | 66.86 | 69.57 | 65.55 | 68.84 |
| C 24 | 56.37 | 52.99 | 56.62 | 51.75 | 54.00 | 53.35 | 54.45 | 53.73 |
| C 25 | 152.24 | 163.75 | 153.48 | 165.04 | 151.06 | 166.24 | 151.38 | 166.62 |
| C 27 | 163.01 | 163.33 | 162.97 | 162.91 | 163.00 | 162.97 | 163.02 | 162.95 |
| C 28 | 158.48 | 149.93 | 159.88 | 159.46 | 169.29 | 173.82 | 168.15 | 171.76 |
| C 29 | 162.80 | 163.40 | 161.50 | 165.42 | 159.41 | 165.82 | 159.02 | 164.16 |
| | | | | | | | | |
| H 8a | 29.13 | 28.64 | 29.19 | 28.60 | 28.56 | 28.64 | 28.61 | 28.60 |
| H 8b | 28.50 | 29.10 | 28.39 | 28.81 | 28.92 | 29.06 | 28.80 | 29.15 |
| H 11 | 27.62 | 27.10 | 26.81 | 26.29 | 27.02 | 27.13 | 27.78 | 27.74 |
| H 13 | 29.41 | 29.10 | 29.44 | 29.76 | 29.07 | 29.30 | 28.55 | 28.41 |
| H 14a | 28.58 | 29.17 | 28.43 | 28.43 | 28.87 | 28.84 | 29.22 | 29.17 |
| H 14b | 29.04 | 28.27 | 28.66 | 28.85 | 28.40 | 28.36 | 28.90 | 29.22 |
| H 16 | 25.53 | 25.66 | 25.53 | 25.57 | 25.59 | 25.56 | 25.55 | 25.54 |
| H 18 | 27.93 | 28.39 | 29.06 | 28.64 | 28.77 | 28.90 | 28.84 | 28.93 |
| H 19a | 28.16 | 28.69 | 28.09 | 28.17 | 28.62 | 28.39 | 28.57 | 28.34 |
| H 19b | 29.40 | 28.81 | 29.46 | 28.95 | 28.96 | 29.27 | 29.00 | 29.25 |
| H 21 | 29.12 | 28.65 | 29.00 | 28.75 | 28.46 | 28.54 | 28.15 | 28.07 |
| H 23 | 25.18 | 25.89 | 25.31 | 25.27 | 25.08 | 24.71 | 24.86 | 24.71 |
| H 25a-c | 29.83 | 29.87 | 29.84 | 30.16 | 30.06 | 29.89 | 29.76 | 29.86 |
| H 27a-c | 29.42 | 29.59 | 29.40 | 29.42 | 29.43 | 29.43 | 29.44 | 29.45 |
| H 28a-c | 30.10 | 29.89 | 30.00 | 30.28 | 30.25 | 30.31 | 30.37 | 30.35 |
| H 29a-c | 29.66 | 30.10 | 29.98 | 30.10 | 29.89 | 30.17 | 29.97 | 29.92 |

| Isotropic shielding constants | | | | | | | | |
|---|---|---|---|---|---|---|---|---|
| Nuclei | Isomer 25 | Isomer 26 | Isomer 27 | Isomer 28 | Isomer 29 | Isomer 30 | Isomer 31 | Isomer 32 |
| C 7 | 7.47 | 10.54 | 8.95 | 12.14 | 10.16 | 9.80 | 8.76 | 11.15 |
| C 8 | 145.70 | 143.44 | 146.39 | 144.78 | 144.89 | 144.96 | 146.17 | 143.20 |
| C 9 | 134.19 | 135.18 | 134.50 | 135.24 | 136.13 | 137.24 | 134.61 | 136.38 |
| C 10 | -12.88 | -11.51 | -15.71 | -14.80 | -17.33 | -15.48 | -15.65 | -15.62 |
| C 11 | 109.80 | 109.93 | 116.91 | 116.53 | 114.65 | 112.33 | 111.04 | 110.76 |
| C 12 | 143.05 | 144.02 | 142.10 | 142.57 | 144.17 | 139.17 | 138.82 | 140.91 |
| C 13 | 137.34 | 138.07 | 137.14 | 137.24 | 140.41 | 138.05 | 148.07 | 147.75 |
| C 14 | 158.85 | 158.54 | 152.28 | 152.91 | 153.67 | 155.18 | 152.18 | 152.34 |
| C 15 | 38.29 | 38.80 | 38.51 | 37.91 | 37.78 | 35.95 | 36.65 | 36.60 |
| C 16 | 68.33 | 68.20 | 68.03 | 68.25 | 68.62 | 68.84 | 69.47 | 69.47 |
| C 17 | 5.84 | 5.66 | 5.70 | 5.92 | 7.82 | 6.17 | 5.88 | 5.90 |
| C 18 | 145.94 | 146.08 | 144.13 | 144.88 | 146.27 | 147.70 | 145.34 | 145.64 |
| C 19 | 143.90 | 143.87 | 150.21 | 148.96 | 152.29 | 144.35 | 146.99 | 147.04 |
| C 20 | -10.90 | -11.01 | -9.38 | -9.30 | -10.13 | -11.08 | -9.12 | -8.29 |
| C 21 | 141.11 | 139.35 | 139.25 | 134.76 | 133.31 | 126.73 | 131.68 | 128.35 |
| C 22 | 145.91 | 145.66 | 144.76 | 144.62 | 148.01 | 144.72 | 145.56 | 145.41 |
| C 23 | 65.04 | 68.61 | 63.74 | 64.97 | 64.64 | 64.62 | 68.51 | 66.06 |
| C 24 | 52.46 | 54.02 | 53.24 | 53.77 | 52.66 | 54.44 | 52.69 | 53.59 |
| C 25 | 158.64 | 164.52 | 161.28 | 165.61 | 163.50 | 164.47 | 159.15 | 162.98 |
| C 27 | 162.96 | 163.00 | 163.01 | 162.97 | 162.92 | 162.87 | 162.99 | 162.97 |
| C 28 | 161.87 | 163.13 | 159.05 | 160.15 | 165.27 | 167.20 | 162.01 | 162.93 |
| C 29 | 163.82 | 168.13 | 165.52 | 168.61 | 166.21 | 162.16 | 164.38 | 167.15 |
| H 8a | 29.09 | 29.46 | 29.17 | 28.46 | 28.59 | 28.72 | 29.19 | 28.43 |
| H 8b | 28.36 | 27.34 | 28.18 | 29.33 | 28.68 | 29.01 | 28.18 | 29.29 |
| H 11 | 27.62 | 27.61 | 26.70 | 26.78 | 27.12 | 27.03 | 26.71 | 26.76 |
| H 13 | 29.64 | 29.67 | 29.24 | 29.35 | 29.50 | 29.38 | 28.63 | 28.84 |
| H 14a | 28.08 | 28.32 | 29.06 | 29.05 | 28.80 | 28.97 | 28.95 | 28.94 |
| H 14b | 29.25 | 29.27 | 28.43 | 28.32 | 28.77 | 28.33 | 27.61 | 27.90 |
| H 16 | 25.60 | 25.59 | 25.49 | 25.52 | 25.46 | 25.56 | 25.61 | 25.61 |
| H 18 | 28.28 | 28.22 | 29.04 | 29.03 | 28.78 | 28.79 | 28.83 | 28.86 |
| H 19a | 28.11 | 28.13 | 28.41 | 28.35 | 28.91 | 28.28 | 28.47 | 28.46 |
| H 19b | 29.22 | 29.22 | 29.12 | 29.17 | 28.05 | 29.05 | 28.86 | 28.87 |
| H 21 | 27.54 | 27.33 | 28.21 | 28.05 | 28.59 | 28.92 | 28.49 | 28.47 |
| H 23 | 25.10 | 25.19 | 24.78 | 24.69 | 24.94 | 25.95 | 25.15 | 25.89 |
| H 25a-c | 29.56 | 29.83 | 29.79 | 30.07 | 29.82 | 29.83 | 29.86 | 30.18 |
| H 27a-c | 29.45 | 29.45 | 29.41 | 29.41 | 29.44 | 29.44 | 29.51 | 29.49 |
| H 28a-c | 30.34 | 30.37 | 30.19 | 30.24 | 29.88 | 30.05 | 29.93 | 29.97 |
| H 29a-c | 29.74 | 30.10 | 29.95 | 30.00 | 29.92 | 29.86 | 29.92 | 30.02 |

| Isotropic shielding constants | | | |
|---|---|---|---|
| Nuclei | Isomer 34 | Isomer 44 | Isomer 50 |
| C 7 | 9.30 | 9.63 | 9.50 |
| C 8 | 146.48 | 143.35 | 145.66 |
| C 9 | 135.38 | 138.74 | 137.57 |
| C 10 | -15.44 | -18.72 | -15.50 |
| C 11 | 112.49 | 110.08 | 111.78 |
| C 12 | 141.35 | 147.67 | 142.15 |
| C 13 | 151.09 | 140.85 | 153.84 |
| C 14 | 155.12 | 156.97 | 155.33 |
| C 15 | 36.58 | 36.86 | 37.26 |
| C 16 | 68.10 | 68.23 | 68.19 |
| C 17 | 7.55 | 7.40 | 7.40 |
| C 18 | 148.33 | 146.12 | 148.59 |
| C 19 | 151.89 | 149.70 | 151.65 |
| C 20 | -11.01 | -8.42 | -11.04 |
| C 21 | 132.58 | 131.53 | 130.16 |
| C 22 | 143.14 | 148.64 | 142.00 |
| C 23 | 68.25 | 66.32 | 74.03 |
| C 24 | 52.73 | 53.99 | 53.41 |
| C 25 | 160.89 | 150.41 | 163.82 |
| C 27 | 163.05 | 163.08 | 163.23 |
| C 28 | 154.82 | 168.71 | 157.14 |
| C 29 | 164.79 | 158.56 | 163.80 |
| | | | |
| H 8a | 28.18 | 28.93 | 28.72 |
| H 8b | 29.23 | 28.54 | 29.12 |
| H 11 | 26.90 | 26.94 | 27.11 |
| H 13 | 28.86 | 28.70 | 29.16 |
| H 14a | 28.27 | 28.53 | 28.18 |
| H 14b | 28.97 | 28.94 | 29.06 |
| H 16 | 25.49 | 25.46 | 25.52 |
| H 18 | 28.57 | 28.41 | 28.31 |
| H 19a | 28.39 | 28.28 | 28.26 |
| H 19b | 28.90 | 28.95 | 28.92 |
| H 21 | 28.39 | 28.23 | 28.68 |
| H 23 | 24.26 | 24.71 | 25.94 |
| H 25a-c | 30.04 | 29.98 | 29.91 |
| H 27a-c | 29.44 | 29.42 | 29.50 |
| H 28a-c | 29.81 | 30.17 | 29.92 |
| H 29a-c | 29.91 | 29.83 | 30.10 |

| | Isomer 1 | Isomer 2 | Isomer 3 | Isomer 4 | Isomer 5 | Isomer 6 | Isomer 7 | Isomer 8 | Isomer 9 | Isomer 10 | Isomer 11 | Isomer 12 | Isomer 13 | Isomer 14 | Isomer 15 | Isomer 16 | Isomer 17 |
|---|---|---|---|---|---|---|---|---|---|---|---|---|---|---|---|---|---|
| $J_{13,14a}$ | 10.97 | 11.29 | 11.27 | 11.43 | 10.57 | 10.62 | 10.89 | 10.84 | 11.56 | 11.06 | 11.00 | 10.80 | 10.56 | 11.10 | 9.97 | 10.14 | 10.97 |
| $J_{13,14b}$ | 3.30 | 4.12 | 2.81 | 3.35 | 4.01 | 3.92 | 3.42 | 3.35 | 4.03 | 3.38 | 3.66 | 3.34 | 3.65 | 3.78 | 4.18 | 3.70 | 3.25 |
| $J_{13,18}$ | 13.00 | 12.89 | 12.45 | 12.44 | 12.62 | 12.63 | 13.06 | 13.05 | 13.02 | 12.72 | 13.26 | 12.67 | 13.08 | 12.37 | 13.27 | 13.21 | 12.92 |
| $J_{18,19a}$ | 12.75 | 10.22 | 13.23 | 10.51 | 12.10 | 11.89 | 12.27 | 12.12 | 10.31 | 11.56 | 11.80 | 11.73 | 10.24 | 9.47 | 10.35 | 10.94 | 13.01 |
| $J_{18,19b}$ | 4.33 | 7.92 | 4.16 | 7.38 | 5.13 | 5.31 | 4.96 | 5.15 | 6.41 | 4.09 | 4.08 | 3.87 | 6.23 | 8.63 | 7.13 | 6.35 | 4.03 |

| | Isomer 18 | Isomer 19 | Isomer 20 | Isomer 21 | Isomer 22 | Isomer 23 | Isomer 24 | Isomer 25 | Isomer 26 | Isomer 27 | Isomer 28 | Isomer 29 | Isomer 30 | Isomer 31 | Isomer 32 | Isomer 34 | Isomer 44 | Isomer 50 |
|---|---|---|---|---|---|---|---|---|---|---|---|---|---|---|---|---|---|---|
| $J_{13,14a}$ | 11.03 | 11.15 | 11.52 | 10.47 | 10.52 | 10.75 | 10.93 | 11.64 | 11.58 | 10.85 | 10.93 | 10.47 | 11.09 | 10.03 | 10.11 | 3.70 | 4.05 | 3.85 |
| $J_{13,14b}$ | 3.20 | 2.75 | 3.23 | 4.00 | 4.05 | 3.55 | 3.45 | 4.03 | 4.01 | 3.47 | 3.61 | 3.45 | 3.65 | 4.33 | 4.23 | 12.43 | 12.72 | 12.75 |
| $J_{13,18}$ | 13.76 | 12.36 | 12.36 | 12.81 | 12.65 | 13.10 | 13.10 | 12.98 | 12.99 | 13.30 | 13.28 | 13.52 | 12.71 | 13.16 | 13.13 | 8.37 | 8.69 | 9.04 |
| $J_{18,19a}$ | 13.89 | 13.39 | 10.55 | 10.75 | 12.27 | 11.06 | 12.41 | 10.38 | 10.29 | 12.40 | 12.07 | 4.02 | 9.97 | 11.47 | 11.21 | 15.78 | 13.03 | 14.94 |
| $J_{18,19b}$ | 3.91 | 3.69 | 7.31 | 6.29 | 4.98 | 6.10 | 4.81 | 5.91 | 6.29 | 3.27 | 3.71 | 10.67 | 7.25 | 6.12 | 6.17 | 3.91 | 5.05 | 4.60 |

**Table S60.** Studied Boltzmann averaged GIAO isotropic magnetic shielding constants (σ) and coupling constants of isomers calculated at the PCM/B3LYP/6-31G**//MMFF level of theory after elimination of conformations incompatible with experimental $^3J_{HH}$ of Zoarenone according to i*J*/d*J*-DP4.

| Isotropic shielding constants | | | | | | | | |
|---|---|---|---|---|---|---|---|---|
| Nuclei | Isomer 1 | Isomer 2 | Isomer 3 | Isomer 4 | Isomer 5 | Isomer 6 | Isomer 7 | Isomer 8 |
| C 7 | 3.23 | 2.27 | 3.35 | 2.92 | 5.67 | 2.25 | 4.42 | 1.38 |
| C 8 | 146.72 | 148.43 | 147.41 | 146.99 | 147.28 | 148.46 | 145.77 | 147.90 |
| C 9 | 134.57 | 133.74 | 135.06 | 135.20 | 133.68 | 131.64 | 133.84 | 131.80 |
| C 10 | -22.11 | -24.48 | -24.59 | -26.78 | -23.42 | -23.04 | -21.38 | -21.22 |
| C 11 | 111.55 | 112.23 | 118.89 | 117.67 | 113.41 | 113.88 | 113.01 | 112.27 |
| C 12 | 137.43 | 141.98 | 132.88 | 144.20 | 136.67 | 135.17 | 139.44 | 138.51 |
| C 13 | 139.76 | 144.53 | 138.01 | 140.55 | 136.09 | 135.79 | 143.53 | 142.72 |
| C 14 | 158.95 | 155.86 | 154.96 | 157.00 | 157.28 | 157.13 | 159.85 | 159.45 |
| C 15 | 40.15 | 38.23 | 35.54 | 37.38 | 37.98 | 38.15 | 39.51 | 39.04 |
| C 16 | 66.81 | 67.63 | 68.37 | 68.30 | 67.94 | 67.83 | 67.04 | 67.18 |
| C 17 | -0.91 | -0.59 | 0.44 | -1.44 | -0.03 | -0.23 | -0.11 | -0.30 |
| C 18 | 143.01 | 148.30 | 143.97 | 149.70 | 147.45 | 147.02 | 147.41 | 146.99 |
| C 19 | 148.55 | 152.31 | 147.45 | 147.70 | 147.50 | 147.92 | 147.36 | 147.84 |
| C 20 | -16.22 | -18.64 | -18.18 | -18.50 | -14.81 | -14.68 | -16.14 | -15.87 |
| C 21 | 125.73 | 132.40 | 124.08 | 131.48 | 130.70 | 134.18 | 133.41 | 136.07 |
| C 22 | 142.08 | 141.39 | 142.43 | 147.27 | 143.41 | 143.63 | 143.41 | 143.89 |
| C 23 | 64.77 | 70.15 | 65.69 | 71.46 | 68.33 | 68.31 | 69.53 | 68.05 |
| C 24 | 51.73 | 48.80 | 51.32 | 48.45 | 49.90 | 49.15 | 50.30 | 49.03 |
| C 25 | 166.37 | 163.22 | 167.01 | 165.19 | 169.39 | 164.91 | 168.40 | 163.42 |
| C 27 | 166.52 | 166.60 | 166.47 | 166.17 | 166.23 | 166.36 | 166.35 | 166.44 |
| C 28 | 164.56 | 152.25 | 165.91 | 163.03 | 176.29 | 175.75 | 174.27 | 173.22 |
| C 29 | 164.24 | 167.04 | 163.77 | 168.33 | 170.56 | 168.58 | 170.10 | 167.75 |
| | | | | | | | | |
| H 8a | 29.51 | 28.60 | 29.41 | 29.09 | 29.63 | 28.61 | 29.63 | 28.74 |
| H 8b | 28.52 | 29.32 | 28.61 | 28.76 | 28.99 | 29.40 | 27.91 | 29.32 |
| H 11 | 27.71 | 27.10 | 27.20 | 27.25 | 27.32 | 27.17 | 27.87 | 27.88 |
| H 13 | 29.71 | 29.45 | 29.72 | 30.04 | 29.52 | 29.44 | 28.69 | 28.72 |
| H 14a | 29.25 | 29.43 | 28.72 | 29.07 | 29.25 | 29.23 | 29.59 | 29.60 |
| H 14b | 29.52 | 28.30 | 29.24 | 29.23 | 28.95 | 29.09 | 29.52 | 29.37 |
| H 16 | 25.65 | 25.71 | 25.68 | 25.68 | 25.67 | 25.66 | 25.65 | 25.63 |
| H 18 | 28.41 | 28.97 | 29.27 | 29.19 | 29.30 | 29.24 | 29.32 | 29.27 |
| H 19a | 28.61 | 28.75 | 28.55 | 28.61 | 28.69 | 28.73 | 28.64 | 28.69 |
| H 19b | 29.66 | 29.36 | 29.70 | 29.13 | 29.64 | 29.55 | 29.61 | 29.54 |
| H 21 | 29.43 | 28.70 | 29.21 | 28.72 | 28.69 | 28.79 | 28.26 | 28.47 |
| H 23 | 26.05 | 24.73 | 26.07 | 25.50 | 25.23 | 25.24 | 25.29 | 25.26 |
| H 25a-c | 30.01 | 30.41 | 30.21 | 30.39 | 30.37 | 30.13 | 30.36 | 30.09 |
| H 27a-c | 29.92 | 29.98 | 29.91 | 29.88 | 29.90 | 29.88 | 29.92 | 29.92 |
| H 28a-c | 30.66 | 30.31 | 30.57 | 30.55 | 30.81 | 30.78 | 30.87 | 30.85 |
| H 29a-c | 30.14 | 30.46 | 30.38 | 30.39 | 30.43 | 30.50 | 30.49 | 30.25 |

| Isotropic shielding constants | | | | | | | | |
|---|---|---|---|---|---|---|---|---|
| Nuclei | Isomer 9 | Isomer 10 | Isomer 11 | Isomer 12 | Isomer 13 | Isomer 14 | Isomer 15 | Isomer 16 |
| C 7 | 3.94 | 2.46 | 4.21 | 2.70 | 4.01 | 2.54 | 3.74 | 2.86 |
| C 8 | 148.26 | 144.74 | 148.96 | 145.38 | 145.89 | 146.53 | 148.53 | 144.99 |
| C 9 | 134.44 | 136.29 | 134.77 | 136.60 | 135.78 | 134.78 | 135.10 | 136.39 |
| C 10 | -20.62 | -25.38 | -23.71 | -28.44 | -27.35 | -23.49 | -23.74 | -24.73 |
| C 11 | 110.07 | 114.16 | 115.24 | 113.91 | 111.50 | 113.23 | 110.87 | 110.49 |
| C 12 | 141.81 | 147.70 | 139.40 | 147.37 | 140.66 | 135.48 | 138.92 | 142.78 |
| C 13 | 139.87 | 134.24 | 138.85 | 136.37 | 142.45 | 137.51 | 150.82 | 146.93 |
| C 14 | 160.90 | 152.15 | 156.43 | 155.85 | 156.83 | 158.99 | 154.50 | 154.28 |
| C 15 | 39.44 | 36.90 | 37.31 | 38.46 | 37.67 | 38.08 | 37.50 | 37.22 |
| C 16 | 67.25 | 67.02 | 67.65 | 66.39 | 67.73 | 67.61 | 67.89 | 67.96 |
| C 17 | -1.16 | 0.06 | -0.19 | 0.09 | 0.14 | -1.23 | -0.26 | 0.06 |
| C 18 | 147.60 | 144.55 | 148.11 | 144.42 | 148.51 | 150.37 | 147.34 | 147.27 |
| C 19 | 146.01 | 151.55 | 148.63 | 151.93 | 149.11 | 147.57 | 150.47 | 151.93 |
| C 20 | -17.85 | -16.31 | -16.48 | -14.88 | -16.77 | -17.10 | -16.48 | -18.19 |
| C 21 | 138.46 | 133.49 | 135.42 | 132.33 | 129.09 | 125.70 | 129.63 | 127.13 |
| C 22 | 143.52 | 145.65 | 142.56 | 146.53 | 145.54 | 143.76 | 141.76 | 145.88 |
| C 23 | 70.13 | 66.97 | 72.13 | 69.50 | 70.18 | 67.85 | 74.82 | 70.74 |
| C 24 | 49.72 | 50.52 | 49.59 | 49.82 | 49.34 | 50.80 | 49.68 | 49.05 |
| C 25 | 169.11 | 154.00 | 168.03 | 154.13 | 167.58 | 157.96 | 166.63 | 154.08 |
| C 27 | 166.35 | 166.50 | 166.23 | 166.53 | 166.25 | 166.17 | 166.51 | 166.51 |
| C 28 | 167.70 | 159.37 | 166.58 | 161.08 | 169.84 | 168.92 | 166.53 | 161.83 |
| C 29 | 166.07 | 162.94 | 168.16 | 162.88 | 168.20 | 164.98 | 167.38 | 165.75 |
| | | | | | | | | |
| H 8a | 29.62 | 29.16 | 29.50 | 29.29 | 29.18 | 28.67 | 29.55 | 29.10 |
| H 8b | 28.46 | 28.79 | 28.63 | 28.77 | 28.96 | 29.39 | 28.69 | 28.87 |
| H 11 | 27.79 | 27.66 | 27.18 | 27.30 | 27.14 | 27.34 | 27.26 | 27.28 |
| H 13 | 29.96 | 29.50 | 29.82 | 29.38 | 29.26 | 29.77 | 28.98 | 29.22 |
| H 14a | 28.89 | 28.62 | 29.38 | 29.49 | 29.33 | 29.30 | 29.39 | 29.47 |
| H 14b | 29.70 | 29.18 | 28.75 | 28.81 | 29.02 | 29.01 | 28.72 | 28.56 |
| H 16 | 25.70 | 25.70 | 25.68 | 25.64 | 25.60 | 25.66 | 25.72 | 25.70 |
| H 18 | 28.59 | 29.16 | 29.24 | 29.26 | 29.32 | 28.99 | 29.25 | 29.25 |
| H 19a | 28.57 | 28.88 | 28.59 | 28.97 | 28.54 | 28.90 | 28.84 | 28.89 |
| H 19b | 29.38 | 29.32 | 29.46 | 29.31 | 29.42 | 28.93 | 29.07 | 29.25 |
| H 21 | 28.01 | 28.33 | 28.70 | 28.65 | 29.28 | 29.09 | 28.91 | 28.95 |
| H 23 | 24.66 | 24.98 | 24.93 | 25.08 | 25.35 | 25.92 | 25.97 | 25.53 |
| H 25a-c | 30.37 | 30.11 | 30.30 | 30.49 | 30.61 | 30.39 | 30.51 | 30.32 |
| H 27a-c | 29.94 | 29.91 | 29.89 | 29.90 | 29.90 | 29.91 | 30.00 | 29.98 |
| H 28a-c | 30.86 | 30.71 | 30.78 | 30.57 | 30.75 | 30.38 | 30.48 | 30.30 |
| H 29a-c | 30.38 | 30.44 | 30.60 | 30.37 | 30.55 | 30.36 | 30.56 | 30.35 |

| Isotropic shielding constants | | | | | | | | |
|---|---|---|---|---|---|---|---|---|
| Nuclei | Isomer 17 | Isomer 18 | Isomer 19 | Isomer 20 | Isomer 21 | Isomer 22 | Isomer 23 | Isomer 24 |
| C 7 | 1.94 | 3.54 | 2.69 | 4.12 | 2.59 | 4.00 | 2.34 | 3.68 |
| C 8 | 145.69 | 148.53 | 146.31 | 145.39 | 145.36 | 148.75 | 144.66 | 148.00 |
| C 9 | 132.95 | 135.86 | 133.22 | 136.69 | 136.70 | 134.79 | 136.38 | 134.69 |
| C 10 | -22.10 | -24.85 | -24.29 | -27.75 | -28.46 | -23.01 | -26.57 | -20.94 |
| C 11 | 111.21 | 111.75 | 118.97 | 116.48 | 112.88 | 113.00 | 117.12 | 113.10 |
| C 12 | 137.53 | 142.63 | 133.14 | 142.18 | 142.32 | 134.87 | 143.36 | 138.58 |
| C 13 | 139.41 | 146.68 | 137.97 | 140.42 | 133.01 | 136.43 | 137.88 | 144.21 |
| C 14 | 159.40 | 154.43 | 155.55 | 156.16 | 155.84 | 156.93 | 159.19 | 160.13 |
| C 15 | 40.21 | 36.24 | 36.41 | 36.77 | 38.05 | 37.68 | 39.41 | 39.36 |
| C 16 | 66.90 | 67.64 | 68.10 | 68.32 | 67.85 | 68.20 | 67.07 | 67.15 |
| C 17 | -0.59 | -0.29 | 0.68 | -1.22 | -0.30 | -0.28 | -0.33 | -0.35 |
| C 18 | 144.24 | 147.66 | 145.75 | 148.71 | 146.02 | 147.03 | 146.42 | 147.31 |
| C 19 | 149.05 | 153.48 | 148.43 | 147.43 | 147.59 | 147.28 | 147.27 | 147.35 |
| C 20 | -15.49 | -18.44 | -16.24 | -18.65 | -13.93 | -15.39 | -15.74 | -16.51 |
| C 21 | 124.69 | 130.26 | 123.04 | 128.92 | 126.79 | 132.63 | 128.70 | 134.54 |
| C 22 | 143.60 | 139.30 | 143.45 | 145.62 | 146.89 | 142.53 | 145.61 | 142.27 |
| C 23 | 64.98 | 73.75 | 65.37 | 71.51 | 69.87 | 72.39 | 68.24 | 71.78 |
| C 24 | 52.16 | 49.45 | 52.01 | 48.18 | 49.63 | 49.49 | 50.06 | 49.83 |
| C 25 | 156.07 | 165.33 | 157.22 | 166.35 | 154.00 | 168.19 | 153.98 | 168.54 |
| C 27 | 166.40 | 166.84 | 166.28 | 166.25 | 166.45 | 166.39 | 166.43 | 166.34 |
| C 28 | 162.16 | 152.18 | 162.82 | 162.48 | 173.83 | 177.67 | 172.50 | 175.32 |
| C 29 | 165.28 | 166.85 | 164.37 | 168.00 | 163.23 | 168.67 | 163.62 | 166.82 |
| | | | | | | | | |
| H 8a | 29.34 | 28.62 | 29.41 | 28.63 | 28.77 | 28.65 | 28.85 | 28.57 |
| H 8b | 28.86 | 29.54 | 28.74 | 29.27 | 29.27 | 29.49 | 29.11 | 29.61 |
| H 11 | 27.76 | 27.28 | 27.11 | 26.94 | 27.38 | 27.38 | 27.92 | 27.87 |
| H 13 | 29.66 | 29.44 | 29.69 | 30.05 | 29.32 | 29.53 | 28.79 | 28.74 |
| H 14a | 28.99 | 29.77 | 28.98 | 28.93 | 29.30 | 29.27 | 29.66 | 29.62 |
| H 14b | 29.50 | 28.62 | 29.13 | 29.25 | 28.93 | 28.91 | 29.26 | 29.58 |
| H 16 | 25.63 | 25.77 | 25.64 | 25.70 | 25.69 | 25.67 | 25.66 | 25.64 |
| H 18 | 28.49 | 29.00 | 29.44 | 29.05 | 29.11 | 29.24 | 29.18 | 29.27 |
| H 19a | 28.43 | 28.91 | 28.40 | 28.59 | 28.98 | 28.68 | 28.92 | 28.63 |
| H 19b | 29.78 | 29.33 | 29.83 | 29.17 | 29.28 | 29.61 | 29.33 | 29.60 |
| H 21 | 29.40 | 28.89 | 29.24 | 28.97 | 28.74 | 28.85 | 28.44 | 28.43 |
| H 23 | 25.43 | 26.12 | 25.59 | 25.64 | 25.42 | 24.81 | 25.24 | 24.76 |
| H 25a-c | 30.28 | 30.30 | 30.30 | 30.58 | 30.55 | 30.38 | 30.24 | 30.37 |
| H 27a-c | 29.91 | 30.06 | 29.87 | 29.90 | 29.90 | 29.91 | 29.91 | 29.93 |
| H 28a-c | 30.59 | 30.35 | 30.47 | 30.71 | 30.74 | 30.81 | 30.88 | 30.85 |
| H 29a-c | 30.18 | 30.55 | 30.45 | 30.50 | 30.37 | 30.64 | 30.45 | 30.39 |

| Isotropic shielding constants | | | | | | | | |
|---|---|---|---|---|---|---|---|---|
| Nuclei | Isomer 25 | Isomer 26 | Isomer 27 | Isomer 28 | Isomer 29 | Isomer 30 | Isomer 31 | Isomer 32 |
| C 7 | 0.72 | 4.61 | 2.19 | 5.74 | 3.48 | 3.20 | 2.20 | 5.15 |
| C 8 | 147.93 | 145.13 | 148.67 | 146.92 | 147.30 | 147.75 | 148.37 | 145.77 |
| C 9 | 131.20 | 133.56 | 132.08 | 133.77 | 135.09 | 135.84 | 132.44 | 134.77 |
| C 10 | -20.74 | -21.73 | -23.23 | -24.01 | -25.20 | -23.87 | -23.04 | -24.16 |
| C 11 | 108.75 | 109.86 | 116.12 | 115.73 | 114.16 | 112.55 | 110.62 | 111.05 |
| C 12 | 140.52 | 142.12 | 140.14 | 140.99 | 142.98 | 136.80 | 136.99 | 138.99 |
| C 13 | 138.66 | 139.66 | 137.96 | 138.02 | 140.99 | 139.34 | 148.92 | 149.08 |
| C 14 | 161.02 | 160.81 | 155.24 | 156.32 | 156.04 | 157.62 | 154.32 | 154.22 |
| C 15 | 39.04 | 39.79 | 38.50 | 37.84 | 38.72 | 37.10 | 37.24 | 37.31 |
| C 16 | 67.27 | 67.06 | 66.82 | 67.37 | 67.25 | 67.87 | 68.37 | 68.35 |
| C 17 | -0.77 | -1.07 | -0.15 | -0.09 | 1.76 | -0.72 | -0.48 | -0.57 |
| C 18 | 147.49 | 147.73 | 146.54 | 147.85 | 148.43 | 148.94 | 147.64 | 147.70 |
| C 19 | 145.91 | 145.78 | 151.99 | 149.91 | 154.17 | 146.55 | 149.94 | 149.96 |
| C 20 | -17.01 | -17.48 | -16.33 | -16.02 | -16.69 | -18.26 | -16.74 | -15.99 |
| C 21 | 140.40 | 138.49 | 137.38 | 133.66 | 131.91 | 126.61 | 131.57 | 128.85 |
| C 22 | 144.45 | 144.25 | 142.84 | 142.78 | 145.75 | 142.84 | 143.12 | 142.57 |
| C 23 | 67.99 | 70.22 | 66.62 | 67.70 | 69.27 | 67.09 | 71.36 | 68.40 |
| C 24 | 48.62 | 50.63 | 49.40 | 50.21 | 48.69 | 50.71 | 48.95 | 50.18 |
| C 25 | 162.37 | 168.32 | 164.61 | 169.32 | 168.28 | 167.47 | 163.61 | 166.62 |
| C 27 | 166.33 | 166.36 | 166.44 | 166.31 | 166.35 | 166.23 | 166.48 | 166.41 |
| C 28 | 165.31 | 167.32 | 162.40 | 164.46 | 169.06 | 170.84 | 164.32 | 165.86 |
| C 29 | 166.46 | 170.07 | 168.28 | 170.50 | 168.67 | 164.96 | 167.67 | 169.54 |
| | | | | | | | | |
| H 8a | 29.30 | 29.62 | 29.40 | 28.96 | 28.81 | 28.76 | 29.43 | 28.85 |
| H 8b | 28.76 | 27.99 | 28.56 | 29.60 | 29.05 | 29.42 | 28.55 | 29.58 |
| H 11 | 27.78 | 27.78 | 27.01 | 27.11 | 27.43 | 27.29 | 26.99 | 27.09 |
| H 13 | 29.91 | 29.95 | 29.57 | 29.75 | 29.72 | 29.62 | 29.07 | 29.34 |
| H 14a | 28.55 | 28.84 | 29.43 | 29.42 | 29.22 | 29.42 | 29.33 | 29.32 |
| H 14b | 29.65 | 29.68 | 28.84 | 28.68 | 29.17 | 28.87 | 28.20 | 28.52 |
| H 16 | 25.71 | 25.70 | 25.61 | 25.66 | 25.56 | 25.68 | 25.70 | 25.71 |
| H 18 | 28.68 | 28.60 | 29.38 | 29.34 | 29.15 | 29.04 | 29.21 | 29.23 |
| H 19a | 28.52 | 28.57 | 28.63 | 28.58 | 29.29 | 28.76 | 28.76 | 28.80 |
| H 19b | 29.43 | 29.38 | 29.54 | 29.56 | 28.38 | 29.15 | 29.29 | 29.22 |
| H 21 | 28.03 | 27.81 | 28.60 | 28.53 | 28.94 | 29.12 | 28.75 | 28.76 |
| H 23 | 25.24 | 25.41 | 24.83 | 25.02 | 25.13 | 26.11 | 25.46 | 25.96 |
| H 25a-c | 30.01 | 30.25 | 30.24 | 30.44 | 30.36 | 30.35 | 30.44 | 30.70 |
| H 27a-c | 29.93 | 29.93 | 29.89 | 29.90 | 29.91 | 29.92 | 29.98 | 29.96 |
| H 28a-c | 30.83 | 30.85 | 30.66 | 30.74 | 30.34 | 30.55 | 30.37 | 30.45 |
| H 29a-c | 30.25 | 30.51 | 30.48 | 30.41 | 30.43 | 30.30 | 30.43 | 30.45 |

| Isotropic shielding constants | | |
|---|---|---|
| Nuclei | Isomer 44 | Isomer 50 |
| C 7 | 2.50 | 3.65 |
| C 8 | 144.71 | 148.30 |
| C 9 | 137.14 | 136.30 |
| C 10 | -27.43 | -23.87 |
| C 11 | 109.88 | 112.01 |
| C 12 | 144.69 | 140.23 |
| C 13 | 141.10 | 154.55 |
| C 14 | 160.04 | 158.53 |
| C 15 | 37.62 | 37.69 |
| C 16 | 67.96 | 67.82 |
| C 17 | 1.65 | 1.44 |
| C 18 | 148.65 | 150.92 |
| C 19 | 150.68 | 153.56 |
| C 20 | -13.85 | -18.40 |
| C 21 | 131.15 | 129.59 |
| C 22 | 146.18 | 139.54 |
| C 23 | 69.38 | 76.55 |
| C 24 | 49.60 | 49.43 |
| C 25 | 153.34 | 165.17 |
| C 27 | 166.10 | 166.23 |
| C 28 | 173.80 | 159.64 |
| C 29 | 162.62 | 167.32 |
| | | |
| H 8a | 29.27 | 28.73 |
| H 8b | 28.72 | 29.55 |
| H 11 | 27.31 | 27.35 |
| H 13 | 28.98 | 29.39 |
| H 14a | 29.02 | 28.79 |
| H 14b | 29.35 | 29.47 |
| H 16 | 25.62 | 25.69 |
| H 18 | 28.73 | 28.93 |
| H 19a | 28.58 | 28.55 |
| H 19b | 29.33 | 29.43 |
| H 21 | 28.62 | 28.87 |
| H 23 | 25.08 | 26.14 |
| H 25a-c | 30.47 | 30.31 |
| H 27a-c | 29.91 | 29.98 |
| H 28a-c | 30.69 | 30.36 |
| H 29a-c | 30.28 | 30.54 |

| | Isomer 1 | Isomer 2 | Isomer 3 | Isomer 4 | Isomer 5 | Isomer 6 | Isomer 7 | Isomer 8 | Isomer 9 | Isomer 10 | Isomer 11 | Isomer 12 | Isomer 13 | Isomer 14 | Isomer 15 | Isomer 16 | Isomer 17 |
|---|---|---|---|---|---|---|---|---|---|---|---|---|---|---|---|---|---|
| $J_{13,14a}$ | 9.98 | 9.68 | 10.31 | 10.31 | 9.56 | 9.66 | 10.05 | 9.99 | 10.67 | 10.28 | 10.18 | 9.83 | 9.57 | 10.09 | 8.89 | 9.10 | 9.97 |
| $J_{13,14b}$ | 3.49 | 4.34 | 2.88 | 3.42 | 4.37 | 4.20 | 3.69 | 3.54 | 4.18 | 3.39 | 4.08 | 3.50 | 3.95 | 3.91 | 4.32 | 3.44 | 3.57 |
| $J_{13,18}$ | 12.27 | 13.43 | 11.74 | 12.19 | 12.06 | 12.07 | 12.24 | 12.23 | 12.43 | 11.97 | 12.42 | 11.94 | 12.41 | 12.46 | 12.60 | 12.63 | 12.20 |
| $J_{18,19a}$ | 12.69 | 12.45 | 12.98 | 9.72 | 11.73 | 11.35 | 11.97 | 11.68 | 9.52 | 10.80 | 10.63 | 10.98 | 10.43 | 7.47 | 9.71 | 11.08 | 12.91 |
| $J_{18,19b}$ | 4.25 | 4.61 | 3.89 | 7.84 | 4.99 | 5.33 | 4.85 | 5.19 | 6.71 | 4.14 | 4.86 | 3.83 | 5.79 | 10.33 | 7.13 | 6.14 | 3.89 |

| | Isomer 18 | Isomer 19 | Isomer 20 | Isomer 21 | Isomer 22 | Isomer 23 | Isomer 24 | Isomer 25 | Isomer 26 | Isomer 27 | Isomer 28 | Isomer 29 | Isomer 30 | Isomer 31 | Isomer 32 | Isomer 44 | Isomer 50 |
|---|---|---|---|---|---|---|---|---|---|---|---|---|---|---|---|---|---|
| $J_{13,14a}$ | 9.93 | 10.18 | 10.41 | 9.55 | 9.53 | 9.84 | 10.05 | 10.80 | 10.68 | 9.86 | 10.03 | 9.49 | 10.17 | 8.73 | 8.85 | 4.81 | 4.29 |
| $J_{13,14b}$ | 3.34 | 2.95 | 3.33 | 4.23 | 4.37 | 3.81 | 3.71 | 4.16 | 4.14 | 3.65 | 4.02 | 3.66 | 3.80 | 4.57 | 4.41 | 11.92 | 11.71 |
| $J_{13,18}$ | 13.18 | 11.66 | 12.04 | 12.17 | 12.08 | 12.32 | 12.29 | 12.34 | 12.39 | 12.46 | 12.47 | 13.07 | 12.43 | 12.54 | 12.56 | 8.96 | 8.55 |
| $J_{18,19a}$ | 13.28 | 13.20 | 10.08 | 10.00 | 11.80 | 10.46 | 12.01 | 9.58 | 9.42 | 11.81 | 11.08 | 3.83 | 8.88 | 11.08 | 10.52 | 12.24 | 14.99 |
| $J_{18,19b}$ | 3.92 | 3.35 | 7.27 | 6.32 | 4.92 | 6.10 | 4.80 | 6.23 | 6.85 | 3.22 | 4.15 | 10.58 | 8.36 | 6.13 | 6.42 | 5.04 | 4.17 |

**Table S61.** Studied Boltzmann averaged GIAO isotropic magnetic shielding constants (σ) and coupling constants of isomers calculated at the PCM/B3LYP/6-31G**//AMBER level of theory after elimination of conformations incompatible with experimental $^{3}J_{HH}$ of Zoaramine according to i*J*/d*J*-DP4.

| Isotropic shielding constants | | | | |
|---|---|---|---|---|
| Nuclei | Isomer 1 | Isomer 2 | Isomer 3 | Isomer 4 |
| C 1 | 145.9 | 146 | 143.1 | 142 |
| C 2 | 128.2 | 128.1 | 128.8 | 129.5 |
| C 3 | 145.4 | 145.4 | 144.3 | 143.3 |
| C 4 | 161.5 | 161.6 | 163.8 | 163.5 |
| C 5 | 154.8 | 154.7 | 154.4 | 152.1 |
| C 6 | 31.2 | 31.24 | 25.35 | 26.88 |
| C 7 | 76.07 | 75.18 | 79.6 | 75.11 |
| C 8 | 155.1 | 155.1 | 155.5 | 155.2 |
| C 9 | 139.5 | 139.5 | 138.8 | 139.7 |
| C 10 | -19.6 | -17 | -20.3 | -17.2 |
| C 11 | 115.6 | 114.7 | 116.2 | 114.7 |
| C 12 | 138.4 | 142.3 | 139 | 142.4 |
| C 13 | 138.6 | 146.1 | 137.7 | 146 |
| C 14 | 158 | 161 | 158.2 | 161 |
| C 15 | 32.75 | 33.77 | 33.2 | 33.84 |
| C 16 | 68.97 | 68.18 | 68.81 | 68.19 |
| C 17 | 3.36 | 3.23 | 3.5 | 3.23 |
| C 18 | 147.8 | 147.5 | 147.9 | 147.6 |
| C 19 | 147.7 | 147.6 | 147.7 | 147.7 |
| C 20 | -12.9 | -14.1 | -12.5 | -14 |
| C 21 | 133.2 | 135.7 | 133 | 135.8 |
| C 22 | 147 | 147.6 | 145.6 | 147.3 |
| C 23 | 143.5 | 142.7 | 145.5 | 142.2 |
| C 24 | 2.89 | 2.41 | 1.92 | 0.72 |
| C 25 | 164.1 | 163.1 | 167.8 | 163.2 |
| C 27 | 165.7 | 165.8 | 165.7 | 165.8 |
| C 28 | 178.3 | 175.9 | 177.3 | 176 |
| C 29 | 168.2 | 166.2 | 168.5 | 166.1 |
| C 30 | 166.2 | 166.1 | 166.1 | 165.1 |
| | | | | |
| H 1a | 28.88 | 28.91 | 28.59 | 28.86 |
| H 1b | 28.79 | 28.81 | 28.88 | 28.96 |
| H 2 | 28.04 | 28.07 | 28.27 | 28.38 |
| H 3a | 30.33 | 30.33 | 31.09 | 30.97 |
| H 3b | 30.23 | 30.23 | 29.91 | 29.94 |
| H 4 | 30.01 | 30.01 | 29.89 | 29.75 |
| H 5a | 29.31 | 29.3 | 29.84 | 29.78 |
| H 5b | 29.56 | 29.52 | 29.54 | 29.44 |
| H 8a | 29.26 | 29.25 | 29.05 | 29.31 |
| H 8b | 29.31 | 29.37 | 29.5 | 29.36 |
| H 11 | 27.76 | 28.2 | 27.58 | 28.23 |
| H 13 | 29.88 | 29.15 | 29.83 | 29.16 |
| H 14a | 29.45 | 29.85 | 29.45 | 29.87 |
| H 14b | 29.23 | 29.7 | 29.33 | 29.72 |
| H 16 | 25.9 | 25.88 | 25.9 | 25.88 |

| | | | | |
|---|---|---|---|---|
| H 18 | 29.52 | 29.53 | 29.52 | 29.52 |
| H 19a | 28.87 | 28.83 | 28.88 | 28.82 |
| H 19b | 29.87 | 29.84 | 29.84 | 29.84 |
| H 21 | 29.39 | 28.88 | 29.39 | 28.89 |
| H 23a | 28.85 | 28.68 | 29.58 | 28.72 |
| H 23b | 29.39 | 29.52 | 28.9 | 29.56 |
| H 25a-c | 30.68 | 30.63 | 30.67 | 30.63 |
| H 27a-c | 29.94 | 29.97 | 29.94 | 29.97 |
| H 28a-c | 31.05 | 31.07 | 31.07 | 31.09 |
| H 29a-c | 30.82 | 30.61 | 30.77 | 30.6 |
| H 30a-c | 30.96 | 30.95 | 30.95 | 30.91 |

| | Isomer 1 | Isomer 2 | Isomer 3 | Isomer 4 |
|---|---|---|---|---|
| $J_{3a,4}$ | 10.02 | 10.07 | 9.8 | 9.8 |
| $J_{3b,4}$ | 4.29 | 4.24 | 2.65 | 1.63 |
| $J_{3a,2}$ | 0.9 | 0.94 | 1.12 | 1.38 |
| $J_{3b,2}$ | 5.21 | 5.3 | 4.8 | 4.08 |
| $J_{4,5a}$ | 10.33 | 10.29 | 10.51 | 10.12 |
| $J_{4,5b}$ | 1.3 | 1.49 | 1.17 | 1.15 |
| $J_{13,14a}$ | 9.28 | 9.89 | 9.3 | 9.89 |
| $J_{13,14b}$ | 4.27 | 3.33 | 4.28 | 3.32 |
| $J_{13,18}$ | 11.4 | 11.57 | 11.39 | 11.56 |
| $J_{18,19a}$ | 4.78 | 4.65 | 4.86 | 4.66 |
| $J_{18,19b}$ | 10.85 | 11.05 | 10.73 | 11.04 |

**Table S62.** Studied Boltzmann averaged GIAO isotropic magnetic shielding constants (σ) and coupling constants of isomers calculated at the PCM/B3LYP/6-31G**//MM3 level of theory after elimination of conformations incompatible with experimental $^3J_{HH}$ of Zoaramine according to i$J$/d$J$-DP4.

| Isotropic shielding constants | | | | |
|---|---|---|---|---|
| Nuclei | Isomer 1 | Isomer 2 | Isomer 3 | Isomer 4 |
| C 1 | 137.30 | 137.34 | 142.23 | 141.97 |
| C 2 | 119.04 | 118.96 | 122.49 | 123.10 |
| C 3 | 138.70 | 138.93 | 141.29 | 141.28 |
| C 4 | 155.96 | 155.74 | 159.95 | 158.26 |
| C 5 | 152.86 | 152.74 | 152.19 | 153.46 |
| C 6 | 50.89 | 49.55 | 49.88 | 50.10 |
| C 7 | 89.29 | 88.95 | 87.64 | 91.66 |
| C 8 | 154.10 | 153.42 | 154.43 | 151.74 |
| C 9 | 139.10 | 138.98 | 139.28 | 138.30 |
| C 10 | -15.19 | -14.82 | -14.79 | -16.87 |
| C 11 | 113.20 | 114.36 | 113.28 | 114.55 |
| C 12 | 137.40 | 141.24 | 137.22 | 141.15 |
| C 13 | 134.72 | 143.63 | 134.74 | 143.97 |
| C 14 | 154.49 | 158.08 | 154.50 | 158.27 |
| C 15 | 36.70 | 38.71 | 36.75 | 39.05 |
| C 16 | 69.40 | 68.52 | 69.34 | 68.33 |
| C 17 | 5.95 | 5.79 | 5.96 | 6.04 |
| C 18 | 145.19 | 145.87 | 145.13 | 146.00 |
| C 19 | 145.70 | 145.70 | 145.80 | 145.37 |
| C 20 | -9.08 | -9.87 | -9.10 | -9.61 |
| C 21 | 131.55 | 134.72 | 131.54 | 135.01 |
| C 22 | 145.90 | 146.00 | 145.99 | 145.70 |
| C 23 | 141.87 | 142.85 | 140.96 | 146.25 |
| C 24 | 3.64 | 4.15 | 3.50 | 1.49 |
| C 25 | 160.89 | 161.01 | 160.42 | 162.48 |
| C 27 | 162.85 | 162.93 | 162.88 | 162.94 |
| C 28 | 174.36 | 172.14 | 174.52 | 172.24 |
| C 29 | 164.22 | 162.60 | 164.18 | 163.00 |
| C 30 | 162.63 | 162.70 | 162.81 | 163.01 |
| | | | | |
| H 1a | 28.02 | 28.03 | 27.91 | 27.95 |
| H 1b | 28.35 | 28.31 | 28.22 | 28.13 |
| H 2 | 27.94 | 27.96 | 27.58 | 27.61 |
| H 3a | 30.12 | 30.14 | 30.41 | 30.47 |
| H 3b | 29.22 | 29.28 | 29.20 | 29.30 |
| H 4 | 29.70 | 29.70 | 29.31 | 29.33 |
| H 5a | 29.46 | 29.51 | 29.47 | 29.66 |
| H 5b | 29.09 | 28.97 | 29.06 | 28.94 |
| H 8a | 28.77 | 28.75 | 28.81 | 28.57 |
| H 8b | 28.79 | 28.95 | 28.63 | 29.15 |
| H 11 | 27.26 | 27.86 | 27.25 | 27.72 |
| H 13 | 29.35 | 28.46 | 29.35 | 28.41 |
| H 14a | 28.86 | 29.20 | 28.86 | 29.22 |
| H 14b | 28.35 | 29.26 | 28.39 | 29.25 |
| H 16 | 25.60 | 25.55 | 25.60 | 25.53 |

| | | | | |
|---|---|---|---|---|
| H 18 | 28.92 | 28.97 | 28.91 | 29.01 |
| H 19a | 28.44 | 28.39 | 28.44 | 28.35 |
| H 19b | 29.33 | 29.31 | 29.31 | 29.31 |
| H 21 | 28.72 | 28.20 | 28.69 | 28.14 |
| H 23a | 28.42 | 28.63 | 28.18 | 29.27 |
| H 23b | 28.68 | 28.35 | 28.90 | 27.31 |
| H 25a-c | 30.04 | 30.02 | 30.01 | 30.09 |
| H 27a-c | 29.45 | 29.46 | 29.45 | 29.44 |
| H 28a-c | 30.34 | 30.44 | 30.32 | 30.47 |
| H 29a-c | 30.22 | 29.98 | 30.21 | 29.89 |
| H 30a-c | 30.27 | 30.28 | 30.30 | 30.32 |

| | Isomer 1 | Isomer 2 | Isomer 3 | Isomer 4 |
|---|---|---|---|---|
| $J_{3a,4}$ | 11.24 | 11.23 | 11.24 | 11.18 |
| $J_{3b,4}$ | 3.07 | 3.11 | 3.03 | 2.83 |
| $J_{3a,2}$ | 10.44 | 10.45 | 1.50 | 1.54 |
| $J_{3b,2}$ | 2.73 | 2.70 | 4.42 | 4.30 |
| $J_{4,5a}$ | 11.00 | 10.94 | 11.14 | 10.75 |
| $J_{4,5b}$ | 1.11 | 1.11 | 1.08 | 0.92 |
| $J_{13,14a}$ | 10.49 | 10.95 | 10.51 | 10.98 |
| $J_{13,14b}$ | 4.08 | 3.50 | 4.06 | 3.58 |
| $J_{13,18}$ | 12.68 | 13.10 | 12.66 | 13.10 |
| $J_{18,19a}$ | 5.01 | 4.77 | 5.06 | 4.62 |
| $J_{18,19b}$ | 12.22 | 12.42 | 12.18 | 12.57 |

**Table S63.** Studied Boltzmann averaged GIAO isotropic magnetic shielding constants (σ) and coupling constants of isomers calculated at the PCM/B3LYP/6-31G**//MMFF level of theory after elimination of conformations incompatible with experimental $^{3}J_{HH}$ of Zoaramine according to i*J*/d*J*-DP4.

| Isotropic shielding constants | | | |
|---|---|---|---|
| Nuclei | Isomer 1 | Isomer 3 | Isomer 4 |
| C 1 | 139.32 | 142.60 | 142.46 |
| C 2 | 118.86 | 126.21 | 126.48 |
| C 3 | 143.85 | 143.86 | 143.65 |
| C 4 | 157.89 | 162.34 | 162.15 |
| C 5 | 157.31 | 154.39 | 154.53 |
| C 6 | 50.96 | 47.34 | 46.82 |
| C 7 | 88.49 | 88.01 | 87.04 |
| C 8 | 153.88 | 155.23 | 154.80 |
| C 9 | 135.78 | 137.26 | 137.05 |
| C 10 | -23.84 | -22.95 | -22.42 |
| C 11 | 113.52 | 113.26 | 113.91 |
| C 12 | 135.71 | 135.47 | 139.35 |
| C 13 | 135.25 | 136.16 | 143.97 |
| C 14 | 157.28 | 157.05 | 160.43 |
| C 15 | 37.93 | 37.64 | 39.28 |
| C 16 | 68.12 | 68.24 | 67.28 |
| C 17 | -0.26 | -0.52 | -0.59 |
| C 18 | 147.25 | 147.02 | 147.50 |
| C 19 | 147.44 | 147.82 | 147.84 |
| C 20 | -15.23 | -15.34 | -16.68 |
| C 21 | 131.30 | 130.59 | 132.83 |
| C 22 | 139.86 | 143.10 | 143.20 |
| C 23 | 143.57 | 142.13 | 142.28 |
| C 24 | -11.83 | -8.21 | -8.56 |
| C 25 | 167.58 | 163.40 | 163.21 |
| C 27 | 166.20 | 166.31 | 166.28 |
| C 28 | 176.41 | 177.89 | 175.44 |
| C 29 | 167.42 | 166.87 | 164.96 |
| C 30 | 166.26 | 165.52 | 165.53 |
| | | | |
| H 1a | 28.48 | 28.43 | 28.43 |
| H 1b | 29.12 | 28.86 | 28.86 |
| H 2 | 28.02 | 28.14 | 28.12 |
| H 3a | 30.48 | 30.80 | 30.79 |
| H 3b | 29.91 | 29.75 | 29.73 |
| H 4 | 30.17 | 29.76 | 29.75 |
| H 5a | 29.83 | 29.88 | 29.92 |
| H 5b | 29.10 | 29.47 | 29.49 |
| H 8a | 28.93 | 28.75 | 28.72 |
| H 8b | 29.52 | 29.08 | 29.13 |
| H 11 | 27.33 | 27.44 | 28.01 |
| H 13 | 29.51 | 29.56 | 28.79 |
| H 14a | 29.27 | 29.28 | 29.62 |
| H 14b | 29.01 | 28.95 | 29.58 |
| H 16 | 25.68 | 25.70 | 25.65 |

| | | | |
|---|---|---|---|
| H 18 | 29.33 | 29.26 | 29.29 |
| H 19a | 28.79 | 28.77 | 28.71 |
| H 19b | 29.64 | 29.64 | 29.64 |
| H 21 | 29.00 | 29.01 | 28.59 |
| H 23a | 29.45 | 28.67 | 28.71 |
| H 23b | 28.05 | 29.10 | 29.00 |
| H 25a-c | 30.57 | 30.44 | 30.41 |
| H 27a-c | 29.91 | 29.91 | 29.94 |
| H 28a-c | 30.83 | 30.82 | 30.91 |
| H 29a-c | 30.71 | 30.68 | 30.43 |
| H 30a-c | 30.76 | 30.79 | 30.81 |

| | Isomer 1 | Isomer 3 | Isomer 4 |
|---|---|---|---|
| $J_{3a,4}$ | 10.85 | 10.41 | 10.4 |
| $J_{3b,4}$ | 3.15 | 2.32 | 2.24 |
| $J_{3a,2}$ | 9.97 | 1.7 | 1.71 |
| $J_{3b,2}$ | 2.59 | 4.12 | 4.08 |
| $J_{4,5a}$ | 10.79 | 10.18 | 10.1 |
| $J_{4,5b}$ | 1.04 | 0.75 | 0.74 |
| $J_{13,14a}$ | 9.53 | 9.53 | 10.04 |
| $J_{13,14b}$ | 4.39 | 4.36 | 3.71 |
| $J_{13,18}$ | 12.11 | 12.1 | 12.3 |
| $J_{18,19a}$ | 5.08 | 5.07 | 4.88 |
| $J_{18,19b}$ | 11.61 | 11.64 | 11.92 |

**Table S64.** Conformer contribution and energy for each conformer of Zoarenone at AMBER 12 kJ/mol MAD 0.5 Å.

| Name | Energy (Hartree) | Boltzmann population |
|---|---|---|
| **01-Zoarenone-miniAMBER-1** | -1305.323053 | 1.00 |
| **02-Zoarenone-miniAMBER-3** | -1305.31163 | 0.89 |
| **02-Zoarenone-miniAMBER-2** | -1305.309651 | 0.11 |
| **03-Zoarenone-miniAMBER-4** | -1305.330064 | 1.00 |
| **04-Zoarenone-miniAMBER-5** | -1305.316487 | 0.50 |
| **04-Zoarenone-miniAMBER-6** | -1305.316477 | 0.50 |
| **05-Zoarenone-miniAMBER-7** | -1305.338395 | 1.00 |
| **06-Zoarenone-miniAMBER-8** | -1305.33226 | 1.00 |
| **07-Zoarenone-miniAMBER-9** | -1305.330265 | 1.00 |
| **08-Zoarenone-miniAMBER-10** | -1305.322105 | 1.00 |
| **09-Zoarenone-miniAMBER-11** | -1305.326736 | 1.00 |
| **10-Zoarenone-miniAMBER-12** | -1305.29435 | 1.00 |
| **10-Zoarenone-miniAMBER-13** | -1305.288056 | 0.00 |
| **11-Zoarenone-miniAMBER-15** | -1305.334134 | 0.56 |
| **11-Zoarenone-miniAMBER-14** | -1305.333907 | 0.44 |
| **12-Zoarenone-miniAMBER-16** | -1305.306248 | 0.50 |
| **12-Zoarenone-miniAMBER-17** | -1305.306244 | 0.50 |
| **13-Zoarenone-miniAMBER-18** | -1305.333289 | 0.89 |
| **13-Zoarenone-miniAMBER-19** | -1305.331356 | 0.11 |
| **13-Zoarenone-miniAMBER-21** | -1305.325584 | 0.00 |
| **13-Zoarenone-miniAMBER-20** | -1305.323866 | 0.00 |
| **14-Zoarenone-miniAMBER-22** | -1305.318539 | 1.00 |
| **15-Zoarenone-miniAMBER-23** | -1305.332735 | 1.00 |
| **15-Zoarenone-miniAMBER-24** | -1305.319699 | 0.00 |
| **16-Zoarenone-miniAMBER-25** | -1305.307784 | 0.02 |
| **16-Zoarenone-miniAMBER-27** | -1305.310674 | 0.49 |
| **16-Zoarenone-miniAMBER-28** | -1305.310668 | 0.48 |
| **16-Zoarenone-miniAMBER-26** | -1305.306743 | 0.01 |
| **17-Zoarenone-miniAMBER-29** | -1305.309995 | 1.00 |
| **18-Zoarenone-miniAMBER-30** | -1305.317711 | 0.00 |
| **18-Zoarenone-miniAMBER-32** | -1305.323627 | 1.00 |
| **18-Zoarenone-miniAMBER-33** | -1305.316273 | 0.00 |
| **18-Zoarenone-miniAMBER-31** | -1305.314881 | 0.00 |
| **19-Zoarenone-miniAMBER-34** | -1305.318288 | 1.00 |
| **20-Zoarenone-miniAMBER-36** | -1305.327062 | 0.85 |
| **20-Zoarenone-miniAMBER-35** | -1305.325427 | 0.15 |
| **21-Zoarenone-miniAMBER-37** | -1305.313078 | 1.00 |
| **22-Zoarenone-miniAMBER-38** | -1305.342592 | 1.00 |
| **23-Zoarenone-miniAMBER-39** | -1305.308134 | 0.50 |
| **23-Zoarenone-miniAMBER-40** | -1305.308132 | 0.50 |

| | | |
|---|---|---|
| **24-Zoarenone-miniAMBER-41** | -1305.334492 | 1.00 |
| **25-Zoarenone-miniAMBER-42** | -1305.310445 | 1.00 |
| **26-Zoarenone-miniAMBER-43** | -1305.322907 | 1.00 |
| **27-Zoarenone-miniAMBER-44** | -1305.323627 | 1.00 |
| **28-Zoarenone-miniAMBER-46** | -1305.330489 | 0.38 |
| **28-Zoarenone-miniAMBER-47** | -1305.330493 | 0.38 |
| **28-Zoarenone-miniAMBER-45** | -1305.330026 | 0.23 |
| **29-Zoarenone-miniAMBER-48** | -1305.321281 | 0.52 |
| **29-Zoarenone-miniAMBER-49** | -1305.320564 | 0.24 |
| **29-Zoarenone-miniAMBER-50** | -1305.320558 | 0.24 |
| **30-Zoarenone-miniAMBER-52** | -1305.330551 | 0.44 |
| **30-Zoarenone-miniAMBER-51** | -1305.330475 | 0.41 |
| **30-Zoarenone-miniAMBER-53** | -1305.329515 | 0.15 |
| **31-Zoarenone-miniAMBER-54** | -1305.319891 | 1.00 |
| **32-Zoarenone-miniAMBER-55** | -1305.326373 | 0.93 |
| **32-Zoarenone-miniAMBER-58** | -1305.323942 | 0.07 |
| **32-Zoarenone-miniAMBER-59** | -1305.319862 | 0.00 |
| **32-Zoarenone-miniAMBER-56** | -1305.31858 | 0.00 |
| **32-Zoarenone-miniAMBER-57** | -1305.317424 | 0.00 |
| **44-Zoarenone-miniAMBER-86** | -1305.301367 | 1.00 |

**Table S65.** Conformer contribution and energy for each conformer of Zoarenone at MM3 12 kJ/mol MAD 0.5 Å.

| Name | Energy (Hartree) | Boltzmann population |
|---|---|---|
| **01-Zoarenone-miniMM3-1** | -1305.320444 | 1.00 |
| **02-Zoarenone-miniMM3-2** | -1305.307115 | 1.00 |
| **03-Zoarenone-miniMM3-4** | -1305.327047 | 1.00 |
| **04-Zoarenone-miniMM3-5** | -1305.31292 | 0.50 |
| **04-Zoarenone-miniMM3-6** | -1305.312907 | 0.50 |
| **05-Zoarenone-miniMM3-7** | -1305.33366 | 1.00 |
| **06-Zoarenone-miniMM3-8** | -1305.328427 | 1.00 |
| **07-Zoarenone-miniMM3-9** | -1305.326132 | 1.00 |
| **08-Zoarenone-miniMM3-10** | -1305.318217 | 1.00 |
| **09-Zoarenone-miniMM3-11** | -1305.323895 | 1.00 |
| **10-Zoarenone-miniMM3-12** | -1305.291683 | 1.00 |
| **11-Zoarenone-miniMM3-15** | -1305.331098 | 0.69 |
| **11-Zoarenone-miniMM3-14** | -1305.330352 | 0.31 |
| **12-Zoarenone-miniMM3-16** | -1305.303093 | 0.50 |
| **12-Zoarenone-miniMM3-17** | -1305.303084 | 0.50 |
| **13-Zoarenone-miniMM3-18** | -1305.328782 | 0.79 |
| **13-Zoarenone-miniMM3-19** | -1305.327531 | 0.21 |
| **14-Zoarenone-miniMM3-22** | -1305.31669 | 1.00 |
| **15-Zoarenone-miniMM3-23** | -1305.329589 | 1.00 |
| **16-Zoarenone-miniMM3-26** | -1305.307152 | 0.15 |
| **16-Zoarenone-miniMM3-27** | -1305.308043 | 0.40 |
| **16-Zoarenone-miniMM3-28** | -1305.308024 | 0.39 |
| **16-Zoarenone-miniMM3-25** | -1305.306288 | 0.06 |
| **17-Zoarenone-miniMM3-29** | -1305.307729 | 1.00 |
| **18-Zoarenone-miniMM3-32** | -1305.320046 | 1.00 |
| **19-Zoarenone-miniMM3-34** | -1305.315546 | 1.00 |
| **20-Zoarenone-miniMM3-36** | -1305.323203 | 0.97 |
| **20-Zoarenone-miniMM3-35** | -1305.320054 | 0.03 |
| **21-Zoarenone-miniMM3-37** | -1305.309738 | 1.00 |
| **22-Zoarenone-miniMM3-38** | -1305.338858 | 1.00 |
| **23-Zoarenone-miniMM3-39** | -1305.304663 | 0.50 |
| **23-Zoarenone-miniMM3-40** | -1305.30466 | 0.50 |
| **24-Zoarenone-miniMM3-41** | -1305.330693 | 1.00 |
| **25-Zoarenone-miniMM3-42** | -1305.307577 | 1.00 |
| **26-Zoarenone-miniMM3-43** | -1305.319341 | 1.00 |
| **27-Zoarenone-miniMM3-44** | -1305.320078 | 1.00 |
| **28-Zoarenone-miniMM3-46** | -1305.326036 | 0.39 |
| **28-Zoarenone-miniMM3-47** | -1305.326027 | 0.38 |
| **28-Zoarenone-miniMM3-45** | -1305.325539 | 0.23 |
| **29-Zoarenone-miniMM3-48** | -1305.317592 | 0.83 |

| | | |
|---|---|---|
| **29-Zoarenone-miniMM3-49** | -1305.316113 | 0.17 |
| **30-Zoarenone-miniMM3-51** | -1305.3281 | 0.84 |
| **30-Zoarenone-miniMM3-52** | -1305.326185 | 0.11 |
| **30-Zoarenone-miniMM3-53** | -1305.325342 | 0.05 |
| **31-Zoarenone-miniMM3-54** | -1305.317372 | 1.00 |
| **32-Zoarenone-miniMM3-55** | -1305.32239 | 0.84 |
| **32-Zoarenone-miniMM3-58** | -1305.320852 | 0.16 |
| **34-Zoarenone-miniMM3-65** | -1305.312407 | 1.00 |
| **44-Zoarenone-miniMM3-86** | -1305.298507 | 1.00 |
| **50-Zoarenone-miniMM3-106** | -1305.320197 | 0.50 |
| **50-Zoarenone-miniMM3-108** | -1305.320182 | 0.50 |

**Table S66.** Conformer contribution and energy for each conformer of Zoarenone at MMFF 12 kJ/mol MAD 0.5 Å.

| Name | Energy (Hartree) | Boltzmann population |
|---|---|---|
| **01-Zoarenone-miniMMFF-1** | -1305.326685 | 1.00 |
| **02-Zoarenone-miniMMFF-3** | -1305.314846 | 0.80 |
| **02-Zoarenone-miniMMFF-2** | -1305.313526 | 0.20 |
| **03-Zoarenone-miniMMFF-4** | -1305.333759 | 1.00 |
| **04-Zoarenone-miniMMFF-5** | -1305.319694 | 0.96 |
| **04-Zoarenone-miniMMFF-6** | -1305.316777 | 0.04 |
| **05-Zoarenone-miniMMFF-7** | -1305.340318 | 1.00 |
| **06-Zoarenone-miniMMFF-8** | -1305.334962 | 1.00 |
| **07-Zoarenone-miniMMFF-9** | -1305.332915 | 1.00 |
| **08-Zoarenone-miniMMFF-10** | -1305.324948 | 1.00 |
| **09-Zoarenone-miniMMFF-11** | -1305.331161 | 1.00 |
| **10-Zoarenone-miniMMFF-12** | -1305.298992 | 1.00 |
| **11-Zoarenone-miniMMFF-14** | -1305.33799 | 0.65 |
| **11-Zoarenone-miniMMFF-15** | -1305.337423 | 0.35 |
| **12-Zoarenone-miniMMFF-16** | -1305.30967 | 0.94 |
| **12-Zoarenone-miniMMFF-17** | -1305.307135 | 0.06 |
| **13-Zoarenone-miniMMFF-18** | -1305.335927 | 0.85 |
| **13-Zoarenone-miniMMFF-19** | -1305.334323 | 0.15 |
| **14-Zoarenone-miniMMFF-22** | -1305.322619 | 1.00 |
| **15-Zoarenone-miniMMFF-23** | -1305.335405 | 1.00 |
| **16-Zoarenone-miniMMFF-25** | -1305.31289 | 0.15 |
| **16-Zoarenone-miniMMFF-28** | -1305.314094 | 0.53 |
| **16-Zoarenone-miniMMFF-27** | -1305.313247 | 0.22 |
| **16-Zoarenone-miniMMFF-26** | -1305.312506 | 0.10 |
| **17-Zoarenone-miniMMFF-29** | -1305.313152 | 1.00 |
| **18-Zoarenone-miniMMFF-32** | -1305.326817 | 1.00 |
| **19-Zoarenone-miniMMFF-34** | -1305.321009 | 1.00 |
| **20-Zoarenone-miniMMFF-36** | -1305.330344 | 0.89 |
| **20-Zoarenone-miniMMFF-35** | -1305.328379 | 0.11 |
| **21-Zoarenone-miniMMFF-37** | -1305.316429 | 1.00 |
| **22-Zoarenone-miniMMFF-38** | -1305.345542 | 1.00 |
| **23-Zoarenone-miniMMFF-39** | -1305.311763 | 0.98 |
| **23-Zoarenone-miniMMFF-40** | -1305.308122 | 0.02 |
| **24-Zoarenone-miniMMFF-41** | -1305.337329 | 1.00 |
| **25-Zoarenone-miniMMFF-42** | -1305.314281 | 1.00 |
| **26-Zoarenone-miniMMFF-43** | -1305.326466 | 1.00 |
| **27-Zoarenone-miniMMFF-44** | -1305.326533 | 1.00 |
| **28-Zoarenone-miniMMFF-46** | -1305.332671 | 0.38 |
| **28-Zoarenone-miniMMFF-47** | -1305.332512 | 0.32 |
| **28-Zoarenone-miniMMFF-45** | -1305.332464 | 0.30 |

| | | |
|---|---|---|
| **29-Zoarenone-miniMMFF-48** | -1305.324371 | 0.81 |
| **29-Zoarenone-miniMMFF-49** | -1305.323007 | 0.19 |
| **30-Zoarenone-miniMMFF-51** | -1305.334629 | 0.89 |
| **30-Zoarenone-miniMMFF-52** | -1305.332286 | 0.07 |
| **30-Zoarenone-miniMMFF-53** | -1305.331615 | 0.04 |
| **31-Zoarenone-miniMMFF-54** | -1305.32337 | 1.00 |
| **32-Zoarenone-miniMMFF-55** | -1305.328862 | 0.91 |
| **32-Zoarenone-miniMMFF-58** | -1305.32669 | 0.09 |
| **44-Zoarenone-miniMMFF-86** | -1305.305464 | 1.00 |
| **50-Zoarenone-miniMMFF-106** | -1305.327043 | 1.00 |

**Table S67.** Conformer contribution and energy for each conformer of Zoaramine at AMBER 12 kJ/mol MAD 0.5 Å.

| Name | Energy (Hartree) | Boltzmann population |
|---|---|---|
| **01-Zoaramine_miniAMBER_RRSRSRSRS_5** | -1634.676535 | 0.11 |
| **01-Zoaramine_miniAMBER_RRSRSRSRS_13** | -1634.678448 | 0.85 |
| **01-Zoaramine_miniAMBER_RRSRSRSRS_8** | -1634.675508 | 0.04 |
| **02-Zoaramine_miniAMBER_RRSSSRSRS_69** | -1634.670451 | 0.95 |
| **02-Zoaramine_miniAMBER_RRSSSRSRS_65** | -1634.667714 | 0.05 |
| **03-Zoaramine_miniAMBER_SRSRSRSRS_144** | -1634.683399 | 0.64 |
| **03-Zoaramine_miniAMBER_SRSRSRSRS_145** | -1634.682855 | 0.36 |
| **04-Zoaramine_miniAMBER_SRSSSRSRS_191** | -1634.674918 | 0.96 |
| **04-Zoaramine_miniAMBER_SRSSSRSRS_192** | -1634.671802 | 0.04 |

**Table S68.** Conformer contribution and energy for each conformer of Zoaramine at MM3 12 kJ/mol MAD 0.5 Å.

| Name | Energy (Hartree) | Boltzmann population |
|---|---|---|
| **01-Zoaramine_miniMM3_RRSRSRSRS_3** | -1634.672988 | 0.02 |
| **01-Zoaramine_miniMM3_RRSRSRSRS_10** | -1634.675938 | 0.42 |
| **01-Zoaramine_miniMM3_RRSRSRSRS_14** | -1634.675314 | 0.21 |
| **01-Zoaramine_miniMM3_RRSRSRSRS_11** | -1634.675094 | 0.17 |
| **01-Zoaramine_miniMM3_RRSRSRSRS_17** | -1634.674628 | 0.10 |
| **01-Zoaramine_miniMM3_RRSRSRSRS_19** | -1634.673821 | 0.04 |
| **01-Zoaramine_miniMM3_RRSRSRSRS_7** | -1634.673233 | 0.02 |
| **01-Zoaramine_miniMM3_RRSRSRSRS_4** | -1634.67234 | 0.01 |
| **02-Zoaramine_miniMM3_RRSSSRSRS_66** | -1634.668543 | 0.27 |
| **02-Zoaramine_miniMM3_RRSSSRSRS_74** | -1634.66856 | 0.27 |
| **02-Zoaramine_miniMM3_RRSSSRSRS_71** | -1634.668305 | 0.21 |
| **02-Zoaramine_miniMM3_RRSSSRSRS_67** | -1634.667926 | 0.14 |
| **02-Zoaramine_miniMM3_RRSSSRSRS_75** | -1634.667761 | 0.12 |
| **02-Zoaramine_miniMM3_RRSSSRSRS_68** | -1634.662837 | 0.00 |
| **02-Zoaramine_miniMM3_RRSSSRSRS_127** | -1634.662415 | 0.00 |
| **02-Zoaramine_miniMM3_RRSSSRSRS_62** | -1634.661927 | 0.00 |
| **03-Zoaramine_miniMM3_SRSRSRSRS_145** | -1634.676646 | 0.97 |
| **03-Zoaramine_miniMM3_SRSRSRSRS_190** | -1634.673419 | 0.03 |
| **03-Zoaramine_miniMM3_SRSRSRSRS_166** | -1634.65836 | 0.00 |
| **04-Zoaramine_miniMM3_SRSSSRSRS_226** | -1634.670259 | 1.00 |
| **04-Zoaramine_miniMM3_SRSSSRSRS_192** | -1634.663147 | 0.00 |

**Table S69.** Conformer contribution and energy for each conformer of Zoaramine at MMFF 12 kJ/mol MAD 0.5 Å.

| Name | Energy (Hartree) | Boltzmann population |
|---|---|---|
| **01-Zoaramine_miniMMFF_RRSRSRSRS_3** | -1634.6807 | 0.69 |
| **01-Zoaramine_miniMMFF_RRSRSRSRS_4** | -1634.68 | 0.31 |
| **03-Zoaramine_miniMMFF_SRSRSRSRS_145** | -1634.6858 | 0.90 |
| **03-Zoaramine_miniMMFF_SRSRSRSRS_144** | -1634.6837 | 0.10 |
| **04-Zoaramine_miniMMFF_SRSSSRSRS_191** | -1634.6782 | 0.99 |
| **04-Zoaramine_miniMMFF_SRSSSRSRS_192** | -1634.6733 | 0.01 |

**Table S70.** CMAE (corrected mean absolute error) values for the isomers of Zoarenone at the PCM/B3LYP/6-31G**//AMBER

| | Absoluted Error Scaled | | | | | | | | | | | | | | | | |
|---|---|---|---|---|---|---|---|---|---|---|---|---|---|---|---|---|---|
| | Isomer1 | Isomer2 | Isomer3 | Isomer4 | Isomer5 | Isomer6 | Isomer7 | Isomer8 | Isomer9 | Isomer10 | Isomer11 | Isomer12 | Isomer13 | Isomer14 | Isomer15 | Isomer16 | Isomer17 |
| C 7 | 3.04 | 3.24 | 4.19 | 4.65 | 6.23 | 3.61 | 4.27 | 2.65 | 3.81 | 3.81 | 4.21 | 4.26 | 4.36 | 4.07 | 3.08 | 4.51 | 2.35 |
| C 8 | 2.07 | 0.07 | 1.59 | 2.76 | 2.08 | 1.81 | 4.51 | 2.58 | 2.22 | 2.05 | 1.55 | 1.81 | 4.69 | 2.13 | 1.74 | 3.62 | 1.88 |
| C 9 | 1.17 | 1.63 | 1.34 | 1.27 | 2.72 | 4.74 | 2.75 | 4.97 | 1.10 | 2.24 | 1.38 | 1.82 | 0.50 | 0.98 | 0.02 | 2.04 | 0.07 |
| C 10 | 2.44 | 6.84 | 5.31 | 7.13 | 4.25 | 4.64 | 1.17 | 1.85 | 0.55 | 6.32 | 5.26 | 9.38 | 6.82 | 5.25 | 5.25 | 6.06 | 3.88 |
| C 11 | 1.10 | 1.36 | 9.54 | 6.54 | 2.08 | 3.44 | 0.31 | 1.04 | 2.06 | 3.16 | 3.56 | 3.99 | 0.67 | 2.91 | 1.64 | 0.33 | 1.58 |
| C 12 | 5.61 | 9.41 | 0.72 | 10.54 | 1.49 | 1.25 | 4.05 | 4.62 | 7.98 | 15.94 | 5.46 | 13.98 | 5.44 | 3.63 | 3.91 | 10.84 | 7.74 |
| C 13 | 6.44 | 12.38 | 4.89 | 4.40 | 0.35 | 0.01 | 5.94 | 6.24 | 3.45 | 2.36 | 3.71 | 3.89 | 6.27 | 1.93 | 15.18 | 13.52 | 5.62 |
| C 14 | 4.65 | 2.22 | 0.42 | 2.24 | 1.72 | 2.13 | 3.38 | 3.80 | 5.38 | 1.43 | 0.07 | 1.32 | 1.39 | 5.71 | 0.47 | 1.96 | 6.45 |
| C 15 | 2.71 | 2.08 | 1.07 | 1.05 | 1.04 | 1.75 | 1.24 | 1.51 | 2.12 | 0.02 | 0.25 | 1.52 | 1.27 | 1.04 | 0.95 | 0.04 | 2.40 |
| C 16 | 0.24 | 1.17 | 2.08 | 0.98 | 0.06 | 0.69 | 1.11 | 0.10 | 0.65 | 0.45 | 0.22 | 0.38 | 0.65 | 1.56 | 0.06 | 1.48 | 0.76 |
| C 17 | 4.52 | 5.34 | 6.97 | 6.10 | 5.94 | 6.51 | 5.38 | 6.13 | 4.97 | 5.25 | 5.57 | 5.38 | 7.08 | 6.41 | 5.35 | 6.51 | 4.59 |
| C 18 | 0.64 | 5.68 | 1.05 | 5.17 | 3.42 | 3.63 | 2.18 | 2.48 | 2.90 | 2.50 | 3.34 | 1.61 | 3.67 | 7.89 | 2.90 | 2.09 | 1.02 |
| C 19 | 4.09 | 7.59 | 3.10 | 0.42 | 0.29 | 1.37 | 0.36 | 1.00 | 0.73 | 7.93 | 2.83 | 7.66 | 1.71 | 1.84 | 3.24 | 7.45 | 5.76 |
| C 20 | 2.49 | 3.18 | 3.35 | 2.86 | 0.71 | 0.04 | 2.55 | 1.65 | 4.82 | 2.94 | 2.31 | 1.15 | 0.70 | 2.58 | 1.82 | 4.68 | 1.57 |
| C 21 | 1.44 | 5.30 | 2.85 | 1.89 | 0.93 | 5.36 | 3.57 | 7.41 | 8.64 | 7.30 | 6.34 | 5.03 | 2.33 | 1.81 | 1.19 | 0.56 | 1.54 |
| C 22 | 0.33 | 2.97 | 0.28 | 2.23 | 2.03 | 1.49 | 2.09 | 1.00 | 2.01 | 2.60 | 3.17 | 2.90 | 0.19 | 0.99 | 3.04 | 4.07 | 2.40 |
| C 23 | 15.40 | 11.72 | 13.67 | 11.09 | 11.81 | 13.45 | 11.05 | 14.29 | 10.87 | 14.18 | 8.06 | 12.14 | 9.59 | 13.94 | 5.66 | 9.89 | 15.49 |
| C 24 | 0.75 | 2.31 | 1.21 | 2.51 | 2.36 | 1.87 | 2.52 | 2.21 | 2.91 | 0.23 | 2.73 | 0.60 | 2.62 | 1.23 | 2.54 | 1.77 | 0.86 |
| C 25 | 2.51 | 5.73 | 2.53 | 4.97 | 1.71 | 2.93 | 0.18 | 4.94 | 2.69 | 10.64 | 1.36 | 12.01 | 1.31 | 9.25 | 0.05 | 13.34 | 9.31 |
| C 27 | 3.42 | 3.02 | 3.29 | 0.89 | 0.41 | 1.17 | 0.08 | 0.89 | 0.60 | 3.56 | 0.71 | 2.67 | 0.72 | 2.75 | 1.17 | 3.26 | 4.70 |
| C 28 | 12.52 | 25.23 | 11.28 | 15.50 | 1.85 | 1.52 | 4.40 | 4.52 | 11.74 | 16.88 | 12.71 | 14.49 | 8.27 | 5.55 | 11.33 | 15.09 | 13.26 |
| C 29 | 2.92 | 0.73 | 1.93 | 0.96 | 2.84 | 1.58 | 2.08 | 0.36 | 2.87 | 2.90 | 0.02 | 4.03 | 0.16 | 0.48 | 1.18 | 1.49 | 0.79 |
| **Average** | **3.66** | **5.42** | **3.76** | **4.37** | **2.56** | **2.95** | **2.96** | **3.47** | **3.87** | **5.21** | **3.40** | **5.09** | **3.20** | **3.82** | **3.26** | **5.21** | **4.27** |

| | | | | | | | | | | | | | | | | | |
|---|---|---|---|---|---|---|---|---|---|---|---|---|---|---|---|---|---|
| H 8a | 0.56 | 0.18 | 0.42 | 0.18 | 0.78 | 0.26 | 0.86 | 0.10 | 0.71 | 0.41 | 0.53 | 0.45 | 0.17 | 0.20 | 0.66 | 0.24 | 0.43 |
| H 8b | 0.64 | 0.21 | 0.50 | 0.39 | 0.36 | 0.16 | 1.23 | 0.11 | 0.48 | 0.33 | 0.38 | 0.38 | 0.13 | 0.16 | 0.40 | 0.29 | 0.34 |
| H 11 | 0.34 | 0.10 | 0.14 | 0.05 | 0.14 | 0.08 | 0.64 | 0.64 | 0.65 | 0.48 | 0.11 | 0.13 | 0.11 | 0.09 | 0.00 | 0.01 | 0.50 |
| H 13 | 0.67 | 0.32 | 0.66 | 0.93 | 0.26 | 0.29 | 0.42 | 0.37 | 0.87 | 0.40 | 0.57 | 0.26 | 0.07 | 0.70 | 0.44 | 0.35 | 0.60 |
| H 14a | 0.31 | 0.23 | 0.51 | 0.19 | 0.21 | 0.15 | 0.24 | 0.28 | 0.50 | 0.55 | 0.01 | 0.11 | 0.07 | 0.07 | 0.04 | 0.28 | 0.57 |
| H 14b | 0.70 | 0.33 | 0.22 | 0.30 | 0.15 | 0.28 | 0.53 | 0.44 | 0.83 | 0.25 | 0.13 | 0.02 | 0.15 | 0.00 | 0.15 | 0.31 | 0.68 |
| H 16 | 0.75 | 0.16 | 0.56 | 0.15 | 0.15 | 0.09 | 0.22 | 0.23 | 0.01 | 0.02 | 0.10 | 0.04 | 0.19 | 0.33 | 0.48 | 0.20 | 0.39 |
| H 18 | 0.89 | 0.02 | 0.01 | 0.04 | 0.07 | 0.10 | 0.12 | 0.11 | 0.61 | 0.13 | 0.12 | 0.20 | 0.07 | 0.10 | 0.11 | 0.16 | 0.75 |
| H 19a | 0.29 | 0.09 | 0.36 | 0.35 | 0.31 | 0.23 | 0.28 | 0.24 | 0.37 | 0.10 | 0.31 | 0.12 | 0.35 | 0.30 | 0.03 | 0.23 | 0.35 |
| H 19b | 0.25 | 0.19 | 0.28 | 0.17 | 0.27 | 0.31 | 0.31 | 0.30 | 0.21 | 0.05 | 0.16 | 0.03 | 0.06 | 0.14 | 0.23 | 0.05 | 0.38 |
| H 21 | 0.92 | 0.32 | 0.70 | 0.27 | 0.00 | 0.30 | 0.36 | 0.03 | 0.50 | 0.15 | 0.16 | 0.13 | 0.79 | 0.63 | 0.39 | 0.41 | 0.90 |
| H 23 | 0.05 | 0.45 | 0.24 | 0.15 | 0.15 | 0.17 | 0.18 | 0.29 | 0.47 | 0.40 | 0.17 | 0.30 | 0.01 | 0.05 | 0.22 | 0.19 | 0.36 |
| H 25a-c | 0.27 | 0.09 | 0.16 | 0.19 | 0.06 | 0.37 | 0.02 | 0.37 | 0.07 | 0.22 | 0.23 | 0.03 | 0.12 | 0.17 | 0.21 | 0.14 | 0.13 |
| H 27a-c | 0.04 | 0.11 | 0.01 | 0.02 | 0.11 | 0.04 | 0.01 | 0.02 | 0.01 | 0.04 | 0.08 | 0.05 | 0.12 | 0.03 | 0.12 | 0.11 | 0.01 |
| H 28a-c | 0.13 | 0.65 | 0.26 | 0.33 | 0.20 | 0.15 | 0.07 | 0.05 | 0.18 | 0.17 | 0.28 | 0.37 | 0.26 | 0.42 | 0.39 | 0.61 | 0.30 |
| H 29a-c | 0.25 | 0.04 | 0.04 | 0.10 | 0.14 | 0.07 | 0.04 | 0.22 | 0.12 | 0.02 | 0.03 | 0.17 | 0.06 | 0.12 | 0.16 | 0.08 | 0.32 |
| **Average** | **0.44** | **0.22** | **0.32** | **0.24** | **0.21** | **0.19** | **0.35** | **0.24** | **0.41** | **0.23** | **0.21** | **0.17** | **0.17** | **0.22** | **0.25** | **0.23** | **0.44** |

| | Absoluted Error Scaled | | | | | | | | | | | | | | | |
|---|---|---|---|---|---|---|---|---|---|---|---|---|---|---|---|---|
| | Isomer18 | Isomer19 | Isomer20 | Isomer21 | Isomer22 | Isomer23 | Isomer24 | Isomer25 | Isomer26 | Isomer27 | Isomer28 | Isomer29 | Isomer30 | Isomer31 | Isomer32 | Isomer44 |
| C 7 | 3.32 | 3.60 | 4.77 | 4.57 | 4.37 | 4.07 | 3.34 | 2.17 | 4.45 | 3.34 | 6.02 | 4.84 | 4.58 | 2.89 | 5.04 | 4.10 |
| C 8 | 0.69 | 1.23 | 4.36 | 1.72 | 1.93 | 3.03 | 2.96 | 1.72 | 4.89 | 1.09 | 2.27 | 3.05 | 2.35 | 1.11 | 3.36 | 3.51 |
| C 9 | 1.77 | 0.43 | 2.24 | 2.10 | 1.77 | 0.93 | 2.12 | 4.19 | 1.93 | 4.04 | 2.24 | 2.79 | 0.51 | 3.89 | 0.49 | 1.39 |
| C 10 | 6.13 | 6.94 | 7.52 | 8.92 | 4.26 | 7.00 | 1.48 | 1.21 | 0.99 | 5.39 | 5.05 | 6.18 | 5.30 | 5.25 | 5.30 | 7.97 |
| C 11 | 0.18 | 10.82 | 5.70 | 3.15 | 1.32 | 6.29 | 0.58 | 1.94 | 2.68 | 5.70 | 4.51 | 1.95 | 1.24 | 0.37 | 1.14 | 1.52 |
| C 12 | 9.15 | 2.51 | 7.74 | 8.94 | 0.07 | 9.72 | 3.51 | 8.40 | 8.04 | 6.67 | 6.43 | 8.46 | 5.22 | 3.65 | 4.06 | 11.16 |
| C 13 | 12.72 | 5.11 | 4.89 | 0.81 | 0.16 | 3.21 | 6.92 | 2.87 | 2.44 | 4.00 | 3.16 | 4.65 | 5.19 | 15.62 | 13.94 | 6.40 |
| C 14 | 0.74 | 2.12 | 1.15 | 2.14 | 1.33 | 4.94 | 3.76 | 6.77 | 5.25 | 0.54 | 0.47 | 0.67 | 1.46 | 0.38 | 0.44 | 4.61 |
| C 15 | 0.42 | 0.42 | 0.63 | 1.84 | 0.93 | 2.62 | 1.42 | 2.32 | 2.01 | 1.69 | 0.66 | 1.67 | 0.91 | 1.35 | 0.71 | 0.90 |
| C 16 | 0.71 | 1.97 | 1.32 | 1.28 | 0.60 | 0.16 | 0.91 | 0.08 | 1.11 | 0.09 | 0.60 | 0.02 | 0.35 | 1.44 | 0.68 | 0.36 |
| C 17 | 4.97 | 6.64 | 6.37 | 6.34 | 6.03 | 6.07 | 5.45 | 5.83 | 4.66 | 5.80 | 5.26 | 7.65 | 6.58 | 5.74 | 5.09 | 7.28 |
| C 18 | 4.20 | 3.20 | 4.08 | 3.83 | 2.97 | 3.46 | 2.23 | 4.07 | 3.02 | 2.71 | 2.72 | 3.51 | 4.20 | 4.29 | 3.49 | 4.17 |
| C 19 | 8.09 | 4.90 | 1.07 | 2.34 | 0.08 | 1.38 | 0.73 | 0.36 | 1.03 | 6.27 | 4.18 | 5.10 | 1.81 | 3.61 | 2.55 | 6.24 |
| C 20 | 2.99 | 2.28 | 2.65 | 0.68 | 1.18 | 2.66 | 2.74 | 3.40 | 4.75 | 1.18 | 2.00 | 1.72 | 2.11 | 1.47 | 1.28 | 0.65 |
| C 21 | 1.44 | 3.01 | 1.19 | 0.69 | 3.03 | 1.06 | 4.71 | 11.96 | 8.81 | 9.68 | 5.10 | 2.58 | 2.21 | 2.64 | 0.83 | 2.67 |
| C 22 | 4.39 | 2.11 | 1.21 | 3.26 | 3.12 | 1.24 | 3.57 | 0.10 | 1.42 | 1.58 | 2.49 | 0.66 | 0.73 | 0.97 | 1.67 | 1.29 |
| C 23 | 6.50 | 14.91 | 8.03 | 11.04 | 7.96 | 12.78 | 9.41 | 14.32 | 9.96 | 15.59 | 12.75 | 13.91 | 10.68 | 11.09 | 10.59 | 12.75 |
| C 24 | 2.41 | 1.65 | 3.42 | 0.59 | 2.48 | 0.54 | 2.92 | 2.35 | 1.93 | 1.70 | 2.08 | 2.52 | 1.21 | 2.43 | 1.89 | 1.15 |
| C 25 | 0.06 | 7.80 | 0.22 | 12.09 | 1.90 | 12.58 | 1.58 | 5.44 | 0.64 | 3.24 | 1.97 | 3.74 | 0.12 | 5.29 | 0.54 | 14.09 |
| C 27 | 2.36 | 4.27 | 1.14 | 2.77 | 0.69 | 2.08 | 0.05 | 1.83 | 0.47 | 1.64 | 0.86 | 0.65 | 1.00 | 2.11 | 1.34 | 1.19 |
| C 28 | 23.87 | 12.52 | 15.47 | 2.30 | 0.84 | 4.44 | 3.91 | 11.95 | 11.40 | 14.58 | 14.10 | 9.08 | 7.76 | 12.72 | 12.15 | 4.48 |
| C 29 | 0.70 | 0.65 | 0.54 | 3.71 | 0.35 | 4.20 | 2.71 | 0.24 | 2.29 | 1.51 | 3.18 | 0.60 | 0.57 | 0.88 | 2.79 | 5.64 |
| **Average** | **4.44** | **4.50** | **3.90** | **3.87** | **2.15** | **4.29** | **3.04** | **4.25** | **3.83** | **4.45** | **4.01** | **3.91** | **3.00** | **4.05** | **3.61** | **4.71** |
| H 8a | 0.17 | 0.50 | 0.03 | 0.03 | 0.03 | 0.02 | 0.10 | 0.52 | 0.91 | 0.57 | 0.03 | 0.05 | 0.16 | 0.67 | 0.06 | 0.51 |

| | | | | | | | | | | | | | | | | |
|---|---|---|---|---|---|---|---|---|---|---|---|---|---|---|---|---|
| H 8b | 0.24 | 0.54 | 0.04 | 0.06 | 0.11 | 0.08 | 0.26 | 0.41 | 1.13 | 0.64 | 0.39 | 0.16 | 0.12 | 0.60 | 0.44 | 0.34 |
| H 11 | 0.03 | 0.05 | 0.38 | 0.14 | 0.34 | 0.70 | 0.69 | 0.61 | 0.57 | 0.02 | 0.04 | 0.31 | 0.01 | 0.10 | 0.24 | 0.21 |
| H 13 | 0.26 | 0.58 | 0.87 | 0.10 | 0.31 | 0.38 | 0.39 | 0.92 | 0.93 | 0.42 | 0.45 | 0.50 | 0.30 | 0.02 | 0.12 | 0.07 |
| H 14a | 0.32 | 0.27 | 0.31 | 0.06 | 0.18 | 0.31 | 0.22 | 0.72 | 0.58 | 0.09 | 0.00 | 0.07 | 0.05 | 0.03 | 0.13 | 0.25 |
| H 14b | 0.02 | 0.11 | 0.22 | 0.11 | 0.09 | 0.31 | 0.57 | 0.86 | 0.85 | 0.03 | 0.09 | 0.29 | 0.06 | 0.17 | 0.10 | 0.58 |
| H 16 | 0.56 | 0.30 | 0.18 | 0.11 | 0.04 | 0.15 | 0.10 | 0.11 | 0.18 | 0.02 | 0.01 | 0.23 | 0.56 | 0.07 | 0.38 | 0.02 |
| H 18 | 0.29 | 0.16 | 0.26 | 0.03 | 0.03 | 0.02 | 0.06 | 0.45 | 0.57 | 0.30 | 0.19 | 0.07 | 0.03 | 0.20 | 0.08 | 0.22 |
| H 19a | 0.08 | 0.38 | 0.38 | 0.05 | 0.30 | 0.11 | 0.33 | 0.38 | 0.38 | 0.14 | 0.24 | 0.14 | 0.30 | 0.05 | 0.12 | 0.16 |
| H 19b | 0.12 | 0.38 | 0.04 | 0.06 | 0.24 | 0.08 | 0.26 | 0.37 | 0.32 | 0.24 | 0.19 | 0.43 | 0.04 | 0.08 | 0.02 | 0.07 |
| H 21 | 0.37 | 0.70 | 0.51 | 0.26 | 0.29 | 0.06 | 0.14 | 0.44 | 0.79 | 0.09 | 0.16 | 0.49 | 0.56 | 0.37 | 0.08 | 0.22 |
| H 23 | 0.26 | 0.10 | 0.19 | 0.12 | 0.32 | 0.32 | 0.42 | 0.36 | 0.11 | 0.30 | 0.17 | 0.31 | 0.29 | 0.16 | 0.35 | 0.30 |
| H 25a-c | 0.03 | 0.21 | 0.13 | 0.05 | 0.15 | 0.19 | 0.14 | 0.37 | 0.01 | 0.28 | 0.02 | 0.19 | 0.01 | 0.05 | 0.36 | 0.04 |
| H 27a-c | 0.12 | 0.05 | 0.08 | 0.03 | 0.10 | 0.02 | 0.06 | 0.11 | 0.08 | 0.04 | 0.11 | 0.02 | 0.02 | 0.18 | 0.07 | 0.06 |
| H 28a-c | 0.54 | 0.41 | 0.25 | 0.18 | 0.29 | 0.07 | 0.22 | 0.03 | 0.04 | 0.31 | 0.34 | 0.43 | 0.22 | 0.46 | 0.49 | 0.18 |
| H 29a-c | 0.11 | 0.11 | 0.04 | 0.16 | 0.01 | 0.07 | 0.17 | 0.13 | 0.13 | 0.08 | 0.19 | 0.05 | 0.03 | 0.05 | 0.03 | 0.15 |
| **Average** | **0.22** | **0.30** | **0.25** | **0.10** | **0.18** | **0.18** | **0.26** | **0.42** | **0.47** | **0.22** | **0.16** | **0.23** | **0.17** | **0.20** | **0.19** | **0.21** |

**Table S71.** CMAE (corrected mean absolute error) values for the isomers of Zoarenone at the PCM/B3LYP/6-31G**//MM3

| | Absoluted Error Scaled | | | | | | | | | | | | | | | | | |
|---|---|---|---|---|---|---|---|---|---|---|---|---|---|---|---|---|---|---|
| | Isomer1 | Isomer2 | Isomer3 | Isomer4 | Isomer5 | Isomer6 | Isomer7 | Isomer8 | Isomer9 | Isomer10 | Isomer11 | Isomer12 | Isomer13 | Isomer14 | Isomer15 | Isomer16 | Isomer17 | Isomer18 |
| C 7 | 1.41 | 1.98 | 2.45 | 2.44 | 4.17 | 1.42 | 1.94 | 0.18 | 2.27 | 1.34 | 2.48 | 1.65 | 2.04 | 2.09 | 1.60 | 1.27 | 0.13 | 1.37 |
| C 8 | 2.30 | 3.45 | 1.31 | 2.96 | 2.52 | 1.16 | 4.72 | 2.42 | 2.65 | 2.32 | 1.49 | 2.19 | 4.37 | 1.42 | 1.76 | 2.75 | 1.47 | 0.55 |
| C 9 | 2.14 | 0.47 | 2.28 | 0.94 | 1.22 | 1.91 | 1.64 | 2.07 | 0.24 | 4.37 | 0.06 | 4.00 | 1.27 | 2.24 | 0.45 | 3.94 | 2.11 | 2.42 |
| C 10 | 3.79 | 6.68 | 5.78 | 7.85 | 4.27 | 4.79 | 2.07 | 3.07 | 1.72 | 7.91 | 5.50 | 10.52 | 7.01 | 5.60 | 5.87 | 7.52 | 5.21 | 6.84 |
| C 11 | 0.27 | 3.07 | 8.22 | 5.78 | 0.78 | 1.92 | 0.68 | 0.01 | 3.24 | 3.95 | 2.76 | 2.92 | 0.74 | 1.90 | 2.66 | 1.94 | 0.54 | 0.72 |
| C 12 | 5.36 | 12.34 | 1.08 | 8.88 | 1.44 | 0.68 | 4.03 | 4.10 | 7.24 | 17.57 | 5.20 | 15.50 | 4.81 | 2.45 | 3.84 | 9.92 | 6.17 | 8.93 |
| C 13 | 4.01 | 3.10 | 2.66 | 3.69 | 2.59 | 2.48 | 5.16 | 5.10 | 1.57 | 1.39 | 1.25 | 0.74 | 5.12 | 0.22 | 13.98 | 12.21 | 4.70 | 10.68 |
| C 14 | 4.88 | 3.49 | 0.29 | 0.44 | 0.84 | 1.02 | 2.70 | 3.15 | 4.50 | 2.48 | 1.13 | 1.47 | 0.07 | 5.01 | 1.97 | 0.38 | 6.25 | 1.31 |
| C 15 | 5.18 | 5.33 | 0.59 | 2.02 | 2.21 | 2.92 | 3.37 | 3.82 | 3.68 | 2.49 | 2.18 | 4.20 | 2.73 | 2.68 | 1.81 | 2.41 | 4.90 | 2.01 |
| C 16 | 1.19 | 1.35 | 0.85 | 0.31 | 0.95 | 0.42 | 2.44 | 1.56 | 1.91 | 0.77 | 1.77 | 1.71 | 0.47 | 0.11 | 1.15 | 0.38 | 0.87 | 0.57 |
| C 17 | 2.85 | 2.94 | 5.12 | 4.27 | 4.12 | 4.59 | 3.51 | 4.26 | 3.25 | 3.46 | 3.54 | 3.62 | 5.50 | 4.17 | 3.54 | 4.87 | 2.85 | 3.51 |
| C 18 | 0.43 | 5.37 | 3.95 | 5.75 | 2.91 | 2.92 | 2.27 | 2.56 | 2.92 | 1.91 | 2.49 | 0.86 | 3.53 | 8.78 | 2.06 | 4.61 | 2.14 | 4.42 |
| C 19 | 3.57 | 1.11 | 2.07 | 0.68 | 0.87 | 1.47 | 0.17 | 0.69 | 1.55 | 7.71 | 3.18 | 7.44 | 2.24 | 1.01 | 2.82 | 7.12 | 5.06 | 7.75 |
| C 20 | 2.78 | 4.75 | 3.50 | 3.80 | 1.61 | 1.24 | 3.18 | 2.43 | 5.71 | 4.47 | 3.86 | 2.83 | 1.65 | 4.01 | 2.69 | 4.40 | 2.93 | 4.81 |
| C 21 | 2.09 | 1.80 | 4.17 | 0.22 | 0.31 | 4.85 | 3.12 | 6.71 | 9.66 | 6.70 | 6.80 | 4.39 | 2.06 | 3.17 | 1.33 | 2.75 | 2.17 | 1.24 |
| C 22 | 1.15 | 3.89 | 1.39 | 5.67 | 0.76 | 1.16 | 0.50 | 0.43 | 0.50 | 6.46 | 0.36 | 6.72 | 2.91 | 2.97 | 0.04 | 5.83 | 3.85 | 2.12 |
| C 23 | 12.66 | 6.64 | 11.62 | 5.30 | 8.58 | 8.51 | 7.33 | 9.17 | 7.59 | 9.24 | 4.52 | 6.88 | 6.62 | 8.52 | 2.27 | 5.03 | 11.17 | 2.96 |
| C 24 | 4.88 | 1.81 | 5.20 | 1.42 | 1.86 | 1.93 | 1.56 | 1.46 | 1.86 | 4.38 | 1.66 | 3.37 | 1.61 | 5.13 | 2.12 | 1.76 | 5.74 | 1.96 |
| C 25 | 1.32 | 1.40 | 1.30 | 4.18 | 0.80 | 3.19 | 1.03 | 5.73 | 2.14 | 11.91 | 0.80 | 12.91 | 0.55 | 8.92 | 0.04 | 15.55 | 10.61 | 0.08 |
| C 27 | 3.25 | 0.72 | 3.04 | 0.62 | 0.62 | 1.15 | 0.10 | 0.56 | 0.42 | 3.69 | 0.66 | 2.92 | 0.52 | 2.70 | 0.92 | 2.86 | 4.25 | 2.52 |
| C 28 | 13.35 | 21.23 | 11.14 | 17.16 | 3.13 | 3.06 | 5.80 | 5.84 | 12.77 | 18.96 | 13.90 | 17.93 | 9.43 | 8.13 | 11.95 | 13.08 | 14.68 | 26.01 |
| C 29 | 2.00 | 0.32 | 2.98 | 0.10 | 3.17 | 0.71 | 2.01 | 0.71 | 2.65 | 4.58 | 0.50 | 4.85 | 0.55 | 1.48 | 1.51 | 3.79 | 0.41 | 1.00 |
| **Average** | **3.68** | **4.24** | **3.68** | **3.84** | **2.26** | **2.43** | **2.70** | **3.00** | **3.64** | **5.82** | **3.00** | **5.44** | **2.99** | **3.76** | **3.02** | **5.20** | **4.46** | **4.26** |

| | | | | | | | | | | | | | | | | | | |
|---|---|---|---|---|---|---|---|---|---|---|---|---|---|---|---|---|---|---|
| H 8a | 0.60 | 0.20 | 0.52 | 0.21 | 0.81 | 0.35 | 0.98 | 0.25 | 0.70 | 0.41 | 0.54 | 0.44 | 0.17 | 0.29 | 0.69 | 0.39 | 0.65 | 0.05 |
| H 8b | 0.45 | 0.26 | 0.32 | 0.34 | 0.46 | 0.31 | 1.79 | 0.26 | 0.36 | 0.27 | 0.27 | 0.32 | 0.03 | 0.34 | 0.22 | 0.20 | 0.47 | 0.25 |
| H 11 | 0.25 | 0.69 | 0.46 | 0.16 | 0.00 | 0.15 | 0.74 | 0.71 | 0.71 | 0.61 | 0.12 | 0.02 | 0.37 | 0.16 | 0.14 | 0.24 | 0.51 | 0.21 |
| H 13 | 0.66 | 0.98 | 0.65 | 0.93 | 0.32 | 0.32 | 0.60 | 0.60 | 0.82 | 0.39 | 0.50 | 0.18 | 0.10 | 0.74 | 0.65 | 0.02 | 0.57 | 0.21 |
| H 14a | 0.19 | 0.87 | 1.12 | 0.38 | 0.25 | 0.22 | 0.19 | 0.20 | 0.58 | 0.70 | 0.01 | 0.15 | 0.15 | 0.19 | 0.05 | 0.15 | 0.49 | 0.21 |
| H 14b | 0.57 | 0.80 | 0.25 | 0.34 | 0.10 | 0.07 | 0.70 | 0.56 | 0.85 | 0.23 | 0.29 | 0.06 | 0.02 | 0.13 | 0.42 | 0.65 | 0.56 | 0.39 |
| H 16 | 0.95 | 0.31 | 0.68 | 0.28 | 0.17 | 0.20 | 0.30 | 0.41 | 0.05 | 0.09 | 0.14 | 0.09 | 0.19 | 0.56 | 0.40 | 0.26 | 0.55 | 0.58 |
| H 18 | 1.31 | 1.34 | 0.07 | 0.02 | 0.06 | 0.08 | 0.15 | 0.11 | 0.71 | 0.05 | 0.10 | 0.11 | 0.02 | 0.21 | 0.02 | 0.12 | 1.10 | 0.59 |
| H 19a | 0.40 | 0.34 | 0.48 | 0.35 | 0.16 | 0.10 | 0.17 | 0.19 | 0.37 | 0.14 | 0.23 | 0.18 | 0.21 | 0.27 | 0.07 | 0.17 | 0.46 | 0.15 |
| H 19b | 0.44 | 0.20 | 0.53 | 0.10 | 0.33 | 0.33 | 0.37 | 0.34 | 0.28 | 0.08 | 0.21 | 0.01 | 0.01 | 0.06 | 0.32 | 0.08 | 0.54 | 0.16 |
| H 21 | 1.00 | 0.31 | 0.85 | 0.14 | 0.08 | 0.09 | 0.56 | 0.35 | 0.82 | 0.33 | 0.04 | 0.05 | 0.71 | 0.48 | 0.34 | 0.42 | 0.84 | 0.27 |
| H 23 | 0.28 | 0.20 | 0.47 | 0.12 | 0.00 | 0.13 | 0.08 | 0.07 | 0.36 | 0.35 | 0.06 | 0.12 | 0.17 | 0.39 | 0.38 | 0.16 | 0.15 | 0.52 |
| H 25a-c | 0.34 | 0.25 | 0.21 | 0.03 | 0.03 | 0.25 | 0.09 | 0.22 | 0.03 | 0.24 | 0.21 | 0.07 | 0.17 | 0.13 | 0.35 | 0.10 | 0.01 | 0.09 |
| H 27a-c | 0.18 | 0.31 | 0.15 | 0.12 | 0.02 | 0.08 | 0.14 | 0.14 | 0.12 | 0.18 | 0.05 | 0.07 | 0.01 | 0.15 | 0.29 | 0.35 | 0.12 | 0.35 |
| H 28a-c | 0.15 | 0.11 | 0.32 | 0.35 | 0.22 | 0.15 | 0.02 | 0.00 | 0.15 | 0.17 | 0.28 | 0.41 | 0.28 | 0.49 | 0.32 | 0.46 | 0.28 | 0.48 |
| H 29a-c | 0.20 | 0.19 | 0.10 | 0.05 | 0.04 | 0.01 | 0.16 | 0.22 | 0.06 | 0.06 | 0.12 | 0.10 | 0.05 | 0.00 | 0.28 | 0.01 | 0.29 | 0.29 |
| **Average** | **0.50** | **0.46** | **0.45** | **0.24** | **0.19** | **0.18** | **0.44** | **0.29** | **0.44** | **0.27** | **0.20** | **0.15** | **0.17** | **0.29** | **0.31** | **0.23** | **0.47** | **0.30** |

| | Absoluted Error Scaled | | | | | | | | | | | | | | | | |
|---|---|---|---|---|---|---|---|---|---|---|---|---|---|---|---|---|---|
| | Isomer19 | Isomer20 | Isomer21 | Isomer22 | Isomer23 | Isomer24 | Isomer25 | Isomer26 | Isomer27 | Isomer28 | Isomer29 | Isomer30 | Isomer31 | Isomer32 | Isomer34 | Isomer44 | Isomer50 |
| C 7 | 1.43 | 3.13 | 2.13 | 2.71 | 1.56 | 1.79 | 0.43 | 1.95 | 0.94 | 3.86 | 2.59 | 2.65 | 0.74 | 3.12 | 1.11 | 1.77 | 0.81 |
| C 8 | 0.62 | 4.59 | 2.28 | 1.84 | 3.82 | 3.74 | 1.20 | 4.62 | 0.55 | 2.70 | 3.49 | 1.80 | 0.55 | 4.14 | 0.92 | 3.90 | 2.47 |
| C 9 | 1.91 | 2.47 | 4.49 | 0.08 | 3.17 | 0.77 | 1.40 | 1.29 | 1.18 | 0.78 | 0.60 | 2.14 | 0.83 | 0.74 | 0.67 | 3.39 | 1.06 |
| C 10 | 7.34 | 7.92 | 9.78 | 4.32 | 8.41 | 2.82 | 2.33 | 1.80 | 5.67 | 5.26 | 6.76 | 4.54 | 5.64 | 5.79 | 5.54 | 8.82 | 6.09 |
| C 11 | 9.79 | 4.51 | 1.94 | 0.07 | 6.28 | 0.48 | 3.09 | 3.91 | 4.50 | 3.65 | 1.11 | 0.06 | 1.70 | 2.33 | 0.71 | 2.93 | 2.10 |
| C 12 | 1.59 | 7.99 | 10.19 | 0.43 | 11.14 | 2.90 | 7.42 | 7.51 | 6.29 | 6.39 | 7.24 | 3.43 | 2.95 | 4.86 | 5.01 | 12.29 | 5.22 |
| C 13 | 3.95 | 3.83 | 3.50 | 2.24 | 1.32 | 6.08 | 1.12 | 0.95 | 0.79 | 0.49 | 3.10 | 2.11 | 12.92 | 12.20 | 15.48 | 4.78 | 17.79 |
| C 14 | 1.52 | 0.27 | 1.31 | 0.08 | 4.24 | 2.78 | 5.88 | 4.58 | 1.35 | 1.06 | 1.25 | 2.08 | 1.20 | 1.40 | 1.26 | 3.69 | 0.81 |
| C 15 | 0.78 | 1.64 | 3.20 | 1.98 | 4.47 | 3.38 | 4.04 | 3.64 | 4.07 | 2.90 | 3.28 | 1.98 | 2.11 | 1.81 | 1.71 | 2.34 | 1.91 |
| C 16 | 0.68 | 0.07 | 0.32 | 0.64 | 1.07 | 2.29 | 1.13 | 2.23 | 1.63 | 1.88 | 1.37 | 0.18 | 0.04 | 0.24 | 1.88 | 1.39 | 2.36 |
| C 17 | 4.70 | 4.55 | 4.46 | 4.19 | 4.05 | 3.57 | 4.10 | 2.95 | 3.71 | 3.39 | 6.38 | 5.01 | 3.90 | 3.71 | 5.51 | 5.65 | 4.84 |
| C 18 | 5.69 | 5.74 | 3.36 | 2.24 | 3.05 | 1.81 | 4.06 | 3.25 | 1.99 | 2.41 | 3.00 | 6.17 | 3.55 | 3.51 | 6.09 | 4.11 | 5.70 |
| C 19 | 4.15 | 0.33 | 2.86 | 0.20 | 1.75 | 0.64 | 0.56 | 1.55 | 6.20 | 4.45 | 7.07 | 0.14 | 2.95 | 2.63 | 7.55 | 5.60 | 6.62 |
| C 20 | 3.31 | 3.24 | 1.48 | 2.48 | 3.24 | 3.71 | 4.28 | 5.36 | 2.89 | 3.37 | 3.12 | 3.88 | 2.63 | 1.92 | 4.83 | 1.73 | 5.35 |
| C 21 | 4.16 | 1.55 | 2.95 | 3.15 | 0.83 | 5.08 | 12.01 | 9.14 | 9.89 | 4.59 | 2.27 | 3.35 | 1.88 | 2.10 | 2.20 | 1.46 | 1.08 |
| C 22 | 3.72 | 3.96 | 6.90 | 0.02 | 4.63 | 1.51 | 2.23 | 0.99 | 0.88 | 0.32 | 3.07 | 1.14 | 1.99 | 1.46 | 1.34 | 5.04 | 3.24 |
| C 23 | 11.20 | 5.37 | 6.13 | 4.38 | 8.12 | 5.82 | 8.70 | 5.79 | 10.30 | 9.45 | 9.65 | 8.75 | 5.00 | 7.95 | 5.72 | 7.46 | 0.03 |
| C 24 | 6.31 | 0.99 | 3.60 | 1.88 | 3.58 | 1.68 | 1.43 | 2.17 | 2.09 | 2.16 | 1.28 | 4.02 | 1.57 | 2.29 | 1.24 | 2.95 | 1.42 |
| C 25 | 9.31 | 0.27 | 13.00 | 1.16 | 13.56 | 0.64 | 6.05 | 0.63 | 3.26 | 1.06 | 2.38 | 0.44 | 5.31 | 1.53 | 4.18 | 15.13 | 1.69 |
| C 27 | 4.09 | 0.87 | 2.96 | 0.52 | 2.03 | 0.42 | 1.54 | 0.62 | 1.52 | 1.09 | 0.11 | 1.61 | 1.77 | 1.36 | 1.06 | 1.53 | 0.57 |
| C 28 | 13.23 | 16.74 | 4.05 | 1.64 | 6.28 | 4.82 | 13.54 | 13.14 | 16.69 | 15.88 | 11.48 | 7.60 | 13.20 | 12.58 | 21.77 | 6.26 | 19.93 |
| C 29 | 1.13 | 0.02 | 4.56 | 0.01 | 5.93 | 2.71 | 1.12 | 2.59 | 0.65 | 3.63 | 0.17 | 2.76 | 0.32 | 2.31 | 0.65 | 6.98 | 2.41 |
| **Average** | **4.57** | **3.64** | **4.34** | **1.65** | **4.66** | **2.70** | **3.99** | **3.67** | **3.96** | **3.67** | **3.67** | **2.99** | **3.31** | **3.64** | **4.38** | **4.96** | **4.25** |

| | | | | | | | | | | | | | | | | | |
|---|---|---|---|---|---|---|---|---|---|---|---|---|---|---|---|---|---|
| H 8a | 0.69 | 0.05 | 0.02 | 0.06 | 0.04 | 0.01 | 0.71 | 1.11 | 0.70 | 0.08 | 0.10 | 0.13 | 0.88 | 0.13 | 0.23 | 0.54 | 0.19 |
| H 8b | 0.59 | 0.10 | 0.04 | 0.12 | 0.12 | 0.23 | 0.51 | 1.69 | 0.70 | 0.43 | 0.17 | 0.12 | 0.63 | 0.52 | 0.48 | 0.25 | 0.32 |
| H 11 | 0.36 | 0.76 | 0.02 | 0.14 | 0.77 | 0.73 | 0.64 | 0.63 | 0.23 | 0.13 | 0.10 | 0.31 | 0.29 | 0.48 | 0.11 | 0.06 | 0.17 |
| H 13 | 0.56 | 0.87 | 0.16 | 0.33 | 0.43 | 0.61 | 0.94 | 0.94 | 0.36 | 0.41 | 0.70 | 0.53 | 0.17 | 0.06 | 0.06 | 0.12 | 0.33 |
| H 14a | 0.66 | 0.62 | 0.13 | 0.22 | 0.23 | 0.13 | 0.96 | 0.68 | 0.10 | 0.02 | 0.15 | 0.05 | 0.12 | 0.02 | 0.62 | 0.38 | 0.97 |
| H 14b | 0.11 | 0.34 | 0.14 | 0.21 | 0.38 | 0.69 | 0.92 | 0.92 | 0.05 | 0.20 | 0.32 | 0.33 | 0.89 | 0.74 | 0.60 | 0.57 | 0.63 |
| H 16 | 0.39 | 0.11 | 0.17 | 0.07 | 0.27 | 0.22 | 0.30 | 0.27 | 0.08 | 0.01 | 0.34 | 0.70 | 0.12 | 0.44 | 0.07 | 0.10 | 0.74 |
| H 18 | 0.21 | 0.24 | 0.08 | 0.00 | 0.03 | 0.03 | 0.56 | 0.64 | 0.24 | 0.16 | 0.02 | 0.11 | 0.14 | 0.05 | 0.16 | 0.35 | 0.65 |
| H 19a | 0.52 | 0.36 | 0.12 | 0.16 | 0.04 | 0.23 | 0.39 | 0.37 | 0.05 | 0.15 | 0.50 | 0.37 | 0.10 | 0.06 | 0.03 | 0.11 | 0.34 |
| H 19b | 0.57 | 0.00 | 0.03 | 0.29 | 0.05 | 0.28 | 0.44 | 0.42 | 0.23 | 0.21 | 0.92 | 0.11 | 0.08 | 0.03 | 0.09 | 0.14 | 0.02 |
| H 21 | 0.68 | 0.42 | 0.12 | 0.17 | 0.25 | 0.35 | 0.88 | 1.12 | 0.09 | 0.28 | 0.31 | 0.59 | 0.30 | 0.12 | 0.20 | 0.00 | 0.35 |
| H 23 | 0.17 | 0.38 | 0.08 | 0.13 | 0.22 | 0.30 | 0.07 | 0.08 | 0.01 | 0.01 | 0.10 | 0.59 | 0.17 | 0.71 | 0.37 | 0.08 | 0.59 |
| H 25a-c | 0.06 | 0.23 | 0.17 | 0.13 | 0.17 | 0.12 | 0.22 | 0.06 | 0.13 | 0.06 | 0.01 | 0.01 | 0.15 | 0.47 | 0.20 | 0.17 | 0.19 |
| H 27a-c | 0.05 | 0.05 | 0.10 | 0.01 | 0.09 | 0.05 | 0.26 | 0.23 | 0.08 | 0.01 | 0.18 | 0.14 | 0.37 | 0.26 | 0.19 | 0.19 | 0.29 |
| H 28a-c | 0.47 | 0.24 | 0.21 | 0.29 | 0.09 | 0.19 | 0.09 | 0.09 | 0.31 | 0.36 | 0.53 | 0.31 | 0.36 | 0.37 | 0.63 | 0.22 | 0.39 |
| H 29a-c | 0.02 | 0.08 | 0.09 | 0.08 | 0.02 | 0.14 | 0.09 | 0.29 | 0.05 | 0.09 | 0.02 | 0.03 | 0.14 | 0.20 | 0.02 | 0.07 | 0.35 |
| **Average** | **0.38** | **0.30** | **0.11** | **0.15** | **0.20** | **0.27** | **0.50** | **0.60** | **0.21** | **0.16** | **0.28** | **0.28** | **0.31** | **0.29** | **0.25** | **0.21** | **0.41** |

**Table S72.** CMAE (corrected mean absolute error) values for the isomers of Zoarenone at the PCM/B3LYP/6-31G**//MMFF

| | Absoluted Error Scaled | | | | | | | | | | | | | | | | |
|---|---|---|---|---|---|---|---|---|---|---|---|---|---|---|---|---|---|
| | Isomer1 | Isomer2 | Isomer3 | Isomer4 | Isomer5 | Isomer6 | Isomer7 | Isomer8 | Isomer9 | Isomer10 | Isomer11 | Isomer12 | Isomer13 | Isomer14 | Isomer15 | Isomer16 | Isomer17 |
| C 7 | 0.21 | 0.46 | 1.13 | 0.92 | 2.81 | 0.37 | 1.15 | 1.44 | 0.80 | 0.57 | 1.10 | 0.20 | 1.99 | 0.03 | 0.51 | 0.09 | 1.50 |
| C 8 | 0.39 | 0.82 | 0.47 | 2.26 | 1.90 | 0.20 | 4.09 | 1.21 | 1.03 | 1.93 | 0.10 | 1.95 | 3.36 | 0.80 | 0.51 | 2.57 | 0.49 |
| C 9 | 0.81 | 2.18 | 0.11 | 2.05 | 3.64 | 5.28 | 4.08 | 5.51 | 3.00 | 1.49 | 2.47 | 1.17 | 1.44 | 0.75 | 2.11 | 0.76 | 1.66 |
| C 10 | 6.13 | 8.11 | 7.81 | 9.33 | 7.02 | 6.31 | 5.24 | 4.55 | 4.37 | 9.63 | 7.58 | 12.56 | 9.98 | 6.89 | 7.74 | 8.48 | 6.70 |
| C 11 | 0.13 | 0.23 | 7.83 | 4.77 | 0.24 | 1.17 | 0.75 | 0.86 | 3.37 | 3.03 | 2.13 | 2.25 | 1.54 | 1.59 | 2.39 | 1.45 | 0.17 |
| C 12 | 1.38 | 5.60 | 3.18 | 6.38 | 1.35 | 2.43 | 0.89 | 0.61 | 3.81 | 12.68 | 1.52 | 11.63 | 2.77 | 0.82 | 1.04 | 6.62 | 2.38 |
| C 13 | 3.72 | 8.16 | 2.07 | 2.53 | 2.05 | 1.89 | 5.00 | 4.85 | 1.71 | 1.57 | 0.85 | 0.03 | 4.51 | 1.20 | 13.26 | 10.85 | 4.27 |
| C 14 | 5.22 | 1.35 | 1.14 | 0.80 | 1.22 | 1.56 | 3.22 | 3.51 | 4.83 | 1.34 | 0.43 | 1.79 | 0.66 | 4.98 | 1.53 | 0.08 | 6.79 |
| C 15 | 9.88 | 7.98 | 5.70 | 7.25 | 7.17 | 7.72 | 8.31 | 8.41 | 8.47 | 6.63 | 6.33 | 8.18 | 7.52 | 8.07 | 6.45 | 6.93 | 9.94 |
| C 16 | 0.01 | 0.79 | 2.14 | 1.15 | 0.30 | 0.59 | 1.12 | 0.37 | 0.60 | 0.48 | 0.09 | 0.43 | 0.56 | 1.05 | 0.11 | 1.19 | 0.35 |
| C 17 | 2.17 | 2.86 | 4.39 | 2.75 | 3.23 | 3.36 | 2.78 | 3.13 | 1.83 | 3.21 | 2.85 | 3.37 | 4.32 | 2.40 | 2.67 | 3.47 | 2.13 |
| C 18 | 0.73 | 5.68 | 1.88 | 5.52 | 3.27 | 3.31 | 2.60 | 2.85 | 3.29 | 2.87 | 4.02 | 2.04 | 4.32 | 8.20 | 3.26 | 4.81 | 2.97 |
| C 19 | 4.13 | 7.46 | 3.11 | 1.07 | 0.93 | 1.84 | 0.15 | 1.33 | 0.75 | 7.83 | 2.16 | 7.49 | 2.54 | 2.88 | 4.10 | 7.27 | 5.66 |
| C 20 | 4.06 | 6.13 | 5.22 | 4.94 | 2.23 | 1.77 | 3.94 | 3.13 | 5.61 | 4.20 | 4.20 | 2.48 | 3.23 | 4.34 | 4.32 | 5.76 | 3.81 |
| C 21 | 4.17 | 2.33 | 5.66 | 0.03 | 0.82 | 3.24 | 1.37 | 4.79 | 7.05 | 4.44 | 4.10 | 2.60 | 2.40 | 4.31 | 1.87 | 3.00 | 4.48 |
| C 22 | 2.05 | 3.31 | 1.52 | 1.22 | 2.70 | 1.99 | 3.32 | 2.14 | 2.72 | 2.22 | 3.52 | 2.45 | 0.52 | 0.49 | 4.32 | 1.56 | 0.50 |
| C 23 | 6.13 | 0.59 | 4.66 | 0.39 | 3.30 | 2.92 | 2.56 | 3.47 | 1.63 | 3.57 | 0.54 | 1.17 | 0.93 | 2.70 | 3.29 | 0.09 | 5.68 |
| C 24 | 4.01 | 0.98 | 4.16 | 0.60 | 1.47 | 1.09 | 1.43 | 0.71 | 1.09 | 2.94 | 1.03 | 2.05 | 1.49 | 3.33 | 1.06 | 1.27 | 4.57 |
| C 25 | 1.29 | 2.68 | 2.01 | 2.50 | 2.02 | 2.10 | 0.33 | 4.10 | 1.61 | 11.10 | 0.73 | 11.71 | 0.01 | 7.79 | 0.67 | 11.99 | 8.43 |
| C 27 | 4.35 | 3.74 | 4.35 | 1.40 | 1.66 | 2.30 | 1.12 | 1.92 | 1.66 | 4.94 | 1.77 | 4.16 | 1.52 | 3.67 | 2.10 | 3.87 | 5.39 |
| C 28 | 11.60 | 25.10 | 10.14 | 15.72 | 1.85 | 1.90 | 4.62 | 4.99 | 10.84 | 16.46 | 11.77 | 15.44 | 8.70 | 7.37 | 11.78 | 14.91 | 12.99 |
| C 29 | 1.64 | 0.59 | 2.07 | 0.02 | 2.53 | 0.99 | 1.38 | 0.33 | 2.23 | 2.40 | 0.16 | 3.26 | 0.08 | 1.17 | 0.60 | 0.52 | 0.61 |
| **Average** | **3.37** | **4.42** | **3.67** | **3.35** | **2.44** | **2.47** | **2.70** | **2.92** | **3.29** | **4.80** | **2.70** | **4.47** | **2.93** | **3.40** | **3.44** | **4.43** | **4.16** |

| | | | | | | | | | | | | | | | | | |
|---|---|---|---|---|---|---|---|---|---|---|---|---|---|---|---|---|---|
| H 8a | 0.65 | 0.17 | 0.53 | 0.21 | 0.69 | 0.28 | 0.77 | 0.17 | 0.75 | 0.34 | 0.59 | 0.41 | 0.24 | 0.28 | 0.71 | 0.26 | 0.44 |
| H 8b | 0.90 | 0.16 | 0.76 | 0.51 | 0.33 | 0.15 | 1.41 | 0.08 | 0.76 | 0.41 | 0.63 | 0.48 | 0.35 | 0.16 | 0.62 | 0.36 | 0.45 |
| H 11 | 0.14 | 0.01 | 0.36 | 0.11 | 0.01 | 0.11 | 0.56 | 0.56 | 0.59 | 0.43 | 0.03 | 0.04 | 0.17 | 0.15 | 0.22 | 0.09 | 0.35 |
| H 13 | 0.48 | 0.25 | 0.47 | 0.81 | 0.17 | 0.15 | 0.63 | 0.60 | 0.68 | 0.29 | 0.50 | 0.09 | 0.09 | 0.55 | 0.34 | 0.02 | 0.38 |
| H 14a | 0.15 | 0.15 | 0.75 | 0.30 | 0.19 | 0.15 | 0.24 | 0.25 | 0.46 | 0.71 | 0.01 | 0.13 | 0.10 | 0.06 | 0.04 | 0.18 | 0.43 |
| H 14b | 0.68 | 0.45 | 0.35 | 0.38 | 0.02 | 0.22 | 0.67 | 0.52 | 0.85 | 0.39 | 0.12 | 0.05 | 0.10 | 0.12 | 0.20 | 0.31 | 0.64 |
| H 16 | 0.90 | 0.04 | 0.69 | 0.37 | 0.27 | 0.23 | 0.37 | 0.41 | 0.07 | 0.19 | 0.08 | 0.22 | 0.31 | 0.64 | 0.54 | 0.40 | 0.57 |
| H 18 | 0.99 | 0.14 | 0.04 | 0.01 | 0.02 | 0.02 | 0.11 | 0.06 | 0.59 | 0.01 | 0.01 | 0.05 | 0.05 | 0.25 | 0.05 | 0.10 | 0.82 |
| H 19a | 0.38 | 0.01 | 0.42 | 0.26 | 0.22 | 0.12 | 0.23 | 0.18 | 0.24 | 0.09 | 0.25 | 0.13 | 0.36 | 0.02 | 0.04 | 0.07 | 0.51 |
| H 19b | 0.41 | 0.15 | 0.44 | 0.17 | 0.28 | 0.25 | 0.33 | 0.26 | 0.10 | 0.09 | 0.13 | 0.01 | 0.06 | 0.41 | 0.24 | 0.01 | 0.50 |
| H 21 | 0.76 | 0.14 | 0.51 | 0.03 | 0.05 | 0.11 | 0.46 | 0.24 | 0.63 | 0.31 | 0.02 | 0.02 | 0.55 | 0.40 | 0.21 | 0.31 | 0.72 |
| H 23 | 0.39 | 0.11 | 0.57 | 0.25 | 0.10 | 0.15 | 0.06 | 0.01 | 0.28 | 0.13 | 0.01 | 0.02 | 0.24 | 0.47 | 0.55 | 0.22 | 0.02 |
| H 25a-c | 0.24 | 0.12 | 0.04 | 0.12 | 0.04 | 0.22 | 0.06 | 0.22 | 0.01 | 0.15 | 0.11 | 0.15 | 0.20 | 0.18 | 0.31 | 0.11 | 0.01 |
| H 27a-c | 0.27 | 0.31 | 0.23 | 0.18 | 0.09 | 0.13 | 0.20 | 0.21 | 0.20 | 0.25 | 0.10 | 0.16 | 0.10 | 0.25 | 0.35 | 0.35 | 0.20 |
| H 28a-c | 0.05 | 0.56 | 0.21 | 0.30 | 0.19 | 0.15 | 0.00 | 0.01 | 0.09 | 0.12 | 0.23 | 0.36 | 0.25 | 0.42 | 0.31 | 0.50 | 0.26 |
| H 29a-c | 0.16 | 0.09 | 0.08 | 0.04 | 0.06 | 0.07 | 0.11 | 0.13 | 0.06 | 0.11 | 0.11 | 0.06 | 0.06 | 0.07 | 0.29 | 0.07 | 0.20 |
| **Average** | **0.47** | **0.18** | **0.40** | **0.25** | **0.17** | **0.16** | **0.39** | **0.24** | **0.40** | **0.25** | **0.18** | **0.15** | **0.20** | **0.28** | **0.31** | **0.21** | **0.41** |

| | Absoluted Error Scaled | | | | | | | | | | | | | | | | |
|---|---|---|---|---|---|---|---|---|---|---|---|---|---|---|---|---|---|
| | Isomer18 | Isomer19 | Isomer20 | Isomer21 | Isomer22 | Isomer23 | Isomer24 | Isomer25 | Isomer26 | Isomer27 | Isomer28 | Isomer29 | Isomer30 | Isomer31 | Isomer32 | Isomer44 | Isomer50 |
| C 7 | 0.57 | 0.36 | 2.30 | 0.17 | 1.12 | 0.31 | 0.35 | 2.05 | 1.44 | 0.68 | 2.67 | 0.79 | 1.25 | 0.65 | 2.28 | 0.37 | 0.03 |
| C 8 | 0.70 | 0.31 | 3.30 | 2.00 | 0.45 | 3.41 | 1.90 | 0.28 | 4.27 | 0.38 | 2.02 | 2.59 | 0.50 | 0.10 | 2.96 | 3.92 | 1.13 |
| C 9 | 0.20 | 1.20 | 0.05 | 1.27 | 2.55 | 0.29 | 3.31 | 5.33 | 3.92 | 4.53 | 3.34 | 2.81 | 0.49 | 4.12 | 2.05 | 0.53 | 1.25 |
| C 10 | 8.81 | 8.63 | 10.28 | 11.97 | 6.55 | 10.13 | 4.81 | 4.11 | 5.57 | 6.84 | 7.94 | 8.43 | 6.48 | 6.64 | 7.89 | 11.19 | 8.25 |
| C 11 | 0.51 | 8.56 | 4.06 | 1.26 | 0.23 | 5.09 | 0.75 | 3.81 | 3.61 | 3.71 | 2.78 | 0.52 | 0.28 | 1.96 | 1.82 | 2.91 | 1.59 |
| C 12 | 6.05 | 2.09 | 4.89 | 6.33 | 3.27 | 6.74 | 0.11 | 3.54 | 4.12 | 3.03 | 3.33 | 4.48 | 0.29 | 0.20 | 1.52 | 7.56 | 2.02 |
| C 13 | 10.17 | 2.91 | 2.98 | 3.47 | 1.76 | 0.95 | 5.59 | 1.51 | 1.48 | 0.67 | 0.16 | 2.34 | 2.23 | 12.08 | 11.87 | 3.74 | 16.74 |
| C 14 | 0.35 | 2.87 | 0.57 | 1.71 | 0.79 | 4.48 | 3.39 | 6.10 | 4.73 | 0.01 | 0.51 | 0.82 | 2.49 | 0.92 | 1.40 | 4.76 | 2.26 |
| C 15 | 5.63 | 6.23 | 6.88 | 8.11 | 6.86 | 9.19 | 8.10 | 8.69 | 8.79 | 7.99 | 6.91 | 7.93 | 7.23 | 6.70 | 6.59 | 7.01 | 6.27 |
| C 16 | 0.54 | 1.87 | 1.53 | 1.35 | 0.55 | 0.11 | 1.08 | 0.17 | 0.83 | 0.43 | 0.30 | 0.60 | 1.18 | 1.20 | 0.95 | 0.65 | 0.34 |
| C 17 | 2.88 | 3.82 | 3.10 | 3.46 | 3.00 | 3.22 | 2.50 | 2.70 | 1.87 | 3.20 | 2.93 | 5.32 | 3.50 | 2.87 | 2.65 | 5.04 | 4.05 |
| C 18 | 4.79 | 4.72 | 5.11 | 3.68 | 2.78 | 3.42 | 2.39 | 4.26 | 3.42 | 3.17 | 3.94 | 3.57 | 5.73 | 4.35 | 4.04 | 5.16 | 6.59 |
| C 19 | 8.46 | 5.15 | 1.39 | 2.92 | 0.64 | 1.90 | 0.03 | 0.23 | 1.00 | 6.42 | 3.67 | 7.06 | 0.87 | 4.33 | 3.98 | 4.86 | 6.92 |
| C 20 | 6.23 | 4.24 | 5.02 | 0.93 | 2.79 | 2.99 | 4.35 | 4.34 | 5.28 | 3.79 | 3.76 | 3.80 | 4.79 | 4.21 | 3.52 | 1.22 | 6.69 |
| C 21 | 0.13 | 6.04 | 2.04 | 3.15 | 1.12 | 1.78 | 2.43 | 10.11 | 7.07 | 6.87 | 2.44 | 0.17 | 4.11 | 0.87 | 2.28 | 0.23 | 2.30 |
| C 22 | 5.71 | 0.50 | 0.13 | 2.79 | 3.66 | 0.78 | 4.60 | 0.69 | 1.98 | 2.47 | 3.11 | 0.98 | 2.36 | 2.14 | 3.07 | 0.80 | 7.00 |
| C 23 | 2.90 | 5.01 | 0.81 | 0.54 | 0.87 | 2.67 | 0.31 | 3.09 | 1.57 | 4.64 | 3.96 | 2.53 | 3.63 | 0.30 | 3.00 | 1.88 | 4.70 |
| C 24 | 1.39 | 4.69 | 0.61 | 2.17 | 1.05 | 2.25 | 0.88 | 0.63 | 1.99 | 1.30 | 1.72 | 0.16 | 3.27 | 0.85 | 1.92 | 1.43 | 0.42 |
| C 25 | 0.70 | 7.07 | 0.65 | 11.90 | 0.71 | 12.63 | 0.35 | 4.20 | 0.79 | 1.99 | 2.27 | 0.03 | 0.95 | 2.98 | 0.26 | 13.88 | 2.56 |
| C 27 | 3.77 | 5.39 | 2.15 | 3.96 | 1.75 | 3.20 | 0.99 | 2.80 | 1.67 | 2.81 | 2.06 | 0.95 | 2.57 | 2.89 | 2.42 | 2.24 | 1.43 |
| C 28 | 25.40 | 12.16 | 15.63 | 2.25 | 0.51 | 4.39 | 3.66 | 12.16 | 11.24 | 15.28 | 13.76 | 10.17 | 6.57 | 13.25 | 12.04 | 3.68 | 19.29 |
| C 29 | 0.18 | 0.23 | 0.35 | 2.99 | 0.51 | 3.32 | 2.12 | 0.66 | 1.90 | 1.12 | 2.80 | 0.27 | 2.34 | 0.53 | 2.07 | 4.97 | 1.04 |
| **Average** | **4.37** | **4.28** | **3.36** | **3.56** | **1.98** | **3.78** | **2.45** | **3.70** | **3.57** | **3.70** | **3.47** | **3.02** | **2.87** | **3.37** | **3.66** | **4.00** | **4.68** |

| | | | | | | | | | | | | | | | | | |
|---|---|---|---|---|---|---|---|---|---|---|---|---|---|---|---|---|---|
| H 8a | 0.38 | 0.50 | 0.30 | 0.14 | 0.26 | 0.07 | 0.34 | 0.50 | 0.82 | 0.54 | 0.05 | 0.05 | 0.21 | 0.67 | 0.07 | 0.48 | 0.23 |
| H 8b | 0.30 | 0.58 | 0.00 | 0.01 | 0.18 | 0.16 | 0.31 | 0.45 | 1.30 | 0.65 | 0.30 | 0.17 | 0.18 | 0.63 | 0.35 | 0.45 | 0.36 |
| H 11 | 0.27 | 0.31 | 0.42 | 0.05 | 0.16 | 0.60 | 0.61 | 0.51 | 0.49 | 0.14 | 0.11 | 0.15 | 0.25 | 0.28 | 0.36 | 0.12 | 0.19 |
| H 13 | 0.15 | 0.39 | 0.77 | 0.02 | 0.18 | 0.54 | 0.59 | 0.75 | 0.77 | 0.29 | 0.41 | 0.49 | 0.36 | 0.12 | 0.05 | 0.22 | 0.13 |
| H 14a | 0.44 | 0.45 | 0.48 | 0.08 | 0.15 | 0.29 | 0.20 | 0.80 | 0.50 | 0.08 | 0.01 | 0.11 | 0.06 | 0.08 | 0.05 | 0.26 | 0.65 |
| H 14b | 0.36 | 0.22 | 0.37 | 0.04 | 0.01 | 0.38 | 0.67 | 0.90 | 0.91 | 0.02 | 0.20 | 0.35 | 0.06 | 0.61 | 0.42 | 0.59 | 0.65 |
| H 16 | 0.59 | 0.47 | 0.30 | 0.31 | 0.07 | 0.36 | 0.17 | 0.31 | 0.33 | 0.08 | 0.11 | 0.39 | 0.68 | 0.24 | 0.46 | 0.20 | 0.70 |
| H 18 | 0.28 | 0.20 | 0.19 | 0.12 | 0.02 | 0.05 | 0.02 | 0.50 | 0.60 | 0.19 | 0.09 | 0.02 | 0.22 | 0.11 | 0.01 | 0.40 | 0.32 |
| H 19a | 0.01 | 0.54 | 0.30 | 0.11 | 0.19 | 0.05 | 0.24 | 0.30 | 0.26 | 0.17 | 0.28 | 0.49 | 0.17 | 0.00 | 0.09 | 0.18 | 0.39 |
| H 19b | 0.01 | 0.53 | 0.16 | 0.03 | 0.24 | 0.02 | 0.25 | 0.22 | 0.15 | 0.25 | 0.21 | 0.92 | 0.18 | 0.11 | 0.09 | 0.13 | 0.17 |
| H 21 | 0.14 | 0.53 | 0.27 | 0.03 | 0.14 | 0.29 | 0.27 | 0.65 | 0.91 | 0.03 | 0.16 | 0.30 | 0.41 | 0.16 | 0.04 | 0.03 | 0.15 |
| H 23 | 0.62 | 0.28 | 0.45 | 0.22 | 0.09 | 0.02 | 0.22 | 0.01 | 0.16 | 0.03 | 0.08 | 0.02 | 0.62 | 0.31 | 0.62 | 0.05 | 0.63 |
| H 25a-c | 0.06 | 0.03 | 0.25 | 0.23 | 0.08 | 0.09 | 0.05 | 0.22 | 0.02 | 0.13 | 0.01 | 0.09 | 0.13 | 0.27 | 0.47 | 0.23 | 0.13 |
| H 27a-c | 0.39 | 0.12 | 0.15 | 0.16 | 0.08 | 0.17 | 0.12 | 0.31 | 0.29 | 0.14 | 0.09 | 0.23 | 0.25 | 0.39 | 0.27 | 0.27 | 0.36 |
| H 28a-c | 0.47 | 0.44 | 0.20 | 0.16 | 0.25 | 0.01 | 0.17 | 0.08 | 0.08 | 0.31 | 0.29 | 0.52 | 0.23 | 0.40 | 0.39 | 0.13 | 0.40 |
| H 29a-c | 0.26 | 0.05 | 0.09 | 0.04 | 0.09 | 0.05 | 0.12 | 0.04 | 0.22 | 0.02 | 0.10 | 0.08 | 0.01 | 0.18 | 0.12 | 0.04 | 0.32 |
| **Average** | **0.30** | **0.35** | **0.29** | **0.11** | **0.14** | **0.20** | **0.27** | **0.41** | **0.49** | **0.19** | **0.16** | **0.27** | **0.25** | **0.29** | **0.24** | **0.24** | **0.36** |

**Table S73.** CMAE (corrected mean absolute error) values for the isomers of Zoaremine at the PCM/B3LYP/6-31G**//AMBER

| | Absoluted Error Scaled | | | |
|---|---|---|---|---|
| | **Isomer1** | **Isomer2** | **Isomer3** | **Isomer4** |
| C 1 | 5.25 | 4.97 | 2.19 | 1.22 |
| C 2 | 6.67 | 6.21 | 7.25 | 8.02 |
| C 3 | 2.90 | 2.53 | 1.58 | 0.71 |
| C 4 | 0.55 | 0.29 | 2.62 | 2.53 |
| C 5 | 0.40 | 0.83 | 1.04 | 3.17 |
| C 6 | 4.16 | 3.80 | 1.24 | 0.05 |
| C 7 | 21.49 | 22.80 | 17.57 | 22.27 |
| C 8 | 3.10 | 3.37 | 2.95 | 3.00 |
| C 9 | 0.46 | 0.81 | 1.29 | 0.25 |
| C 10 | 3.40 | 1.16 | 3.15 | 0.46 |
| C 11 | 0.81 | 0.44 | 1.50 | 0.00 |
| C 12 | 0.81 | 2.89 | 0.33 | 3.33 |
| C 13 | 1.73 | 9.09 | 0.64 | 9.37 |
| C 14 | 1.08 | 3.88 | 1.09 | 4.19 |
| C 15 | 0.67 | 1.32 | 1.72 | 2.12 |
| C 16 | 0.88 | 0.33 | 1.05 | 0.30 |
| C 17 | 6.61 | 6.06 | 7.54 | 6.89 |
| C 18 | 2.57 | 1.97 | 2.46 | 2.34 |
| C 19 | 0.14 | 0.35 | 0.13 | 0.08 |
| C 20 | 1.10 | 2.75 | 0.23 | 1.75 |
| C 21 | 2.77 | 5.08 | 2.56 | 5.50 |
| C 22 | 1.87 | 1.62 | 3.44 | 1.51 |
| C 23 | 3.40 | 4.64 | 1.60 | 4.80 |
| C 24 | 0.98 | 1.89 | 1.18 | 2.79 |
| C 25 | 4.89 | 6.23 | 1.42 | 5.84 |
| C 27 | 0.49 | 0.21 | 0.19 | 0.48 |
| C 28 | 1.84 | 1.02 | 0.38 | 0.68 |
| C 29 | 1.81 | 0.69 | 1.74 | 0.45 |
| C 30 | 0.97 | 0.59 | 0.59 | 0.17 |
| **Average** | **2.89** | **3.37** | **2.44** | **3.25** |
| H 1a | 0.28 | 0.29 | 0.02 | 0.19 |
| H 1b | 0.18 | 0.18 | 0.27 | 0.30 |
| H 2 | 0.35 | 0.37 | 0.08 | 0.08 |
| H 3a | 0.20 | 0.15 | 0.47 | 0.51 |
| H 3b | 0.43 | 0.48 | 0.03 | 0.11 |
| H 4 | 0.25 | 0.22 | 0.44 | 0.55 |
| H 5a | 0.40 | 0.41 | 0.10 | 0.08 |
| H 5b | 0.18 | 0.14 | 0.11 | 0.01 |
| H 8a | 0.29 | 0.28 | 0.05 | 0.30 |
| H 8b | 0.60 | 0.53 | 0.44 | 0.59 |

| | | | | |
|---|---|---|---|---|
| H 11 | 0.20 | 0.62 | 0.08 | 0.61 |
| H 13 | 0.38 | 0.39 | 0.27 | 0.43 |
| H 14a | 0.09 | 0.36 | 0.13 | 0.34 |
| H 14b | 0.07 | 0.44 | 0.00 | 0.42 |
| H 16 | 0.38 | 0.55 | 0.21 | 0.60 |
| H 18 | 0.08 | 0.11 | 0.04 | 0.05 |
| H 19a | 0.35 | 0.41 | 0.34 | 0.46 |
| H 19b | 0.36 | 0.36 | 0.27 | 0.31 |
| H 21 | 0.40 | 0.16 | 0.36 | 0.20 |
| H 23a | 0.94 | 1.14 | 0.21 | 1.14 |
| H 23b | 0.49 | 0.63 | 0.04 | 0.63 |
| H 25a-c | 0.07 | 0.06 | 0.19 | 0.11 |
| H 27a-c | 0.00 | 0.06 | 0.07 | 0.02 |
| H 28a-c | 0.02 | 0.12 | 0.09 | 0.10 |
| H 29a-c | 0.23 | 0.07 | 0.06 | 0.01 |
| H 30a-c | 0.19 | 0.25 | 0.05 | 0.16 |
| **Average** | **0.28** | **0.34** | **0.17** | **0.32** |

**Table S74.** CMAE (corrected mean absolute error) values for the isomers of Zoaramine at the PCM/B3LYP/6-31G**//MM3

| | Absoluted Error Scaled | | | |
|---|---|---|---|---|
| | **Isomer1** | **Isomer2** | **Isomer3** | **Isomer4** |
| C 1 | 1.40 | 2.09 | 3.43 | 2.06 |
| C 2 | 1.71 | 2.47 | 1.60 | 1.33 |
| C 3 | 1.67 | 2.15 | 0.61 | 0.49 |
| C 4 | 1.75 | 2.79 | 1.96 | 1.11 |
| C 5 | 0.85 | 0.07 | 0.50 | 0.30 |
| C 6 | 21.31 | 19.41 | 20.09 | 19.98 |
| C 7 | 8.61 | 9.55 | 10.72 | 7.02 |
| C 8 | 0.79 | 2.32 | 1.06 | 5.16 |
| C 9 | 1.47 | 0.60 | 1.12 | 1.02 |
| C 10 | 6.20 | 6.01 | 5.56 | 7.63 |
| C 11 | 0.91 | 0.29 | 1.25 | 0.73 |
| C 12 | 0.41 | 3.87 | 0.33 | 2.87 |
| C 13 | 0.23 | 8.78 | 0.74 | 8.22 |
| C 14 | 0.83 | 3.97 | 0.22 | 3.11 |
| C 15 | 0.66 | 2.47 | 0.66 | 2.92 |
| C 16 | 0.71 | 2.17 | 0.98 | 2.58 |
| C 17 | 3.26 | 2.81 | 3.37 | 3.48 |
| C 18 | 2.64 | 2.63 | 2.00 | 1.82 |
| C 19 | 0.80 | 0.04 | 0.33 | 1.26 |
| C 20 | 4.11 | 5.20 | 3.96 | 4.37 |
| C 21 | 3.00 | 5.75 | 2.47 | 5.22 |
| C 22 | 0.18 | 0.83 | 0.66 | 2.10 |
| C 23 | 2.59 | 2.27 | 4.15 | 0.49 |
| C 24 | 6.37 | 6.08 | 6.41 | 8.55 |
| C 25 | 4.45 | 5.13 | 5.62 | 4.64 |
| C 27 | 1.40 | 0.66 | 0.76 | 0.44 |
| C 28 | 2.31 | 0.98 | 1.76 | 2.07 |
| C 29 | 1.60 | 1.00 | 0.88 | 1.67 |
| C 30 | 1.15 | 0.41 | 0.68 | 0.36 |
| **Average** | **2.88** | **3.54** | **2.89** | **3.55** |
| H 1a | 0.12 | 0.15 | 0.19 | 0.11 |
| H 1b | 0.24 | 0.17 | 0.15 | 0.08 |
| H 2 | 0.05 | 0.03 | 0.29 | 0.21 |
| H 3a | 0.18 | 0.23 | 0.52 | 0.52 |
| H 3b | 0.07 | 0.00 | 0.05 | 0.03 |
| H 4 | 0.01 | 0.02 | 0.39 | 0.39 |
| H 5a | 0.34 | 0.40 | 0.38 | 0.55 |
| H 5b | 0.24 | 0.10 | 0.24 | 0.11 |
| H 8a | 0.32 | 0.28 | 0.40 | 0.15 |
| H 8b | 0.60 | 0.44 | 0.74 | 0.18 |

| | | | | |
|---|---|---|---|---|
| H 11 | 0.15 | 0.77 | 0.20 | 0.76 |
| H 13 | 0.39 | 0.61 | 0.43 | 0.58 |
| H 14a | 0.17 | 0.21 | 0.13 | 0.25 |
| H 14b | 0.48 | 0.52 | 0.39 | 0.53 |
| H 16 | 0.29 | 0.45 | 0.21 | 0.14 |
| H 18 | 0.00 | 0.05 | 0.03 | 0.13 |
| H 19a | 0.27 | 0.35 | 0.22 | 0.30 |
| H 19b | 0.36 | 0.34 | 0.37 | 0.35 |
| H 21 | 0.23 | 0.37 | 0.24 | 0.33 |
| H 23a | 0.86 | 0.65 | 1.08 | 0.09 |
| H 23b | 0.28 | 0.11 | 0.56 | 1.11 |
| H 25a-c | 0.15 | 0.15 | 0.15 | 0.12 |
| H 27a-c | 0.05 | 0.07 | 0.09 | 0.05 |
| H 28a-c | 0.11 | 0.03 | 0.11 | 0.02 |
| H 29a-c | 0.20 | 0.04 | 0.22 | 0.18 |
| H 30a-c | 0.07 | 0.11 | 0.12 | 0.08 |
| **Average** | **0.24** | **0.26** | **0.30** | **0.28** |

**Table S75.** CMAE (corrected mean absolute error) values for the isomers of Zoaramine at the PCM/B3LYP/6-31G**//MMFF

| | Absoluted Error Scaled | | |
|---|---|---|---|
| | **Isomer1** | **Isomer3** | **Isomer4** |
| C 1 | 0.44 | 2.50 | 1.87 |
| C 2 | 1.94 | 5.22 | 5.06 |
| C 3 | 2.45 | 2.00 | 1.29 |
| C 4 | 2.00 | 2.14 | 1.40 |
| C 5 | 3.40 | 0.10 | 0.47 |
| C 6 | 25.41 | 21.25 | 20.50 |
| C 7 | 7.71 | 8.63 | 9.96 |
| C 8 | 3.16 | 2.23 | 3.19 |
| C 9 | 3.21 | 2.13 | 2.82 |
| C 10 | 7.20 | 6.64 | 6.10 |
| C 11 | 0.27 | 0.99 | 0.72 |
| C 12 | 2.48 | 3.19 | 0.35 |
| C 13 | 0.66 | 0.17 | 7.43 |
| C 14 | 1.57 | 0.86 | 3.82 |
| C 15 | 6.81 | 6.11 | 7.62 |
| C 16 | 0.89 | 0.59 | 0.67 |
| C 17 | 3.54 | 2.89 | 2.74 |
| C 18 | 3.17 | 2.47 | 2.47 |
| C 19 | 0.97 | 0.90 | 0.42 |
| C 20 | 2.87 | 3.36 | 4.77 |
| C 21 | 1.95 | 0.76 | 2.62 |
| C 22 | 8.08 | 5.19 | 5.57 |
| C 23 | 2.24 | 4.19 | 4.52 |
| C 24 | 15.55 | 12.17 | 12.59 |
| C 25 | 0.06 | 4.87 | 5.61 |
| C 27 | 2.21 | 1.85 | 1.26 |
| C 28 | 1.09 | 2.14 | 0.98 |
| C 29 | 2.17 | 1.13 | 1.40 |
| C 30 | 2.27 | 1.03 | 0.49 |
| **Average** | **3.99** | **3.71** | **4.09** |
| H 1a | 0.08 | 0.01 | 0.02 |
| H 1b | 0.70 | 0.45 | 0.43 |
| H 2 | 0.11 | 0.03 | 0.09 |
| H 3a | 0.02 | 0.42 | 0.45 |
| H 3b | 0.20 | 0.08 | 0.08 |
| H 4 | 0.01 | 0.36 | 0.35 |
| H 5a | 0.26 | 0.36 | 0.42 |
| H 5b | 0.14 | 0.25 | 0.27 |
| H 8a | 0.12 | 0.06 | 0.11 |
| H 8b | 0.24 | 0.66 | 0.62 |

| | | | |
|---|---|---|---|
| H 11 | 0.06 | 0.11 | 0.64 |
| H 13 | 0.13 | 0.21 | 0.59 |
| H 14a | 0.12 | 0.10 | 0.26 |
| H 14b | 0.13 | 0.18 | 0.47 |
| H 16 | 0.16 | 0.28 | 0.45 |
| H 18 | 0.04 | 0.02 | 0.01 |
| H 19a | 0.23 | 0.26 | 0.34 |
| H 19b | 0.25 | 0.28 | 0.29 |
| H 21 | 0.16 | 0.18 | 0.28 |
| H 23a | 0.16 | 0.93 | 0.91 |
| H 23b | 0.68 | 0.36 | 0.25 |
| H 25a-c | 0.13 | 0.19 | 0.19 |
| H 27a-c | 0.07 | 0.12 | 0.17 |
| H 28a-c | 0.18 | 0.10 | 0.04 |
| H 29a-c | 0.15 | 0.20 | 0.02 |
| H 30a-c | 0.01 | 0.13 | 0.19 |
| **Average** | **0.18** | **0.24** | **0.31** |

**Table S76.** i*J*/d*J*-DP4 results of Zoarenone with AMBER 12 kJ/mol MAD 0.5 Å.

| Settings | Type of data (shifts) |
|---|---|
| Default | Shielding tensors |

| Default settings | TMS | σ | ν |
|---|---|---|---|
| H | 31.830573 | 0.185 | 14.18 |
| C | 192.29325 | 2.306 | 11.38 |
| J | - | 0.992 | 3.06 |
| Slope scaling J | | 0.9509 | |
| Intercept scaling J | | -0.1405 | |

| Custom settings | TMS | σ | ν |
|---|---|---|---|
| H | 31.830573 | 0.185 | 14.18 |
| C | 192.29325 | 2.306 | 11.38 |
| J | - | 0.992 | 3.06 |
| Slope scaling J | | 0.9509 | |
| Intercept scaling J | | -0.1405 | |

| Most Likely Isomers | | |
|---|---|---|
| Rank | Isomer | Probability |
| 1º | 22 | 100.00 |
| 2º | 5 | 0.00 |
| 3º | 6 | 0.00 |
| 4º | 30 | 0.00 |

| Isomer Nº | | 1 | 2 | 3 | 4 | 5 | 6 | 7 | 8 | 9 | 10 | 11 | 12 | 13 | 14 | 15 |
|---|---|---|---|---|---|---|---|---|---|---|---|---|---|---|---|---|
| DP4 (%) | H | 0.00 | 0.00 | 0.00 | 0.00 | 0.00 | 0.00 | 0.00 | 0.00 | 0.00 | 0.00 | 0.00 | 0.00 | 0.00 | 0.00 | 0.00 |
| | C | 0.00 | 0.00 | 0.00 | 0.00 | 0.33 | 0.00 | 0.00 | 0.00 | 0.00 | 0.00 | 0.00 | 0.00 | 0.00 | 0.00 | 0.00 |
| | *H+C* | *0.00* | *0.00* | *0.00* | *0.00* | *0.00* | *0.00* | *0.00* | *0.00* | *0.00* | *0.00* | *0.00* | *0.00* | *0.00* | *0.00* | *0.00* |
| | J | 11.22 | 0.58 | 5.05 | 0.85 | 2.27 | 2.19 | 16.50 | 9.33 | 0.71 | 3.13 | 2.83 | 1.96 | 1.36 | 0.15 | 0.01 |
| | **all data** | **0.00** | **0.00** | **0.00** | **0.00** | **0.00** | **0.00** | **0.00** | **0.00** | **0.00** | **0.00** | **0.00** | **0.00** | **0.00** | **0.00** | **0.00** |
| ype | Exp | 1 | 2 | 3 | 4 | 5 | 6 | 7 | 8 | 9 | 10 | 11 | 12 | 13 | 14 | 15 |

| 16 | 17 | 18 | 19 | 20 | 21 | 22 | 23 | 24 | 25 | 26 | 27 | 28 | 29 | 30 | 31 | 32 | 33 |
|---|---|---|---|---|---|---|---|---|---|---|---|---|---|---|---|---|---|
| 0.00 | 0.00 | 0.00 | 0.00 | 0.00 | 99.96 | 0.01 | 0.00 | 0.00 | 0.00 | 0.00 | 0.00 | 0.02 | 0.00 | 0.00 | 0.00 | 0.00 | 0.00 |
| 0.00 | 0.00 | 0.00 | 0.00 | 0.00 | 0.00 | 99.67 | 0.00 | 0.00 | 0.00 | 0.00 | 0.00 | 0.00 | 0.00 | 0.00 | 0.00 | 0.00 | 0.00 |
| *0.00* | *0.00* | *0.00* | *0.00* | *0.00* | *0.00* | *100.00* | *0.00* | *0.00* | *0.00* | *0.00* | *0.00* | *0.00* | *0.00* | *0.00* | *0.00* | *0.00* | *0.00* |
| 2.21 | 8.02 | 3.21 | 2.59 | 0.96 | 0.24 | 2.40 | 0.91 | 15.93 | 0.93 | 0.62 | 1.54 | 1.95 | 0.01 | 0.26 | 0.04 | 0.06 | 0.00 |
| **0.00** | **0.00** | **0.00** | **0.00** | **0.00** | **0.00** | **100.00** | **0.00** | **0.00** | **0.00** | **0.00** | **0.00** | **0.00** | **0.00** | **0.00** | **0.00** | **0.00** | **0.00** |
| 16 | 17 | 18 | 19 | 20 | 21 | 22 | 23 | 24 | 25 | 26 | 27 | 28 | 29 | 30 | 31 | 32 | 33 |

**Table S77.** i*J*/d*J*-DP4 results of Zoarenone with MM3 12 kJ/mol MAD 0.5 Å.

| Settings | Type of data (shifts) |
|---|---|
| Default | Shielding tensors |

| Default settings | TMS | σ | ν |
|---|---|---|---|
| H | 31.830573 | 0.185 | 14.18 |
| C | 192.29325 | 2.306 | 11.38 |
| J | - | 0.992 | 3.06 |
| Slope scaling J | | 0.9509 | |
| Intercept scaling J | | -0.1405 | |

| Custom settings | TMS | σ | ν |
|---|---|---|---|
| H | 31.830573 | 0.185 | 14.18 |
| C | 192.29325 | 2.306 | 11.38 |
| J | - | 0.992 | 3.06 |
| Slope scaling J | | 0.9509 | |
| Intercept scaling J | | -0.1405 | |

| Most Likely Isomers | | |
|---|---|---|
| Rank | Isomer | Probability |
| 1º | 22 | 100.00 |
| 2º | 6 | 0.00 |
| 3º | 5 | 0.00 |
| 4º | 13 | 0.00 |

| Isomer Nº | | 1 | 2 | 3 | 4 | 5 | 6 | 7 | 8 | 9 | 10 | 11 | 12 | 13 | 14 | 15 |
|---|---|---|---|---|---|---|---|---|---|---|---|---|---|---|---|---|
| DP4 (%) | H | 0.00 | 0.00 | 0.00 | 0.00 | 0.00 | 0.02 | 0.00 | 0.00 | 0.00 | 0.00 | 0.00 | 0.23 | 0.01 | 0.00 | 0.00 |
| | C | 0.00 | 0.00 | 0.00 | 0.00 | 0.04 | 0.00 | 0.00 | 0.00 | 0.00 | 0.00 | 0.00 | 0.00 | 0.00 | 0.00 | 0.00 |
| | *H+C* | *0.00* | *0.00* | *0.00* | *0.00* | *0.00* | *0.00* | *0.00* | *0.00* | *0.00* | *0.00* | *0.00* | *0.00* | *0.00* | *0.00* | *0.00* |
| | J | 4.31 | 0.05 | 2.21 | 0.33 | 9.31 | 9.77 | 7.56 | 8.30 | 0.15 | 9.14 | 4.56 | 11.97 | 0.71 | 0.04 | 0.13 |
| | **all data** | **0.00** | **0.00** | **0.00** | **0.00** | **0.00** | **0.00** | **0.00** | **0.00** | **0.00** | **0.00** | **0.00** | **0.00** | **0.00** | **0.00** | **0.00** |
| ype | Exp | 1 | 2 | 3 | 4 | 5 | 6 | 7 | 8 | 9 | 10 | 11 | 12 | 13 | 14 | 15 |

| 16 | 17 | 18 | 19 | 20 | 21 | 22 | 23 | 24 | 25 | 26 | 27 | 28 | 29 | 30 | 31 | 32 | 33 | 34 | 35 |
|---|---|---|---|---|---|---|---|---|---|---|---|---|---|---|---|---|---|---|---|
| 0.00 | 0.00 | 0.00 | 0.00 | 0.00 | 99.26 | 0.44 | 0.00 | 0.00 | 0.00 | 0.00 | 0.00 | 0.03 | 0.00 | 0.00 | 0.00 | 0.00 | 0.00 | 0.00 | 0.00 |
| 0.00 | 0.00 | 0.00 | 0.00 | 0.00 | 0.00 | 99.96 | 0.00 | 0.00 | 0.00 | 0.00 | 0.00 | 0.00 | 0.00 | 0.00 | 0.00 | 0.00 | 0.00 | 0.00 | 0.00 |
| *0.00* | *0.00* | *0.00* | *0.00* | *0.00* | *0.00* | *100.00* | *0.00* | *0.00* | *0.00* | *0.00* | *0.00* | *0.00* | *0.00* | *0.00* | *0.00* | *0.00* | *0.00* | *0.00* | *0.00* |
| 1.18 | 2.69 | 0.38 | 1.32 | 0.40 | 1.17 | 8.77 | 1.83 | 6.64 | 0.27 | 0.18 | 1.41 | 2.65 | 0.00 | 0.17 | 1.25 | 1.14 | 0.00 | 0.00 | 0.00 |
| **0.00** | **0.00** | **0.00** | **0.00** | **0.00** | **0.00** | **100.00** | **0.00** | **0.00** | **0.00** | **0.00** | **0.00** | **0.00** | **0.00** | **0.00** | **0.00** | **0.00** | **0.00** | **0.00** | **0.00** |
| 16 | 17 | 18 | 19 | 20 | 21 | 22 | 23 | 24 | 25 | 26 | 27 | 28 | 29 | 30 | 31 | 32 | 33 | 34 | 35 |

**Table S78.** i*J*/d*J*-DP4 results of Zoarenone with MMFF 12 kJ/mol MAD 0.5 Å.

| Settings | Type of data (shifts) |
|---|---|
| Default | Shielding tensors |

| Default settings | TMS | σ | ν |
|---|---|---|---|
| H | 31.830573 | 0.185 | 14.18 |
| C | 192.29325 | 2.306 | 11.38 |
| J | - | 0.992 | 3.06 |
| Slope scaling J | | 0.9509 | |
| Intercept scaling J | | -0.1405 | |

| Custom settings | TMS | σ | ν |
|---|---|---|---|
| H | 31.830573 | 0.185 | 14.18 |
| C | 192.29325 | 2.306 | 11.38 |
| J | - | 0.992 | 3.06 |
| Slope scaling J | | 0.9509 | |
| Intercept scaling J | | -0.1405 | |

| Most Likely Isomers | | |
|---|---|---|
| Rank | Isomer | Probability |
| 1º | 22 | 99.98 |
| 2º | 6 | 0.02 |
| 3º | 5 | 0.00 |
| 4º | 11 | 0.00 |

| Isomer Nº | | 1 | 2 | 3 | 4 | 5 | 6 | 7 | 8 | 9 | 10 | 11 | 12 | 13 | 14 | 15 |
|---|---|---|---|---|---|---|---|---|---|---|---|---|---|---|---|---|
| DP4 (%) | H | 0.00 | 0.01 | 0.00 | 0.00 | 0.02 | 0.87 | 0.00 | 0.00 | 0.00 | 0.00 | 0.00 | 0.33 | 0.00 | 0.00 | 0.00 |
| | C | 0.00 | 0.00 | 0.00 | 0.00 | 0.44 | 0.24 | 0.01 | 0.00 | 0.00 | 0.00 | 0.00 | 0.00 | 0.00 | 0.00 | 0.00 |
| | *H+C* | *0.00* | *0.00* | *0.00* | *0.00* | *0.00* | *0.03* | *0.00* | *0.00* | *0.00* | *0.00* | *0.00* | *0.00* | *0.00* | *0.00* | *0.00* |
| | J | 5.45 | 0.82 | 6.06 | 0.18 | 5.14 | 3.47 | 14.11 | 13.01 | 0.10 | 9.52 | 3.29 | 5.09 | 0.63 | 0.00 | 0.02 |
| | **all data** | **0.00** | **0.00** | **0.00** | **0.00** | **0.00** | **0.02** | **0.00** | **0.00** | **0.00** | **0.00** | **0.00** | **0.00** | **0.00** | **0.00** | **0.00** |
| Type | Exp | 1 | 2 | 3 | 4 | 5 | 6 | 7 | 8 | 9 | 10 | 11 | 12 | 13 | 14 | 15 |

| 16 | 17 | 18 | 19 | 20 | 21 | 22 | 23 | 24 | 25 | 26 | 27 | 28 | 29 | 30 | 31 | 32 | 33 | 34 |
|---|---|---|---|---|---|---|---|---|---|---|---|---|---|---|---|---|---|---|
| 0.00 | 0.00 | 0.00 | 0.00 | 0.00 | 91.69 | 6.68 | 0.00 | 0.00 | 0.00 | 0.00 | 0.00 | 0.38 | 0.00 | 0.00 | 0.00 | 0.00 | 0.00 | 0.00 |
| 0.00 | 0.00 | 0.00 | 0.00 | 0.00 | 0.00 | 99.19 | 0.00 | 0.12 | 0.00 | 0.00 | 0.00 | 0.00 | 0.00 | 0.00 | 0.00 | 0.00 | 0.00 | 0.00 |
| *0.00* | *0.00* | *0.00* | *0.00* | *0.00* | *0.00* | *99.97* | *0.00* | *0.00* | *0.00* | *0.00* | *0.00* | *0.00* | *0.00* | *0.00* | *0.00* | *0.00* | *0.00* | *0.00* |
| 0.69 | 2.86 | 0.79 | 2.66 | 0.50 | 0.21 | 5.04 | 0.79 | 13.17 | 0.18 | 0.09 | 2.63 | 3.23 | 0.00 | 0.03 | 0.15 | 0.08 | 0.00 | 0.00 |
| **0.00** | **0.00** | **0.00** | **0.00** | **0.00** | **0.00** | **99.98** | **0.00** | **0.00** | **0.00** | **0.00** | **0.00** | **0.00** | **0.00** | **0.00** | **0.00** | **0.00** | **0.00** | **0.00** |
| 16 | 17 | 18 | 19 | 20 | 21 | 22 | 23 | 24 | 25 | 26 | 27 | 28 | 29 | 30 | 31 | 32 | 33 | 34 |

**Table S79.** i*J*/d*J*-DP4 results of Zoaramine with AMBER 12 kJ/mol MAD 0.5 Å.

| Settings | Type of data (shifts) |
|---|---|
| Default | Shielding tensors |

| Default settings | TMS | σ | ν | Custom settings | TMS | σ | ν | Most Likely Isomers: Rank | Isomer | Probability |
|---|---|---|---|---|---|---|---|---|---|---|
| H | 31.830573 | 0.185 | 14.18 | H | 31.830573 | 0.185 | 14.18 | 1º | 3 | 100.00 |
| C | 192.29325 | 2.306 | 11.38 | C | 192.29325 | 2.306 | 11.38 | 2º | 1 | 0.00 |
| J | - | 0.992 | 3.06 | J | - | 0.992 | 3.06 | 3º | 4 | 0.00 |
| Slope scaling J | 0.9509 | | | Slope scaling J | 0.9509 | | | 4º | 2 | 0.00 |
| Intercept scaling J | -0.1405 | | | Intercept scaling J | -0.1405 | | | | | |

| Isomer Nº | | 1 | 2 | 3 | 4 | 5 | 6 | 7 | 8 | 9 | 10 | 11 | 12 | 13 | 14 | 15 |
|---|---|---|---|---|---|---|---|---|---|---|---|---|---|---|---|---|
| DP4 (%) | H | 0.00 | 0.00 | 100.00 | 0.00 | - | - | - | - | - | - | - | - | - | - | - |
| | C | 0.05 | 0.00 | 99.95 | 0.00 | - | - | - | - | - | - | - | - | - | - | - |
| | *H+C* | *0.00* | *0.00* | *100.00* | *0.00* | - | - | - | - | - | - | - | - | - | - | - |
| | J | 3.74 | 2.22 | 17.64 | 76.40 | - | - | - | - | - | - | - | - | - | - | - |
| | **all data** | **0.00** | **0.00** | **100.00** | **0.00** | - | - | - | - | - | - | - | - | - | - | - |
| Type | Exp | 1 | 2 | 3 | 4 | 5 | 6 | 7 | 8 | 9 | 10 | 11 | 12 | 13 | 14 | 15 |

**Table S80.** i*J*/d*J*-DP4 results of Zoaramine with MM3 12 kJ/mol MAD 0.5 Å.

| Settings | Type of data (shifts) |
|---|---|
| Default | Shielding tensors |

| Default settings | TMS | σ | ν | Custom settings | TMS | σ | ν | Most Likely Isomers: Rank | Isomer | Probability |
|---|---|---|---|---|---|---|---|---|---|---|
| H | 31.830573 | 0.185 | 14.18 | H | 31.830573 | 0.185 | 14.18 | 1º | 1 | 100.00 |
| C | 192.29325 | 2.306 | 11.38 | C | 192.29325 | 2.306 | 11.38 | 2º | 2 | 0.00 |
| J | - | 0.992 | 3.06 | J | - | 0.992 | 3.06 | 3º | 3 | 0.00 |
| Slope scaling J | 0.9509 | | | Slope scaling J | 0.9509 | | | 4º | 4 | 0.00 |
| Intercept scaling J | -0.1405 | | | Intercept scaling J | -0.1405 | | | | | |

| Isomer Nº | | 1 | 2 | 3 | 4 | 5 | 6 | 7 | 8 | 9 | 10 | 11 | 12 | 13 | 14 | 15 |
|---|---|---|---|---|---|---|---|---|---|---|---|---|---|---|---|---|
| DP4 (%) | H | 99.48 | 0.52 | 0.00 | 0.00 | - | - | - | - | - | - | - | - | - | - | - |
| | C | 78.71 | 0.00 | 21.29 | 0.00 | - | - | - | - | - | - | - | - | - | - | - |
| | *H+C* | *100.00* | *0.00* | *0.00* | *0.00* | - | - | - | - | - | - | - | - | - | - | - |
| | J | 86.89 | 13.05 | 0.05 | 0.01 | - | - | - | - | - | - | - | - | - | - | - |
| | **all data** | **100.00** | **0.00** | **0.00** | **0.00** | - | - | - | - | - | - | - | - | - | - | - |
| Type | Exp | 1 | 2 | 3 | 4 | 5 | 6 | 7 | 8 | 9 | 10 | 11 | 12 | 13 | 14 | 15 |

**Table S81.** i*J*/d*J*-DP4 results of Zoaramine with MMFF 12 kJ/mol MAD 0.5 Å.

| Settings | Type of data (shifts) |
|---|---|
| Default | Shielding tensors |

| Default settings | TMS | σ | ν | Custom settings | TMS | σ | ν | Most Likely Isomers: Rank | Isomer | Probability |
|---|---|---|---|---|---|---|---|---|---|---|
| H | 31.830573 | 0.185 | 14.18 | H | 31.830573 | 0.185 | 14.18 | 1º | 1 | 100.00 |
| C | 192.29325 | 2.306 | 11.38 | C | 192.29325 | 2.306 | 11.38 | 2º | 2 | 0.00 |
| J | - | 0.992 | 3.06 | J | - | 0.992 | 3.06 | 3º | 3 | 0.00 |
| Slope scaling J | 0.9509 | | | Slope scaling J | 0.9509 | | | 4º | - | - |
| Intercept scaling J | -0.1405 | | | Intercept scaling J | -0.1405 | | | | | |

| Isomer Nº | | 1 | 2 | 3 | 4 | 5 | 6 | 7 | 8 | 9 | 10 | 11 | 12 | 13 | 14 | 15 |
|---|---|---|---|---|---|---|---|---|---|---|---|---|---|---|---|---|
| DP4 (%) | H | 100.00 | 0.00 | 0.00 | - | - | - | - | - | - | - | - | - | - | - | - |
| | C | 1.57 | 98.41 | 0.03 | - | - | - | - | - | - | - | - | - | - | - | - |
| | *H+C* | *100.00* | *0.00* | *0.00* | - | - | - | - | - | - | - | - | - | - | - | - |
| | J | 99.76 | 0.15 | 0.09 | - | - | - | - | - | - | - | - | - | - | - | - |
| | **all data** | **100.00** | **0.00** | **0.00** | - | - | - | - | - | - | - | - | - | - | - | - |
| Type | Exp | 1 | 2 | 3 | 4 | 5 | 6 | 7 | 8 | 9 | 10 | 11 | 12 | 13 | 14 | 15 |
